# Divergent Syntheses of Near‐Infrared Light‐Activated Molecular Jackhammers for Cancer Cell Eradication

**DOI:** 10.1002/advs.202405965

**Published:** 2024-10-13

**Authors:** Bowen Li, Ciceron Ayala‐Orozco, Tengda Si, Lixin Zhou, Zicheng Wang, Angel A. Martí, James M. Tour

**Affiliations:** ^1^ Department of Chemistry Rice University Houston TX 77005 USA; ^2^ Department of Bioengineering Rice University Houston TX 77005 USA; ^3^ Department of Materials Science and Nanoengineering Rice University Houston TX 77005 USA; ^4^ Smalley‐Curl Institute Rice University Houston TX 77005 USA; ^5^ NanoCarbon Center and the Rice Advanced Materials Institute Rice University Houston TX 77005 USA

**Keywords:** aminocyanines, molecular jackhammers, structure‐activity relationships, vibronic‐driven action

## Abstract

Aminocyanines incorporating Cy7 and Cy7.5 moieties function as molecular jackhammers (MJH) through vibronic‐driven action (VDA). This mechanism, which couples molecular vibrational and electronic modes, results in picosecond‐scale concerted stretching of the entire molecule. When cell‐associated and activated by near‐infrared light, MJH mechanically disrupts cell membranes, causing rapid necrotic cell death. Unlike photodynamic and photothermal therapies, the ultrafast vibrational action of MJH is unhindered by high concentrations of reactive oxygen species scavengers and induces only a minimal temperature increase. Here, the efficient synthesis of a library of MJH is described using a practical approach to access a key intermediate and facilitating the preparation of various Cy7 and Cy7.5 MJH with diverse side chains in moderate to high yields. Photophysical characterization reveals that structural modifications significantly affect molar extinction coefficients and quantum yields while maintaining desirable absorption and emission wavelengths. The most promising compounds, featuring dimethylaminoethyl and dimethylcarbamoyl substitutions, demonstrate up to sevenfold improvement in phototherapeutic index compared to Cy7.5 amine across multiple cancer cell lines. This synthetic strategy provides a valuable platform for developing potent, light‐activated therapeutic agents for cancer treatment, with potentially broad applicability across various cancer types.

## Graphical abstract

A library of near‐infrared light‐activated molecular jackhammers is synthesized and their photophysical characteristics are analyzed. The most active candidates are potent against multiple cancer cell lines through vibronic‐driven action, a coupling of molecular vibrational and electronic modes over the entire molecule.

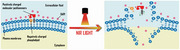

## 1 Introduction

Cancer, a life‐threatening malignant disease encompassing over 100 different types, ranks as the second leading cause of death globally.^[^ ^1^, ^2^, ^3^, ^4^ ^]^ In recent years, phototherapy has emerged as a minimally invasive treatment for cancer, offering improved tissue specificity^[^ ^5^, ^6^, ^7^, ^8^ ^]^ and significantly reduced side effects compared to traditional cancer therapies.^[^ ^9^, ^10^, ^11^ ^]^ Phototherapy comprises two main categories: photodynamic therapy (PDT)^[^ ^12^, ^13^, ^14^, ^15^, ^16^, ^17^, ^18^ ^]^ and photothermal therapy (PTT).^[^ ^19^, ^20^, ^21^, ^22^ ^]^ PDT relies on photosensitizers that generate reactive oxygen species upon light activation, while PTT utilizes materials that convert light energy into localized heat.^[^ ^23^, ^24^, ^25^ ^]^ Despite their promise, both approaches face limitations. The efficacy of PDT and PTT can be constrained by limited tissue penetration of certain wavelengths, potential photosensitivity in PDT patients, and the risk of thermal damage to surrounding healthy tissue in PTT. Moreover, the oxygen dependence of PDT presents challenges in hypoxic tumor microenvironments.^[^ ^26^, ^27^, ^28^ ^]^ Our approach aims to expand upon PDT and PTT by providing a mechanical mode of action that operates independently of oxygen levels and does not rely on heat generation. This mechanism offers a complementary strategy for light‐activated cancer therapy that will be better suited in some cases.

**Figure 1:**
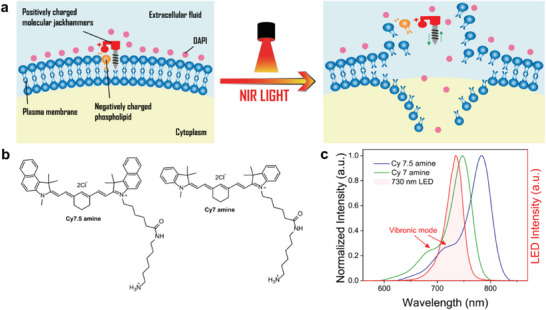
Molecular jackhammer model for cell membrane disruption, chemical structures, and UV–vis spectrum. a) Proposed mechanisms of MJH‐induced cell membrane disruption. b) Chemical structures of Cy 7 amine and Cy 7.5 amine. c) Absorption spectra of Cy 7 and Cy7.5 amines (1 µm in methanol), highlighting the vibronic mode (absorption shoulder at shorter wavelengths indicated by the arrows). The 730 nm LED intensity spectrum (obtained from Prizmatix) is overlaid, demonstrating selective stimulation of the vibronic mode in Cy7.5 amine but not in Cy7 amine, despite significant overlap with the main absorption band of Cy7 amine.

We recently introduced the concept of vibronic‐driven action (VDA) using Cy7.5 amine.^[^ ^29^ ^]^ VDA leverages the mechanical action resulting from coupling the molecule's vibrational and electronic mode,^[^ ^30^, ^31^ ^]^ to create a molecular plasmon.^[^ ^32^, ^33^, ^34^ ^]^ This coupling induces longitudinal and axial stretching of the molecules, resulting in a mechanical action akin to that of a jackhammer, hence the term molecular jackhammers (MJH).^[^ ^29^ ^]^ This approach has shown remarkable efficiency in eradicating human melanoma cells by mechanically disassembling cell membranes through VDA, a mechanism distinctly different from PDT and PTT (**Figure** **1**a).^[^ ^29^, ^35^ ^]^ Accordingly, the mechanical effect of MJH on cell membranes is neither impeded by high doses of ROS inhibitors nor does it induce an increase in media temperature.^[^ ^29^, ^36^ ^]^ The mode of action of MJH could offer an interesting approach to cancer therapy that overcomes limitations of existing phototherapies, potentially offering a more universally applicable and efficient method for cancer cell eradication.

Cyanine dyes are characterized by two nitrogen‐containing heterocycles, one of which carries a positive charge and is conjugated through a polymethine bridge to the other nitrogen center.^[^ ^37^, ^38^, ^39^, ^40^ ^]^ These dyes exhibit unique properties, including high molar extinction coefficients and narrow absorption bands. The chemical structure of Cy 7 and Cy 7.5 amines, shown in Figure 1b, feature cyclohexene rings in the polymethine bridge that enhance structural rigidity and stability, thereby improving photostability and quantum yield.

The absorption spectra of Cy 7 and Cy 7.5 amines (Figure 1c) feature a sharp and intense absorption band in the near‐infrared (NIR) region, with an additional vibronic shoulder located at higher energies (lower wavelengths).^[^ ^29^, ^41^ ^]^ The absorption of NIR light activates the vibronic mode in the molecule, coupling collective electronic excitation and vibrational states. This phenomenon is supported by density functional theory (DFT) calculations and Raman spectroscopy studies.^[^ ^29^, ^35^ ^]^ The vibronic mode exhibits a vibrational frequency of ≈1370 cm^−1^, which remains nearly constant among Cy7 and Cy7.5 molecules. This frequency corresponds to ultrafast oscillations of 25 femtoseconds or 41 terahertz. These molecular motions in the terahertz range underscore the extremely rapid nature of the mechanical action of MJH, which is crucial for their ability to open cell membranes.^[^ ^36^ ^]^ When associated with lipid membranes, these molecular vibrations can exert substantial mechanical forces. Theoretical calculations suggest that if only 10% of the energy carried by a 730 nm photon is converted to mechanical force by MJH (i.e., *F* = 0.027 nN, stress = 27 mN m^−1^), it would be sufficient to rupture the membrane. This aligns with experimental data showing that the mechanical stress needed to rupture most membranes is 1–30 mN m^−1^.^[^ ^35^, ^36^ ^]^ Notably, experimental measurements of the vibrational energy associated with the whole‐molecule oscillation (≈1370 cm^−1^) correspond to ≈164 meV. When this energy is converted to mechanical work, it results in a force of 0.026 nN and a stress of 26 mN m^−1^, which falls within the range capable of rupturing cellular membranes. This finding further supports the membrane‐disrupting capability of MJH.^[^ ^35^ ^]^

The vibronic shoulder represents a collective vibrational mode that spreads throughout the length and width of the molecule. This is supported by our time‐dependent density functional theory (TD‐DFT) calculations, which show that the shoulder band (experimentally at 730 nm, calculated at 750 nm) exhibits the strongest vibronic character among all observed spectral features.^[^ ^29^ ^]^ The vibronic shoulder also plays a role in the structure‐activity relationships we observed. Molecules with extended π‐conjugation and fused aromatic rings, which enhance the coupling between longitudinal and transversal plasmon modes, showed improved VDA activity.^[^ ^29^ ^]^ Furthermore, this suggests that the vibronic shoulder acts as a spectroscopic signature of effective plasmon‐phonon coupling in these molecules

In our previous work, we combined experimental results with theoretical calculations and observed a vibronic mode at 680 nm for Cy 7 amine and 730 nm for Cy7.5 amine. This observation supports the hypothesis that Cy7.5 amine (excitation at 730 nm) is more efficient than Cy 7 amine (excitation at 680 nm) in disrupting the cell membrane of A375 melanoma cells, likely due to the higher plasmonicity of benzoindole in the former compared to indole in the latter.^[^ ^29^, ^35^ ^]^ Plasmonicity, in this context, refers to the molecule's capacity to support collective electron density oscillations upon optical excitation. A higher plasmonicity index indicates a stronger coupling between the molecule's electronic and vibrational modes, resulting in more efficient vibronic‐driven action. This property is crucial for the effectiveness of MJH in mechanically disrupting cell membranes upon near‐infrared light activation and this effect has been quantitated.^[^ ^29^, ^35^ ^]^

Despite the recent progress in biomedical applications of Cy 7 and Cy 7.5 amines, the practical and systematic construction of these molecules remains challenging.^[^ ^42^, ^43^, ^44^ ^]^ We hypothesized that synthesizing the corresponding cyclic glutacondianil immediate **2a** (as shown in **Table** **1**) and its subsequent substitution would provide a straightforward approach to these compounds. However, achieving this is challenging due to the instability of **2a**. In a recent work by Burgess and collaborators, **2a** was successfully synthesized through a Vilsmeier–Haack reaction starting from cyclohexene, albeit under harsher conditions that resulted in lower yields.^[^ ^42^ ^]^

**Table 1:** Optimization for the synthesis of key intermediate 2 from salt 1.^[^ ^45^, ^46^ ^]^

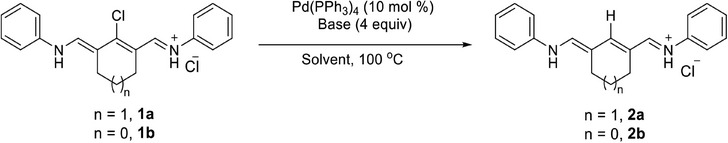				
Entry^a)^	N	Base	Solvent	Yield [%]^b)^
1	1	K_3_PO_4_	DMF/H_2_O (5:1)	31
2	1	K_2_HPO_4_	DMF/H_2_O (5:1)	30
3	1	KF	DMF/H_2_O (5:1)	25
4	1	K_2_CO_3_	DMF/H_2_O (5:1)	32
5	1	NaO*t*Bu	DMF/H_2_O (5:1)	0
6	1	NaOAc	DMF/H_2_O (5:1)	0
7	1	CsF	DMF/H_2_O (5:1)	3
8	1	Cs_2_CO_3_	DMF/H_2_O (5:1)	10
9	1	K_2_CO_3_	DMF/H_2_O/IPA (5:1:1)	35
10	1	K_2_CO_3_	EtOH	37
11	1	K_2_CO_3_	IPA	66 (61)^c)^
12^d)^	1	K_2_CO_3_	IPA	77
13^e)^	1	K_2_CO_3_	IPA	68
14^f)^	0	K_2_CO_3_	IPA	75
15	2	K_2_CO_3_	IPA	Trace

^a)^ The reactions were carried out with conditions: 1) **1** (0.25 mmol, 1 equiv), Pd(PPh_3_)_4_ (10 mol %), base (4 equiv) in solvent at 100 °C for 24 h;

^b)^ yields were determined by HPLC on a C18 reverse‐phase column;

^c)^ isolated yield;

^d)^ 48 h, isolated yield;

^e)^ **1a** (8.38 mmol), 48 h, isolated yield;

^f)^ **1b** (2.91 mmol), 48 h, isolated yield.

In this work, we describe a synthetic approach for obtaining various Cy7 and 7.5 MJH incorporating diverse substituents on their heterocyclic groups. These analogs were successfully and efficiently applied to eradicate several cancer cell lines through VDA under NIR light irradiation.

## 2 Results and Discussion

We began our investigation of dehalogenation reaction using Schiff base **1a** as a model substate (Table 1). Encouragingly, we observed the formation of product **2a** from these substrates, achieving a 31% yield when using 10 mol% of Pd(PPh_3_)_4_ as a catalyst and DMF/H_2_O as the solvent. Initially, we systematically assessed various bases (Table 1, entries 1–8) and found that K_2_CO_3_ provided a superior yield of **2a**. In contrast, using a stronger base (Table 1, entry 5) or a weaker base (Table 1, entry 6) did not produce the desired product **2a**. Further investigation into various solvents (entries 9–11) revealed that isopropyl alcohol (IPA), which likely serves as a hydrogen source due to its β‐hydrogen, was highly effective in promoting the dechlorination reaction, resulting in a 66% yield (61% isolated yield, Table 1, entry 11). Extending the reaction time from 24 h to 48 h increased the yield to 77% (Table 1, entry 12). This strategy demonstrated scalability, affording multigram‐scale product **2a** with 68% yield (Table 1, entry 13). The same protocol was successful in synthesizing the five‐membered product **2b** with good yield (Table 1, entry 14). However, applying the reaction scheme to the seven‐membered substrate resulted in only trace amounts of product (Table 1, entry 15), presumably due to its twisted conformation.

Having established the key intermediate **2**, we investigated the scope of substitutions, as detailed in **Table** **2**. The symmetrical analogs (**4a** to **4o)** were synthesized in a one‐step reaction between **2** and heterocyclic salts **3** in the presence of NaOAc at 80 °C in EtOH,^[^ ^47^, ^48^, ^49^, ^50^ ^]^ all in satisfactory yields. Various functional substituents, such as methyl (**4a)**, carboxylic acid (**4i)**, ester (**4j**), dimethylamino (**4h** and **4k**), 1,3‐dioxoisoindolin‐2‐yl (**4l**), and dimethylcarbamoyl (**4g**) were well tolerated, forming the corresponding products in moderate to good yields (54‐84%). We subsequently assessed the reactivity of identical substitutions on side chains bearing different heterocyclics. A benzoindolium salt produced product **4b** in 79% yield. Products **4c** and **4d**, formed from quinolinium and indolium‐based heterocycles, respectively, were obtained in good yields (70% and 83%). Synthesis of pyrrolidine‐based heterocycle was also compatible with the current protocol, providing the desired product **4e** with a 50% yield.

**Table 2:** Synthesis of the Cy7/Cy7.5 amine analogs 4 from *N*‐heterocyclic salt 3/3’ and key intermediate 2.^a^ ^)^

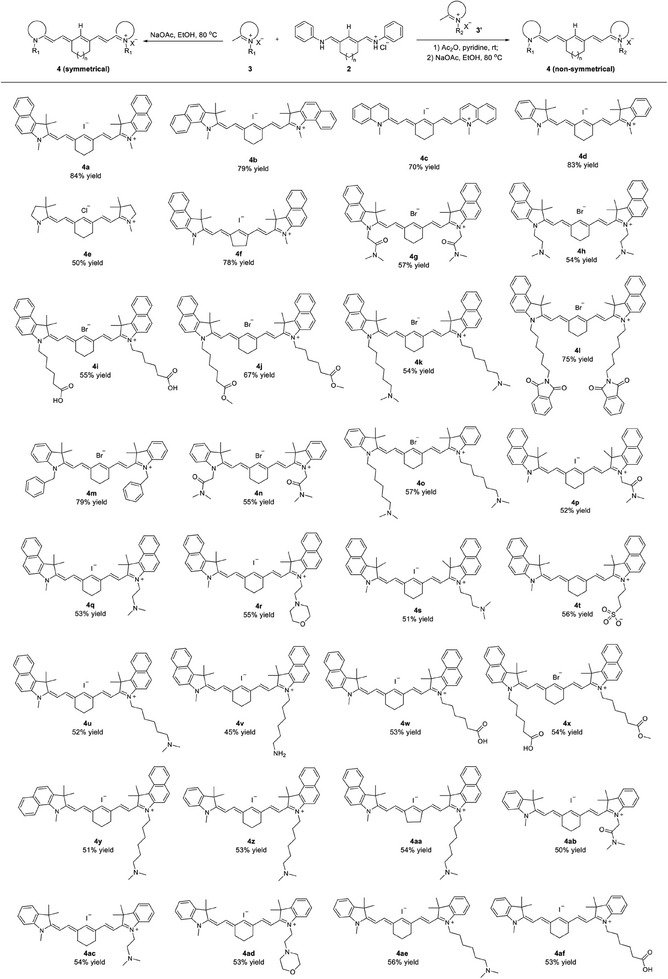

^a)^ Reaction conditions for the synthesis of symmetrical **4** (**4a**‐**4o**): **3** (2.0 equiv), **2** (1.0 equiv), and NaOAc (3.0 equiv) in EtOH at 80 °C; nonsymmetrical **4** (**4p**‐**4af**): 1) **3** (1.0 equiv), **2** (1.0 equiv) in Ac_2_O and pyridine at rt; 2) **3’** (1.0 equiv), and NaOAc (3.0 equiv) in EtOH at 80 °C; isolated yields.

The synthesis of nonsymmetric analogs from **4p** to **4af** involved two steps: first, reacting equimolar amounts of **2** and heterocyclic salts **3** in Ac_2_O and pyridine to obtain the corresponding acetylated precursors. These precursors were then condensed with equimolar amounts of heterocycles with various substitutions **3’**.^[^ ^51^, ^52^, ^53^ ^]^ Likewise, the products featuring several *N*‐alkyl substitutions, including dimethylcarbamoyl (**4p**), dimethylamino (**4q**, **4s** and **4u**), morpholino (**4r**), and sulfonate (**4t**), were also obtained through this condensation sequence, albeit in moderate yields (51–56%) due to the inevitable formation of competing symmetrical byproducts. The substrate with an aminohexyl substituent delivered **4v** in a lower yield (45%). In addition, employing identical *N‐*alkyl substitutions for both indolium‐based salts (**4ab**‐**4af**) and benzoindoles, resulted in similar yields. Products featuring distinct heterocyclics, specifically **4y** from a benzoindolium salt with different positions and **4z** from an indolium salt, were achieved with yields of 51% and 53%, respectively. Analogs containing five‐membered rings (**4f** and **4aa**) were also synthesized with yields of 78% and 54%, respectively, comparable to those of six‐membered rings.

### 2.1 Photophysical Characterization

The photophysical properties,^[^ ^41^, ^43^ ^]^ including absorption (λ_abs_) maxima, emission (λ_em_) maxima, molar extinction coefficients (ε), and quantum yields (*Φ_F_*), for all synthesized molecules, were determined in methanol (Figures s1–s34, Supporting Information), and the summarized data are presented in **Table** **3**. The absorption spectra of the molecules exhibited one major absorption band with maxima ranging from 744 to 748 nm for Cy 7 dyes and 780–788 nm for Cy7.5 dyes. Similar features were observed in the emission spectra, with maxima at 766–773 nm for Cy 7 dyes and 802–816 nm for Cy7.5 dyes. Small Stokes shifts, ranging from 15 to 26 cm^−1^, were also observed.

**Table 3:** Photophysical Properties of the Prepared Cyanine Dyes.

Cyanine dyes	*λ*_max_[abs]/nm	*λ*_max_[em]/nm	*∆λ*/nm	*ε*_max_ ^a)^	*Φ*_F_
**ICG**	784	812	28	2.33 × 10^5^	0.063
**4a**	780	801	21	2.55 × 10^5^	0.074
**4b**	791	816	25	2.82 × 10^5^	0.027
**4c**	789	815	26	1.61 × 10^5^	0.037
**4d**	744	765	21	2.04 × 10^5^	0.130
**4e**	648	668	20	0.80 × 10^5^	0.052
**4f**	818	837	19	2.02 × 10^5^	0.037
**4g**	780	804	24	3.24 × 10^5^	0.077
**4h**	785	809	24	2.41 × 10^5^	0.074
**4i**	787	808	21	3.12 × 10^5^	0.089
**4j**	785	808	23	2.29 × 10^5^	0.072
**4k**	788	809	21	2.39 × 10^5^	0.078
**4l**	788	808	20	2.35 × 10^5^	0.091
**4m**	752	773	21	2.63 × 10^5^	0.130
**4n**	745	767	22	3.26 × 10^5^	0.125
**4o**	751	772	21	2.03 × 10^5^	0.123
**4p**	780	802	22	2.49 × 10^5^	0.063
**4q**	782	806	24	2.33 × 10^5^	0.073
**4r**	781	807	26	2.82 × 10^5^	0.077
**4s**	783	804	21	2.35 × 10^5^	0.066
**4t**	783	804	21	2.17 × 10^5^	0.064
**4u**	785	804	19	2.43 × 10^5^	0.065
**4v**	784	804	20	2.02 × 10^5^	0.061
**4w**	783	804	21	2.69 × 10^5^	0.075
**4x**	787	808	21	2.84 × 10^5^	0.085
**4y**	787	812	25	2.26 × 10^5^	0.033
**4z**	765	787	22	2.95 × 10^5^	0.079
**4aa**	823	838	15	2.20 × 10^5^	0.040
**4ab**	744	766	22	2.62 × 10^5^	0.125
**4ac**	746	768	22	2.54 × 10^5^	0.111
**4ad**	748	769	21	2.81 × 10^5^	0.117
**4ae**	747	768	21	2.38 × 10^5^	0.129
**4af**	748	769	21	2.58 × 10^5^	0.139

^a)^ All values were determined using 1 µm cyanine dye in methanol at 25 °C. *ε*_max_ is in mol^−1^ L cm^−1^. Quantum yields were determined using ICG (*Φ*_F_ = 0.13 in DMSO) as standard.

In general, the presence of different side chains in these compounds did not significantly affect the maximum absorption wavelength or the shape of the absorption curves, except for notable changes in the molar extinction coefficients of **4g** and **4i** of up to ≈3 × 10^5^ m ^−1^cm^−1^. The substitution of side chains with different heterocyclic dyes substantially influenced the absorption maxima, while the Stokes shifts appeared relatively insensitive to the various side chains. The nonsymmetric molecule **4z**, derived from indolium and benzoindolium salts, exhibited a maximum absorption wavelength between that of Cy 7 and Cy 7.5 dyes, specifically at 765 nm. Cyclopentene rings in **4f** and **4aa** induced a bathochromic shift of the maximum absorption to 818 nm and 823 nm, respectively, compared to the cyclohexene rings in **4a** and **4u**. Conversely, the presence of the pyrrolidine group in **4e** resulted in a blue shift of the maximum to 648 nm. Moreover, most synthesized dyes showed higher fluorescence quantum yields than indocyanine green (ICG).^[^ ^54^ ^]^ ICG is the only Food and Drug Administration (FDA) approved NIR fluorescent cyanine dye. It is commercially available, non‐toxic, relatively stable, displays amphiphilic properties, and its quantum yield in DMSO has been characterized.^[^ ^41^, ^54^ ^]^ Notably, the brightness of **4af** exhibited a 2.2‐fold improvement over ICG. The presence of cyclopentene rings instead of cyclohexene rings in **4f** and **4aa**, pyrrolidine **4e,** and quinoline salts **4c**, decreases the quantum yields.

### 2.2 Assessment of VDA Activity of MJH for Cell Membrane Permeabilization

**Figure 2:**
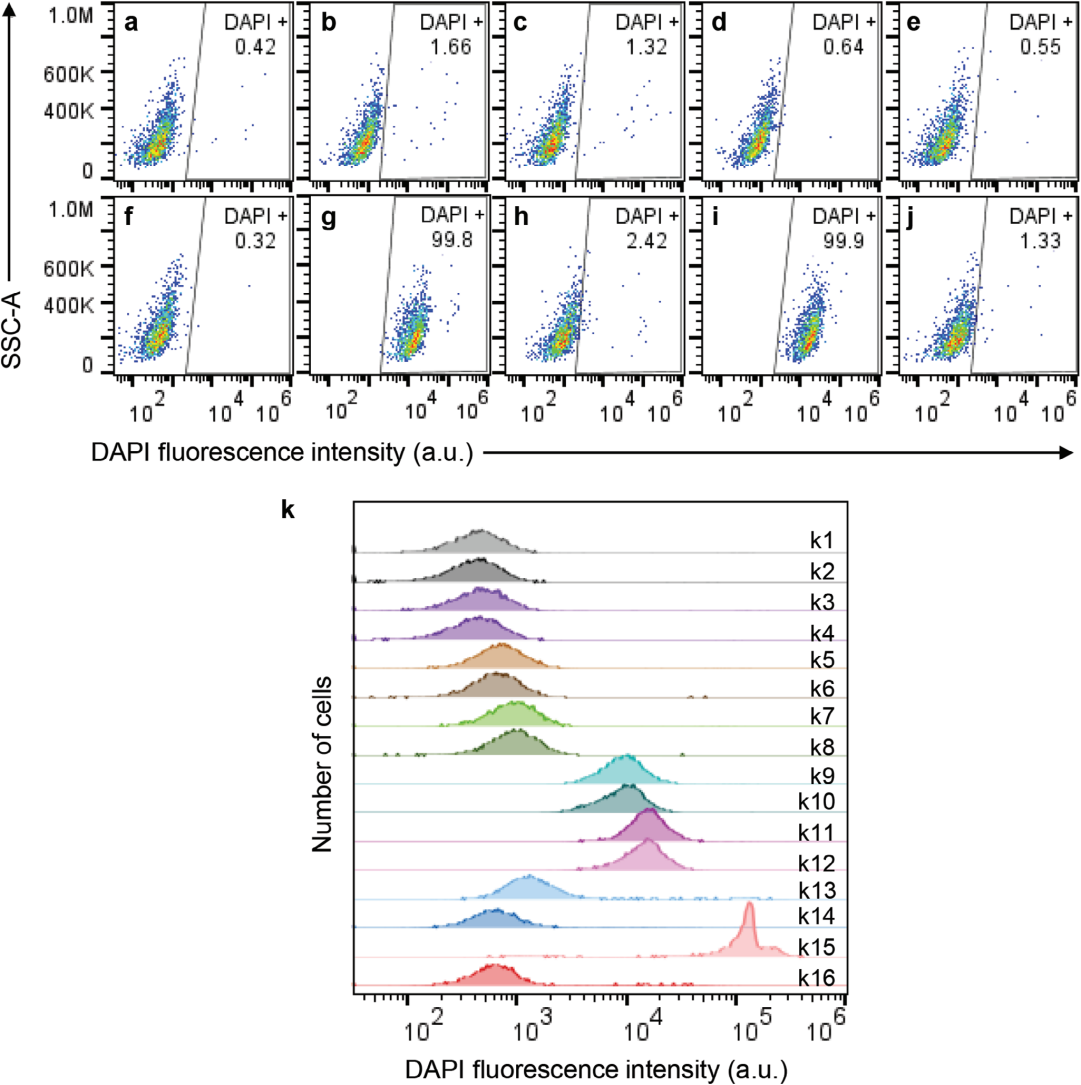
Assessment of MJH‐mediated cell membrane permeabilization through flow cytometry analysis in KPC cells. Molecules with benzoindole (**4q** and **4u**) versus indole (**4ac** and **4ae**) are compared. a,b) Flow cytometry analysis of cell suspension samples without light activation: a) 0.1% DMSO, b) 0.5 µm **4q**, c) 0.5 µm **4ac**, d) 0.5 µm **4u** and e) 0.5 µm **4ae**. f–j) Flow cytometry analysis of cell suspension samples treated with light activation: f) 0.1% DMSO + NIR light, g) 0.5 µm **4q** + NIR light, h) 0.5 µm **4ac** + NIR light, i) 0.5 µm **4u** + NIR light and j) 0.5 µm **4ae** + NIR light. k) Assessment of ROS contributions to membrane permeabilization through the addition of ROS scavengers (100 mm thiourea and 2.5 mm sodium azide) and/or hydrogen peroxide (H_2_O_2_, 50 mm). k1) 0.1% DMSO, k2) 0.1% DMSO + ROS scavengers, k3) 0.1% DMSO + NIR light, k4) 0.1% DMSO + ROS scavengers + NIR light, k5) 0.5 µm **4q**, k6) 0.5 µm **4q** + ROS scavengers, k7) 0.5 µm **4u**, k8) 0.5 µm **4u** + ROS scavengers, k9) 0.5 µm **4q** + NIR light, k10) 0.5 µm **4q** + ROS inhibitors + NIR light, k11) 0.5 µm **4u** + NIR light, k12) 0.5 µm **4u** + ROS scavengers + NIR light, k13) 50 mm H_2_O_2_ for 10 min, k14) 50 mm H_2_O_2_ for 10 min + ROS scavengers, k15) 50 mm H_2_O_2_ for 1 h, k16) 50 mm H_2_O_2_ for 1 h + ROS scavengers.

Flow cytometry analysis was conducted to evaluate the ability of MJH‐driven VDA activity to permeabilize the cell membrane of KPC mouse pancreatic cancer cells^[^ ^55^, ^56^, ^57^, ^58^ ^]^ under NIR light activation (730 nm, 80 mW cm^−2^ for 10 min) in vitro (**Figure** **2**; Figures S35–S37, Supporting Information). KPC cells, which have *LSL‐Kras^G12D/+^, LSL‐Trp53^R172H/+^*, and *Pdx‐1‐Cre* mutations, represent a well‐established and extensively studied preclinical genetic model of pancreatic ductal adenocarcinoma (PDAC), exhibiting numerous significant features observed in human PDAC.^[^ ^56^, ^58^ ^]^ The VDA activity of NIR‐activated MJH was compared against controls without light activation. In this assessment, MJH molecules with benzoindole groups (strong MJH: **4q** and **4u**) versus indole groups (weak MJH: **4ac** and **4ae**) were compared.^[^ ^29^ ^]^ Membrane damage was assessed using 4′,6‐diamidino‐2‐phenylindole (DAPI) as it does not absorb NIR light. When cell membranes are ruptured, the blue fluorescent DAPI enters the cell and immediately stains the nucleus.^[^ ^59^ ^]^ This is indicative of a rapid necrotic cell death and demonstrates that MJH‐induced VDA has permeabilized the cell membrane. The effective concentration needed to permeabilize 50% of the cells (VDA IC_50_) in the presence of NIR light activation (730 nm, 80 mW cm^−2^ for 10 min) was estimated for each molecule (Figure S37, Supporting Information).

We consistently observed that MJH containing benzoindole groups were the most effective at permeabilizing KPC cancer cells (Figures S35–S37, Supporting Information). This observation aligns with our understanding of plasmonicity in these molecules. Plasmonicity is associated with the number of electrons available in the molecule to participate in whole‐molecule vibration, and a higher plasmonicity index is associated with an increased ability to mechanically open cell membranes.^35^ We previously reported that benzoindole‐containing MJH has a higher plasmonicity index than indole‐containing MJH, as the benzoindole group provides a higher number of electrons in the system to support a stronger collective whole‐molecule vibration.^[^ ^35^ ^]^ This prior finding explains our current observation of enhanced permeabilization efficacy in benzoindole‐containing MJH since the higher plasmonicity translates to more potent mechanical disruption of cell membranes upon NIR light activation.

Next, we investigated the potential involvement of ROS in MJH‐mediated cell membrane permeabilization. We first validated the effectiveness of a ROS scavenger mixture (100 mm thiourea and 2.5 mm sodium azide) in preventing ROS‐mediated cell membrane permeabilization.^[^ ^36^ ^]^ Exposure of KPC cells to 50 mm H_2_O_2_ for 1 h effectively permeabilized the cells (Figure 2‐k15). However, pretreatment with the ROS scavenger mixture significantly inhibited H_2_O_2_‐induced membrane permeabilization (Figure 2‐k16), confirming the efficacy of our ROS scavenging approach. Crucially, when we applied the same ROS scavenger mixture to cells treated with light‐activated MJH, we observed no inhibition of VDA‐mediated membrane permeabilization (Figure 2‐k10,k12), indicating that ROS are not involved in the mechanism of MJH‐induced VDA. These results provide compelling evidence that VDA relies on mechanical disruption of cell membranes rather than oxidative damage and that MJH‐driven VDA operates through a mechanism distinct from PDT.^[^ ^29^, ^36^, ^60^ ^]^

In addition, we conducted measurements of ROS levels and media temperature to confirm that photodynamic and photothermal effects are negligible contributors to cell permeabilization in our experiments. We compared ROS production, assessed with the fluorescent probe 2',7'‐dichlorodihydrofluorescein diacetate (H2DCF‐DA), and cell permeabilization efficacy between benzoindole‐containing MJH (strong MJH: **4q** and 4u) and indole‐containing MJH (weak MJH: 4ac and 4ae) in KPC cells (Figure S38, Supporting Information). Interestingly, we found that indole‐containing molecules produced higher levels of ROS but were less effective at permeabilizing KPC cells. Conversely, benzoindole‐containing MJH, despite generating lower ROS levels, demonstrated superior cell permeabilization capabilities. This inverse relationship between ROS production and permeabilization efficacy strongly suggests that ROS is not the primary mechanism driving MJH‐induced cell permeabilization. These findings are consistent with previous observations in A375 melanoma cells which revealed that ROS levels generated by light‐activated MJH were insufficient to cause cell permeabilization.^[^ ^29^ ^]^ Collectively, these results establish that the VDA mechanism of MJH operates independently of ROS production.

Next, we investigated the potential photothermal effects of the potent MJH molecule **4q** at a concentration of 2 µM, which is 167 times higher than the IC_50_ (0.12 µm) needed to permeabilize KPC cells (Figure S39, Supporting Information). We observed only a minimal temperature increase of 0.5 °C in the surrounding liquid, which was not higher than the control with 0.1% DMSO and can be attributed to light absorption by media components. This demonstrates that photothermal effects are insignificant at concentrations far exceeding those required for effective cell permeabilization. These findings are consistent with our previous study, which showed that VDA does not cause a significant increase in the surrounding liquid temperature.^[^ ^29^ ^]^ and that photothermal effects become noticeable only above 8 µM for most MJH.^[^ ^29^, ^36^ ^]^ The current results confirm that at therapeutically relevant concentrations, photothermal effects do not contribute significantly to the MJH mechanism of action.

We measured the chemical and biological stability of Cy7.5 (strong MJH: **4q** and **4u**) and Cy7 (weak MJH: **4ac** and **4ae**) in cell culture media over time (Figure S40, Supporting Information). The stability ranking of the molecules from most to least stable was **4u** > **4ae** > **4ac** > **4q**. Interestingly, the most potent molecule for permeabilizing cancer cells (**4q**) was the least stable in cell culture media, indicating that the stability of MJH molecules does not correlate with higher VDA activity for permeabilizing cancer cells.

The mechanism by which Cy7 and Cy7.5 amines mechanically disrupt cell membranes under NIR light irradiation involves a complex interplay of photophysical and photomechanical processes. Recent advances in photoacoustic imaging provide valuable insights into this phenomenon. Bohndiek and collaborators demonstrated that NIR‐absorbing dyes can generate mechanical waves upon excitation, a principle that underlies the action of our molecular jackhammers (MJH).^[^ ^61^ ^]^ Upon absorption of NIR light, Cy7 and Cy7.5 molecules undergo rapid electronic excitation. This excitation is followed by nonradiative relaxation processes, which typically occur on picosecond to nanosecond timescales. In conventional photoacoustic agents, this relaxation leads to localized heating and subsequent thermoelastic expansion, generating acoustic waves. However, these MJH appear to operate through a distinct mechanism that does not rely on thermal effects. Instead, we propose that the energy from electronic excitation in our MJH is channeled primarily into specific vibrational modes through strong vibronic coupling. This coupling, as discussed earlier, is evidenced by the prominent vibronic shoulder in the absorption spectra of these dyes. The rapid population of these vibrational states leads to coherent, large‐amplitude molecular motions. When the MJH is associated with cell membranes, these motions couple to membrane lipids and proteins, exerting mechanical forces that disrupt membrane integrity. The efficiency of this process depends on several factors. First, the strength of vibronic coupling, can be greater in Cy7.5 than in Cy7 due to its extended π‐conjugation system. Second, the match between the frequency of the dominant molecular vibrations and the natural frequencies of membrane components. Third, the orientation and proximity of the MJHs relative to the membrane, would affect the efficiency of mechanical energy transfer.^[^ ^29^, ^35^ ^]^

### 2.3 Cellular Clearance of MJH from Normal and Cancer Cells

To address concerns about potential toxicity of unused MJH and their metabolism, we measured the clearance of the strong MJH 4u from normal cells (HEK293T) and cancer cells (KPC) over time (Figures S41 and S42, Supporting Information). We found that a non‐toxic dose of 4u (0.5 µM) was safe and did not cause damage to normal or cancer cells, even at prolonged incubation times. Importantly, our results indicate that MJH is cleared from normal cells, likely through exocytosis. These findings complement our previous studies on the cellular interactions of MJH. We have previously shown that MJH first binds to the cellular membrane and can then be internalized into the cytoplasm, likely by passive diffusion, ultimately accumulating in the mitochondria or endoplasmic reticulum (ER).^[^ ^35^, ^36^ ^]^ Notably, we observed that the fraction of MJH bound to the external surface of the cell membrane is sufficient to induce membrane permeabilization through VDA upon light activation.^[^ ^36^ ^]^ Accordingly, observations in synthetic lipid vesicles revealed that MJH bound to the outer leaflet of the membrane bilayer was able to mechanically disrupt the vesicle structure.^[^ ^36^ ^]^ In both cellular and synthetic systems, complete internalization of MJH was not necessary for their membrane‐disrupting effect.^[^ ^35^, ^36^ ^]^ This mechanism explains the rapid permeabilization of cell membranes we observed in our current study, even before significant internalization of MJH could occur. The relatively rapid clearance of MJH from cells observed in this study suggests a potential pathway for their elimination from the body. Once in the bloodstream, these molecules can be eliminated by the urinary system and/or metabolized in the liver.^[^ ^62^ ^]^ The resulting by‐products can then be released into the blood or bile and be expelled through the urine or feces. This clearance mechanism provides important insights into the potential safety profile of MJH for therapeutic applications.

### 2.4 Anticancer Activity of MJH

**Figure 3:**
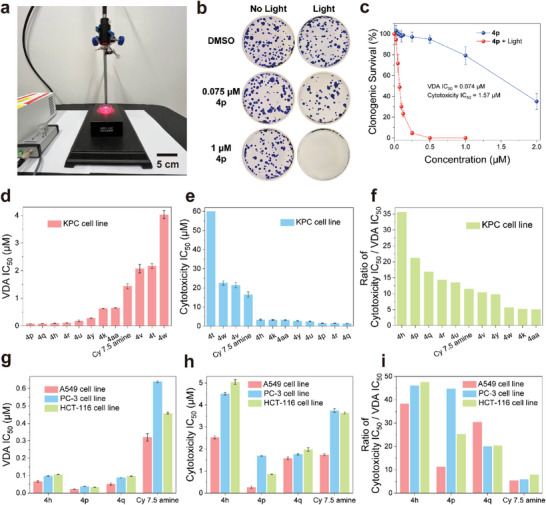
Assessment of cell survival and proliferative potential after MJH treatment under 730 nm light (80 mW cm^−2^, 10 min) and without light by clonogenic assay. a) Experimental setup used for cancer cell eradication. Scale bar, 5 cm. b) Representative images showing cell colony growth in controls (0.1% DMSO with or without light) and complete eradication of KPC cells when treated with 1 µm **4p** and light. (730 nm light, 80 mW cm^−2^, 10 min). Dish diameter, 35 mm. c) Quantification of colony‐forming cells to assess cell survival after treatment with **4p**. d) Concentration of MJH necessary to kill 50% of KPC cells with light activation (VDA IC_50_). e) Concentration of MJH necessary to kill 50% of KPC cells without light activation (cytotoxicity IC_50_). The cytotoxicity IC_50_ of **4t** was greater than 60 µm and no error bar is given. f) Ratio of cytotoxicity IC_50_ / VDA IC_50_ for each MJH on KPC cells. g) Concentration of **4h**, **4p**, **4q** and Cy7.5 amine necessary to kill 50% of A549 cells, PC‐3 cells and HCT‐116 cells with light activation (VDA IC_50_). h) Concentration of **4h**, **4p**, **4q** and Cy7.5 amine necessary to kill 50% of A549 cells, PC‐3 cells and HCT‐116 cells without light activation (cytotoxicity IC_50_). i) Ratio of cytotoxicity IC_50_ / VDA IC_50_ for each molecule on A549 cells, PC‐3 cells and HCT‐116 cells. Error bars represent standard deviation, and all results are normalized relative to the DMSO control. The experimental design included three independent sample repetitions (n = 3).

To evaluate the efficiency of MJH in eradicating cancer cell lines under NIR light activation (730 nm, 80 mW cm^−2^ for 10 min) in vitro, we employed the clonogenic assay^[^ ^63^ ^]^ on KPC cells^[^ ^55^, ^56^, ^57^, ^58^ ^]^ under both light and dark conditions following treatment with diverse MJH at various concentrations (**Figure** **3** **)**. The experimental setup is depicted in Figure 3a. Flow cytometry analysis demonstrated superior VDA activity in benzoindolium‐based MJH over indolium‐based ones for cell permeabilization. Therefore, we focused subsequent clonogenic assays exclusively on benzoindolium‐based MJH. We defined VDA IC_50_ as the effective concentration needed to kill 50% of the cell population under light activation, and cytotoxicity IC_50_ as the concentration needed to inhibit cell growth by 50% under dark conditions.

Analysis of twelve MJH revealed that nonsymmetric molecules exhibit higher activity under light conditions but also demonstrate higher dark toxicity compared to symmetric ones (Figure 3d,e). MJH **4p** with dimethylcarbamoyl substitution exhibited the highest VDA activity among the tested molecules (Figure 3b,c, VDA IC_50_ = 74 nm). MJH **4q** and **4h** with dimethylamino ethyl substitutions also demonstrated excellent VDA activity in cancer cell eradication. However, MJH **4h** exhibited significantly lower toxicity than **4q**, resulting in the highest phototherapeutic index (ratio of cytotoxicity IC_50_/VDA IC_50_, as shown in Figure 3f) among the tested compounds.

Furthermore, MJH **4r** (morpholino) showed comparable activity to **4h** but was more toxic. MJH with amino (Cy 7.5 amine and **4v**) and carboxylic acid (**4w**) substitutions indicated relatively lower VDA activities and phototherapy indices despite presenting lower toxicity. Interestingly, **4aa** with cyclopentene ring substitutions exhibited a notable decrease in VDA activity compared to **4u**, despite both having similar cytotoxicity IC_50_ values, providing the lowest phototherapeutic index. Notably, compound **4t**, featuring a sulfonate substitution similar to ICG,^[^ ^54^ ^]^ proved to be a comparatively safe MJH, with more than 90% of cells maintaining viability even when exposed to 60 µm despite its lower VDA activity (Figures S43–S48, Supporting Information).

We subsequently investigated the VDA efficiency of MJH **4h**, **4p**, **4q,** and Cy 7.5 amines on different human cancer cell lines (lung cancer cells A549, prostate cancer cells PC‐3, and colorectal cancer cells HCT‐116),^[^ ^26^, ^64^, ^65^ ^]^ as depicted in Figure 3g–i. The results were consistent across these cell lines and comparable to the observations in KPC cells. As expected, (Figures S49–S57, Supporting Information), **4p** exhibited significantly higher VDA activity (VDA IC_50_ less than 40 nm, shown in Figures S49, S52, and S55, Supporting Information) compared to other MJH among these three cell lines, while Cy 7.5 amine displayed the lowest activity (Figure 3g). Remarkably, **4h** showed the highest phototherapeutic index, with more than a sevenfold improvement compared to Cy 7.5 amine in both A549 (7.8‐fold) and PC‐3 (7.1‐fold) cell lines, along with a sixfold improvement in HCT‐116 cells (Figure 3i). Additionally, compound **4p** demonstrated a substantial improvement, up to 7.6‐fold, albeit only 2.1‐fold in A549 cells and 3.2‐fold in HCT‐116 cells. **4q** showed up to a 5.6‐fold improvement in the A549 cell line, a 3.6‐fold improvement in PC‐3 cells, and a 2.9‐fold improvement in HCT‐116 cells compared to Cy 7.5 amine. These results underscore the wide applicability of **4h** in different cancer cell lines and highlight the significance of structural optimization, encouraging further exploration of NIR‐activated MJH for cancer treatment in vivo.

## 3 Conclusion

In summary, we have developed a practical method to construct cyclohexene‐modified cyclic glutacondianil intermediates via a palladium‐catalyzed dehalogenation reaction. These intermediates served as a platform for the synthesis of various symmetrical and nonsymmetrical Cy7 and Cy 7.5 MJH with diverse functional substitutions on the heterocyclics in moderate to good yields. Additionally, we evaluated the photophysical properties of the substituted derivatives and demonstrated that side chains on heterocyclics showed minimal influence on the maximum absorption and emission wavelength. However, molar extinction coefficients and quantum yields were affected. Furthermore, we enhanced the efficiency of VDA to permeabilize cancer cell membranes, leading to cell death at low concentrations, and improved the phototherapeutic index using MJH with dimethylaminoethyl and dimethylcarbamoyl substitutions. Mechanistic studies revealed that the VDA's mechanical action to destroy cells was unaffected by high doses of ROS scavengers, highlighting a mode of action distinct from PDT. Near‐infrared (NIR) light alone at the intensities used in the study (80 mW cm^−2^) is generally considered safe for normal tissue. In our previous publications and this study, we have validated that 730 nm LED light at 80 mW/cm^2^ for 10 min does not cause cellular damage. This dose was also safe for in vivo studies, and we have shown it does not cause visible damage to normal tissue. The 730 nm light falls within the NIR optical therapeutic window where the tissue penetration is maximal and the absorption by water and hemoglobin is minimal. In this study, throughout our experiments, we consistently showed that this light dose is innocuous to cells as in Figures 2 and 3, and Figures S35–S38, S43–S57 (Supporting Information).

This work provides a general synthetic approach and contributes to the synthetic methodologies for cyanine structural optimization based on structure‐activity relationships, with encouraging results and prospects for cancer treatment. The high efficiency and broad applicability of these optimized MJH across multiple cancer cell lines suggest their potential as a versatile platform for cancer treatment. The ability to fine‐tune the phototherapeutic index through structural modifications offers a promising avenue for developing targeted therapies with minimal off‐target effects. Future work will focus on translating these findings to in vivo models and exploring the potential of these MJH for combination therapies. Additionally, the unique VDA mechanism of these compounds may open new possibilities for overcoming drug resistance in cancer treatment, a direction that warrants further investigation.

## Conflict of Interest

Rice University owns intellectual property on the use of MJH for the permeabilization of cell membranes. The authors declare no other potential conflicts.

## Supporting information



Supporting Information

## Data Availability

The data that support the findings of this study are available in the supplementary material of this article.
