# Supplementary material for: Divergent Syntheses of Near‐Infrared Light‐Activated Molecular Jackhammers for Cancer Cell Eradication
Source: Adv Sci (Weinh). 2024 Oct 13;11(45):2405965. doi: 10.1002/advs.202405965 (PMC11615805; doi:10.1002/advs.202405965)

ADVANCED SCIENCE
Open Access

# Supporting Information

for *Adv. Sci.*, DOI 10.1002/advs.202405965

Divergent Syntheses of Near-Infrared Light-Activated Molecular Jackhammers for Cancer Cell Eradication

*Bowen Li, Ciceron Ayala-Orozco*, Tengda Si, Lixin Zhou, Zicheng Wang, Angel A. Martí and James M. Tour**

**Supporting Information**

**Divergent Syntheses of Near-Infrared Light-Activated Molecular Jackhammers for Cancer Cell Eradication**

*Bowen Li, Ciceron Ayala-Orozco,* Tengda Si, Lixin Zhou, Zicheng Wang, Angel A. Martí, James M. Tour**

## 1. General information

**Materials and Methods.** All glassware was oven-dried overnight prior to use. All chemicals were purchased from commercial suppliers and used without further purification. All reactions were carried out under $N_2$ atmosphere unless otherwise noted. Flash column chromatography was performed using 230-400 mesh silica gel. Thin layer chromatography (TLC) was performed using glass plates pre-coated with silica gel $F_{254}$ 0.25 mm layer thickness purchased from EM science. $^1$H-NMR spectra were recorded at 600 MHz and $^{13}$C-NMR spectra were recorded at 151 MHz. Chemical shifts (δ) are reported in ppm relative to tetramethylsilane (TMS). HRMS (ESI) were conducted on Agilent Quadrupole-TOF LC/MS spectrometer.

**UV-vis and emission spectra measurements.** UV-vis spectra were acquired in a 1-cm quartz cuvette using a Shimadzu UV-2450 spectrophotometer. Steady-state photoluminescence emission spectra were obtained on a Horiba Jobin Yvon FluoroLog exciting with a 450 W Xe lamp in front face (FF) mode. Relative quantum yield measurements were determined using ICG in DMSO as a reference ($\Phi$ = 0.13) as a standard. Photoluminescence quantum yield of unknown (unk) sample can be calculated by this equation, $\Phi_{std}$ is the quantum yield of standard (std), A is absorbance

intensity, I is emission intensity, n is the refractive index of solvents used.

$$\Phi_{unk} = \Phi_{std} \times \frac{A_{std}}{I_{std}} \times \frac{I_{unk}}{A_{unk}} \times \frac{n_{unk}^2}{n_{std}^2}$$

**LED illumination systems.** The 730 nm LED (model UHP-F-730) was purchased from Prizmatix, Israel.

**Cell lines and culture conditions.** KPC pancreatic cancer cells were obtained from MD Anderson Cancer Center, A549 (Human lung cancer cell line, CCL-185™), PC-3 (Human prostate cancer cell line, CRL-1435 ™) and HCT-116 (Human colon cancer cell line, CCL-247 ™) cells were purchased from the American Type Culture Collection (ATCC). KPC cells was cultured with RPMI 1640 Medium (Gibco), A549 and PC-3 cells were cultured with Kaighn's Modification of Ham's F-12 Medium (F-12 K medium, ATCC, 30-2004 ™), HCT-116 cells was cultured with McCoy's 5A Medium (ATCC, 30-2007 ™), all the medium were supplemented with 10 % FBS (Fetal Bovine Serum, Corning, MT35010CV) and penicillin-streptomycin (Gibco, 15-140-122) in a humidified incubation system containing 5% $CO_2$ at 37$^o$C. For the passage step, cells are detached with 0.05 % trypsin-EDTA (Gibco, 25-300-054).

**Flow cytometry analysis.** The membrane permeabilization in cancer cells was measured by flow cytometry analysis following the procedure previously published.[1,2] Here the assessment was done in the pancreatic cancer cell line KPC. Briefly, KPC cells were cultured as described before. The cells were harvested using 0.05% trypsin-EDTA (Gibco, 25-300-054), then the cells were counted and were adjusted to a cell density of 2 x $10^5$ cells m/L in RPMI media (Gibco) and supplemented with 10% FBS (Corning, MT35010CV) and penicillin/streptomycin (Gibco, 15-

140-122). 1 mL of this cell suspension containing 2 x $10^5$ cells was used in each treatment. In a 1.5 mL Eppendorf tube, a mixture was prepared containing 1 mL of cell suspension and several cyanine dye (MJH) concentrations in the µM range. The mixture always contained 0.1% DMSO because DMSO was used as a solvent to prepare the stock cyanine solution in the mM range. The stock solution was diluted 1:1000 in the cell suspension mixture, which gives 0.1% DMSO. The mixture was then incubated at 37 °C and 5% $CO_2$ for 30 min. Then, 1 µM DAPI was added into the cell suspension and the cell suspension was transferred to a 35 mL polystyrene tissue culture dish. The cells were immediately treated under NIR light at 730 nm and 80 mW/cm over 10 min using LED light sources (Prizmatix, UHP-F-730, Israel). As soon as the 10 min light treatment was completed, the cell suspension was rapidly transferred from the 35 mm dish to a flow cytometry tube and the cells were analyzed for DAPI permeabilization by flow cytometry analysis (SONY, MA900 Multi-Application Cell Sorter). It took ~30 s to load the sample and to start cell counting. The permeabilization of cells was measured as DAPI positive cells and occurred immediately. For the ROS scavenger experiment, the same procedure was followed except that 100 mM thiourea and 2.5 mM sodium azide were added to the cell suspension containing 2 x $10^5$ cells/mL. The thiourea was prepared at a stock solution of 2.5 M in sterile DI water and sodium azide at 2.5 M in DI water. This mixture of ROS-scavengers was previously used and validated.[1,2,3]

**ROS measurements using $H_2$DCF-DA**. The method for measuring the ROS levels using $H_2$DCF-DA (2’,7’-dichlorodihydrofluorescein diacetate) in the presence of light-activated MJH was previously described.[1] Briefly, $H_2$DCF-DA is deacetylated by cellular esterases to form 2’,7’-dichlorodihydrofluorescein ($H_2$DCF), a non-fluorescent compound, which is rapidly oxidized in

the presence of ROS into 2',7'-dichlorofluorescein (DCF). DCF is highly fluorescent and is detected with excitation / emission at 488 nm / 535 nm. KPC cells in suspension containing 2x$10^5$ cells/mL were first prepared in RPMI media supplemented with 10% FBS and penicillin/streptomycin. Then the cells were incubated for 30 min at 37 °C with MJH at 0.5 μM concentration in the media. Then, $H_2$DCF-DA (Sigma-Aldrich) was added to cell suspension in media to the final concentration of 5 μM (the stock of $H_2$DCF-DA was at 5 mM in DMSO stored at -20 °C). Then transfer the cells to a 96-well plate, 100 μL to each well. The following treatment conditions were prepared: MJH + light, MJH only, DMSO + light, DMSO only, cells only, media only. Then, immediately after the cells were treated with NIR 730 nm light at 80 mW/$cm^2$ for 10 min. After the light treatment, the DCF fluorescence intensity was measured immediately after the light treatment using a 96-well plate reader at $\lambda_{ex}$ = 488/9 nm and $\lambda_{em}$ = 535/20 nm. The measurements were normalized with respect to the fluorescence intensity in the media only. The same method was followed for A459 cells but using F-12K medium supplemented with 10% FBS and penicillin/streptomycin.

**Temperature measurements**. The temperature of the media was measured as described before.[1] Briefly, the temperature of the media was measured using the temperature probe (Model SC-TT-K-30-36-PP; Omega Engineering, Inc.) immersed in the liquid during the NIR light illumination. The initial temperature of the media was ~20 °C (room temperature). KPC cells were treated with molecule **4q** at 2 μM or 0.1% DMSO and with 730 nm LED light illumination at 80 mW/$cm^2$ for 10 min. DMSO control contains 0.1% DMSO in the media because DMSO is used to pre-solubilize the **4q** stock solution at 2 mM and diluted to 1:1000 to obtain 2 μM **4q** in media

containing 0.1% DMSO. The temperature of the media stays nearly constant at room temperature of ~20 °C upon illumination of the media with the 730 nm LED light at 80 mW/cm$^2$ for 10 min. There is only a minor temperature increase of 0.6 °C that is attributed to the light absorption by the media components.

**Stability of Cy7 and Cy7.5 molecules in biological media over time.** To assess the chemical stability in biological media, the UV-vis absorption spectrum of Cy7 molecules (**4ac** and **4ae**) and Cy7.5 molecules (**4q** and **4u**) was measured over time in RPMI media supplemented with 10% Fetal Bovine Serum. The molecules were added to the media (concentration = 2 μM) in a polystyrene cuvette and incubated at 37 °C. The polystyrene cuvettes were covered with plastic caps to avoid liquid evaporation. The spectra were recorded immediately after addition, then at 2 h, 4 h, and 20 h.

**Cellular uptake and clearance of MJH in cells over time.** Fluorescence confocal microscopy was conducted to evaluate the cellular uptake and clearance of MJH from cells. For this purpose, 30,000 KPC cells were seeded in a glass bottom dish (IBIDI, μ-dish 35 mm high glass bottom) with RPMI media supplemented with 10% FBS and penicillin/streptomycin and were culture for 2 d at 37 °C and 5% $CO_2$. Then, molecule **4u** was added to the KPC cells and continued the incubation conditions. The cells were removed from incubation for a period of ~10 min to record the confocal microscopy images at each time point. Imaging conditions: molecule **4u**, loading concentration $C_{\text{loading}}$ = 0.5 μM, $\lambda_{ex}$ = 640 nm, $\lambda_{em}$ = 663-738 nm. The cells were washed 3 times with fresh media after recording the data point at 1.5 h and washed 1 time after recording the data point at 6 h.

**Clonogenic assay.** Cancer cells were seeded in 35 mm cell culture dishes (Falcon, 353001) at predetermined densities to allow for an approximately equal number of resultant colonies. After incubated 24 h, the cells were treated with different MJH at various concentrations and incubated for 40 min before the illumination with 730 nm light at 80 $mW/cm^2$ for 10 min (the same number of samples were not illuminated, acting as controls). The media was replaced with fresh media after the illumination and cells were cultured for 7-12 d to allow for colony formation. Cells were then washed once with PBS and fixed stained in a 0.5% (w/v) crystal violet in methanol/water solution (1:1) during 15 min. The excess crystal violet was washed off with water, the plates were dried at room temperature and then the colonies were counted, and the survival fraction was determined.

## 2. General procedures for synthesis of cyanine dyes and data characterization

**Synthesis of key intermediate compound 2:**

General procedure: To a screw-capped vial was added **1** (1 equiv), $Pd(PPh_3)_4$ (10 mol%), Base (4 equiv). The vial was sealed with a PTFE septum and then evacuated and backfilled with $N_2$ for three times, followed by addition of solvent via syringe and vigorous stirring. The sealed reaction was heated to 100 °C in oil bath heating for 48 h, then the reaction was cooled to room temperature and concentrated under reduced pressure, followed by 6M HCl at 0 °C and stirred for 10 minutes at room temperature. The precipitate was filtered, washed with $H_2O$, $Et_2O$ and acetone, dried in vacuo to provide compound **2** as a dark red solid.

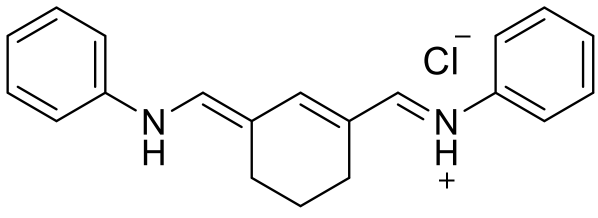


**2a**

**Synthesis of 2a:** Prepared according to the general procedure from **1a** (3 g, 8.38 mmol, 1 equiv). Yield: 68% (1.86 g). $^{1}$H-NMR (600 MHz, $CD_3OD$) δ 8.14 (s, 2H), 7.79 (s, 1H), 7.49 – 7.39 (m, 8H), 7.28 – 7.23 (m, 2H), 2.58 (t, *J* = 6.2 Hz, 4H), 1.99 – 1.93 (m, 2H). $^{13}$C NMR (151 MHz, $CD_3OD$) δ 163.58, 153.18, 140.74, 130.98, 127.16, 119.97, 119.18, 22.96, 21.58. HRMS (ESI) for calculated for [M+H, $C_{20}H_{21}N_2$]$^{+}$: 289.1699, found: 289.1707.

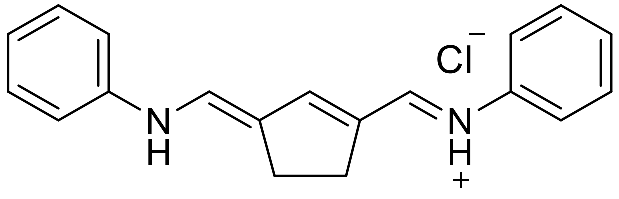


**2b**

**Synthesis of 2b:** Prepared according to the general procedure from **1b** (1 g, 2.91 mmol, 1 equiv). Yield: 75% (675 mg). $^{1}$H-NMR (600 MHz, $CD_3OD$) δ 8.39 – 8.34 (m, 2H), 7.96 (s, 1H), 7.43 – 7.38 (m, 8H), 7.23 – 7.19 (m, 2H), 2.99 – 2.95 (m, 4H). $^{13}$C-NMR (151 MHz, $CD_3OD$) δ 167.75, 146.77, 146.74, 140.73, 130.98, 129.72, 126.96, 118.85, 28.18. HRMS (ESI) calculated for [M+H, $C_{19}H_{19}N_2$]$^{+}$: 275.1543, found: 275.1565.

**Scheme S1**: Summary of heterocyclic salts **3** used in synthesis of cyanine dyes.[4,5]

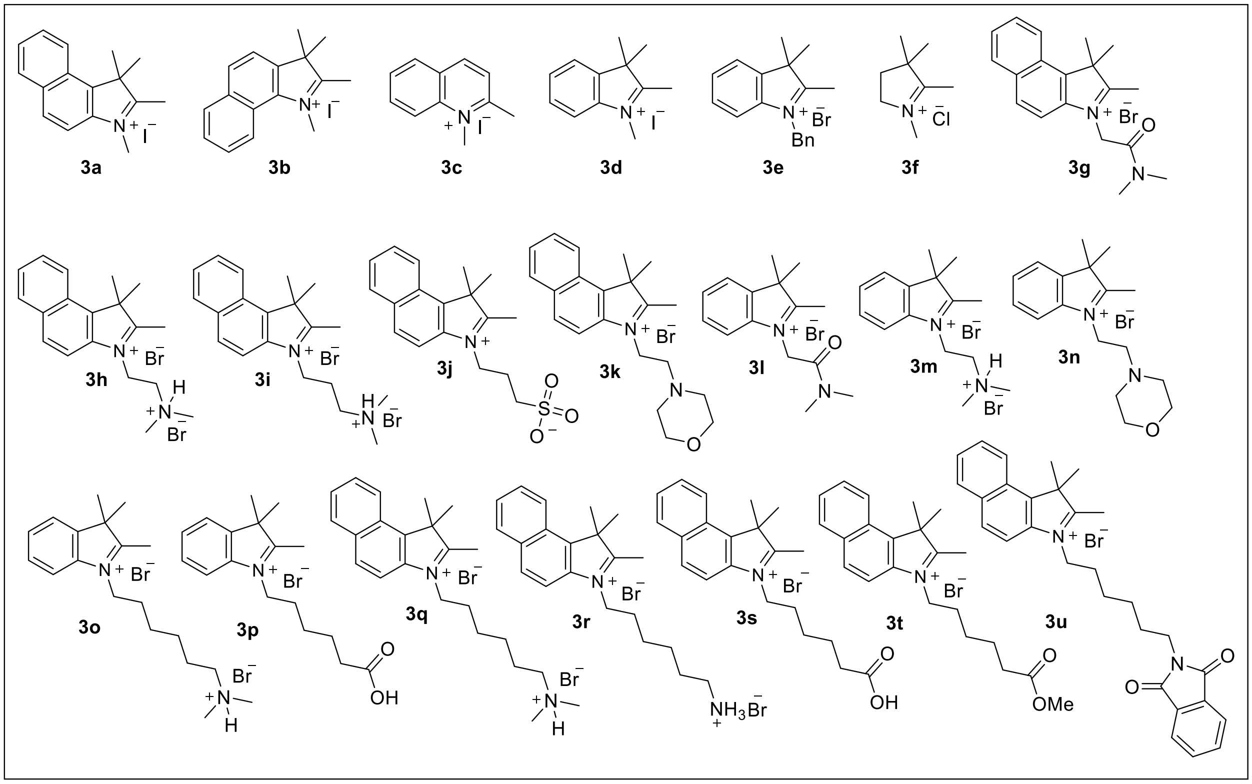

**Scheme S2**: Synthesis of cyanine dye **4** between heterocyclic salts **3/3'** and key intermediate **2**.

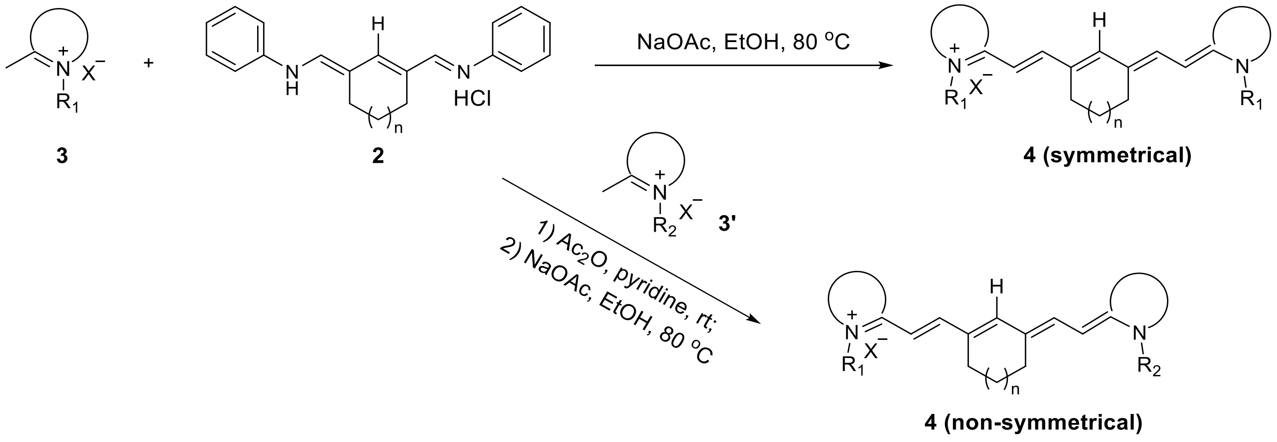

**For symmetric cyanine dyes (4a-4o):**

General procedure **a**: To a screw-capped vial charged with *N*-heterocyclic salt **3** (2 equiv), **2** (1 equiv) and NaOAc (3 equiv) dissolved in anhydrous ethanol. The mixture was heated at 80 °C under $N_2$ atmosphere until the reaction was completed. The solvent was removed under reduced

pressure and the crude product was purified by silica gel chromatography with DCM/MeOH as eluent to afford dye **4**.

**For non-symmetric cyanine dyes (4p-4af):**

General procedure **b**: *N*-heterocyclic salt **3** (1 equiv) and **2** (1 equiv) were added to a solution of $Ac_2O$ and pyridine. The reaction mixture was stirred at room temperature for 2 h, then a large volume of diethyl ether was added, and the reaction mixture was filtered off to afford dark red solid. Subsequently, to a screw-capped vial charged with the residue, another different salt **3'** (1 equiv), anhydrous sodium acetate (3 equiv) in anhydrous ethanol and stirred at 80 °C under $N_2$ atmosphere. After the reaction was complete, the product was purified by silica gel column chromatography with DCM/MeOH as eluent to afford dye **4**.

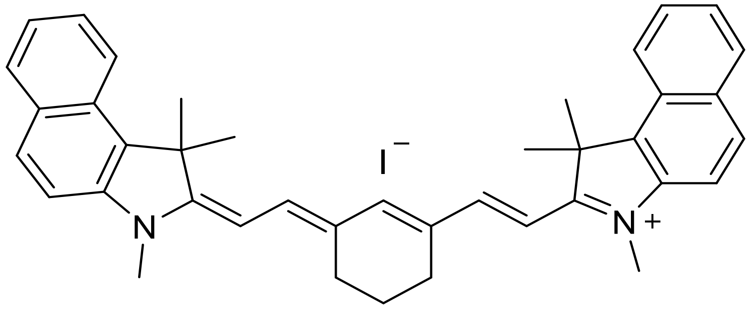


**4a**

**Synthesis of 4a:**[2] Prepared according to the general procedure **a** from **3a** (70 mg, 0.2 mmol, 2 equiv) and **2a** (33 mg, 0.1 mmol, 1 equiv). Dark-green solid. Yield: 84% (57 mg). $^1$H-NMR (600 MHz, $CD_3OD$) δ 8.24 – 8.21 (m, 2H), 8.00 (d, *J* = 8.8 Hz, 2H), 7.97 (d, *J* = 8.6 Hz, 2H), 7.87 (d, *J* = 14.2 Hz, 2H), 7.64 – 7.60 (m, 2H), 7.57 (d, *J* = 8.8 Hz, 2H), 7.53 (s, 1H), 7.48 – 7.44 (m, 2H), 6.19 (d, *J* = 14.1 Hz, 2H), 3.72 (s, 6H), 2.61 (t, *J* = 6.1 Hz, 4H), 2.03 – 1.95 (m, 14H). $^{13}$C-NMR (151 MHz, $CD_3OD$) δ 174.72, 148.86, 141.83, 134.45, 133.79, 133.77, 133.69, 133.30, 133.10,

133.04, 133.00, 132.30, 131.59, 131.09, 130.02, 129.94, 129.46, 128.65, 125.80, 123.31, 111.80, 100.44, 51.99, 31.82, 27.51, 25.02, 22.77. HRMS (ESI) calculated for [M, $C_{40}H_{41}N_2$]$^+$: 549.3264, found: 549.3275.

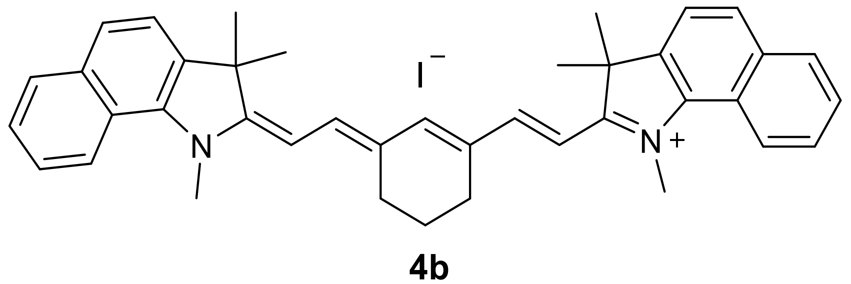

**4b**

**Synthesis of 4b:** Prepared according to the general procedure **a** from **3b** (70 mg, 0.2 mmol, 2 equiv) and **2a** (33 mg, 0.1 mmol, 1 equiv). Dark-green solid. Yield: 79% (53 mg). $^1$H-NMR (600 MHz, $CD_2Cl_2$) δ 8.53 – 8.45 (m, 2H), 7.96 (d, *J* = 7.5 Hz, 1H), 7.78 (d, *J* = 8.2 Hz, 2H), 7.72 – 7.58 (m, 4H), 7.57 – 7.50 (m, 4H), 7.49 – 7.45 (m, 1H), 6.29 – 6.12 (m, 2H), 4.18 (s, 6H), 2.61 (t, *J* = 6.4 Hz, 4H), 2.02 – 1.96 (m, 2H), 1.76 (s, 12H). $^{13}$C NMR (151 MHz, $CD_2Cl_2$) δ 173.38, 155.65, 148.16, 138.38, 137.69, 134.87, 133.31, 133.02, 132.62, 131.94, 131.88, 131.85, 131.84, 129.69, 129.66, 128.52, 128.44, 126.94, 126.28, 126.23, 125.96, 121.45, 121.42, 121.39, 121.36, 119.29, 100.47, 48.86, 37.32, 27.64, 24.13, 21.48. HRMS (ESI) calculated for [M, $C_{40}H_{41}N_2$]$^+$: 549.3264, found: 549.3247.

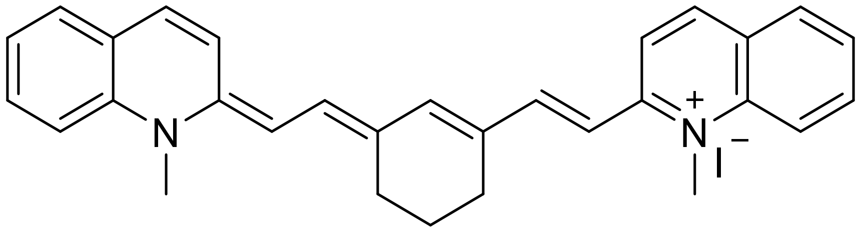

**4c**

**Synthesis of 4c:**[2] Prepared according to the general procedure **a** from **3c** (57 mg, 0.2 mmol, 2 equiv) and **2a** (33 mg, 0.1 mmol, 1 equiv). Ochre solid. Yield: 70% (38 mg). $^1$H-NMR (600 MHz, DMSO-d6) δ 7.89 (s, 4H), 7.79 – 7.73 (m, 4H), 7.66 – 7.62 (m, 2H), 7.54 (d, *J* = 13.6 Hz, 2H), 7.38 – 7.34 (m, 2H), 7.10 (s, 1H), 6.12 (d, *J* = 13.6 Hz, 2H), 3.83 (s, 6H), 2.52 – 7.50 (m, 4H), 1.85 – 1.79 (m, 2H). $^{13}$C-NMR (151 MHz, DMSO-d6) δ 150.63, 149.52, 143.90, 140.09, 135.16, 132.19, 130.46, 128.73, 124.64, 124.54, 120.05, 116.15, 104.06, 36.21, 24.24, 21.34. HRMS (ESI) calculated for [M, $C_{30}H_{29}N_2$]$^+$: 417.2325, found: 417.2329.

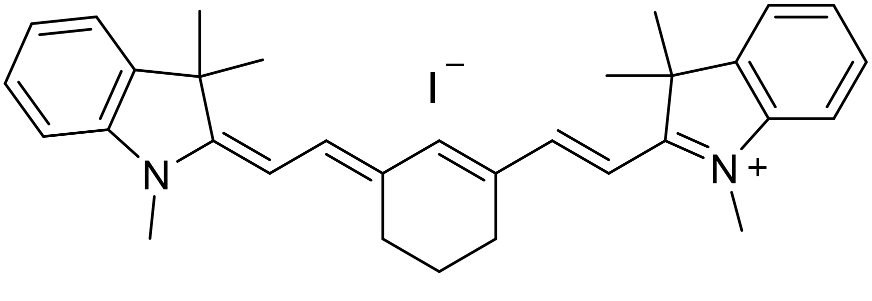

**4d**

**Synthesis of 4d:** Prepared according to the general procedure **a** from **3d** (60 mg, 0.2 mmol, 2 equiv) and **2a** (33 mg, 0.1 mmol, 1 equiv). Dark-green solid. Yield: 83% (48 mg). $^1$H-NMR (600 MHz, $CD_3OD$) δ 7.76 (d, *J* = 14.0 Hz, 2H), 7.49 – 7.44 (m, 3H), 7.39 (td, *J* = 7.7, 1.2 Hz, 2H), 7.27 – 7.21 (m, 4H), 6.15 (d, *J* = 14.0 Hz, 2H), 3.60 (s, 6H), 2.57 (t, *J* = 6.2 Hz, 4H), 1.98 – 1.92

(m, 2H), 1.71 (s, 12H). $^{13}$C-NMR (151 MHz, $CD_3OD$) δ 173.41, 149.81, 144.46, 142.27, 133.79, 129.68, 125.85, 123.24, 111.46, 100.77, 50.15, 31.40, 27.91, 24.96, 22.73. HRMS (ESI) calculated for [M, $C_{32}H_{37}N_2$]$^+$: 449.2951, found: 449.2946.

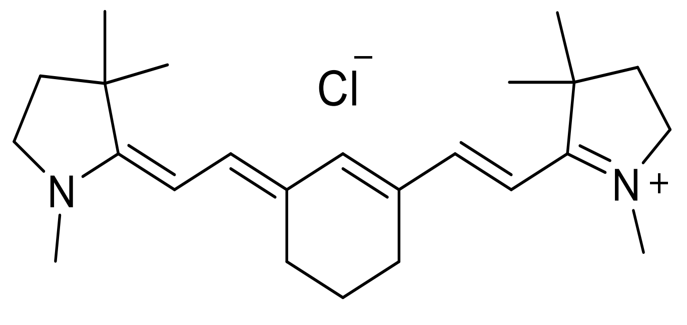

**4e**

**Synthesis of 4e:** Prepared according to the general procedure **a** from **3f** (50 mg, 0.3 mmol, 2 equiv) and **2a** (50 mg, 0.15 mmol, 1 equiv). Blue solid. Yield: 50% (29 mg). $^1$H-NMR (600 MHz, $CD_3OD$) δ 7.49 (d, $J$ = 14.2 Hz, 2H), 7.08 (s, 1H), 5.52 (d, $J$ = 14.1 Hz, 2H), 3.70 (t, $J$ = 7.2 Hz, 4H), 3.15 (s, 6H), 2.40 (t, $J$ = 6.2 Hz, 4H), 2.00 – 1.94 (m, 4H), 1.88 – 1.82 (m, 2H), 1.47 (s, 12H). $^{13}$C-NMR (151 MHz, $CD_3OD$) δ 174.96, 155.01, 149.29, 129.21, 96.97, 55.24, 48.03, 39.05, 35.82, 27.61, 24.91, 22.79. HRMS (ESI) calculated for [M, $C_{24}H_{37}N_2$]$^+$: 353.2951, found: 353.2957.

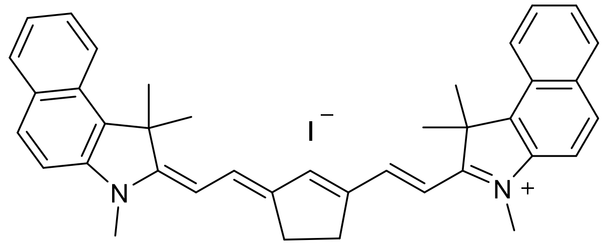

**4f**

**Synthesis of 4f:** Prepared according to the general procedure **a** from **4a** (70 mg, 0.2 mmol, 2 equiv)

and **2b** (31 mg, 0.1 mmol, 1 equiv). Dark-green solid. Yield: 78% (52 mg). $^{1}$H-NMR (600 MHz, $CD_2Cl_2$) δ 8.15 – 8.12 (m, 2H), 7.97 – 7.93 (m, 4H), 7.87 (d, *J* = 13.9 Hz, 2H), 7.63 – 7.60 (m, 2H), 7.52 (s, 1H), 7.48 – 7.43 (m, 4H), 5.92 (d, *J* = 13.9 Hz, 2H), 3.71 (s, 6H), 2.98 (s, 4H), 1.98 (s, 12H). $^{13}$C-NMR (151 MHz, $CD_2Cl_2$) δ 172.46, 156.80, 144.93, 140.72, 139.92, 133.62, 132.28, 132.21, 132.19, 132.18, 132.09, 130.80, 130.33, 128.86, 128.86, 128.78, 128.78, 128.53, 127.98, 125.15, 122.55, 110.85, 102.16, 54.20, 54.02, 53.84, 53.66, 53.48, 51.10, 32.35, 28.46, 27.46. HRMS (ESI) calculated for [M, $C_{39}H_{39}N_2$]$^+$: 535.3108, found: 535.3149.

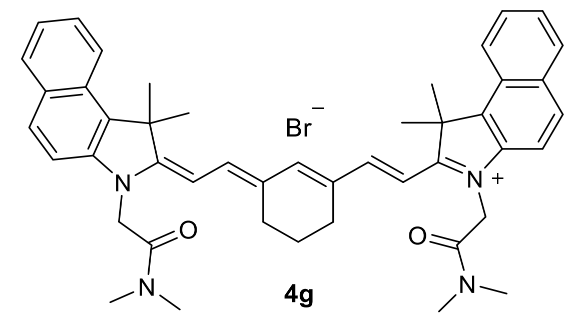


**Synthesis of 4g:** Prepared according to the general procedure **a** from **3g** (75 mg, 0.2 mmol, 2 equiv) and **2a** (33 mg, 0.1 mmol, 1 equiv). Dark-green solid. Yield: 57% yield (44 mg). $^{1}$H-NMR (600 MHz, $CD_3OD$) δ 8.23 (d, *J* = 8.5 Hz, 2H), 7.97 – 7.93 (m, 4H), 7.86 (d, *J* = 14.0 Hz, 2H), 7.64 – 7.60 (m, 2H), 7.50 (s, 1H), 7.47 – 7.42 (m, 4H), 6.06 (d, *J* = 13.9 Hz, 2H), 5.23 (s, 4H), 3.31 (s, 6H), 3.04 (s, 6H), 2.54 (t, *J* = 6.2 Hz, 4H), 2.05 (s, 12H), 1.94 – 1.90 (m, 2H). $^{13}$C-NMR (151 MHz, $CD_3OD$) δ 175.68, 167.08, 156.60, 149.40, 141.74, 134.16, 133.33, 131.46, 131.08, 129.45, 128.61, 125.79, 123.32, 111.75, 100.76, 52.25, 46.48, 37.01, 36.28, 27.62, 24.93, 22.73. HRMS (ESI) calculated for [M, $C_{46}H_{51}N_4O_2$]$^+$: 691.4007, found:691.4068.

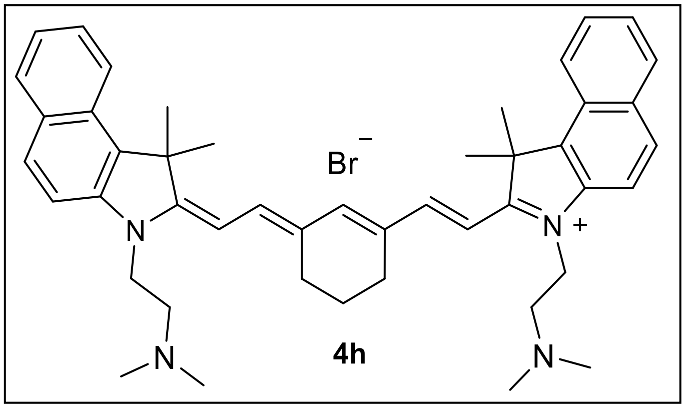

**Synthesis of 4h:** Prepared according to the general procedure **a** from **3h** (88 mg, 0.2 mmol, 2 equiv) and **2a** (33 mg, 0.1 mmol, 1 equiv). Dark-green solid. Yield: 54% yield (40 mg). $^{1}$H-NMR (600 MHz, $CD_3OD$) δ 8.21 (d, *J* = 8.6 Hz, 2H), 8.01 – 7.88 (m, 6H), 7.73 (s, 1H), 7.63 – 7.58 (m, 2H), 7.56 (d, *J* = 8.8 Hz, 2H), 7.45 (t, *J* = 7.5 Hz, 2H), 6.25 (d, *J* = 14.0 Hz, 2H), 4.33 (t, *J* = 7.3 Hz, 4H), 2.78 (t, *J* = 7.3 Hz, 4H), 2.61 (t, *J* = 6.1 Hz, 4H), 2.42 (s, 12H), 2.07 – 1.91 (m, 14H); $^{13}$C-NMR (151 MHz, $CD_3OD$) δ 174.32, 156.47, 148.90, 141.01, 134.55, 134.00, 133.29, 131.67, 131.09, 129.47, 128.68, 125.88, 123.34, 111.82, 100.66, 56.70, 52.11, 46.00, 43.29, 27.67, 25.05, 22.79. HRMS (ESI) calculated for $[M, C_{46}H_{54}N_4]^+$: 663.4421, found: 663.4465.

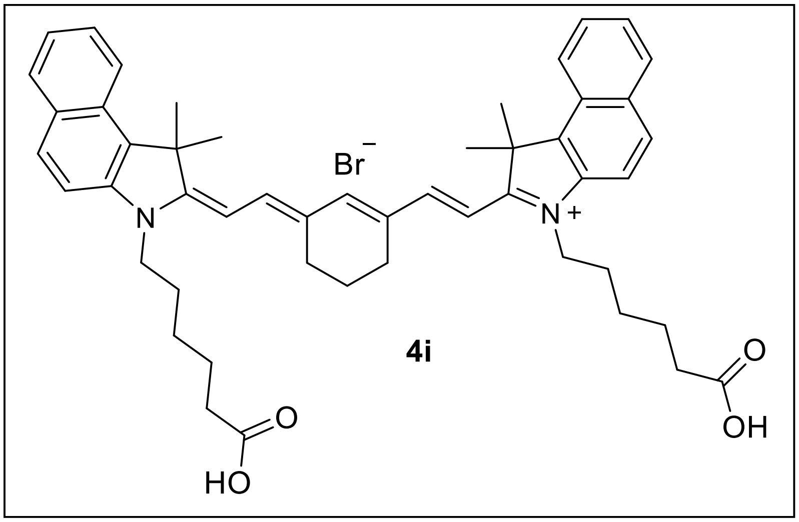

**Synthesis of 4i:** Prepared according to the general procedure **a** from **2a** (33 mg, 0.1 mmol, 1 equiv)

and **3s** (81 mg, 0.2 mmol, 2 equiv). Dark-green solid. Yield: 55% (46 mg). $^1$H-NMR (600 MHz, $CD_3OD$) δ 8.22 (d, *J* = 8.5 Hz, 2H), 8.00 – 7.95 (m, 4H), 7.85 (d, *J* = 14.0 Hz, 2H), 7.64 – 7.60 (m, 2H), 7.57 (d, *J* = 8.8 Hz, 2H), 7.49 – 7.43 (m, 3H), 6.22 (d, *J* = 14.0 Hz, 2H), 4.22 (t, *J* = 7.5 Hz, 4H), 2.60 (t, *J* = 6.2 Hz, 4H), 2.22 (t, *J* = 7.3 Hz, 4H), 2.07 – 1.93 (m, 14H), 1.93 – 1.87 (m, 4H), 1.74 – 1.69 (m, 4H), 1.56 – 1.50 (m, 4H). $^{13}$C-NMR (151 MHz, $CD_3OD$) δ 181.39, 174.02, 156.03, 148.80, 141.25, 134.60, 133.73, 133.29, 131.67, 131.10, 129.51, 128.63, 125.83, 123.32, 112.04, 100.39, 52.06, 44.97, 38.01, 28.35, 27.81, 27.57, 26.98, 25.00, 22.78. HRMS (ESI) calculated for [M, $C_{50}H_{57}N_2O_4$]$^+$: 749.4313, found: 749.4312.

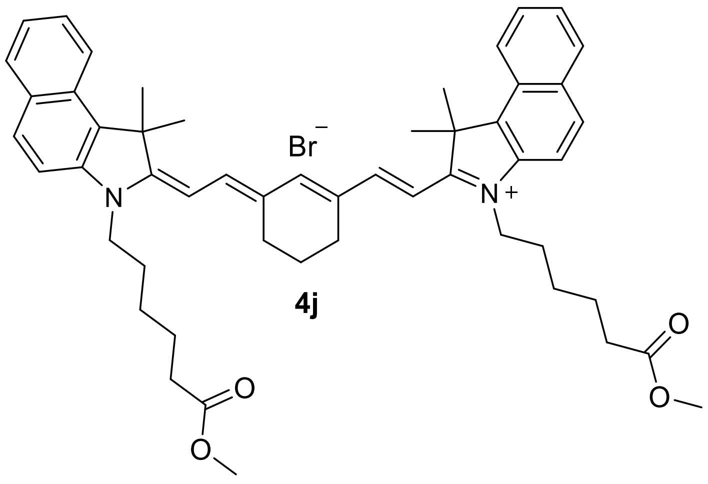

**4j**

**Synthesis of 4j:**[2] Prepared according to the general procedure **a** from **2a** (65 mg, 0.2 mmol, 1 equiv) and **3t** (167 mg, 0.4 mmol, 2 equiv). Dark-green solid. Yield: 67% (115 mg). $^1$H-NMR (600 MHz, $CD_3OD$) δ 8.19 (d, *J* = 8.8 Hz, 2H), 7.98 – 7.93 (m, 4H), 7.87 (d, *J* = 14.0 Hz, 2H), 7.63 – 7.56 (m, 3H), 7.53 (d, *J* = 8.8 Hz, 2H), 7.46 – 7.41 (m, 2H), 6.18 (d, *J* = 14.0 Hz, 2H), 4.19 (t, *J* = 7.5 Hz, 4H), 3.60 (s, 6H), 2.65 – 2.49 (m, 4H), 2.34 (t, *J* = 7.3 Hz, 4H), 2.00 – 1.91 (m, 14H), 1.89 – 1.83 (m, 4H), 1.73 – 1.67 (m, 4H), 1.53 – 1.47 (m, 4H). $^{13}$C-NMR (151 MHz, $CD_3OD$) δ 175.53,

174.06, 156.30, 148.85, 141.13, 141.03, 134.60, 133.66, 133.25, 131.66, 131.09, 129.47, 128.68, 125.87, 123.31, 111.97, 111.75, 100.36, 52.06, 52.01, 44.80, 34.46, 28.17, 27.62, 27.28, 25.63, 25.00, 22.78. HRMS (ESI) calculated for [M, $C_{52}H_{61}N_2O_4$]$^+$: 777.4626, found: 777.4626.

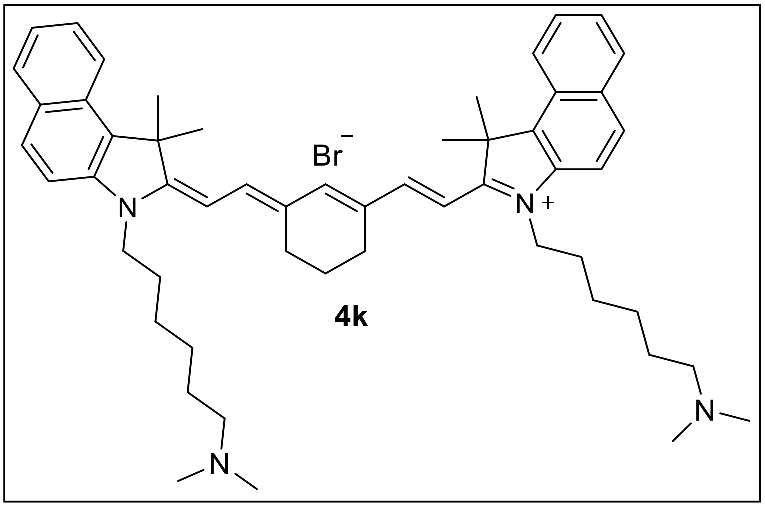


**Synthesis of 4k:**[2] Prepared according to the general procedure **a** from **2a** (81 mg, 0.25 mmol, 1 equiv) and **3q** (248 mg, 0.5 mmol, 2 equiv). Dark-green solid. Yield: 54% (115 mg). $^1$H-NMR (600 MHz, $CD_3OD$) δ 8.24 – 8.17 (m, 2H), 8.02 – 7.86 (m, 6H), 7.76 – 7.69 (m, H), 7.64 – 7.55 (m, 4H), 7.47 – 7.40 (m, 2H), 6.20 (brs, 2H), 4.33 – 4.16 (m, 4H), 3.20 – 3.15 (m, 4H), 2.89 (s, 12H), 2.61 (brs, 4H), 2.05 – 1.85 (m, 18H), 1.80 – 1.74 (m, 4H), 1.76 – 1.72 (m, 4H), 1.62 – 1.57 (m, 4H), 1.53 – 1.47 (m, 4H). $^{13}$C-NMR (151 MHz, $CD_3OD$) δ 174.02, 156.50, 148.88, 141.18, 134.62, 133.75, 133.23, 131.66, 131.07, 129.46, 128.66, 125.84, 123.35, 112.19, 100.42, 58.93, 52.09, 45.02, 43.67, 28.39, 27.74, 27.47, 27.26, 25.55, 25.23, 22.82. HRMS (ESI) calculated for [M, $C_{54}H_{71}N_4$]$^+$: 775.5673, found: 775.5662.

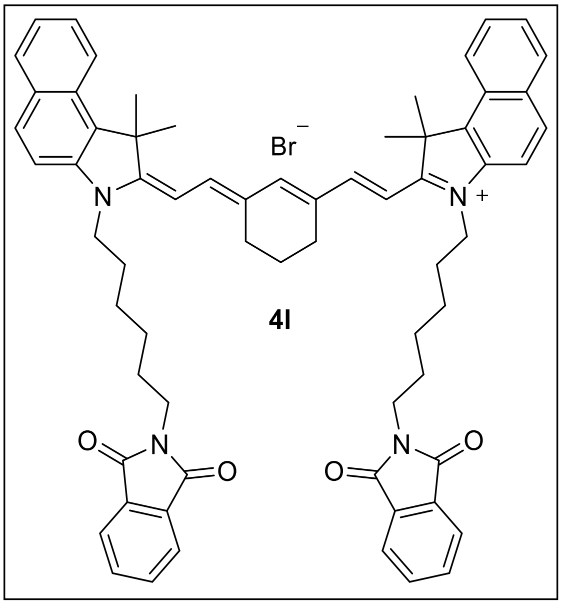

**Synthesis of 4l:** Prepared according to the general procedure **a** from **2a** (32 mg, 0.1 mmol, 1 equiv) and **3u** (103 mg, 0.2 mmol, 2 equiv). Dark-green solid. Yield: 75% (79 mg). $^{1}$H-NMR (600 MHz, $CD_2Cl_2$) δ 8.18 – 8.13 (m, 2H), 7.96 – 7.92 (m, 4H), 7.83 – 7.75 (m, 6H), 7.73 – 7.70 (m, 4H), 7.63 – 7.59 (m, 2H), 7.54 (s, 1H), 7.48 – 7.44 (m, 2H), 7.40 (d, *J* = 8.8 Hz, 2H), 6.08 (d, *J* = 14.7 Hz, 2H), 4.13 (t, *J* = 7.6 Hz, 4H), 3.67 (t, *J* = 7.1 Hz, 4H), 2.57 (t, *J* = 6.2 Hz, 4H), 2.01 (s, 12H), 1.98 – 1.95 (m, 2H), 1.91 – 1.85 (m, 4H), 1.73 – 1.67 (m, 4H), 1.57 – 1.51 (m, 4H), 1.47 – 1.42 (m, 4H). $^{13}$C-NMR (151 MHz, $CD_2Cl_2$) δ 172.84, 168.60, 155.72, 147.85, 140.04, 134.27, 133.73, 132.66, 132.41, 132.03, 130.76, 130.25, 128.49, 127.93, 125.12, 123.25, 123.20, 122.55, 110.89, 99.40, 51.17, 44.65, 37.89, 28.64, 27.65, 27.56, 26.84, 26.82, 24.31, 21.80. HRMS (ESI) calculated for [M, $C_{66}H_{67}N_4O_4$]$^+$: 979.5157, found: 979.5183.

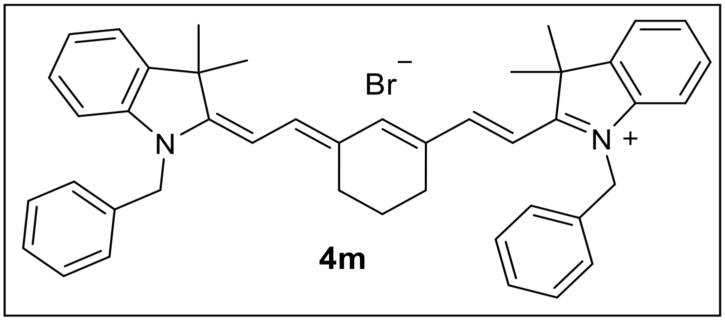

**Synthesis of 4m:** Prepared according to the general procedure **a** from **2a** (32 mg, 0.1 mmol, 1 equiv) and **3e** (66 mg, 0.2 mmol, 2 equiv). Dark-green solid. Yield: 79% (54 mg). $^{1}$H-NMR (600 MHz, $CD_2Cl_2$) δ 7.70 – 7.60 (m, 2H), 7.46 – 7.39 (m, 3H), 7.39 – 7.35 (m, 4H), 7.34 – 7.30 (m, 4H), 7.28 – 7.25 (m, 4H), 7.25 – 7.22 (m, 2H), 7.10 (d, *J* = 7.9 Hz, 2H), 6.08 (d, *J* = 13.9 Hz, 2H), 5.27 (s, 4H), 2.35 (t, *J* = 6.2 Hz, 4H), 1.82 – 1.73 (m, 14H); $^{13}$C-NMR (151 MHz, $CD_2Cl_2$) δ 72.09, 157.00, 149.25, 143.02, 141.15, 134.54, 133.59, 129.57, 129.02, 128.59, 126.85, 125.37, 122.72, 110.89, 100.71, 49.56, 48.12, 28.20, 24.17, 21.58. HRMS (ESI) calculated for [M, $C_{44}H_{45}N_2]^{+}$: 601.3577, found: 601.3637.

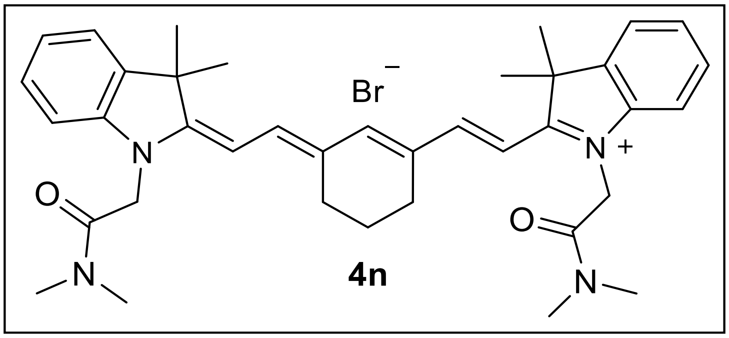

**Synthesis of 4n:** Prepared according to the general procedure **a** from **2a** (64 mg, 0.2 mmol, 1 equiv) and **3l** (130 mg, 0.4 mmol, 2 equiv). Dark-green solid. Yield: 55% (74 mg). $^{1}$H-NMR (600 MHz, $CD_3OD$) δ 7.76 (d, *J* = 13.9 Hz, 2H), 7.49 – 7.44 (m, 3H), 7.37 – 7.33 (m, 2H), 7.25 – 7.21 (m, 2H), 7.14 (d, *J* = 7.9 Hz, 2H), 6.02 (d, *J* = 13.9 Hz, 2H), 5.11 (s, 4H), 3.27 (s, 6H), 3.02 (s, 6H),

2.51 (t, $J$ = 6.1 Hz, 4H), 1.92 – 1.87 (m, 2H), 1.76 (s, 12H). $^{13}$C-NMR (151 MHz, $CD_3OD$) δ 174.38, 166.93, 157.29, 150.36, 144.32, 142.06, 134.19, 129.54, 125.85, 123.24, 111.34, 101.15, 50.42, 36.89, 36.20, 28.08, 24.87, 22.69. HRMS (ESI) calculated for [M, $C_{38}H_{47}N_4O_2$]$^+$: 591.3694, found: 591.3732.

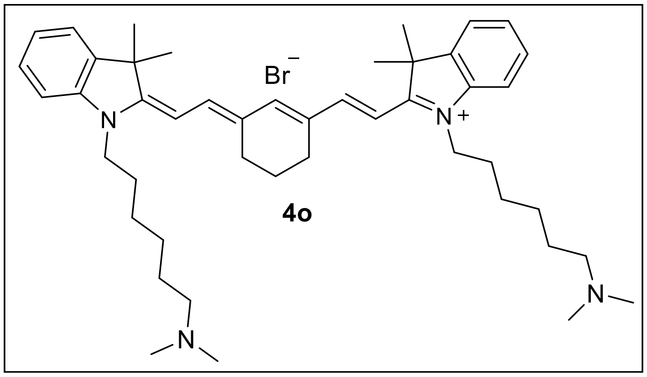

**Synthesis of 4o:** Prepared according to the general procedure **a** from **2a** (64 mg, 0.2 mmol, 1 equiv) and **3o** (178 mg, 0.4 mmol, 2 equiv). Dark-green solid. Yield: 57% (86 mg). $^1$H-NMR (600 MHz, $CD_3OD$) δ 7.79 (d, $J$ = 13.9 Hz, 2H), 7.66 – 7.57 (m, 1H), 7.50 – 7.43 (m, 2H), 7.42 – 7.36 (m, 2H), 7.33 – 7.27 (m, 2H), 7.23 (t, $J$ = 7.4 Hz, 2H), 6.28 – 6.09 (m, 2H), 4.15 (t, $J$ = 7.5 Hz, 4H), 3.20 – 3.15 (m, 4H), 2.90 (s, 12H), 2.59 (brs, 4H), 1.97 – 1.92 (m, 2H), 1.89 – 1.82 (m, 4H), 1.80 – 1.69 (m, 16H), 1.59 – 1.54 (m, 4H), 1.53 – 1.47 (m, 4H). $^{13}$C NMR (151 MHz, MeOD) δ 172.66, 157.01, 149.78, 143.76, 142.39, 133.86, 129.71, 125.83, 123.39, 111.86, 100.84, 58.92, 50.26, 44.84, 43.66, 28.14, 28.09, 27.48, 27.23, 25.52, 25.17, 22.77. HRMS (ESI) calculated for [M, $C_{46}H_{67}N_4$]$^+$: 675.5360, found: 675.5391.

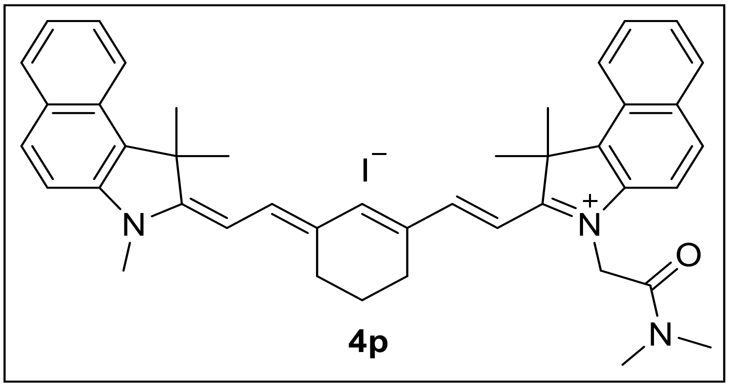

**Synthesis of 4p:** Prepared according to the general procedure **b** from **2a** (65 mg, 0.2 mmol, 1 equiv), **3a** (70 mg, 0.2 mmol, 1 equiv) and **3g** (75 mg, 0.2 mmol, 1 equiv). Dark-green solid. Yield: 52% (77 mg). $^{1}$H-NMR (600 MHz, $CD_3OD$) δ 8.16 – 8.11 (m, 2H), 7.94 – 7.88 (m, 2H), 7.86 – 7.81 (m, 3H), 7.69 (d, $J$ = 13.8 Hz, 1H), 7.56 – 7.49 (m, 3H), 7.43 – 7.38 (m, 2H), 7.35 – 7.32 (m, 1H), 7.30 (d, $J$ = 8.8 Hz, 1H), 6.17 (d, $J$ = 14.3 Hz, 1H), 5.88 (d, $J$ = 13.7 Hz, 1H), 5.08 (s, 2H), 3.67 (s, 3H), 3.22 (s, 3H), 2.94 (s, 3H), 2.50 (t, $J$ = 6.2 Hz, 2H), 2.46 (t, $J$ = 6.2 Hz, 2H), 1.96 (s, 6H), 1.91 (s, 6H), 1.88 – 1.82 (m, 2H). $^{13}$C-NMR (151 MHz, $CD_3OD$) δ 174.77, 172.68, 165.98, 154.83, 149.06, 146.27, 140.60, 140.25, 133.71, 132.79, 132.18, 132.13, 131.72, 130.32, 129.93, 129.74, 129.66, 128.19, 127.96, 127.38, 127.08, 124.76, 124.06, 122.02, 121.85, 110.61, 110.18, 100.14, 98.32, 51.01, 50.46, 44.88, 35.61, 34.87, 30.69, 26.28, 26.00, 23.59, 21.37. HRMS (ESI) calculated for [M, $C_{43}H_{46}N_3O$]$^+$: 620.3635, found: 620.3634.

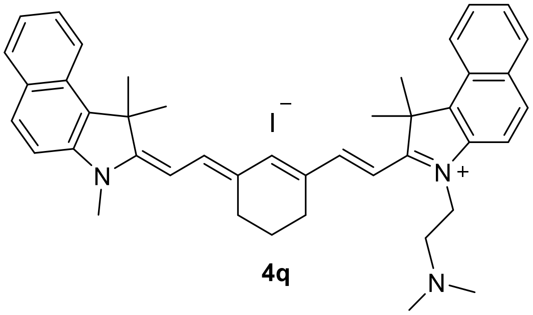


**Synthesis of 4q:**[2] Prepared according to the general procedure **b** from **3a** (70 mg, 0.2 mmol, 1 equiv), **2a** (65 mg, 0.2 mmol, 1 equiv) and **3h** (88 mg, 0.2 mmol, 1 equiv). Dark-green solid. Yield: 53% (77 mg). $^{1}$H-NMR (600 MHz, $CD_3OD$) δ 8.24 – 8.18 (m, 2H), 8.01 – 7.92 (m, 5H), 7.83 (d, *J* = 13.9 Hz, 1H), 7.65 – 7.57 (m, 4H), 7.52 (d, *J* = 8.8 Hz, 1H), 7.48 – 7.41 (m, 2H), 6.24 (d, *J* = 14.2 Hz, 1H), 6.18 (d, *J* = 13.9 Hz, 1H), 4.28 (t, *J* = 7.4 Hz, 2H), 3.75 (s, 3H), 2.77 – 2.72 (m, 2H), 2.60 (t, *J* = 6.2 Hz, 4H), 2.41 (s, 6H), 2.03 – 1.93 (m, 14H). $^{13}$C-NMR (151 MHz, $CD_3OD$) δ 175.75, 173.16, 156.23, 149.99, 147.70, 141.67, 141.20, 134.93, 134.06, 133.63, 133.47, 133.13, 131.66, 131.59, 131.11, 131.07, 129.57, 129.36, 128.73, 128.60, 126.04, 125.64, 123.39, 123.27, 111.98, 111.64, 101.27, 99.81, 56.61, 52.29, 51.81, 46.01, 43.08, 32.13, 27.68, 27.48, 25.02, 22.77. HRMS (ESI) calculated for [M, $C_{43}H_{48}N_3$]$^{+}$: 606.3843, found: 606.3847.

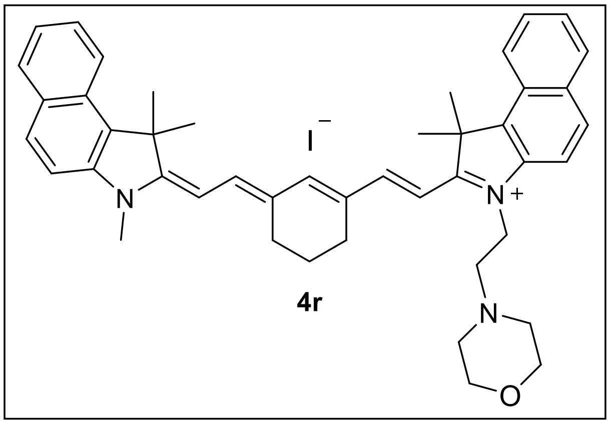

**Synthesis of 4r:** Prepared according to the general procedure **b** from **3a** (70 mg, 0.2 mmol, 1 equiv), **2a** (65 mg, 0.2 mmol, 1 equiv) and **3k** (88 mg, 0.2 mmol, 1 equiv). Dark-green solid. Yield: 55% (85 mg). $^{1}$H-NMR (600 MHz, $CD_2Cl_2$) δ 8.21 – 8.09 (m, 2H), 8.00 – 7.88 (m, 4H), 7.81 (d, *J* = 14.0 Hz, 2H), 7.67 – 7.52 (m, 3H), 7.50 – 7.37 (m, 4H), 6.16 (d, *J* = 14.1 Hz, 1H), 6.08 (d, *J* = 14.2 Hz, 1H), 4.29 (t, *J* = 6.5 Hz, 2H), 3.71 (s, 3H), 3.59 (t, *J* = 4.6 Hz, 4H), 2.83 (t, *J* = 6.5 Hz, 2H), 2.65 – 2.46 (m, 8H), 2.11 – 1.88 (m, 14H). $^{13}$C-NMR (151 MHz, $CD_2Cl_2$) δ 173.46, 155.87, 147.89, 147.78, 140.64, 140.10, 133.67, 133.56, 132.87, 132.71, 132.09, 130.76, 130.68, 130.31, 130.28, 128.51, 128.49, 127.97, 125.17, 125.12, 122.60, 122.58, 111.14, 110.82, 99.90, 99.66, 67.16, 55.66, 54.51, 51.24, 51.16, 43.12, 32.31, 30.00, 27.66, 27.56, 24.43, 21.82. HRMS (ESI) calculated for [M, $C_{45}H_{50}N_3O]^+$: 648.3948, found: 648.3983.

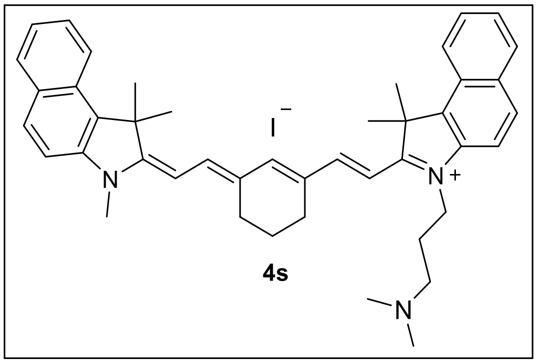


**Synthesis of 4s:** Prepared according to the general procedure **b** from **3a** (70 mg, 0.2 mmol, 1 equiv), **2a** (65 mg, 0.2 mmol, 1 equiv) and **3i** (91 mg, 0.2 mmol, 1 equiv). Dark-green solid. Yield: 51% (76 mg). $^{1}$H-NMR (600 MHz, $CD_3OD$) δ 8.23 (d, *J* = 8.6 Hz, 1H), 8.20 (d, *J* = 8.4 Hz, 1H), 8.02 – 7.92 (m, 5H), 7.84 (d, *J* = 13.0 Hz, 1H), 7.65 – 7.56 (m, 5H), 7.48 – 7.41 (m, 2H), 6.29 – 6.16 (m, 2H), 4.27 (t, *J* = 7.4 Hz, 2H), 3.76 (s, 3H), 2.83 – 2.75 (m, 2H), 2.65 – 2.56 (m, 4H), 2.49 (s, 6H), 2.15 – 2.10 (m, 2H), 2.03 – 1.93 (m, 14H). $^{13}$C-NMR (151 MHz, $CD_3OD$) δ 175.77, 172.98, 156.27, 150.03, 147.77, 141.67, 141.21, 134.95, 134.11, 133.74, 133.71, 133.49, 133.12, 131.67, 131.61, 131.11, 131.07, 129.59, 129.36, 128.73, 128.61, 126.05, 125.65, 123.40, 123.28, 111.98, 111.82, 101.27, 99.78, 56.90, 52.31, 51.82, 44.99, 42.47, 32.14, 27.71, 27.48, 25.47, 25.18, 25.05, 22.79. HRMS (ESI) calculated for $[M, C_{44}H_{50}N_3]^+$: 620.3999, found: 620.4000.

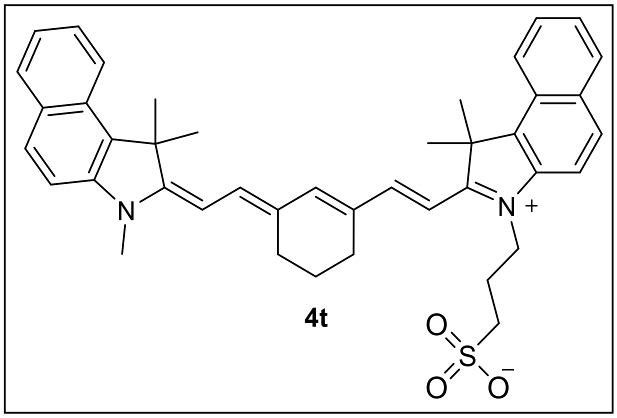


**Synthesis of 4t:** Prepared according to the general procedure **b** from **2a** (97 mg, 0.3 mmol, 1 equiv),

**3a** (105 mg, 0.3 mmol, 1 equiv) and **3j** (99 mg, 0.3 mmol, 1 equiv). Dark-green solid. Yield: 56% (110 mg). $^{1}$H-NMR (600 MHz, $CD_3OD$) δ 8.23 – 8.16 (m, 2H), 7.99 – 7.91 (m, 4H), 7.87 – 7.79 (m, 2H), 7.64 – 7.54 (m, 4H), 7.53 – 7.48 (m, 1H), 7.46 – 7.41 (m, 2H), 6.32 (d, *J* = 12.5 Hz, 1H), 6.15 (d, *J* = 13.8 Hz, 1H), 4.41 (t, *J* = 7.9 Hz, 2H), 3.71 (s, 3H), 3.01 (d, *J* = 6.7 Hz, 2H), 2.63 – 2.56 (m, 2H), 2.55 – 2.47 (m, 2H), 2.33 – 2.28 (m, 2H), 2.02 – 1.86 (m, 14H). $^{13}$C-NMR (151 MHz, $CD_3OD$) δ 174.76, 173.91, 156.30, 149.01, 141.80, 141.14, 135.12, 134.49, 134.29, 133.96, 133.31, 133.24, 131.69, 131.57, 131.08, 131.07, 129.54, 129.44, 128.64, 128.56, 125.80, 123.35, 123.30, 111.96, 111.83, 100.55, 52.01, 49.57, 43.83, 31.84, 27.62, 27.51, 25.00, 24.28, 22.75. HRMS (ESI) calculated for [M+H, $C_{42}H_{45}N_2O_3S$]$^+$: 657.3037, found: 657.3165.

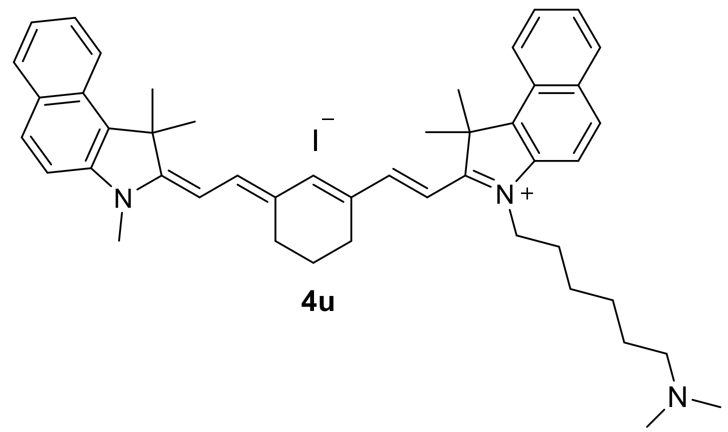

**4u**

**Synthesis of 4u:**[2] Prepared according to the general procedure **b** from **3a** (70 mg, 0.2 mmol, 1 equiv), **2a** (65 mg, 0.2 mmol, 1 equiv) and **3q** (99 mg, 0.2 mmol, 1 equiv). Dark-green solid. Yield: 52% (82 mg). $^{1}$H-NMR (600 MHz, $CD_3OD$) δ 8.26 – 8.21 (m, 2H), 8.02 – 7.96 (m, 4H), 7.93 – 7.84 (m, 2H), 7.65 – 7.61 (m, 2H), 7.60 – 7.54 (m, 3H), 7.49 – 7.45 (m, 2H), 6.25 – 6.17 (m, 2H), 4.25 (t, *J* = 7.4 Hz, 2H), 3.75 (s, 3H), 3.09 – 3.04 (m, 2H), 2.81 (s, 6H), 2.67 – 2.56 (m, 4H), 2.07

– 1.95 (m, 14H), 1.94 – 1.89 (m, 2H), 1.76 – 1.71 (m, 2H), 1.61 – 1.56 (m, 2H), 1.52 – 1.48 (m, 2H). $^{13}$C-NMR (151 MHz, $CD_3OD$) δ 175.09, 173.71, 156.26, 149.28, 148.48, 141.78, 141.27, 134.62, 134.47, 133.81, 133.57, 133.36, 133.22, 131.64, 131.61, 131.10, 131.08, 129.53, 129.43, 128.67, 128.66, 125.88, 125.80, 123.35, 123.32, 112.07, 111.87, 100.71, 100.15, 59.08, 52.11, 52.00, 44.90, 43.76, 31.97, 28.39, 27.68, 27.55, 27.53, 27.38, 25.80, 25.17, 25.02, 22.80. HRMS (ESI) calculated for [M, $C_{47}H_{56}N_3$]$^+$: 662.4469, found: 662.4476.

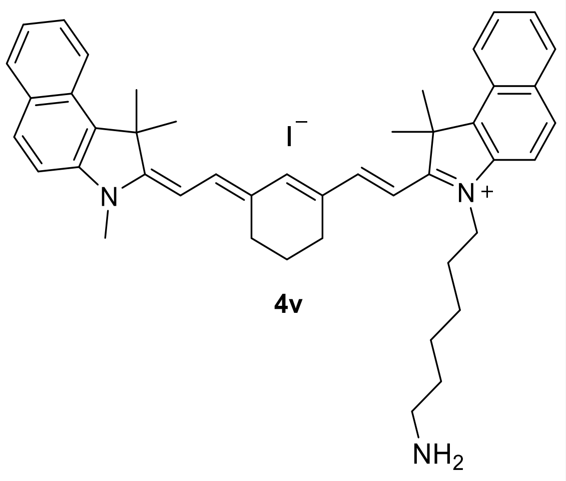

**4v**

**Synthesis of 4v:** Prepared according to the general procedure **b** from **3a** (70 mg, 0.2 mmol, 1 equiv), **2a** (65 mg, 0.2 mmol, 1 equiv) and **3r** (94 mg, 0.2 mmol, 1 equiv). Dark-green solid. Yield: 45% (68 mg). $^1$H-NMR (600 MHz, $CD_3OD$) δ 8.26 – 8.19 (m, 2H), 8.03 – 7.95 (m, 4H), 7.92 – 7.83 (m, 2H), 7.65 – 7.56 (m, 4H), 7.54 (s, 1H), 7.49 – 7.44 (m, 2H), 6.25 – 6.17 (m, 2H), 4.25 (t, *J* = 7.4 Hz, 2H), 3.74 (s, 3H), 2.95 – 2.90 (m, 2H), 2.64 – 2.58 (m, 4H), 2.04 – 1.96 (m, 14H), 1.94 – 1.88 (m, 2H), 1.72 – 1.66 (m, 2H), 1.58 – 1.54 (m, 2H), 1.53 – 1.49 (m, 2H). $^{13}$C-NMR (151 MHz, $CD_3OD$) δ 175.21, 173.65, 156.10, 149.40, 148.36, 141.78, 141.29, 134.67, 134.44, 133.83, 133.51, 133.40, 133.22, 131.64, 131.11, 131.09, 129.99, 129.54, 129.42, 128.69, 128.68, 125.93,

125.85, 125.81, 123.35, 123.31, 111.97, 111.88, 100.80, 100.07, 52.13, 51.97, 44.80, 40.64, 31.90, 28.52, 28.40, 27.62, 27.56, 27.47, 27.29, 25.04, 22.80. HRMS (ESI) calculated for [M, $C_{45}H_{52}N_3$]$^+$: 634.4156, found: 634.4153.

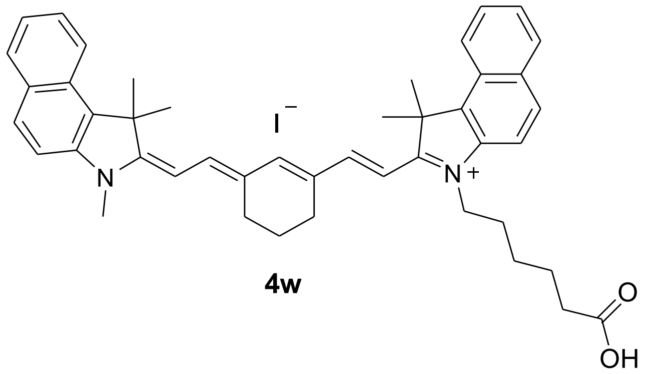


4w

**Synthesis of 4w:**[2] Prepared according to the general procedure **b** from **3a** (70 mg, 0.2 mmol, 1 equiv), **2a** (65 mg, 0.2 mmol, 1 equiv) and **3s** (81 mg, 0.2 mmol, 1 equiv). Dark-green solid. Yield: 53% (82 mg). $^1$H-NMR (600 MHz, $CD_3OD$) δ 8.12 (d, *J* = 8.5 Hz, 2H), 7.91 – 7.85 (m, 4H), 7.84 – 7.69 (m, 2H), 7.55 – 7.51 (m, 2H), 7.50 – 7.32 (m, 5H), 6.21 – 5.95 (m, 2H), 4.20 – 4.06 (m, 2H), 3.63 (s, 3H), 2.68 – 2.30 (m, 4H), 2.23 (t, *J* = 7.3 Hz, 2H), 1.99 – 1.84 (m, 14H), 1.83 – 1.77 (m, 2H), 1.65 – 1.60 (m, 2H), 1.48 – 1.41 (m, 2H); $^{13}$C-NMR (151 MHz, $CD_3OD$) δ 177.49, 174.88, 173.98, 156.12, 149.06, 148.67, 141.83, 141.22, 134.55, 133.35, 133.29, 132.30, 131.66, 131.61, 131.10, 129.68, 129.53, 129.46, 128.66, 125.84, 124.39, 123.33, 113.51, 111.95, 111.83, 100.54, 100.28, 52.05, 44.83, 34.81, 31.83, 28.22, 27.60, 27.50, 27.41, 25.78, 25.02, 22.78, 22.07. HRMS (ESI) calculated for [M, $C_{45}H_{49}N_2O_2$]$^+$: 649.3789, found: 649.3793.

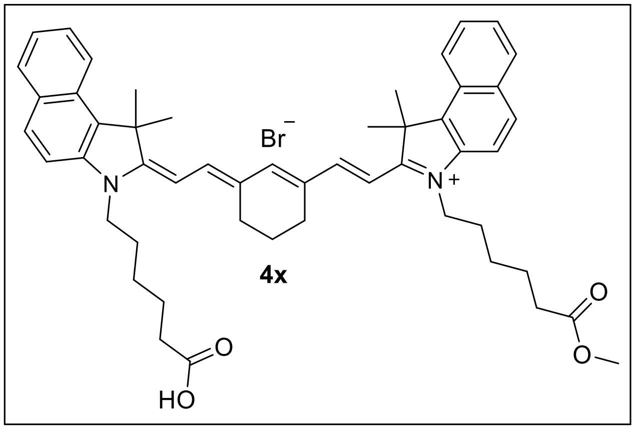


**Synthesis of 4x:** Prepared according to the general procedure **b** from **3s** (81 mg, 0.2 mmol, 1 equiv), **2a** (65 mg, 0.2 mmol, 1 equiv) and **3t** (83 mg, 0.2 mmol, 1 equiv). Dark-green solid. Yield: 54% (91 mg). $^1$H-NMR (600 MHz, $CD_3OD$) δ 8.21 (d, *J* = 8.5 Hz, 2H), 8.00 – 7.95 (m, 4H), 7.89 – 7.82 (m, 2H), 7.64 – 7.60 (m, 2H), 7.55 (dd, *J* = 8.8, 5.5 Hz, 2H), 7.50 (s, 1H), 7.46 (t, *J* = 7.5 Hz, 2H), 6.20 (t, *J* = 11.9 Hz, 2H), 4.25 – 4.17 (m, 4H), 3.61 (s, 3H), 2.59 (brs, 4H), 2.36 (t, *J* = 7.3 Hz, 2H), 2.31 (t, *J* = 7.3 Hz, 2H), 2.01 – 1.95 (m, 14H), 1.91 – 1.85 (m, 4H), 1.74 – 1.68 (m, 4H), 1.56 – 1.49 (m, 4H). $^{13}$C-NMR (151 MHz, $CD_3OD$) δ 178.66, 175.59, 174.18, 173.99, 156.09, 148.95, 148.71, 141.18, 134.67, 134.58, 133.69, 133.31, 133.28, 131.69, 131.67, 131.10, 129.51, 129.49, 128.68, 125.91, 125.87, 123.33, 123.31, 111.99, 111.95, 100.45, 100.31, 52.11, 52.05, 52.02, 44.89, 44.77, 35.55, 34.48, 28.27, 28.16, 27.57, 27.55, 27.50, 27.31, 26.04, 25.64, 25.00, 22.78. HRMS (ESI) calculated for [M, $C_{51}H_{59}N_2O_4$]$^+$: 763.4469, found: 763.4455.

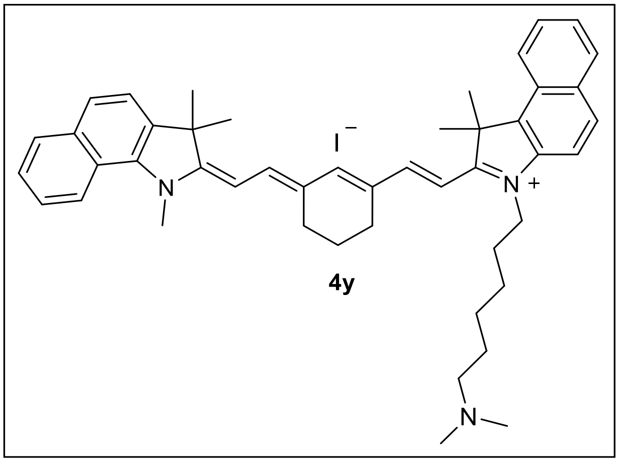

**Synthesis of 4y:** Prepared according to the general procedure **b** from **3b** (97 mg, 0.3 mmol, 1 equiv), **2a** (97 mg, 0.3 mmol, 1 equiv) and **3q** (149 mg, 0.3 mmol, 1 equiv). Dark-green solid. Yield: 51% (120 mg). $^{1}$H-NMR (600 MHz, $CD_3OD$) δ 8.58 (d, *J* = 8.7 Hz, 1H), 8.23 (d, *J* = 8.5 Hz, 1H), 8.03 – 7.94 (m, 3H), 7.92 – 7.86 (m, 1H), 7.85 – 7.75 (m, 2H), 7.65 – 7.44 (m, 7H), 6.35 – 6.15 (m, 2H), 4.33 – 4.08 (m, 5H), 3.13 – 3.07 (m, 2H), 2.84 (s, 6H), 2.74 – 2.48 (m, 4H), 2.07 – 1.67 (m, 18H), 1.62 – 1.56 (m, 2H), 1.53 – 1.48 (m, 2H). $^{13}$C-NMR (151 MHz, $CD_3OD$) δ 174.63, 174.45, 156.23, 149.26, 149.01, 141.15, 139.66, 139.07, 136.44, 134.83, 133.98, 133.83, 133.36, 131.71, 131.09, 130.72, 129.47, 128.73, 127.85, 127.19, 126.95, 125.99, 123.38, 122.85, 122.76, 120.44, 112.16, 101.32, 100.73, 59.02, 52.21, 49.93, 45.05, 43.68, 37.55, 28.46, 27.99, 27.61, 27.55, 27.35, 25.71, 25.18, 25.08, 22.81. HRMS (ESI) calculated for [M, $C_{47}H_{56}N_3$]$^+$: 662.4469, found: 662.4520.

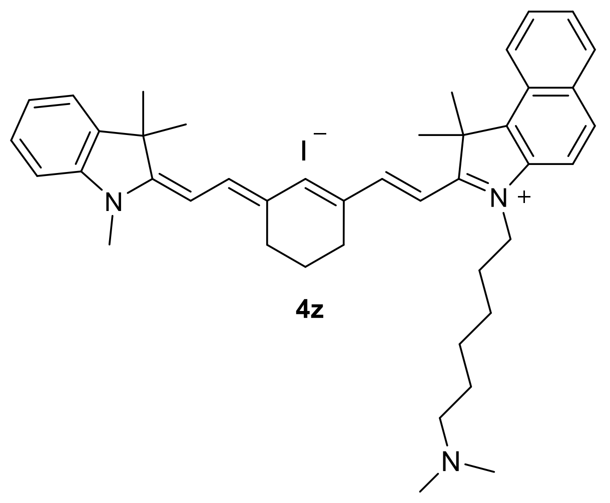

**4z**

**Synthesis of 4z:** Prepared according to the general procedure **b** from **3d** (90 mg, 0.3 mmol, 1 equiv), **2a** (97 mg, 0.3 mmol, 1 equiv) and **3q** (149 mg, 0.3 mmol, 1 equiv). Dark-green solid. Yield: 53% (117 mg). $^{1}$H-NMR (600 MHz, $CD_3OD$) δ 8.23 (d, *J* = 8.7 Hz, 1H), 8.03 – 7.95 (m, 2H), 7.90 (d, *J* = 14.1 Hz, 1H), 7.76 (d, *J* = 14.0 Hz, 1H), 7.65 – 7.55 (m, 3H), 7.49 – 7.42 (m, 2H), 7.40 – 7.36 (m, 1H), 7.25 – 7.18 (m, 2H), 6.24 (d, *J* = 14.1 Hz, 1H), 6.12 (d, *J* = 13.8 Hz, 1H), 4.28 (t, *J* = 7.5 Hz, 2H), 3.59 (s, 3H), 3.13 – 3.07 (m, 2H), 2.83 (s, 6H), 2.68 – 2.47 (m, 4H), 2.04 – 1.85 (m, 10H), 1.79 – 1.65 (m, 8H), 1.62 – 1.56 (m, 2H), 1.53 – 1.47 (m, 2H). $^{13}$C-NMR (151 MHz, $CD_3OD$) δ 174.66, 172.79, 156.59, 149.59, 149.07, 144.51, 142.18, 141.11, 134.87, 133.75, 133.36, 131.72, 131.09, 129.63, 129.45, 128.72, 125.99, 125.60, 123.38, 123.21, 112.21, 111.26, 100.76, 100.44, 59.03, 52.24, 50.01, 45.09, 43.73, 31.43, 28.45, 28.04, 27.63, 27.52, 27.34, 25.73, 25.17, 24.99, 22.77. HRMS (ESI) calculated for [M, $C_{43}H_{54}N_3$]$^{+}$: 612.4312, found: 612.4375.

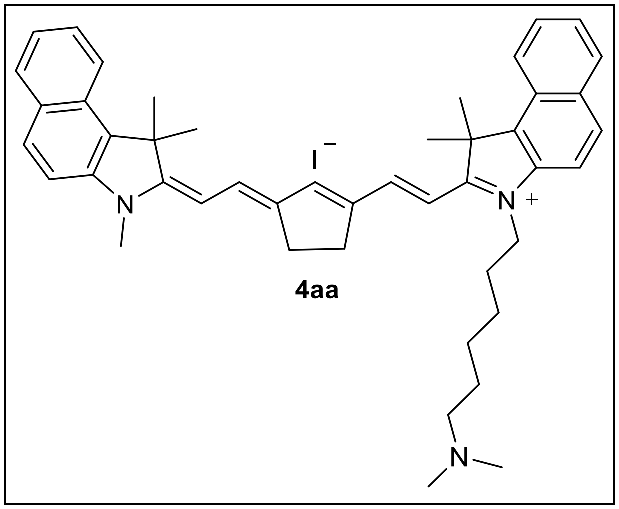

**Synthesis of 4aa:** Prepared according to the general procedure **b** from **3a** (71 mg, 0.2 mmol, 1 equiv), **2b** (62 mg, 0.2 mmol, 1 equiv) and **3q** (99 mg, 0.2 mmol, 1 equiv). Dark-green solid. Yield: 54% (83 mg). $^{1}$H-NMR (600 MHz, $CD_2Cl_2$) δ 8.16 – 8.09 (m, 2H), 8.01 – 7.91 (m, 4H), 7.87 – 7.78 (m, 2H), 7.64 – 7.57 (m, 2H), 7.54 – 7.37 (m, 5H), 6.00 (d, *J* = 14.0 Hz, 1H), 5.88 (d, *J* = 13.8 Hz, 1H), 4.20 (t, *J* = 7.7 Hz, 2H), 3.66 (s, 3H), 3.33 – 3.27 (m, 2H), 3.10 – 3.94 (m, 4H), 2.88 (s, 6H), 2.00 – 1.90 (m, 14H), 1.68 – 1.50 (m, 6H). $^{13}$C-NMR (151 MHz, $CD_2Cl_2$) δ 172.44, 171.86, 156.61, 145.10, 144.97, 140.83, 140.47, 140.12, 139.22, 134.03, 133.39, 132.28, 132.03, 131.10, 130.79, 130.42, 130.37, 128.64, 128.56, 128.02, 128.00, 125.36, 125.07, 122.56, 122.53, 111.40, 110.66, 102.61, 101.76, 57.70, 51.38, 50.96, 44.98, 43.13, 31.97, 30.07, 28.81, 28.42, 27.62, 27.58, 27.53, 26.68, 26.63, 24.53. HRMS (ESI) calculated for [M, $C_{46}H_{54}N_3$]$^{+}$: 648.4312, found: 648.4318.

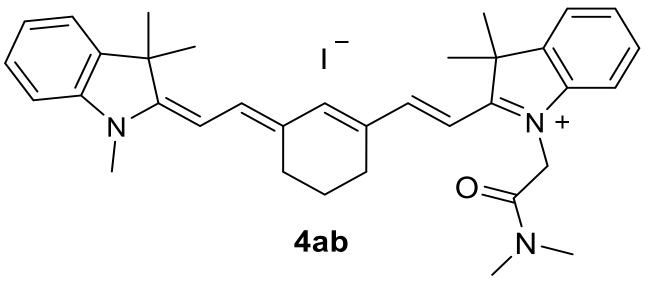

4ab

**Synthesis of 4ab:** Prepared according to the general procedure **b** from **2a** (64 mg, 0.2 mmol, 1 equiv), **3d** (60 mg, 0.2 mmol, 1 equiv) and **3l** (65 mg, 0.2 mmol, 1 equiv). Dark-green solid. Yield: 50% (65 mg). $^{1}$H-NMR (600 MHz, $CD_3OD$) δ 7.83 (d, $J$ = 14.1 Hz, 1H), 7.70 (d, $J$ = 13.6 Hz, 1H), 7.53 (s, 1H), 7.50 – 7.48 (m, 1H), 7.44 – 7.39 (m, 2H), 7.33 – 7.29 (m, 2H), 7.27 (t, $J$ = 7.4 Hz, 1H), 7.17 (t, $J$ = 7.4 Hz, 1H), 7.09 (d, $J$ = 7.9 Hz, 1H), 6.22 (d, $J$ = 14.3 Hz, 1H), 5.99 – 5.89 (m, 1H), 5.07 (s, 2H), 3.65 (s, 3H), 3.28 (s, 3H), 3.02 (s, 3H), 2.61 – 2.44 (m, 4H), 1.93 – 1.88 (m, 2H), 1.75 (s, 6H), 1.72 (s, 6H). $^{13}$C-NMR (151 MHz, $CD_3OD$) δ 173.34, 171.38, 165.81, 155.59, 149.90, 147.26, 143.17, 142.87, 141.18, 140.40, 132.89, 132.41, 128.37, 128.04, 125.01, 123.89, 121.93, 121.78, 110.58, 109.56, 100.42, 98.88, 49.16, 48.63, 44.80, 35.73, 34.88, 30.43, 26.87, 26.46, 23.58, 21.32. HRMS (ESI) calculated for [M, $C_{35}H_{42}N_3O]^+$: 520.3322, found: 520.3379.

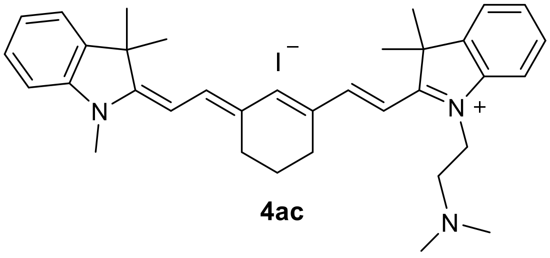

4ac

**Synthesis of 4ac:** Prepared according to the general procedure **b** from **2a** (97 mg, 0.3 mmol, 1 equiv), **3d** (90 mg, 0.3 mmol, 1 equiv) and **3m** (117 mg, 0.3 mmol, 1 equiv). Dark-green solid. Yield: 54% (102 mg). $^{1}$H-NMR (600 MHz, $CD_3OD$) δ 7.84 (d, $J$ = 14.2 Hz, 1H), 7.73 (d, $J$ = 13.9

Hz, 1H), 7.54 – 7.49 (m, 2H), 7.48 – 7.43 (m, 2H), 7.43 – 7.38 (m, 1H), 7.34 – 7.32 (m, 1H), 7.31 – 7.27 (m, 1H), 7.25 – 7.20 (m, 2H), 6.25 (d, *J* = 14.2 Hz, 1H), 6.18 (d, *J* = 13.8 Hz, 1H), 4.20 (t, *J* = 7.4 Hz, 2H), 3.67 (s, 3H), 2.72 (t, *J* = 7.4 Hz, 2H), 2.63 – 2.57 (m, 4H), 2.42 (s, 6H), 1.99 – 1.94 (m, 2H), 1.74 (s, 6H), 1.73 (s, 6H). $^{13}$C-NMR (151 MHz, $CD_3OD$) δ 174.53, 171.75, 156.73, 151.00, 148.55, 144.31, 143.80, 142.52, 142.08, 134.18, 133.74, 129.75, 129.67, 126.32, 125.51, 123.37, 123.30, 111.88, 111.11, 101.65, 100.21, 56.29, 50.49, 49.95, 45.92, 42.85, 31.67, 28.11, 27.82, 24.97, 22.74. HRMS (ESI) calculated for [M, $C_{35}H_{44}N_3$]$^+$: 506.3530, found: 506.3535.

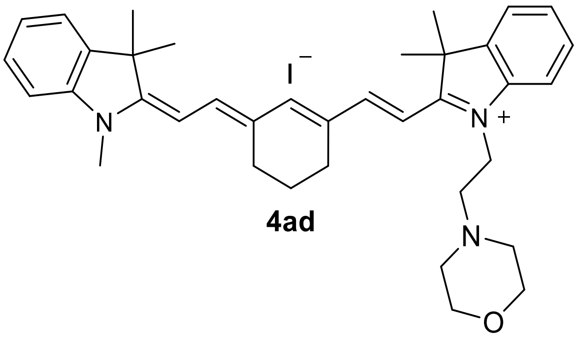


**4ad**

**Synthesis of 4ad:** Prepared according to the general procedure **b** from **2a** (65 mg, 0.2 mmol, 1 equiv), **3d** (60 mg, 0.2 mmol, 1 equiv) and **3n** (70 mg, 0.2 mmol, 1 equiv). Dark-green solid. Yield: 53% (71 mg). $^{1}$H-NMR (600 MHz, $CD_3OD$) δ 7.82 – 7.72 (m, 2H), 7.55 (s, 1H), 7.49 – 7.45 (m, 2H), 7.42 – 7.36 (m, 2H), 7.28 – 7.20 (m, 4H), 6.25 – 6.15 (m, 2H), 4.24 (t, *J* = 6.7 Hz, 2H), 3.65 – 3.59 (m, 7H), 2.75 (t, *J* = 6.7 Hz, 2H), 2.61 – 2.53 (m, 8H), 1.96 – 1.91 (m, 2H), 1.72 (s, 12H). $^{13}$C-NMR (151 MHz, $CD_3OD$) δ 173.76, 172.83, 156.68, 150.17, 149.19, 144.39, 143.77, 142.36, 142.25, 133.92, 133.65, 129.69, 129.62, 125.99, 125.72, 123.35, 123.26, 111.61, 101.11, 101.00, 67.96, 55.95, 55.08, 50.27, 50.17, 42.73, 31.60, 31.57, 28.13, 27.92, 25.03, 22.74. HRMS (ESI) calculated for [M, $C_{37}H_{46}N_3O$]$^+$: 548.3635, found: 548.3692.

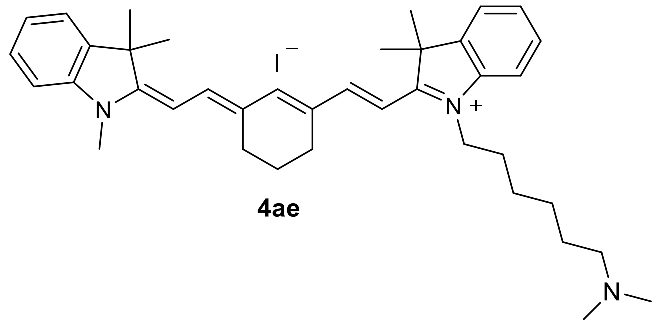

**Synthesis of 4ae:** Prepared according to the general procedure **b** from **3d** (90 mg, 0.3 mmol, 1 equiv), **2a** (97 mg, 0.3 mmol, 1 equiv) and **3o** (134 mg, 0.3 mmol, 1 equiv). Yield: 56% (116 mg). $^{1}$H-NMR (600 MHz, $CD_3OD$) δ 7.82 – 7.72 (m, 2H), 7.54 (s, 1H), 7.50 – 7.45 (m, 2H), 7.42 – 7.37 (m, 2H), 7.29 – 7.20 (m, 4H), 6.22 – 6.12 (m, 2H), 4.13 (t, *J* = 7.4 Hz, 2H), 3.62 (s, 3H), 3.09 – 3.04 (m, 2H), 2.82 (s, 6H), 2.58 (t, *J* = 6.2 Hz, 4H), 1.98 – 1.92 (m, 2H), 1.89 – 1.83 (m, 2H), 1.78 – 1.67 (m, 14H), 1.58 – 1.52 (m, 2H), 1.51 – 1.45 (m, 2H). $^{13}$C-NMR (151 MHz, $CD_3OD$) δ 173.77, 172.36, 156.86, 150.21, 149.41, 144.40, 143.85, 142.36, 142.32, 133.91, 133.67, 129.70, 125.98, 125.74, 123.38, 123.26, 111.69, 111.59, 101.04, 100.56, 59.10, 50.26, 50.17, 44.73, 43.77, 31.56, 28.10, 27.92, 27.56, 27.37, 25.81, 25.11, 24.97, 22.75. HRMS (ESI) calculated for $[M, C_{39}H_{52}N_3]^+$: 562.4156, found: 562.4183.

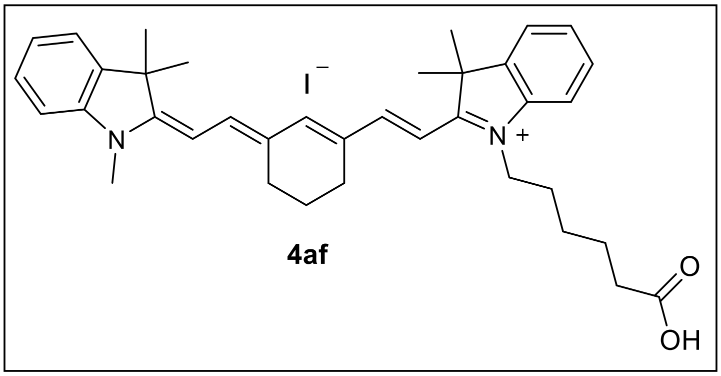

**Synthesis of 4af:** Prepared according to the general procedure **b** from **2a** (65 mg, 0.2 mmol, 1 equiv), **3d** (60 mg, 0.2 mmol, 1 equiv) and **3p** (71 mg, 0.2 mmol, 1 equiv). Dark-green solid. Yield: 53% (72 mg). $^1$H-NMR (600 MHz, $CD_3OD$) δ 7.83 – 7.69 (m, 2H), 7.55 – 7.43 (m, 3H), 7.42 – 7.36 (m, 2H), 7.29 – 7.18 (m, 4H), 6.24 – 6.08 (m, 2H), 4.15 – 4.06 (m, 2H), 3.69 – 3.54 (m, 3H) , 2.68 – 2.45 (m, 4H), 2.30 (t, $J$ = 7.3 Hz, 2H), 1.98 – 1.91 (m, 2H), 1.86 – 1.81 (m, 2H), 1.78 – 1.60 (m, 14H), 1.53 – 1.48 (m, 2H). $^{13}$C-NMR (151 MHz, $CD_3OD$) δ 178.14, 173.52, 172.65, 156.82, 149.96, 149.67, 144.44, 143.80, 142.37, 142.31, 133.84, 133.74, 129.71, 129.71, 129.68, 125.88, 125.84, 123.37, 123.24, 111.68, 111.49, 100.85, 100.69, 50.23, 50.19, 44.69, 35.20, 31.45, 28.03, 27.94, 27.92, 27.47, 25.90, 24.96, 22.73. HRMS (ESI) calculated for [M, $C_{37}H_{45}N_2O_2$]$^+$: 549.3476, found: 549.3510.

## 3. Photophysical properties

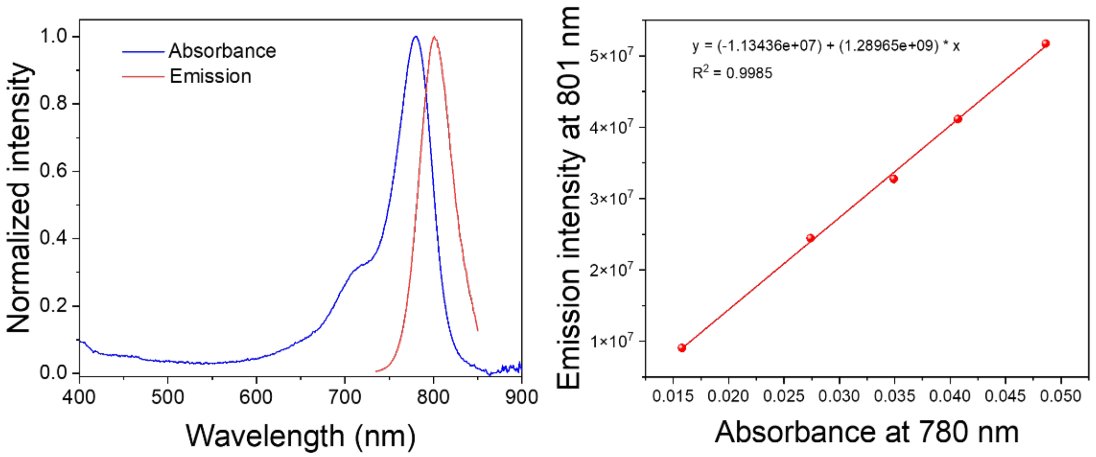


**Figure S1.** (left) UV-Vis absorption and emission spectra of **4a** in methanol. (right) Linear plot of integrated emission intensity at 801 nm versus absorbance at 780 nm for **4a**.

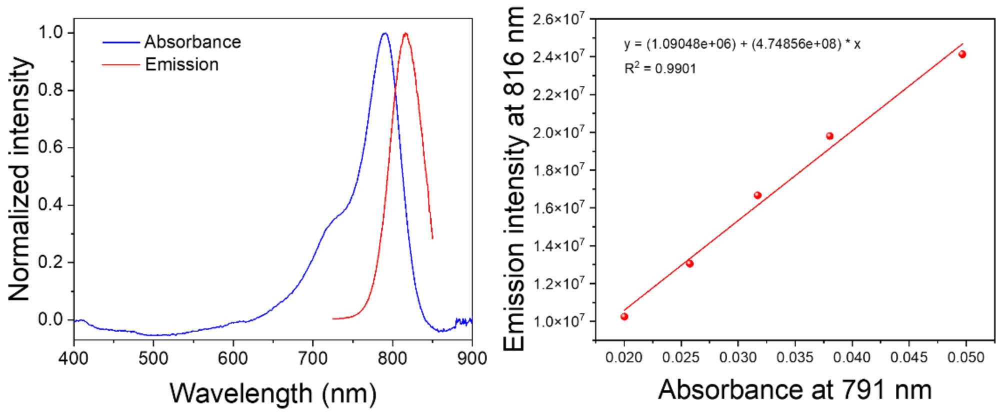


**Figure S2.** (left) UV-Vis absorption and emission spectra of **4b** in methanol. (right) Linear plot of integrated emission intensity at 816 nm versus absorbance at 791 nm for **4b**.

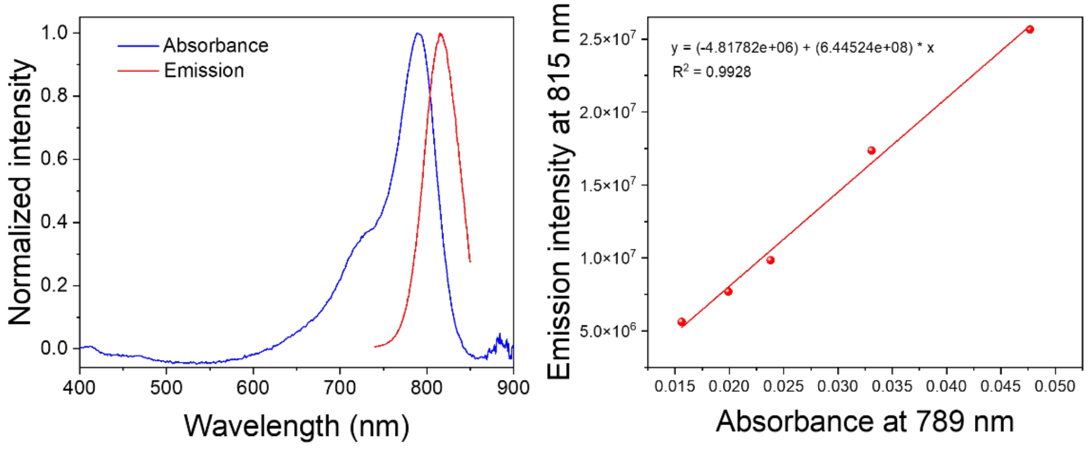

**Figure S3.** (left) UV-Vis absorption and emission spectra of **4c** in methanol. (right) Linear plot of integrated emission intensity at 815 nm versus absorbance at 789 nm for **4c**.

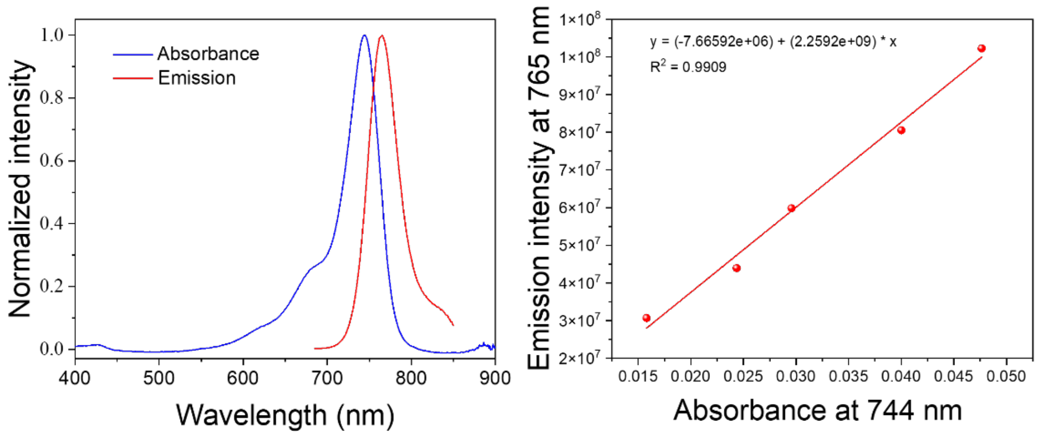


**Figure S4.** (left) UV-Vis absorption and emission spectra of **4d** in methanol. (right) Linear plot of integrated emission intensity at 765 nm versus absorbance at 744 nm for **4d**.

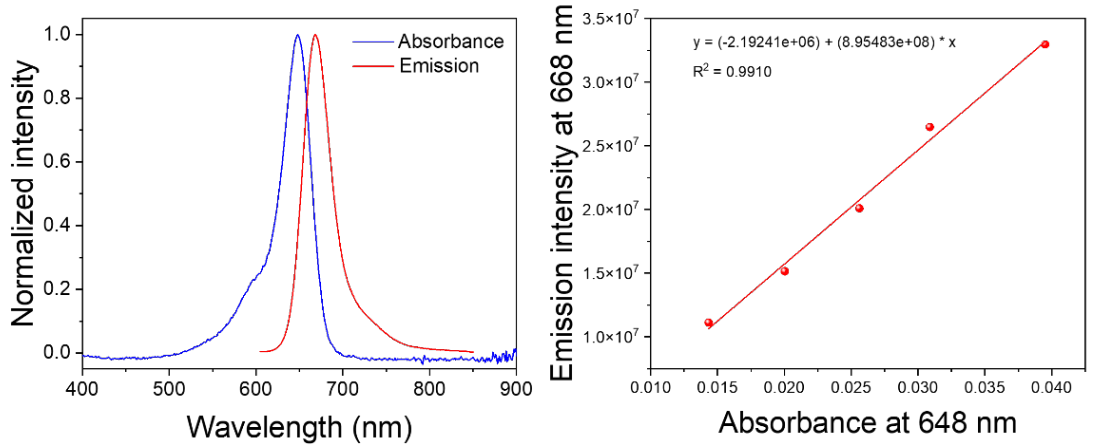


**Figure S5.** (left) UV-Vis absorption and emission spectra of **4e** in methanol. (right) Linear plot of integrated emission intensity at 668 nm versus absorbance at 648 nm for **4e**.

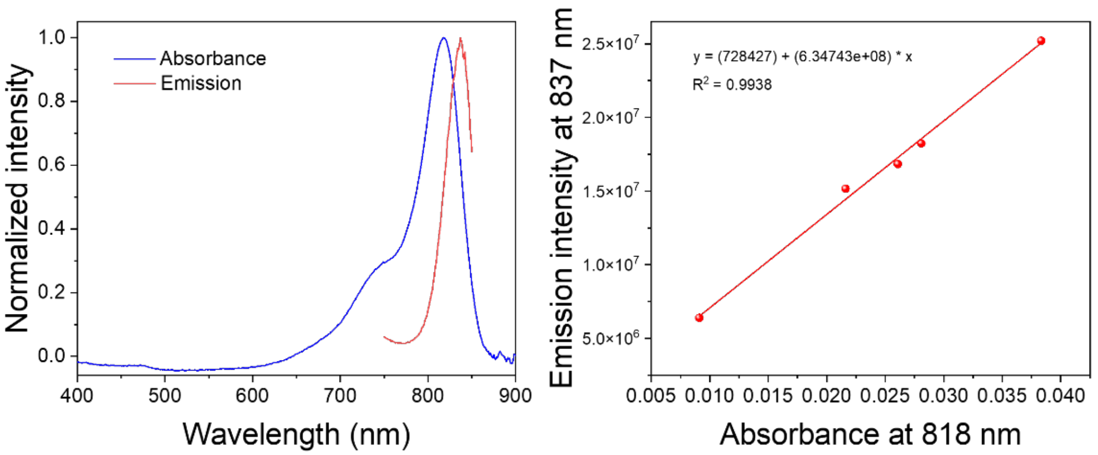


**Figure S6.** (left) UV-Vis absorption and emission spectra of **4f** in methanol. (right) Linear plot of integrated emission intensity at 837 nm versus absorbance at 818 nm for **4f**.

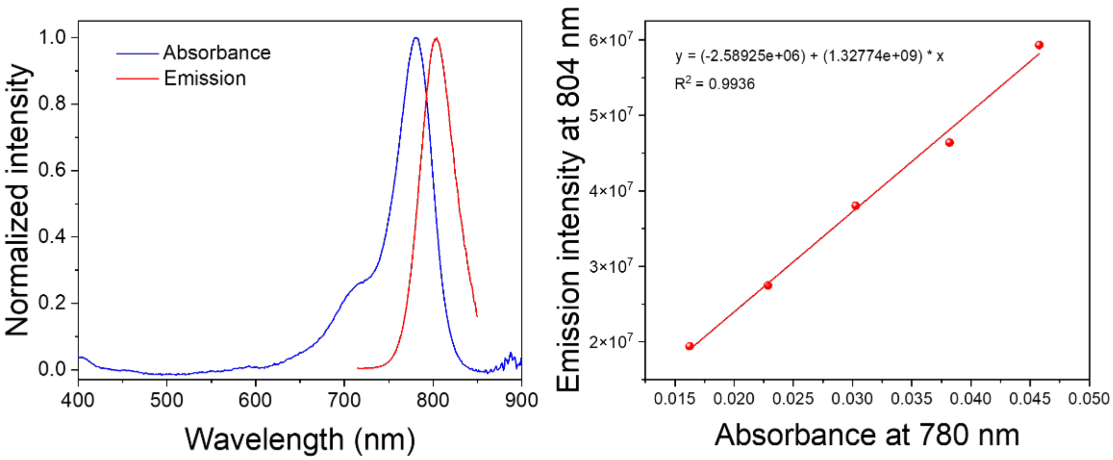


**Figure S7.** (left) UV-Vis absorption and emission spectra of **4g** in methanol. (right) Linear plot of integrated emission intensity at 804 nm versus absorbance at 780 nm for **4g**.

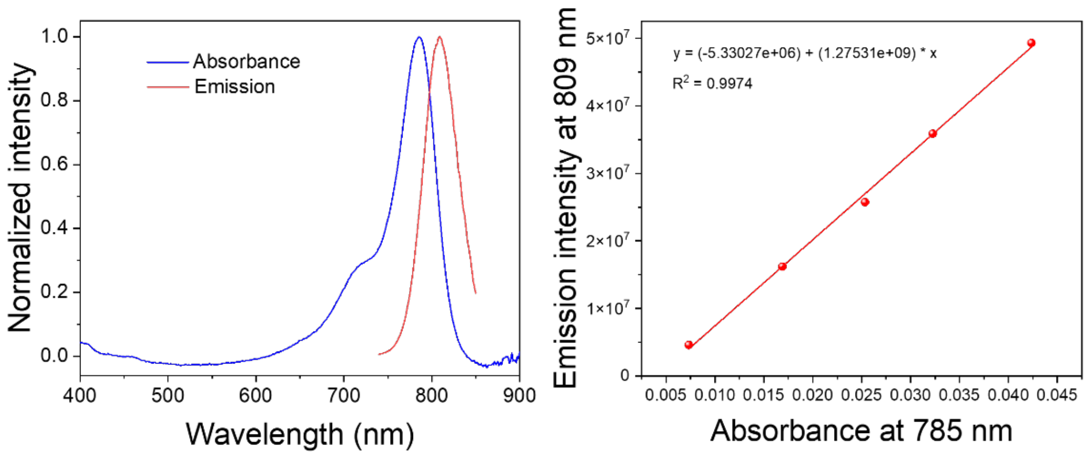


**Figure S8.** (left) UV-Vis absorption and emission spectra of **4h** in methanol. (right) Linear plot of integrated emission intensity at 809 nm versus absorbance at 785 nm for **4h**.

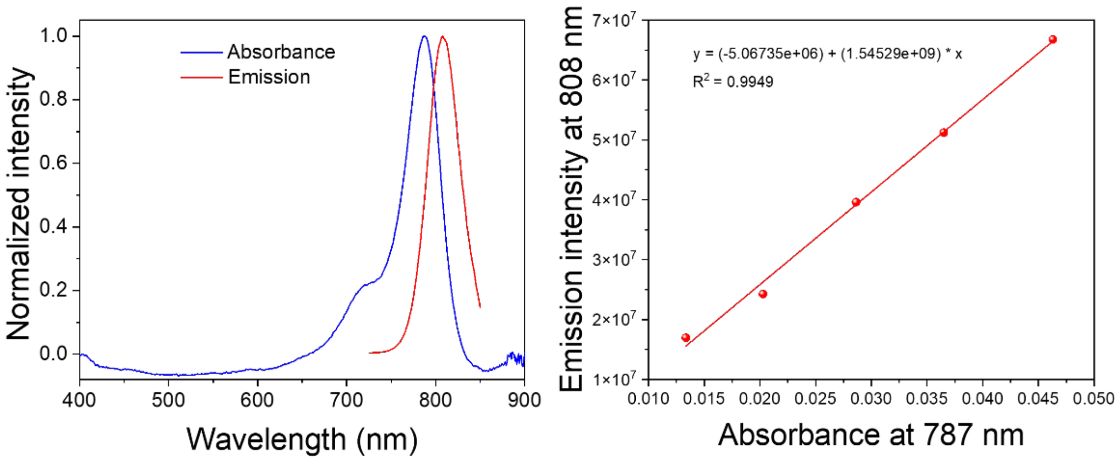


**Figure S9.** (left) UV-Vis absorption and emission spectra of **4i** in methanol. (right) Linear plot of integrated emission intensity at 808 nm versus absorbance at 787 nm for **4i**.

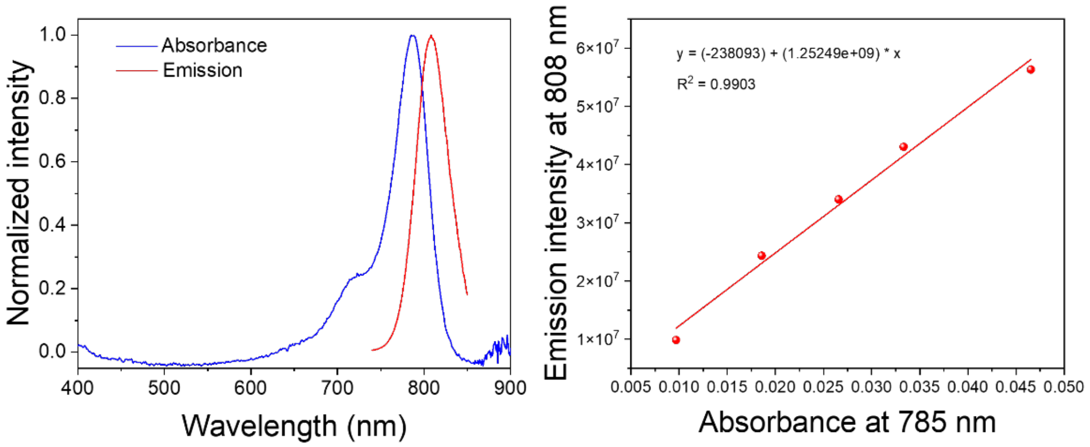


**Figure S10.** (left) UV-Vis absorption and emission spectra of **4j** in methanol. (right) Linear plot of integrated emission intensity at 808 nm versus absorbance at 785 nm for **4j**.

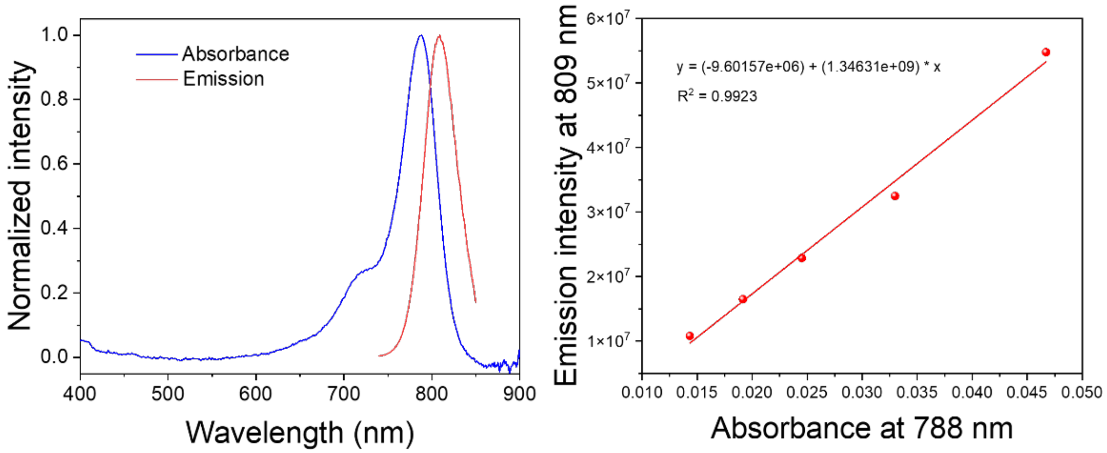


**Figure S11.** (left) UV-Vis absorption and emission spectra of **4k** in methanol. (right) Linear plot of integrated emission intensity at 809 nm versus absorbance at 788 nm for **4k**.

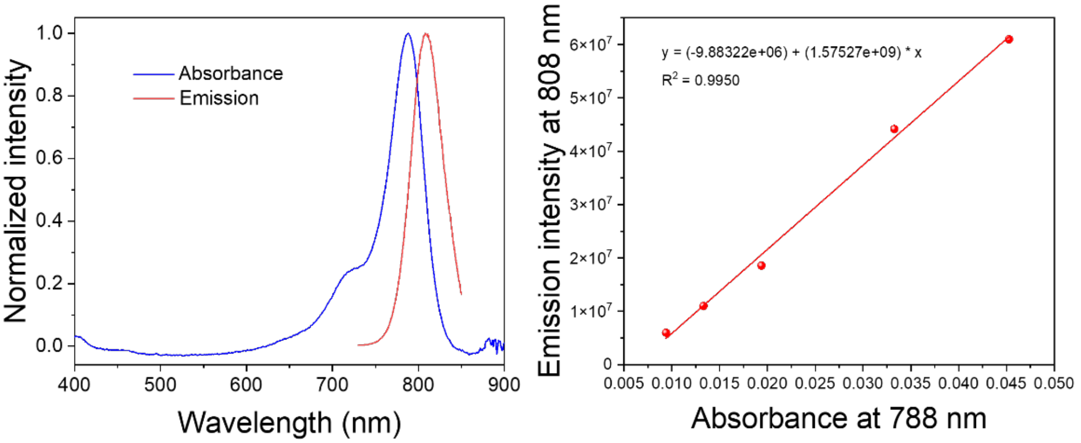


**Figure S12.** (left) UV-Vis absorption and emission spectra of **4l** in methanol. (right) Linear plot of integrated emission intensity at 808 nm versus absorbance at 788 nm for **4l**.

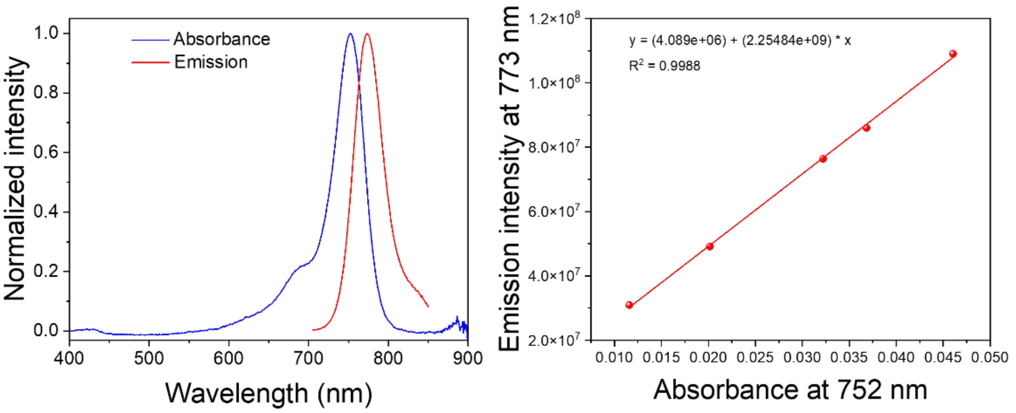


**Figure S13.** (left) UV-Vis absorption and emission spectra of **4m** in methanol. (right) Linear plot of integrated emission intensity at 773 nm versus absorbance at 752 nm for **4m**.

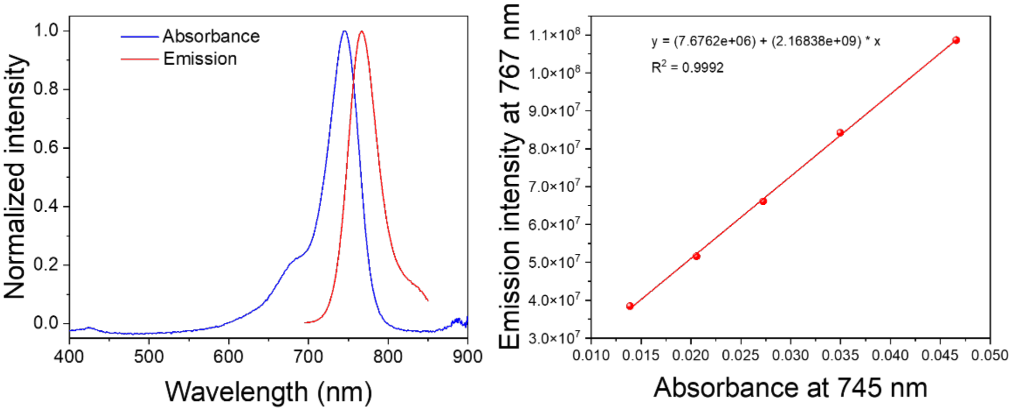


**Figure S14.** (left) UV-Vis absorption and emission spectra of **4n** in methanol. (right) Linear plot

of integrated emission intensity at 767 nm versus absorbance at 745 nm for **4n**.

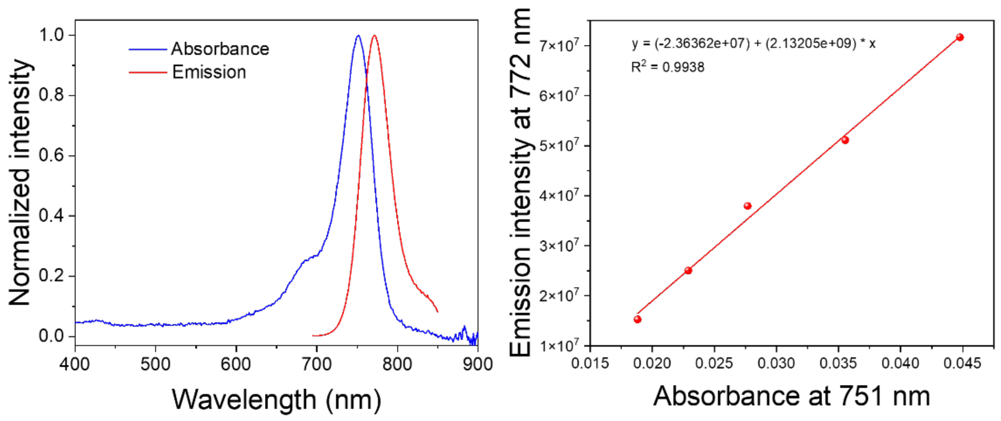


**Figure S15.** (left) UV-Vis absorption and emission spectra of **4o** in methanol. (right) Linear plot of integrated emission intensity at 772 nm versus absorbance at 751 nm for **4o**.

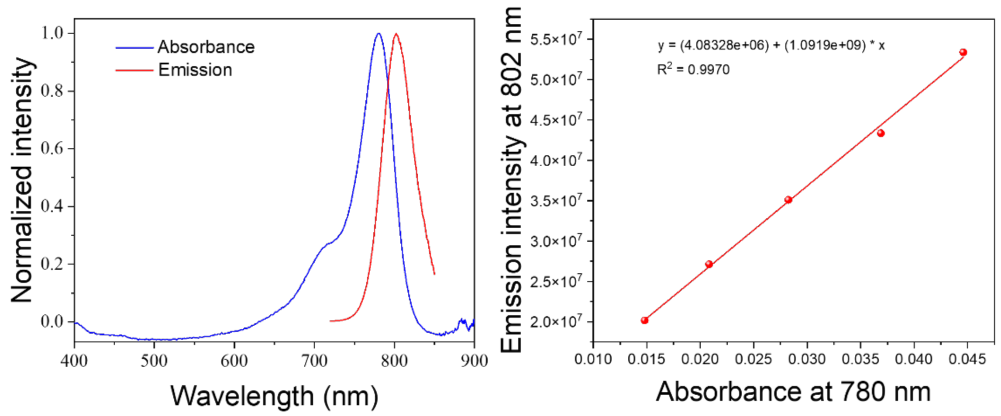


**Figure S16.** (left) UV-Vis absorption and emission spectra of **4p** in methanol. (right) Linear plot of integrated emission intensity at 802 nm versus absorbance at 780 nm for **4p**.

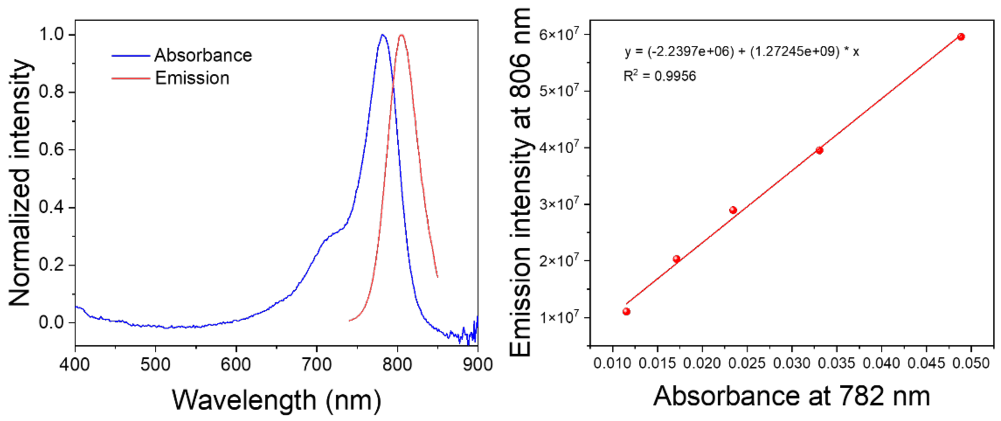

**Figure S17.** (left) UV-Vis absorption and emission spectra of **4q** in methanol. (right) Linear plot of integrated emission intensity at 806 nm versus absorbance at 782 nm for **4q**.

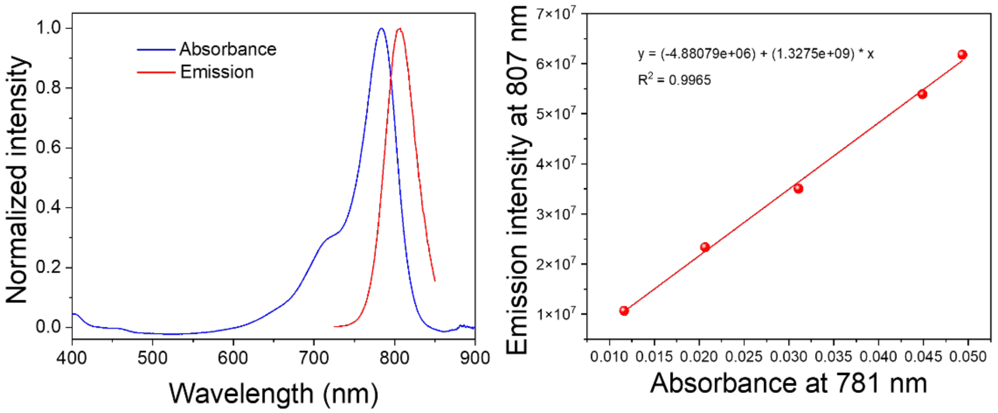


**Figure S18.** (left) UV-Vis absorption and emission spectra of **4r** in methanol. (right) Linear plot of integrated emission intensity at 807 nm versus absorbance at 781 nm for **4r**.

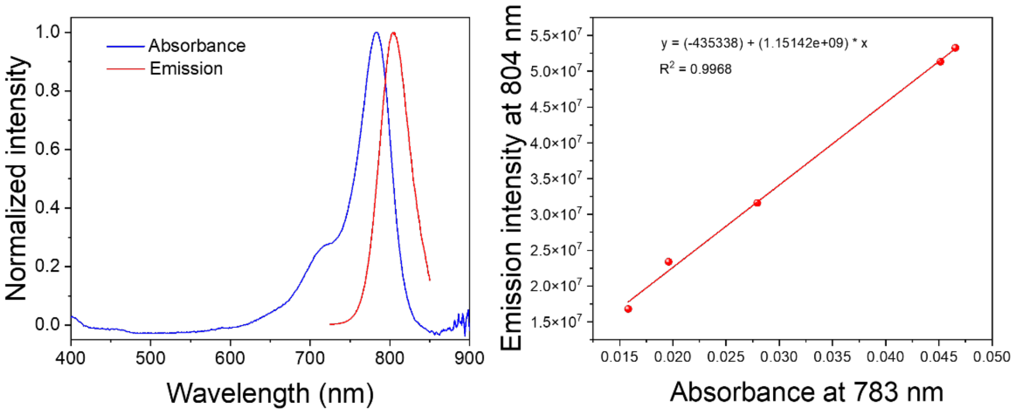


**Figure S19.** (left) UV-Vis absorption and emission spectra of **4s** in methanol. (right) Linear plot of integrated emission intensity at 804 nm versus absorbance at 783 nm for **4s**.

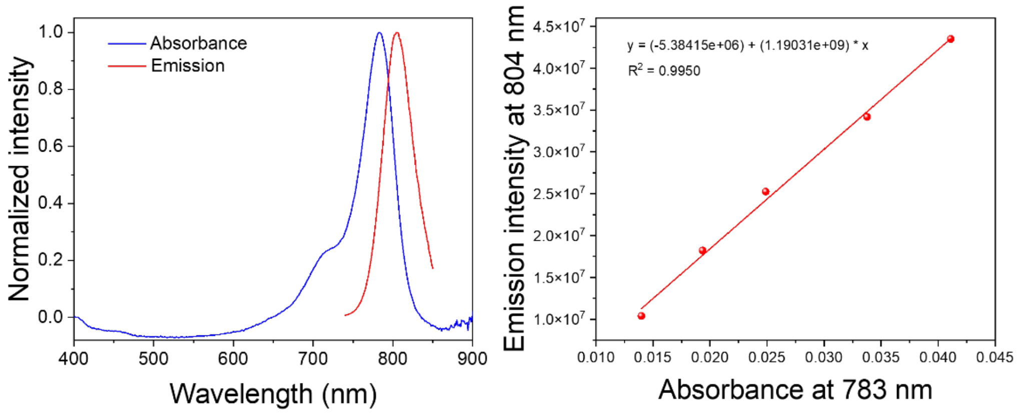


**Figure S20.** (left) UV-Vis absorption and emission spectra of **4t** in methanol. (right) Linear plot of integrated emission intensity at 804 nm versus absorbance at 783 nm for **4t**.

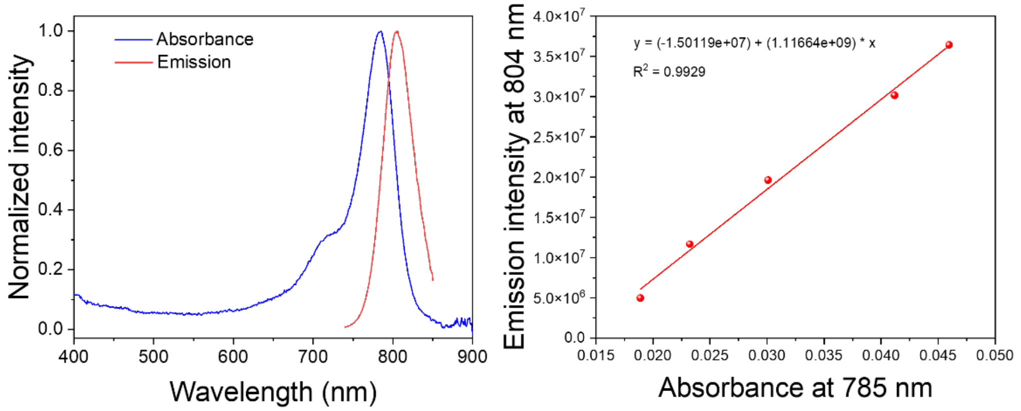


**Figure S21.** (left) UV-Vis absorption and emission spectra of **4u** in methanol. (right) Linear plot of integrated emission intensity at 804 nm versus absorbance at 785 nm for **4u**.

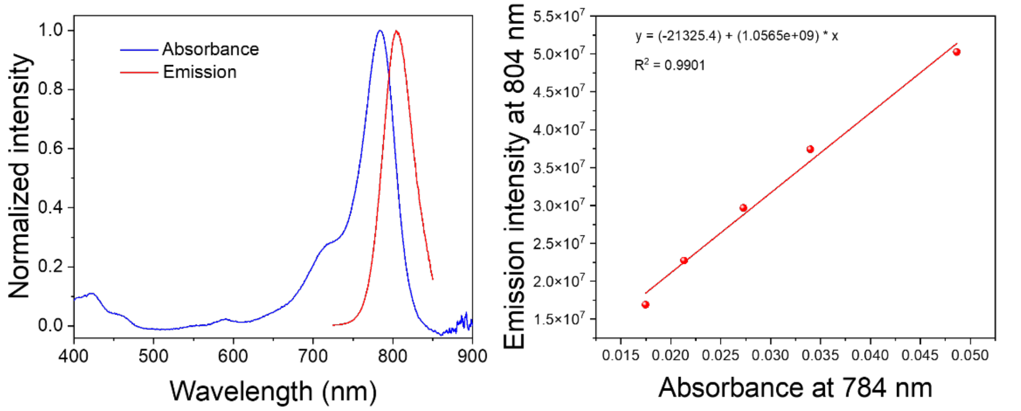


**Figure S22.** (left) UV-Vis absorption and emission spectra of **4v** in methanol. (right) Linear plot

of integrated emission intensity at 804 nm versus absorbance at 784 nm for **4v**.

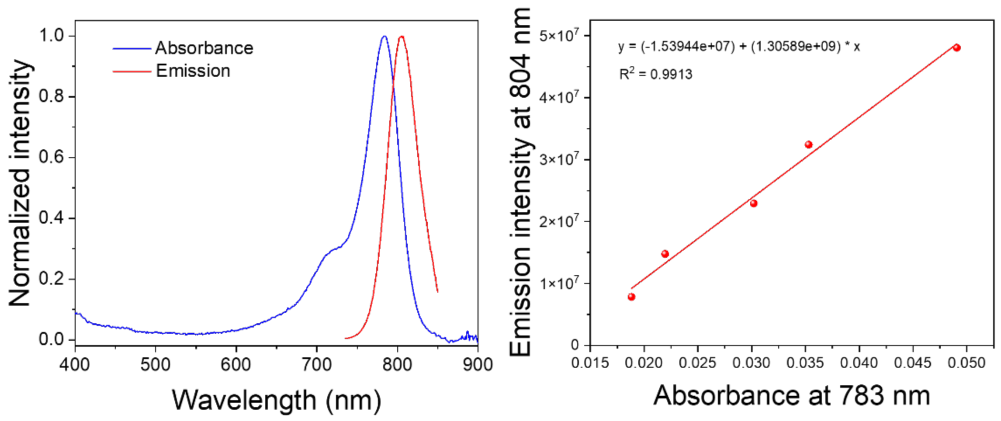


**Figure S23.** (left) UV-Vis absorption and emission spectra of **4w** in methanol. (right) Linear plot of integrated emission intensity at 804 nm versus absorbance at 783 nm for **4w**.

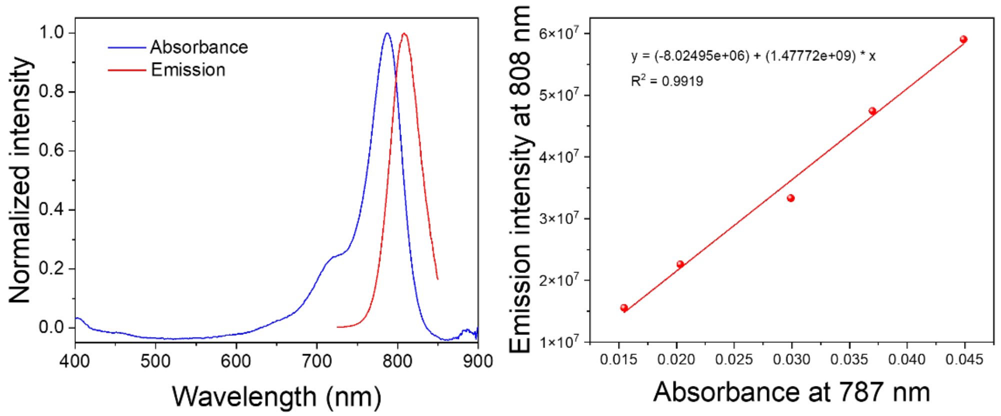


**Figure S24.** (left) UV-Vis absorption and emission spectra of **4x** in methanol. (right) Linear plot of integrated emission intensity at 808 nm versus absorbance at 787 nm for **4x**.

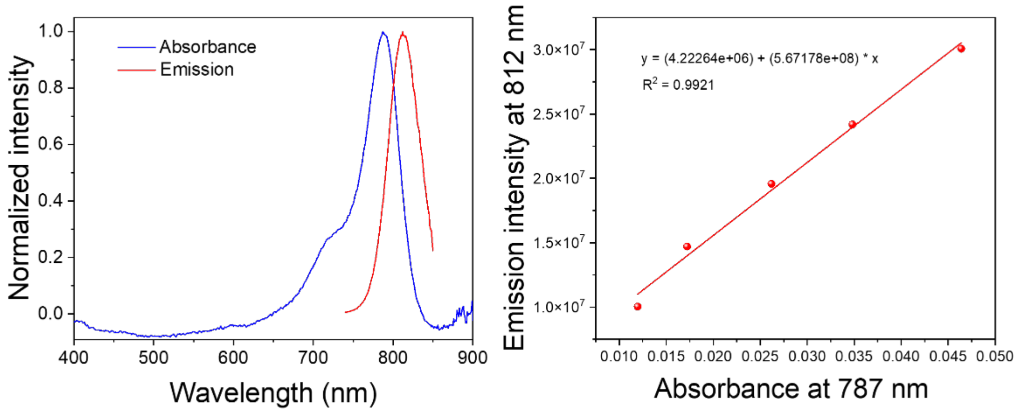

**Figure S25.** (left) UV-Vis absorption and emission spectra of **4y** in methanol. (right) Linear plot of integrated emission intensity at 812 nm versus absorbance at 787 nm for **4y**.

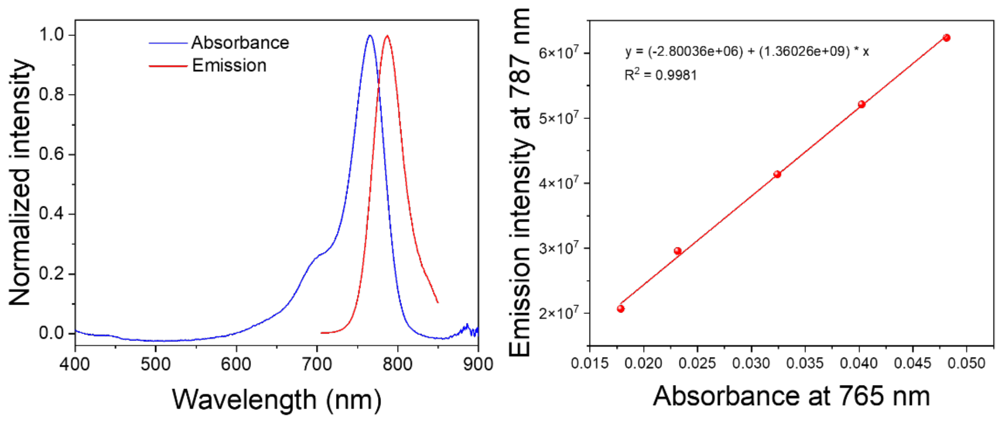


**Figure S26.** (left) UV-Vis absorption and emission spectra of **4z** in methanol. (right) Linear plot of integrated emission intensity at 787 nm versus absorbance at 765 nm for **4z**.

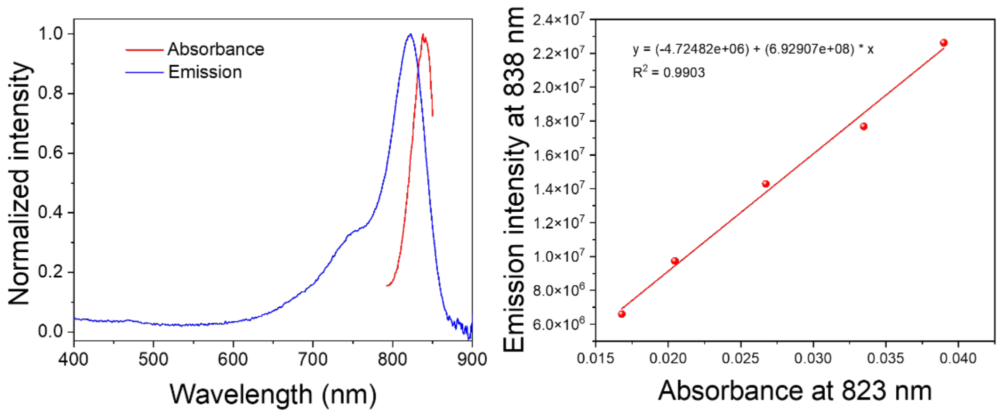


**Figure S27.** (left) UV-Vis absorption and emission spectra of **4aa** in methanol. (right) Linear plot of integrated emission intensity at 838 nm versus absorbance at 823 nm for **4aa**.

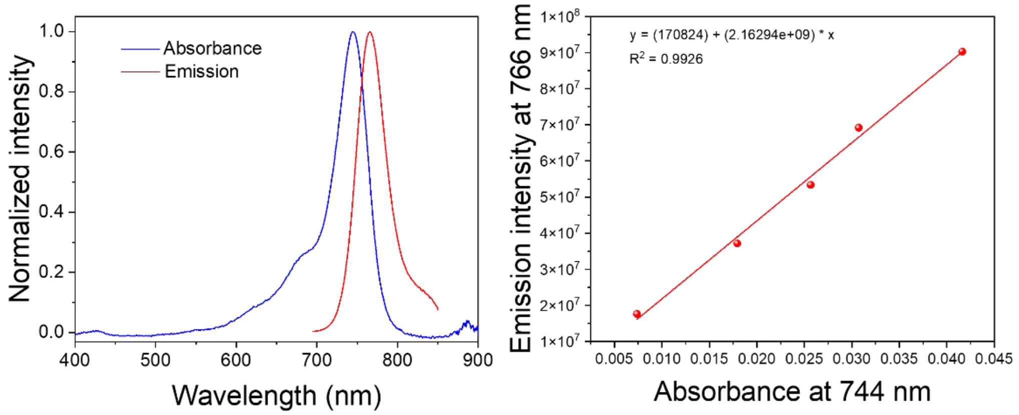


**Figure S28.** (left) UV-Vis absorption and emission spectra of **4ab** in methanol. (right) Linear plot of integrated emission intensity at 766 nm versus absorbance at 744 nm for **4ab**.

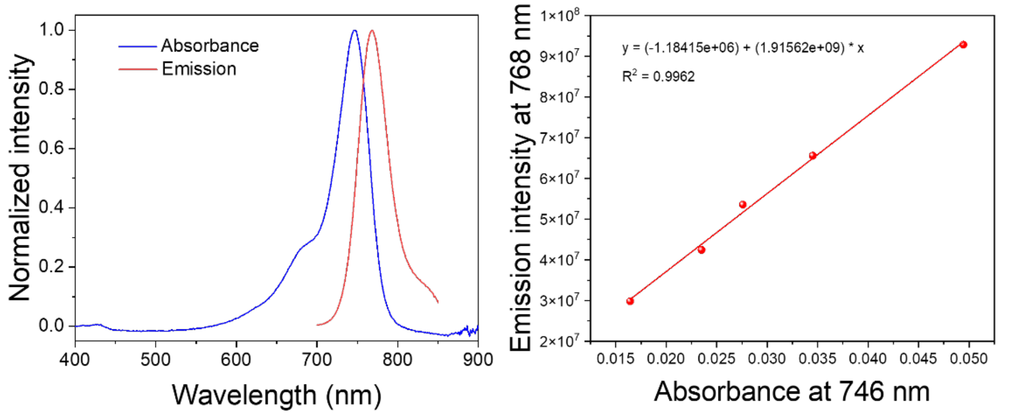


**Figure S29.** (left) UV-Vis absorption and emission spectra of **4ac** in methanol. (right) Linear plot of integrated emission intensity at 768 nm versus absorbance at 746 nm for **4ac**.

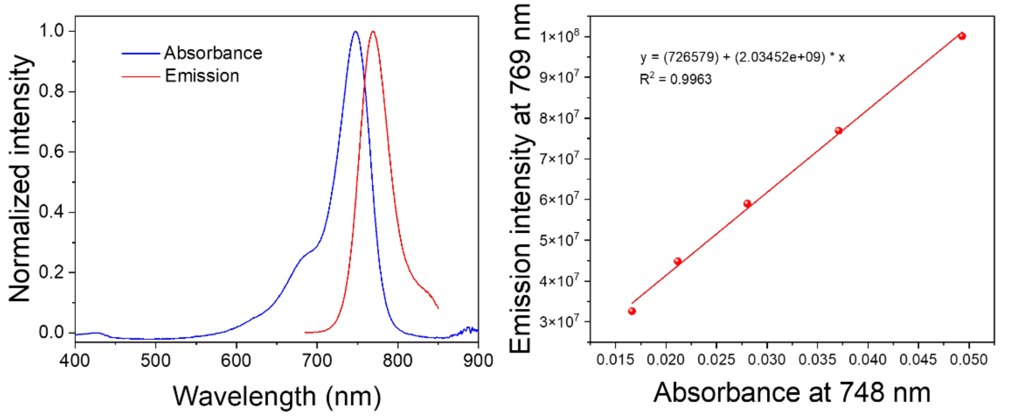


**Figure S30.** (left) UV-Vis absorption and emission spectra of **4ad** in methanol. (right) Linear plot

of integrated emission intensity at 769 nm versus absorbance at 748 nm for **4ad**.

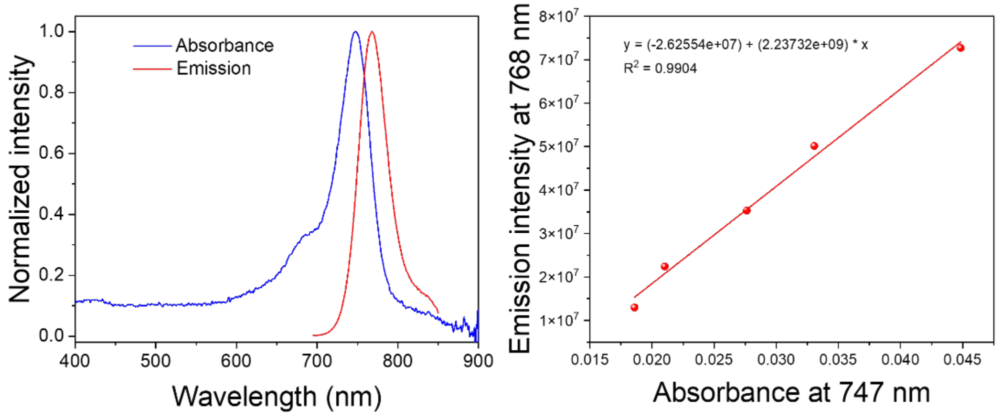


**Figure S31.** (left) UV-Vis absorption and emission spectra of **4ae** in methanol. (right) Linear plot of integrated emission intensity at 768 nm versus absorbance at 747 nm for **4ae**.

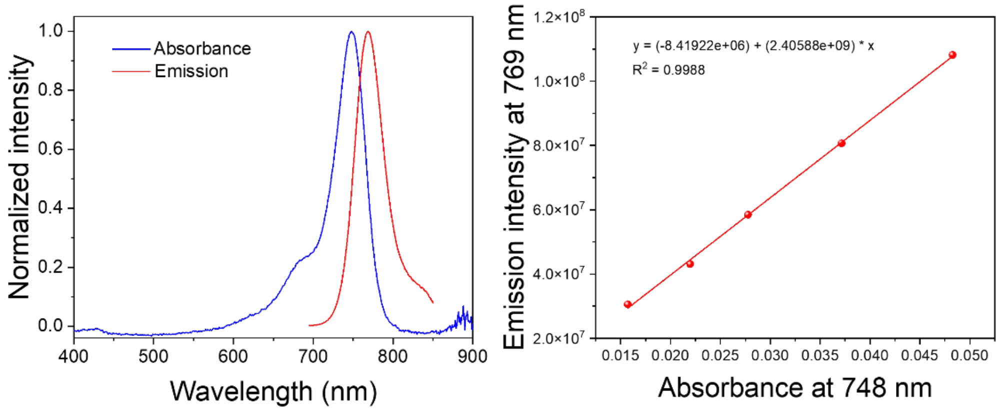


**Figure S32.** (left) UV-Vis absorption and emission spectra of **4af** in methanol. (right) Linear plot of integrated emission intensity at 769 nm versus absorbance at 748 nm for **4t**.

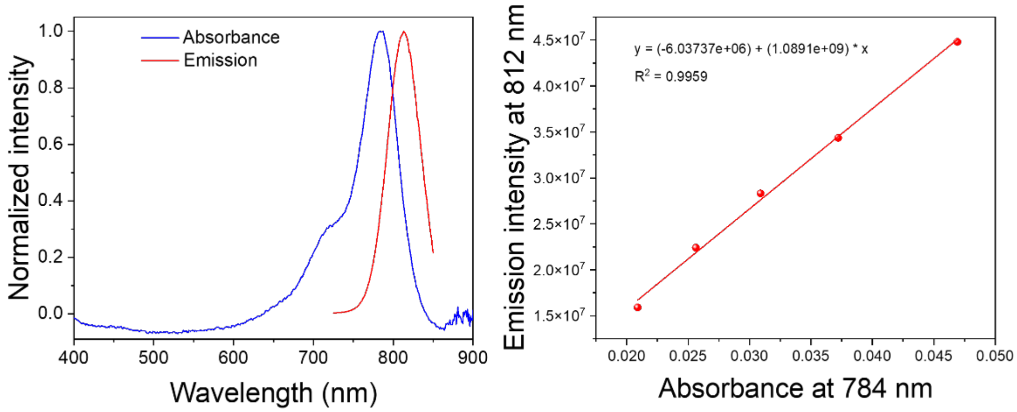

**Figure S33.** (left) UV-Vis absorption and emission spectra of **ICG** in methanol. (right) Linear plot of integrated emission intensity at 812 nm versus absorbance at 784 nm for **ICG**.

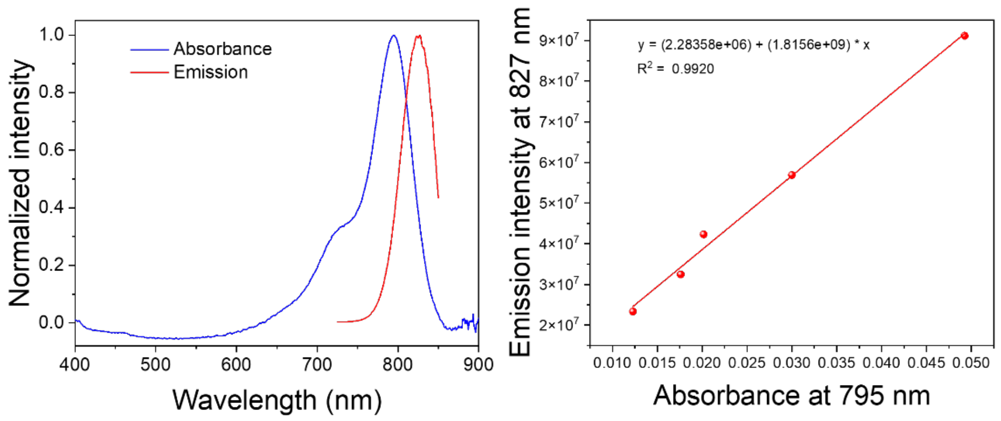


**Figure S34.** (left) UV-Vis absorption and emission spectra of **ICG** in DMSO. (right) Linear plot of integrated emission intensity at 827 nm versus absorbance at 795 nm for **ICG**.

## 4. Flow cytometry

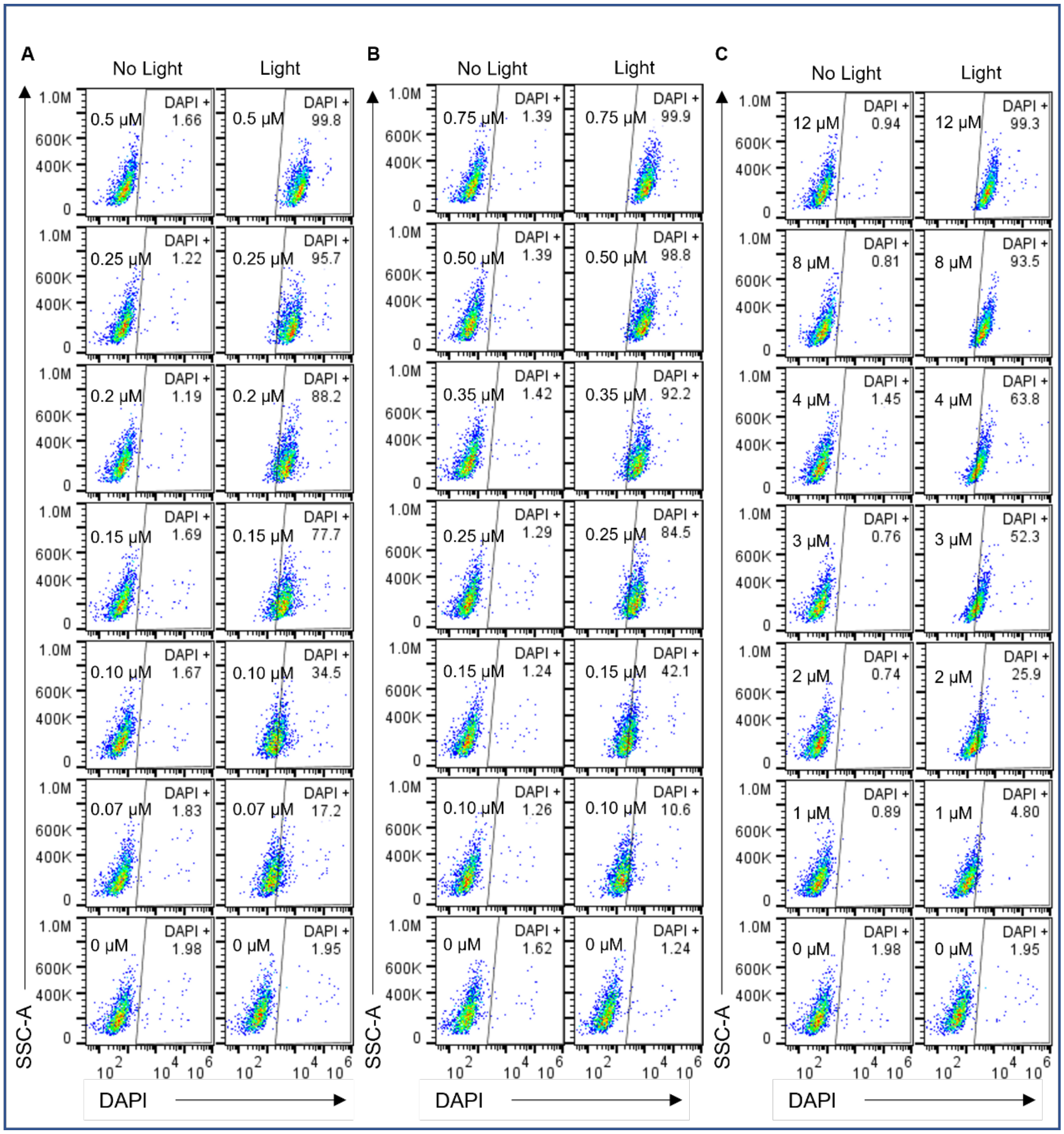


**Figure S35. Flow cytometry analysis of cell membrane permeabilization in KPC cells.** DAPI fluorescence intensity (DAPI) versus side scattering area (SSC-A). (A) Molecule **4q**. (B) Molecule **4h**. (C) Molecule **4ac**. The insets indicate the concentration of cyanine in each treatment and the percentage of DAPI positive cells (membrane permeabilized cells). In each panel, plots on the left column are without light treatment and plots on the right column are with light treatment. Light treatment consisted of 730 nm LED light at 80 mW/cm$^2$ during 10 min. 10,000 cells are analyzed in each plot.

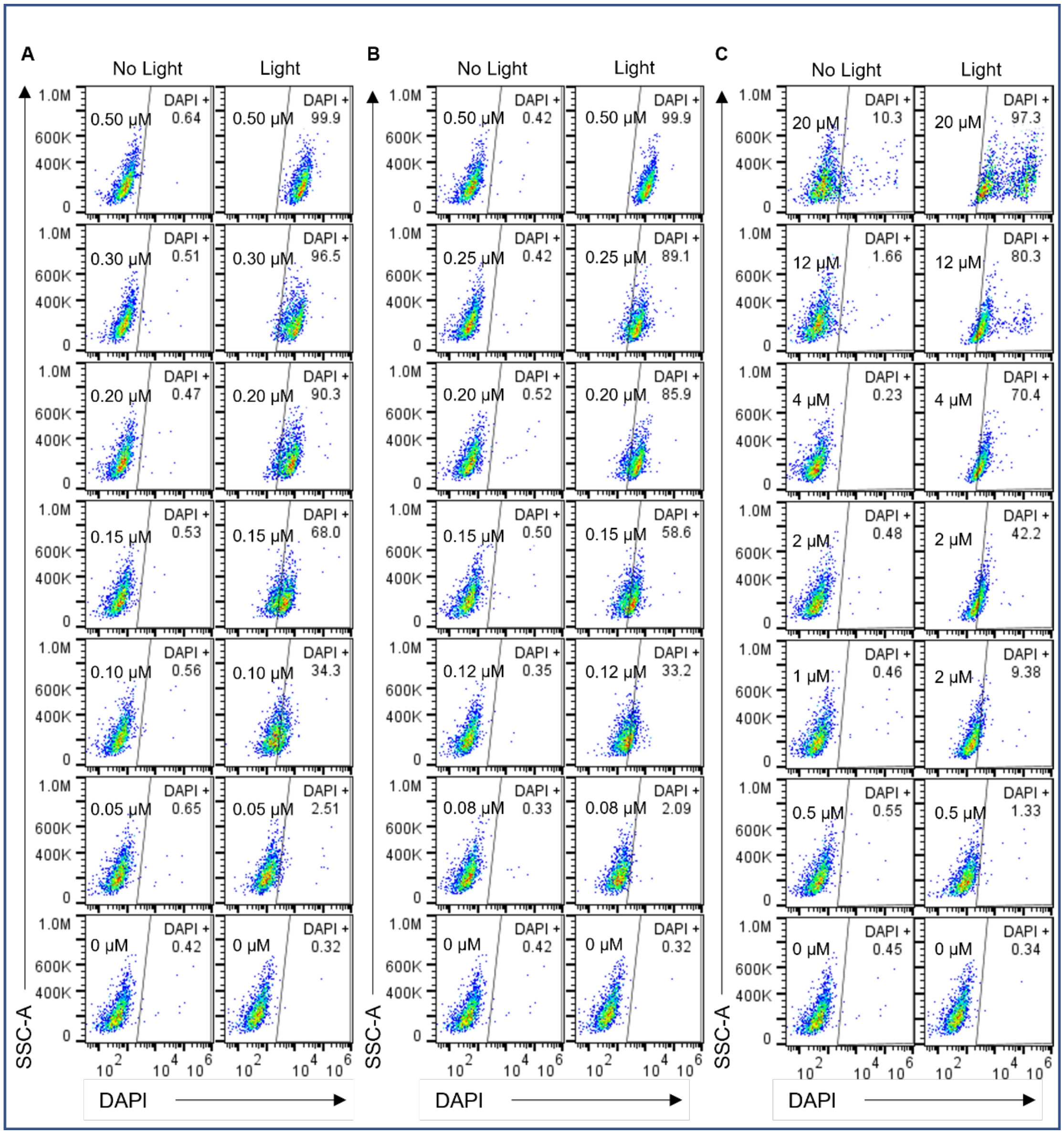


**Figure S36. Flow cytometry analysis of cell membrane permeabilization in KPC cells**. DAPI fluorescence intensity (DAPI) versus side scattering area (SSC-A). (A) Molecule **4u**. (B) Molecule **4r**. (C) Molecule **4ae**. The insets indicate the concentration of cyanine in each treatment and the percentage of DAPI positive cells (membrane permeabilized cells). In each panel, plots on the left column are without light treatment and plots on the right column are with light treatment. Light treatment consisted of 730 nm LED light at 80 mW/cm$^2$ during 10 min. 10,000 cells are analyzed in each plot.

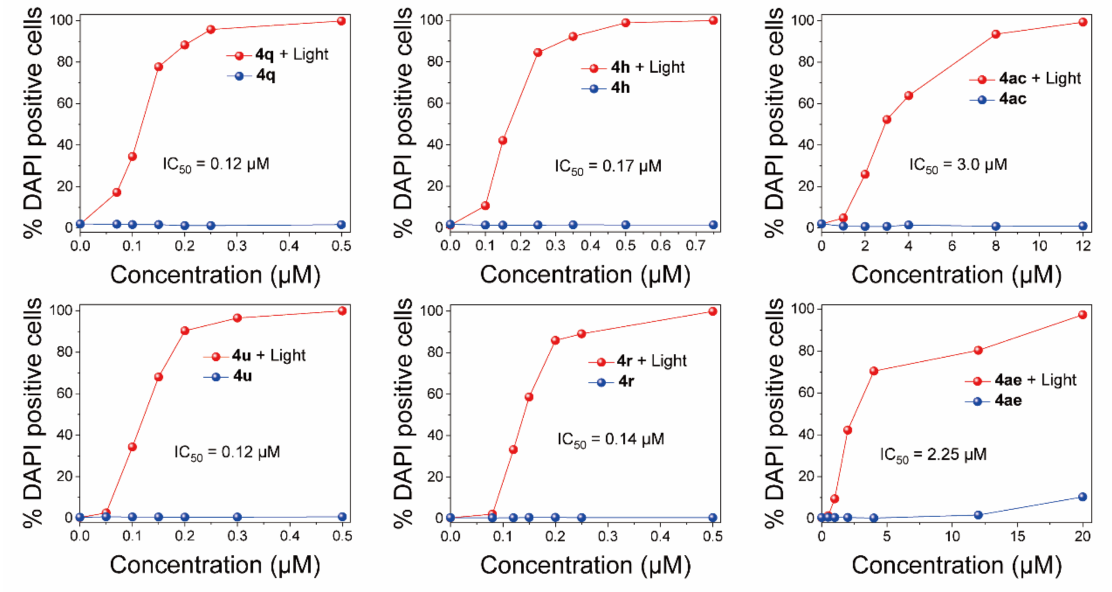


**Figure S37. Effect of the concentration of MJH for permeabilizing KPC cells with or without light activation.** Each data point is obtained from the flow cytometry plots in Figure S35-S36. The IC50 value in the inset corresponds to the effective concentration needed to permeabilize cells by 50% with light activated molecules (this is the VDA $IC_{50}$). The light treatment consisted of 730 nm LED light at 80 mW/cm$^2$ for 10 min.

## 5. Measurements of ROS levels and temperature of the media

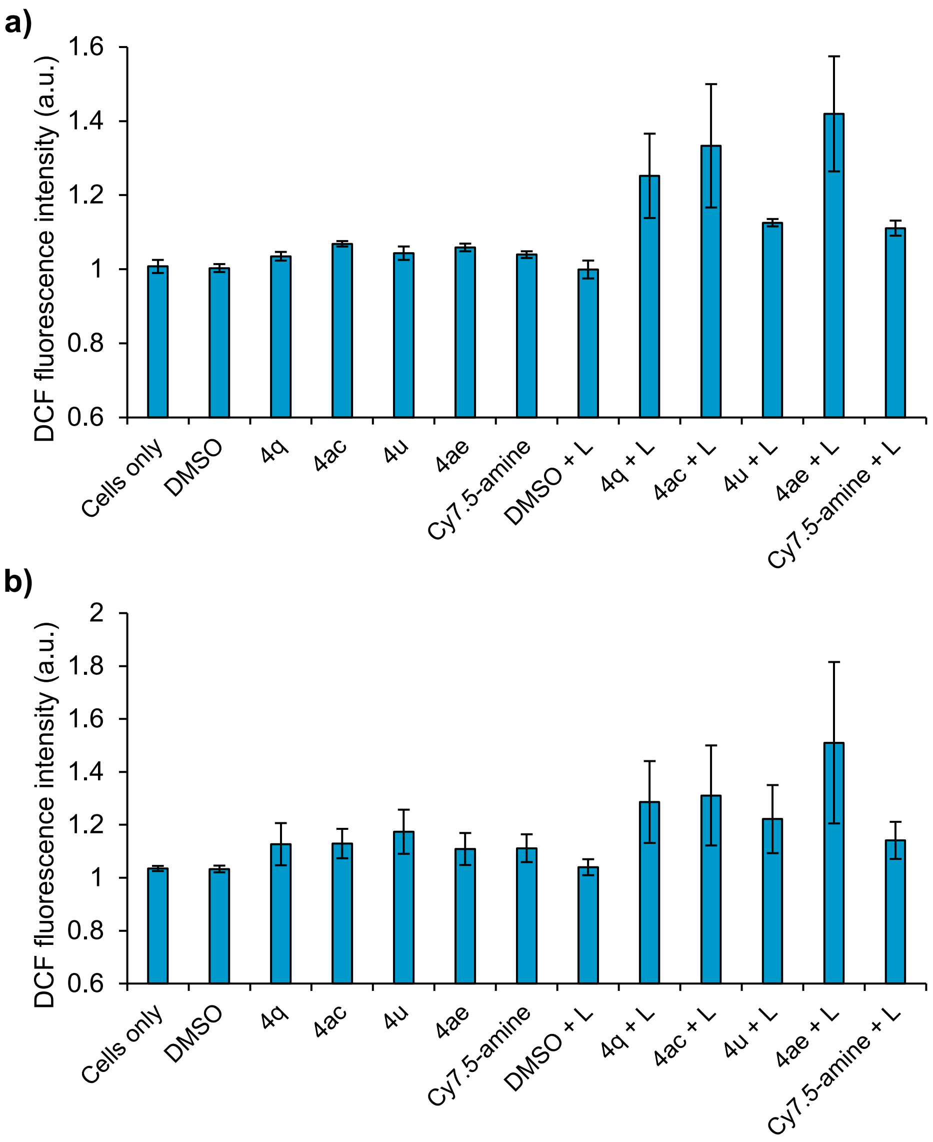


**Figure S38. Quantification of ROS levels while cells were treated with 730 nm LED light at 80 mW/cm$^2$ for 10 min and MJH.** (a) Measurement of ROS levels in A549 cells using 2',7'-dichlorodihydrofluorescein diacetate (DCF-DA) as the ROS probe in the presence of various MJH molecules at 0.5 μM concentration with and without light (L). (b) Measurement of ROS levels in KPC cells using DCF-DA as the ROS probe in the presence of various MJH molecules at 0.5 μM concentration with and without light (L). DMSO control contains 0.1% DMSO in the media because DMSO is used to pre-solubilize the MJH stock solution at 0.5 mM and diluted to 1:1000 to obtain 0.5 μM MJH in media containing 0.1% DMSO. Data are presented as mean values +/-

SD (n = 4). The molecules **4ae** and **4ac** produced approximately slightly higher levels of ROS yet **4ae** and **4ac** are much weaker MJH in cell permeabilization (Figure S37). There is no correlation between ROS levels and cell permeabilization.

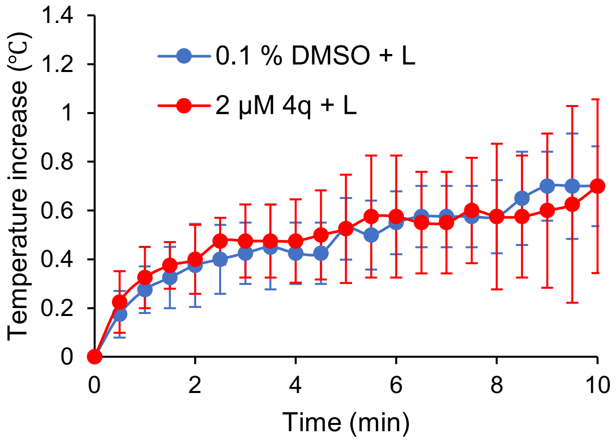


**Figure S39. The temperature of the media while cells were treated with 730 nm LED light and MJH. KPC cells were treated with molecule 4q or DMSO and 730 nm LED light (L) at 80 mW/cm$^2$ for 10 min.** The temperature increase is relative to the initial temperature of the media. The initial temperature of the media was ~20 °C (room temperature). DMSO control contains 0.1% DMSO in the media because DMSO is used to pre-solubilize the **4q** stock solution at 2 mM and diluted to 1:1000 to obtain 2 µM **4q** in media containing 0.1% DMSO. Data are presented as mean values +/- SD (n = 4).

**6. The chemical and biological stability of MJH**

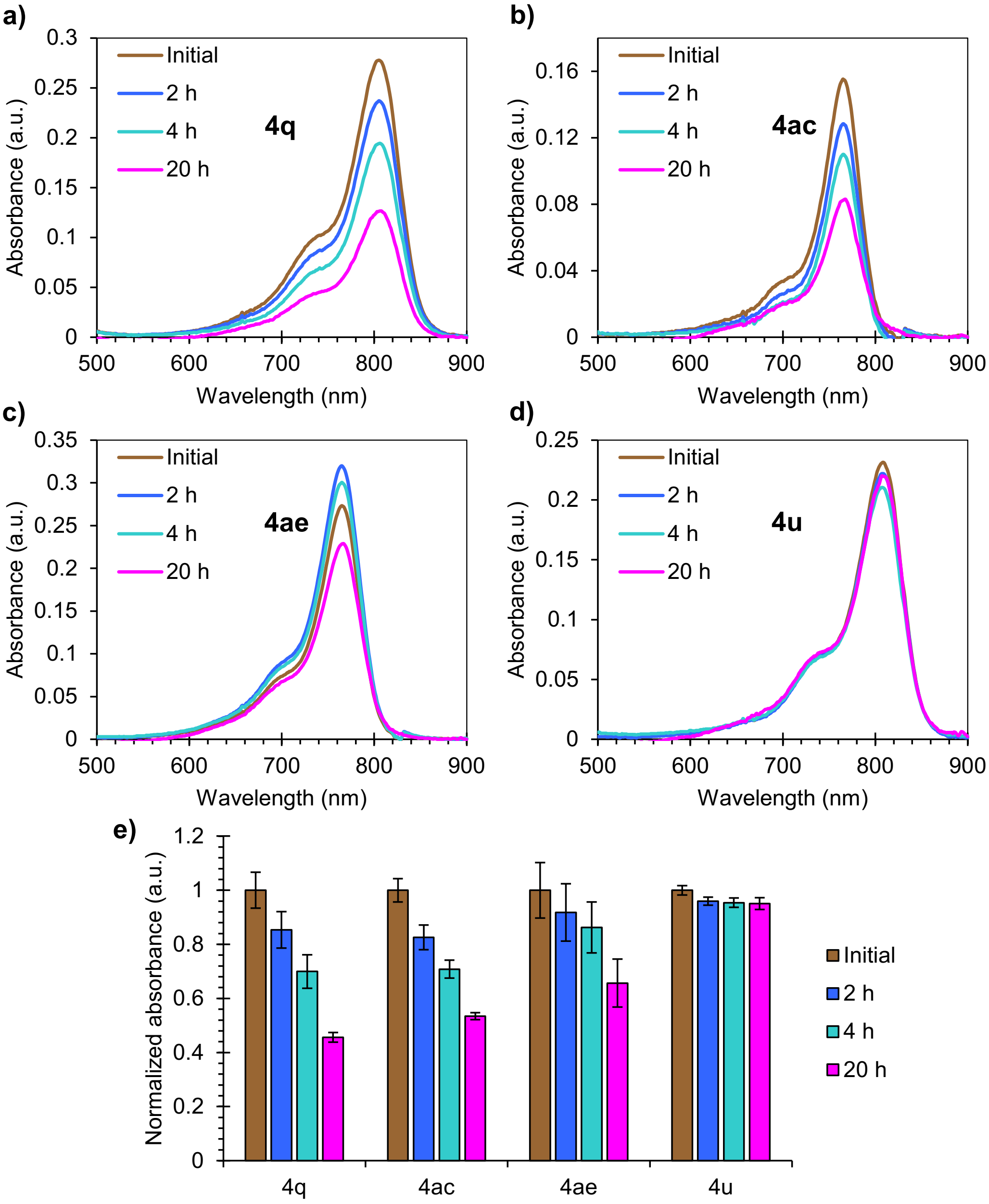


**Figure S40. Stability of Cy7 (4ac and 4ae) and Cy7.5 (4q and 4u) MJH in RPMI media over time.** UV-vis absorption spectrum of (a) molecule **4q**, (b) molecule **4ac**, (c) molecule **4ae**, (d) molecule **4u** in RPMI media supplemented with 10% Fetal Bovine Serum (FBS). (e) Summary of the absorption intensity at the $\lambda_{max}$ over time for each of molecule. The absorption intensities are normalized to the initial intensity. Data are presented as mean values +/- SD (n = 3).

**7. Cellular clearance of MJH from normal and cancer cells**

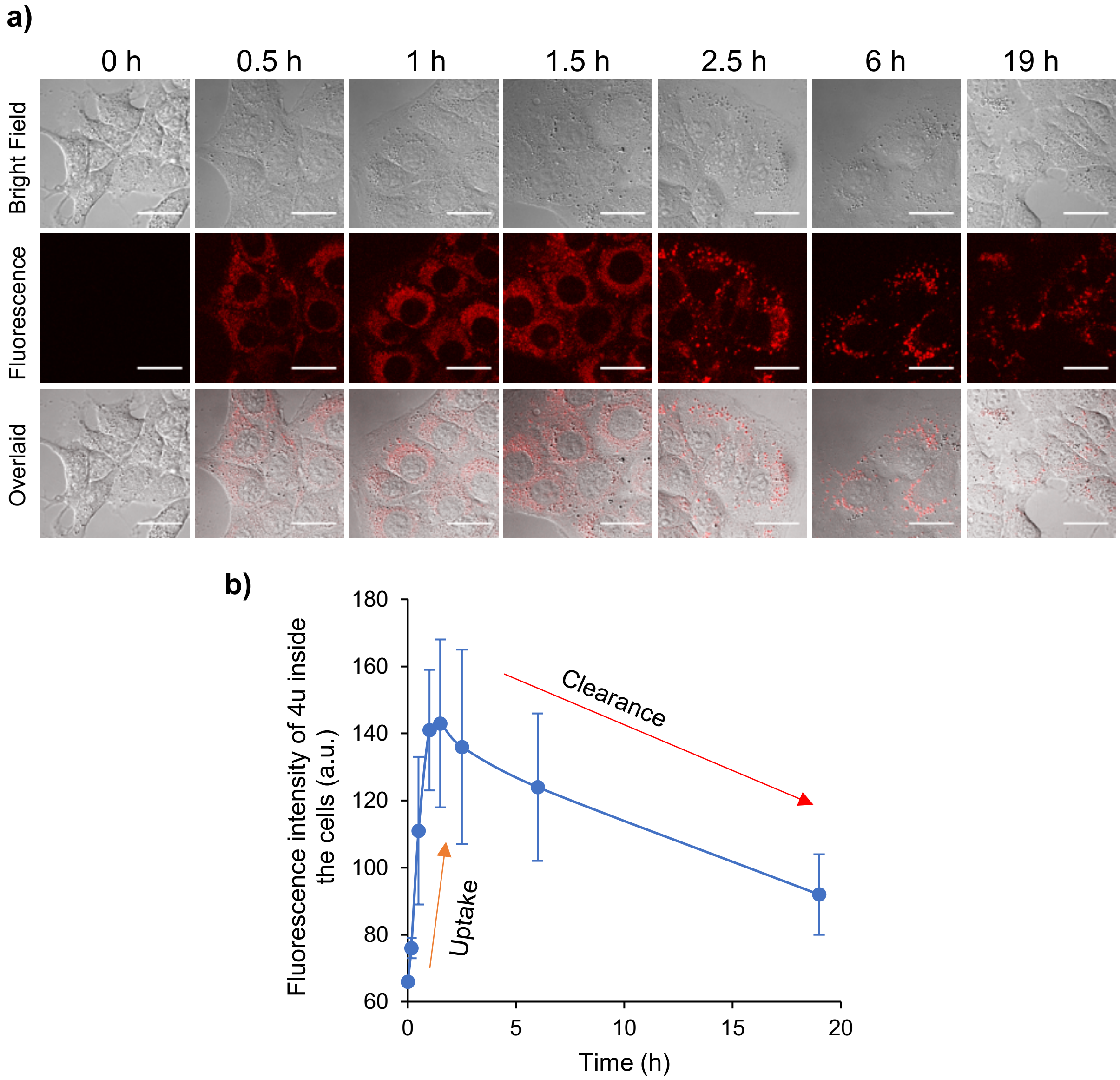


**Figure S41. Cellular uptake and clearance of molecule 4u in KPC cells over time.** (a) fluorescence confocal microscopy imaging of **4u** in KPC cells. Imaging conditions: molecule **4u**, loading concentration $C_{\text{loading}} = 0.5$ µM, $\lambda_{ex} = 640$ nm, $\lambda_{em} = 663\text{-}738$ nm. The red color represents the fluorescence emission of molecule **4u**. (b) Quantification of the fluorescence intensity of molecule **4u** in the cytoplasm. Incubation conditions: 30,000 KPC cells were seeded in a glass bottom dish (IBIDI, µ-dish 35 mm high glass bottom) with RPMI media supplemented with 10% FBS and penicillin/streptomycin and were culture for 2 d at 37 °C and 5% $CO_2$. Then, molecule

**4u** was added to the cellular culture and continued the incubation conditions. The cells were removed from incubation for a period of ~10 min to record the confocal microscopy images at each time point. The cells were washed 3 times with fresh media after recording the data point at 1.5 h and washed 1 time after recording the data point at 6 h. At 2.5 h and after, molecule **4u** is observed in vesicles which likely are exocytosis vesicles. Data are presented as mean values +/- SD (n = 20). Scale bars = 25 μm.

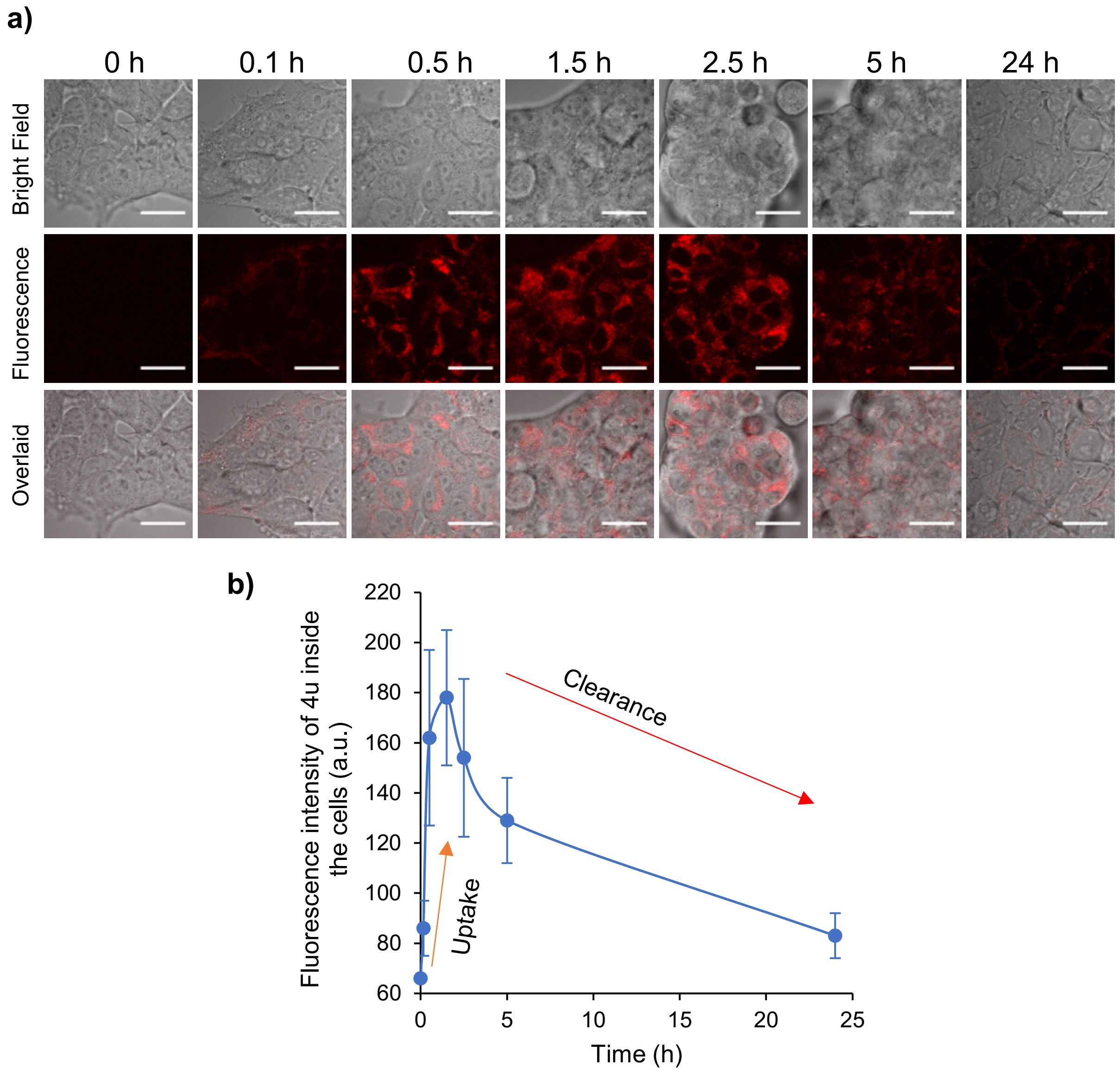


**Figure S42. Cellular uptake and clearance of molecule 4u in HEK293T cells over time.** (a)

fluorescence confocal microscopy imaging of **4u** in HEK293T cells. Imaging conditions: molecule **4u**, loading concentration $C_{loading}$ = 0.5 µM, $\lambda_{ex}$ = 640 nm, $\lambda_{em}$ = 663-738 nm. The red color represents the fluorescence emission of molecule **4u**. (b) Quantification of the fluorescence intensity of molecule **4u** in the cytoplasm. Incubation conditions: 200,000 HEK293T cells were seeded in a glass bottom dish (IBIDI, µ-dish 35 mm high glass bottom) with DMEM media supplemented with 10% FBS and penicillin/streptomycin and were culture for 2 d at 37 °C and 5% $CO_2$. Then, molecule **4u** was added to the cellular culture and continued the incubation conditions. The cells were removed from incubation for a period of ~10 min to record the confocal microscopy images at each time point. The cells were washed 3 times with fresh media after recording the data point at 1.5 h and washed 1 time after recording the data point at 5 h. Data are presented as mean values +/- SD (n = 20). Scale bars = 25 µm.

**8. Clonogenic assay**

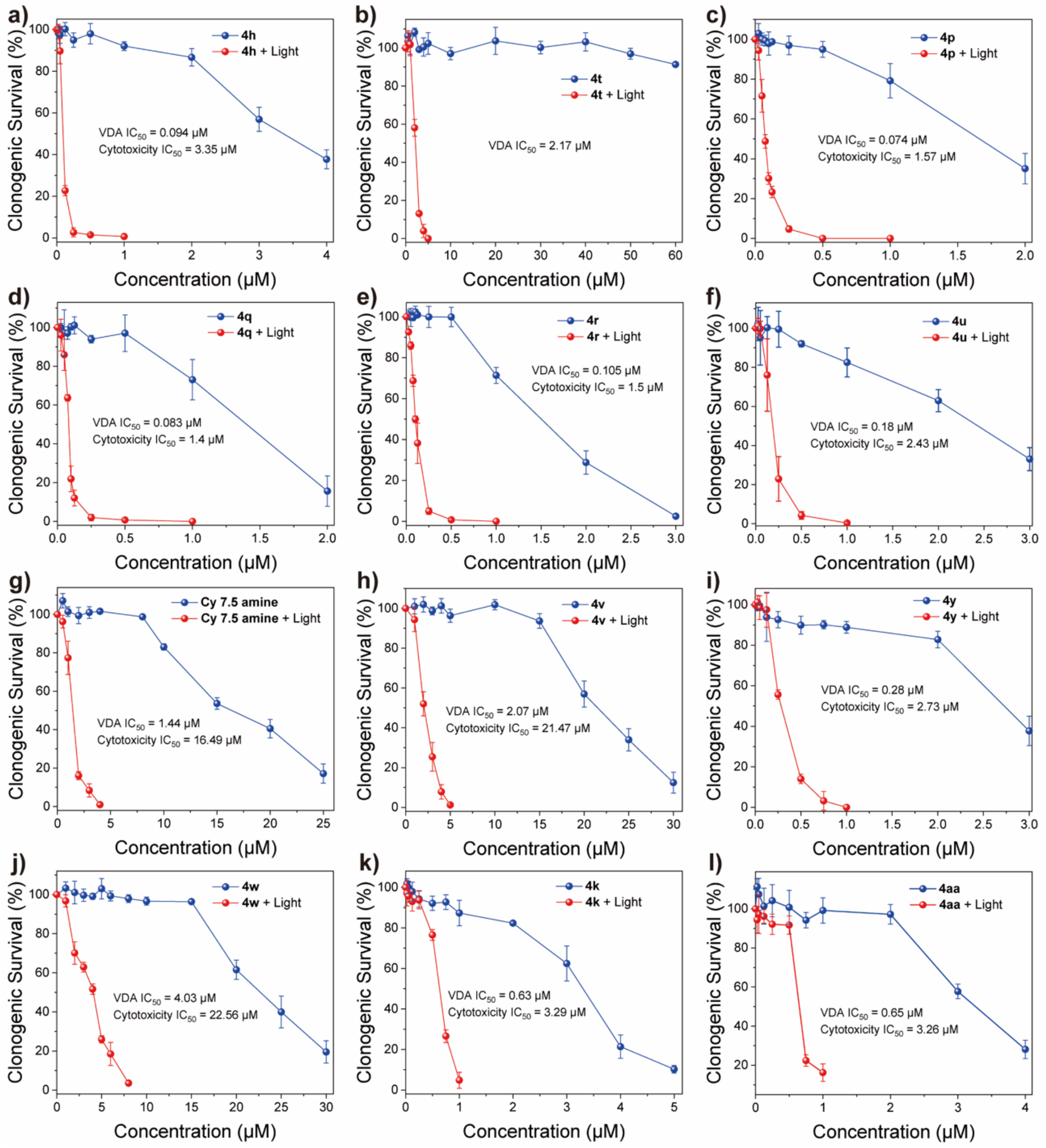


**Figure S43. Clonogenic assay to measure VDA activity (with light) and toxicity (without light) with KPC cells under the same experiment.** Both properties were measured using clonogenic assay with 40 min of incubation (contact time between molecules and KPC cells) and 7 d for colony formation. The light treatment consisted of 730 nm LED light at 80 mW/cm$^2$ for 10 min. In average, 300 cells were seeded and analyzed for each data point.

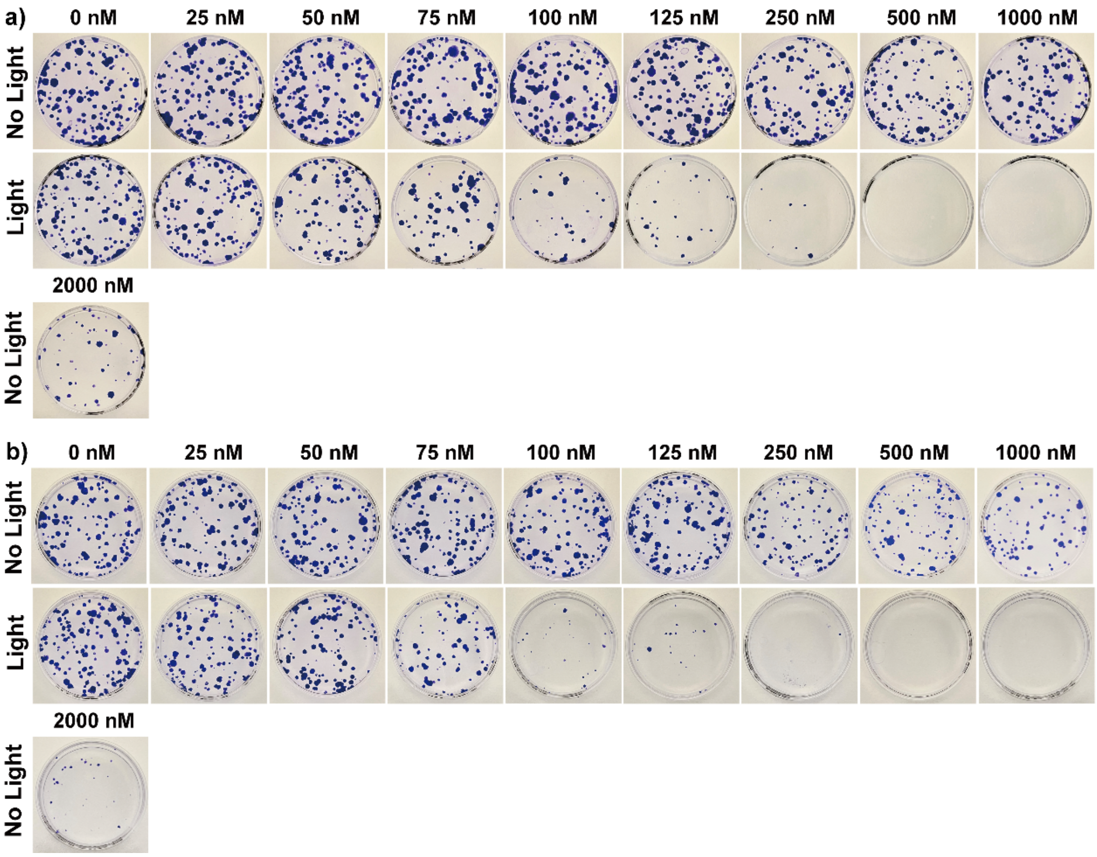


**Figure S44. Picture of clonogenic assay for the VDA activity (with light) and toxicity (without light) with KPC cells.** Both properties were measured using clonogenic assay with 40 min of incubation (contact time between molecules and KPC cells) and 7 d for colony formation. The light treatment consisted of 730 nm LED light at 80 mW/cm$^2$ for 10 min. (a) Molecule **4p** 0-2 µM. (b) Molecule **4q** at 0-2 µM. In average, 300 cells were seeded and analyzed in each plate.

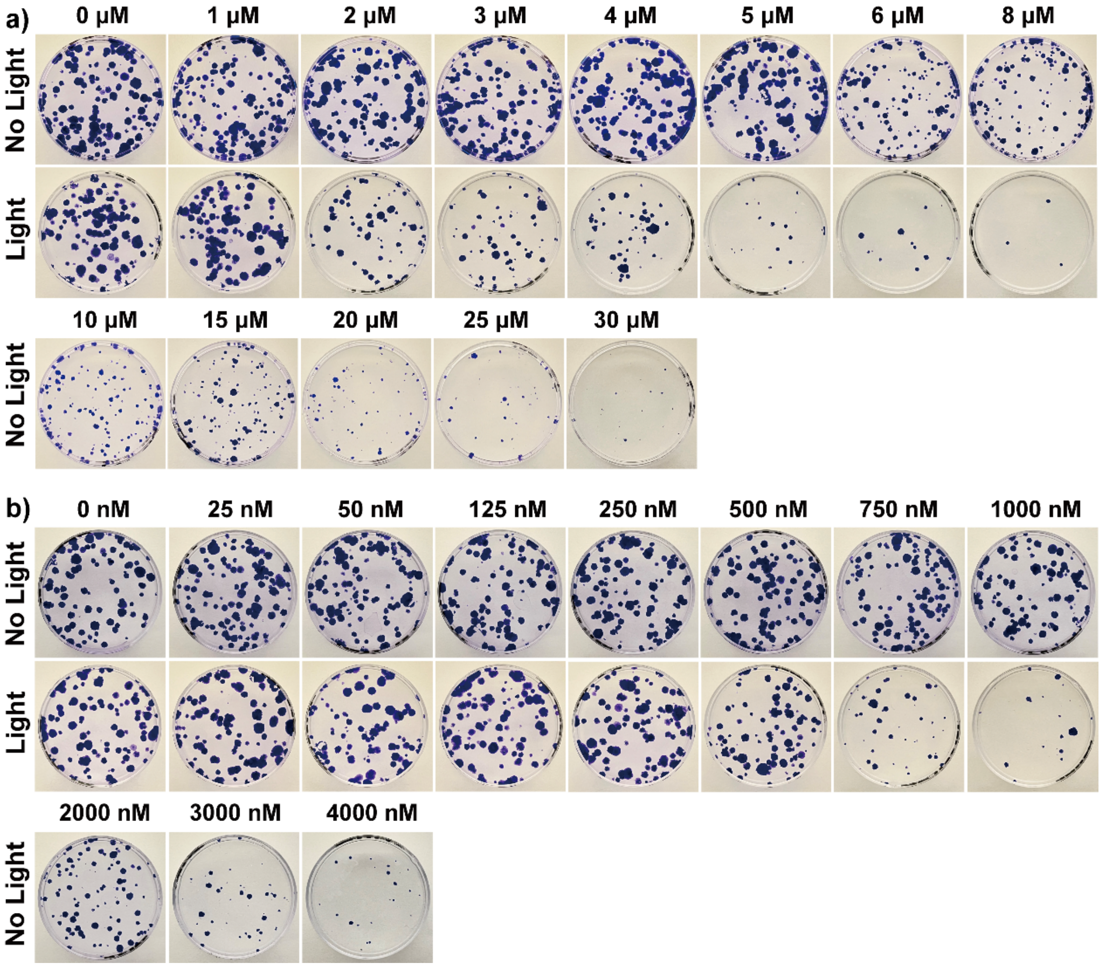


**Figure S45. Picture of clonogenic assay for the VDA activity (with light) and toxicity (without light) with KPC cells.** Both properties were measured using clonogenic assay with 40 min of incubation (contact time between molecules and KPC cells) and 7 d for colony formation. The light treatment consisted of 730 nm LED light at 80 mW/cm$^2$ for 10 min. (a) Molecule **4w** at 0-30 µM. (b) Molecule **4aa** at 0-4 µM. In average, 300 cells were seeded and analyzed in each plate.

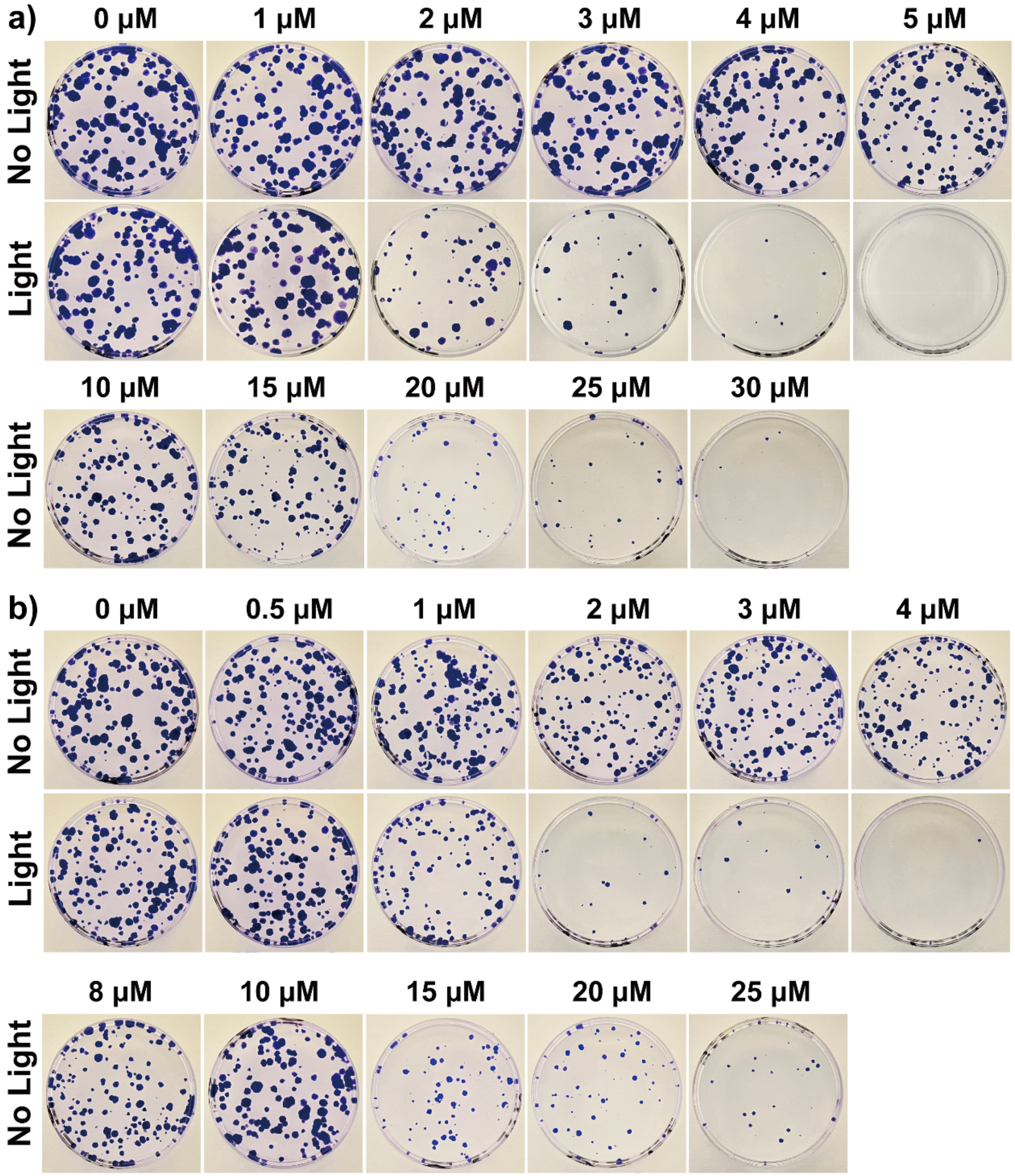


**Figure S46. Picture of clonogenic assay for the VDA activity (with light) and toxicity (without light) with KPC cells.** Both properties were measured using clonogenic assay with 40 min of incubation (contact time between molecules and KPC cells) and 7 d for colony formation. The light treatment consisted of 730 nm LED light at 80 mW/cm$^2$ for 10 min. (a) Molecule **4v** 0-30 μM. (b) Cy7.5 amine at 0-25 μM. In average, 300 cells were seeded and analyzed in each plate.

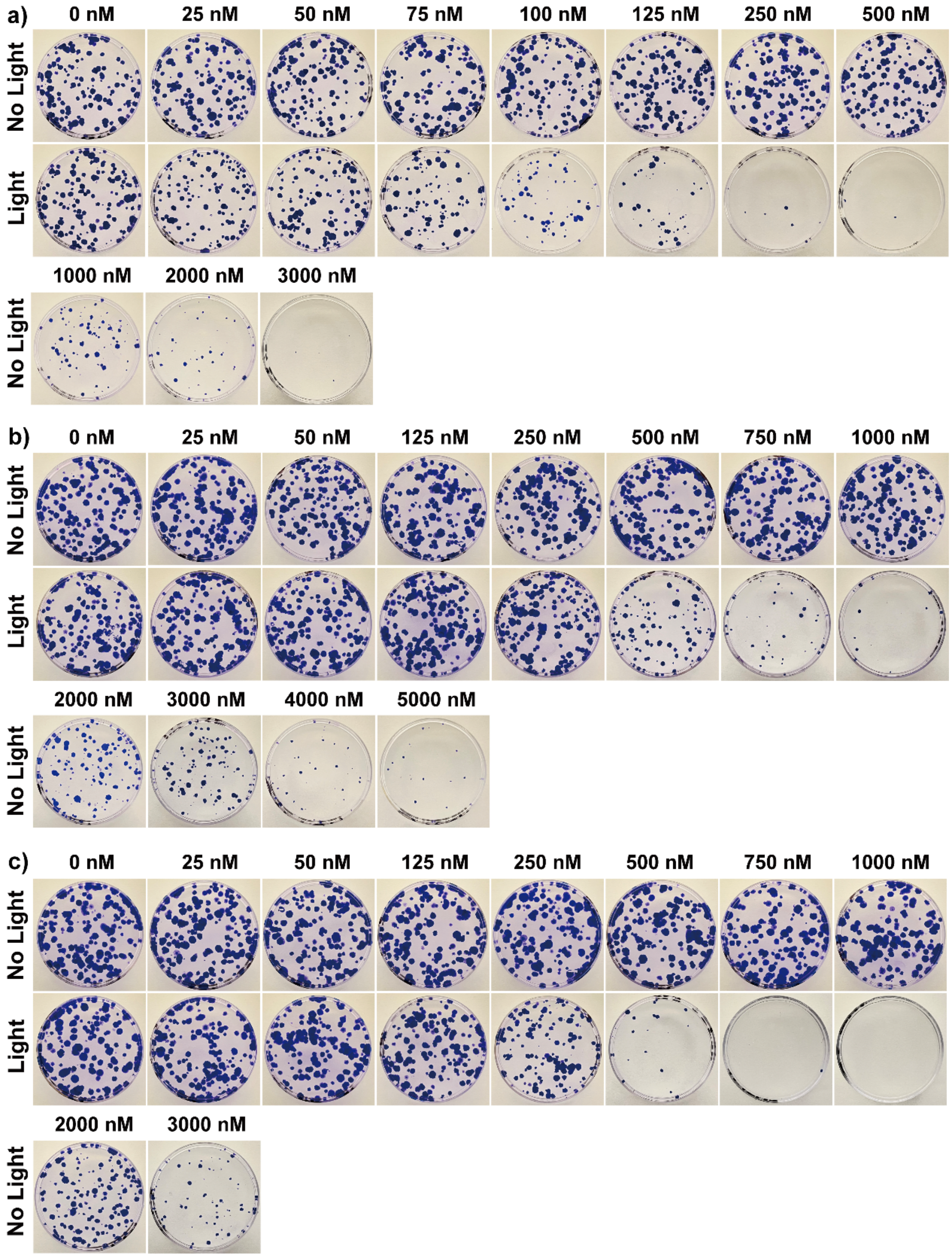


**Figure S47. Picture of clonogenic assay for the VDA activity (with light) and toxicity (without light) with KPC cells.** Both properties were measured using clonogenic assay with 40 min of incubation (contact time between molecules and KPC cells) and 7 d for colony formation. The

light treatment consisted of 730 nm LED light at 80 mW/cm$^2$ for 10 min. (a) Molecule **4r** 0-3 μM. (b) Molecule **4k** at 0-5 μM. (c) Molecule **4y** at 0-3 μM. In average, 300 cells were seeded and analyzed in each plate.

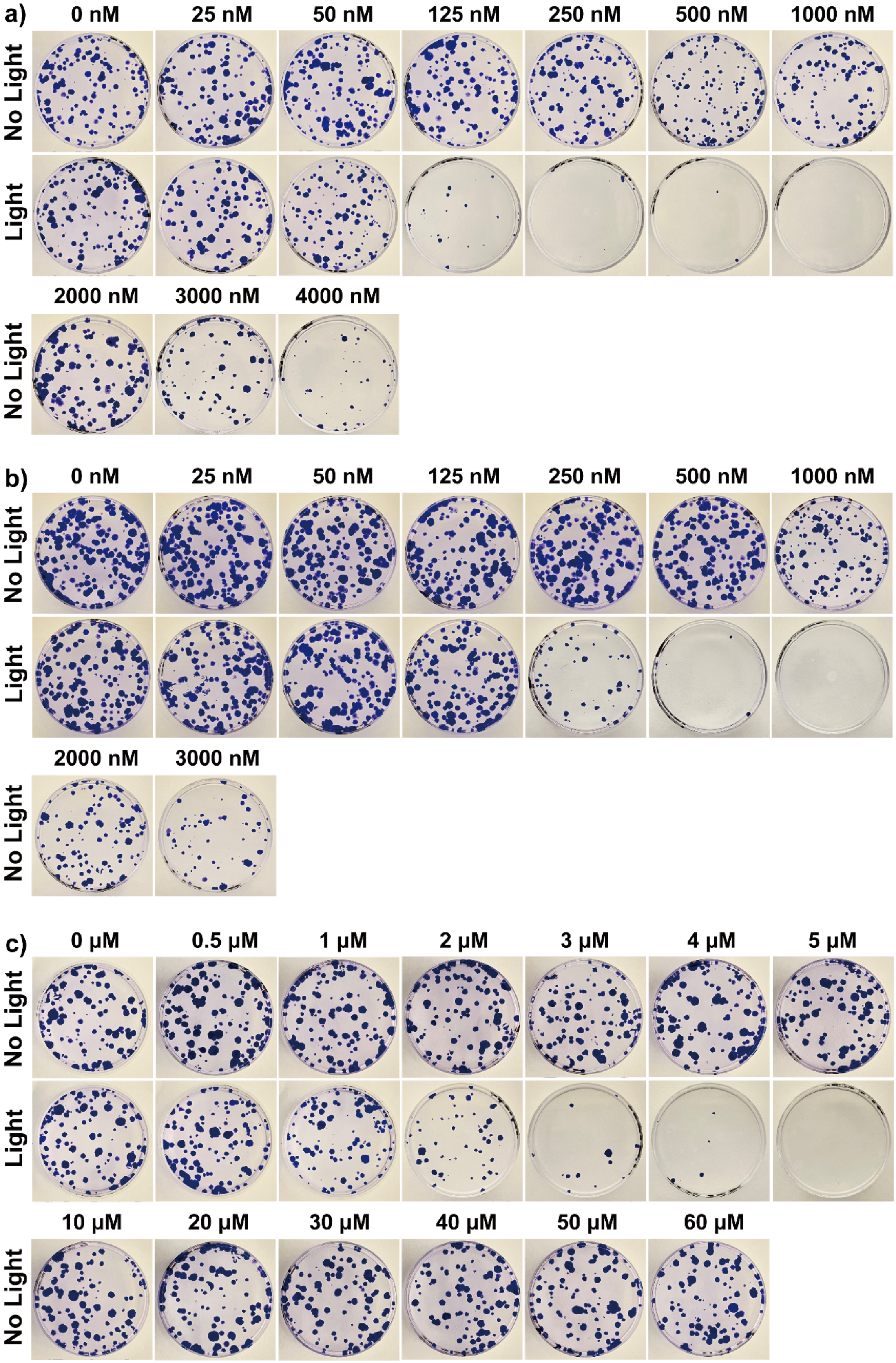
a)
0 nM
25 nM
50 nM
125 nM
250 nM
500 nM
1000 nM
No Light
Light
2000 nM
3000 nM
4000 nM
No Light
b)
0 nM
25 nM
50 nM
125 nM
250 nM
500 nM
1000 nM
No Light
Light
2000 nM
3000 nM
No Light
c)
0 μM
0.5 μM
1 μM
2 μM
3 μM
4 μM
5 μM
No Light
Light
10 μM
20 μM
30 μM
40 μM
50 μM
60 μM
No Light

**Figure S48. Picture of clonogenic assay for the VDA activity (with light) and toxicity (without light) with KPC cells.** Both properties were measured using clonogenic assay with 40 min of incubation (contact time between molecules and KPC cells) and 7 d for colony formation. The light treatment consisted of 730 nm LED light at 80 mW/cm$^2$ for 10 min. (a) Molecule **4h** 0-4 µM. (b) Molecule **4u** at 0-3 µM. (c) Molecule **4t** at 0-60 µM. In average, 300 cells were seeded and analyzed in each plate.

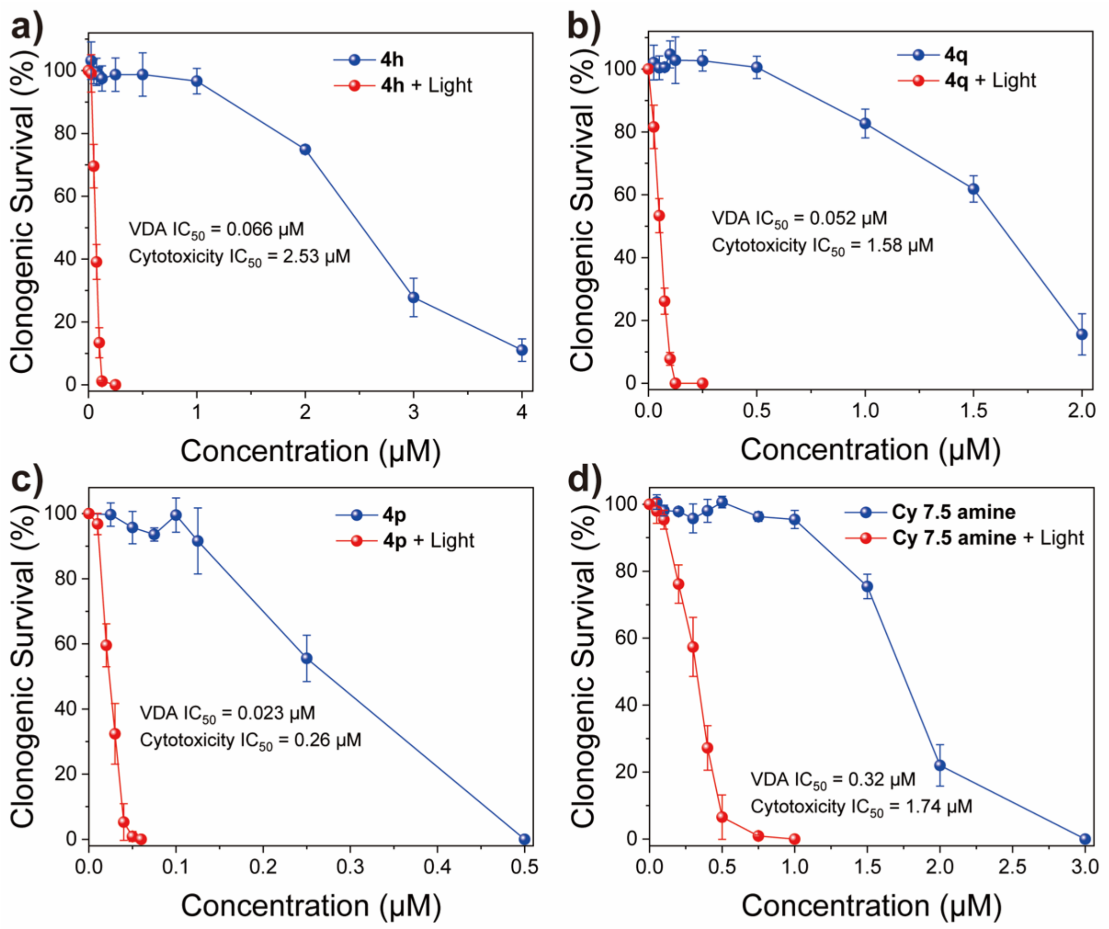


**Figure S49. Clonogenic assay to measure VDA activity (with light) and toxicity (without light) with A549 cells under the same experiment.** Both properties were measured using clonogenic assay with 40 min of incubation (contact time between molecules and A549 cells) and 12 d for colony formation. The light treatment consisted of 730 nm LED light at 80 mW/cm$^2$ for 10 min. In average, 300 cells were seeded and analyzed for each data point.

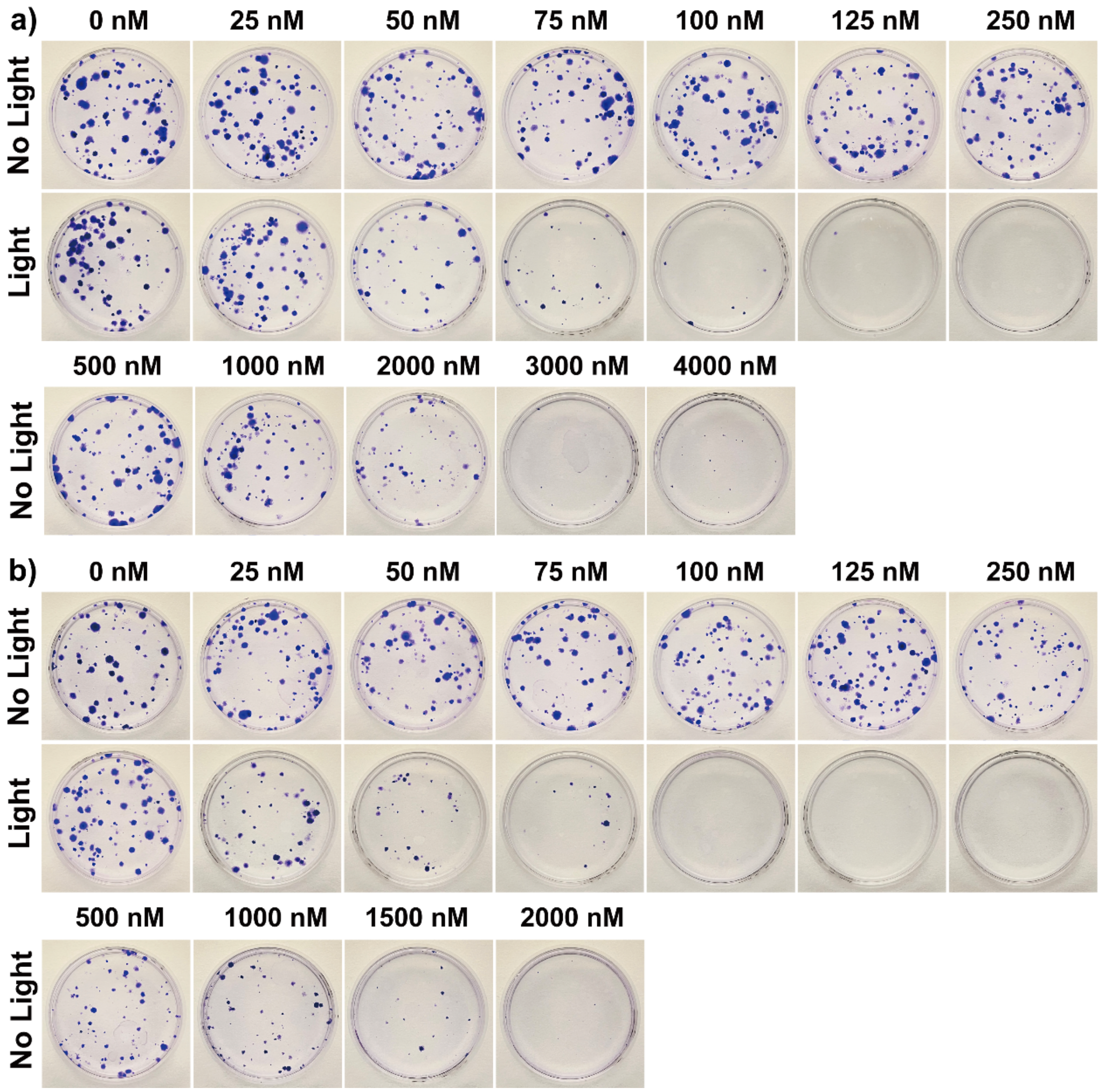


**Figure S50. Picture of clonogenic assay for the VDA activity (with light) and toxicity (without light) with A549 cells.** Both properties were measured using clonogenic assay with 40 min of incubation (contact time between molecules and A549 cells) and 12 d for colony formation. The light treatment consisted of 730 nm LED light at 80 mW/cm$^2$ for 10 min. (a) Molecule **4h** 0-4 µM. (b) Molecule **4q** at 0-2 µM. In average, 300 cells were seeded and analyzed in each plate.

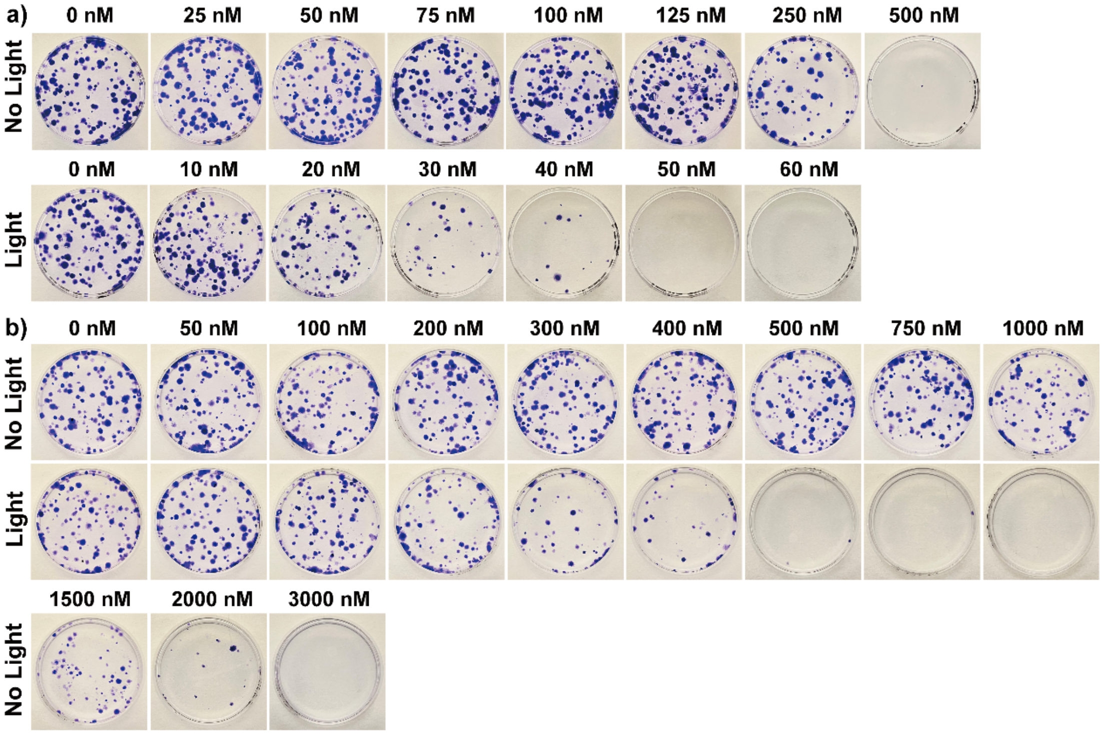


**Figure S51. Picture of clonogenic assay for the VDA activity (with light) and toxicity (without light) with A549 cells.** Both properties were measured using clonogenic assay with 40 min of incubation (contact time between molecules and A549 cells) and 12 d for colony formation. The light treatment consisted of 730 nm LED light at 80 mW/cm$^2$ for 10 min. (a) Molecule **4p** 0-500 nM. (b) Cy7.5 amine at 0-3 µM. In average, 300 cells were seeded and analyzed in each plate.

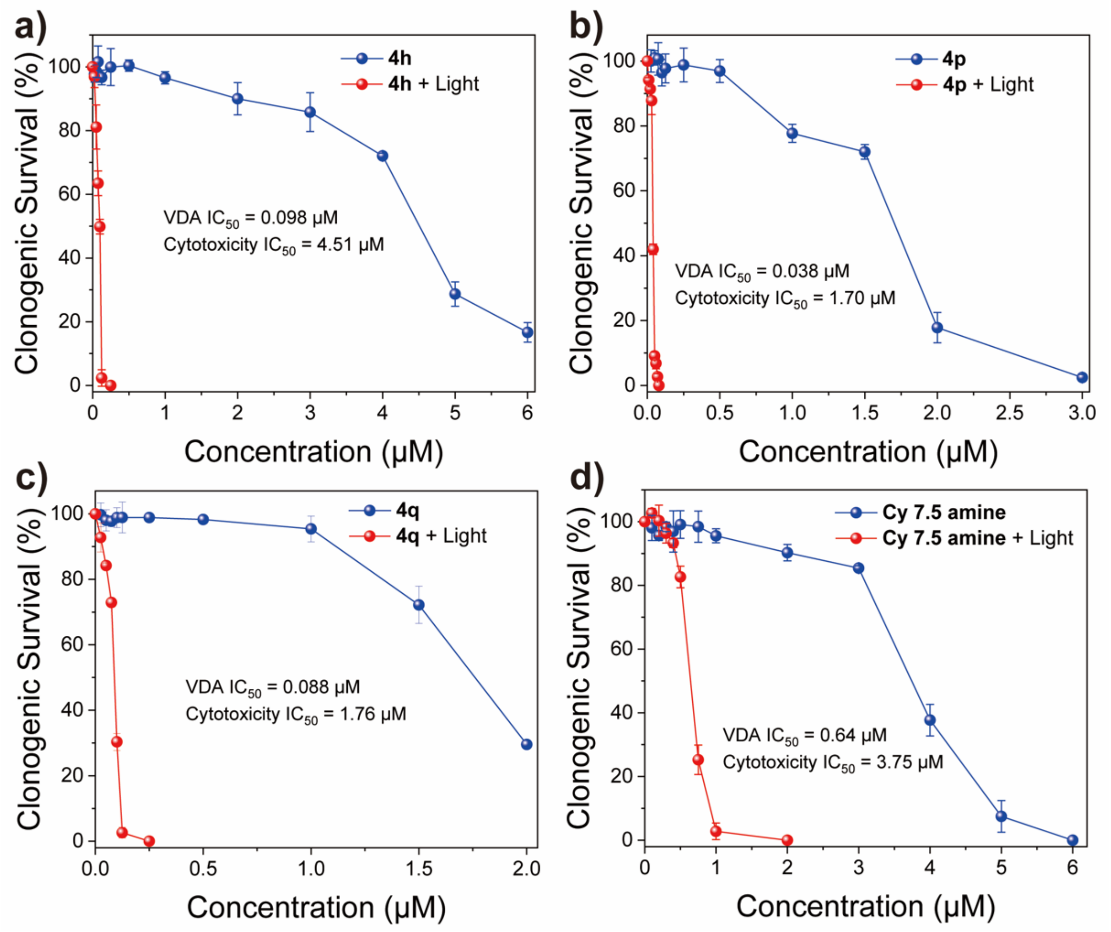


**Figure S52. Clonogenic assay to measure VDA activity (with light) and toxicity (without light) with PC-3 cells under the same experiment.** Both properties were measured using clonogenic assay with 40 min of incubation (contact time between molecules and PC-3 cells) and 12 d for colony formation. The light treatment consisted of 730 nm LED light at 80 mW/cm$^2$ for 10 min. In average, 300 cells were seeded and analyzed for each data point.

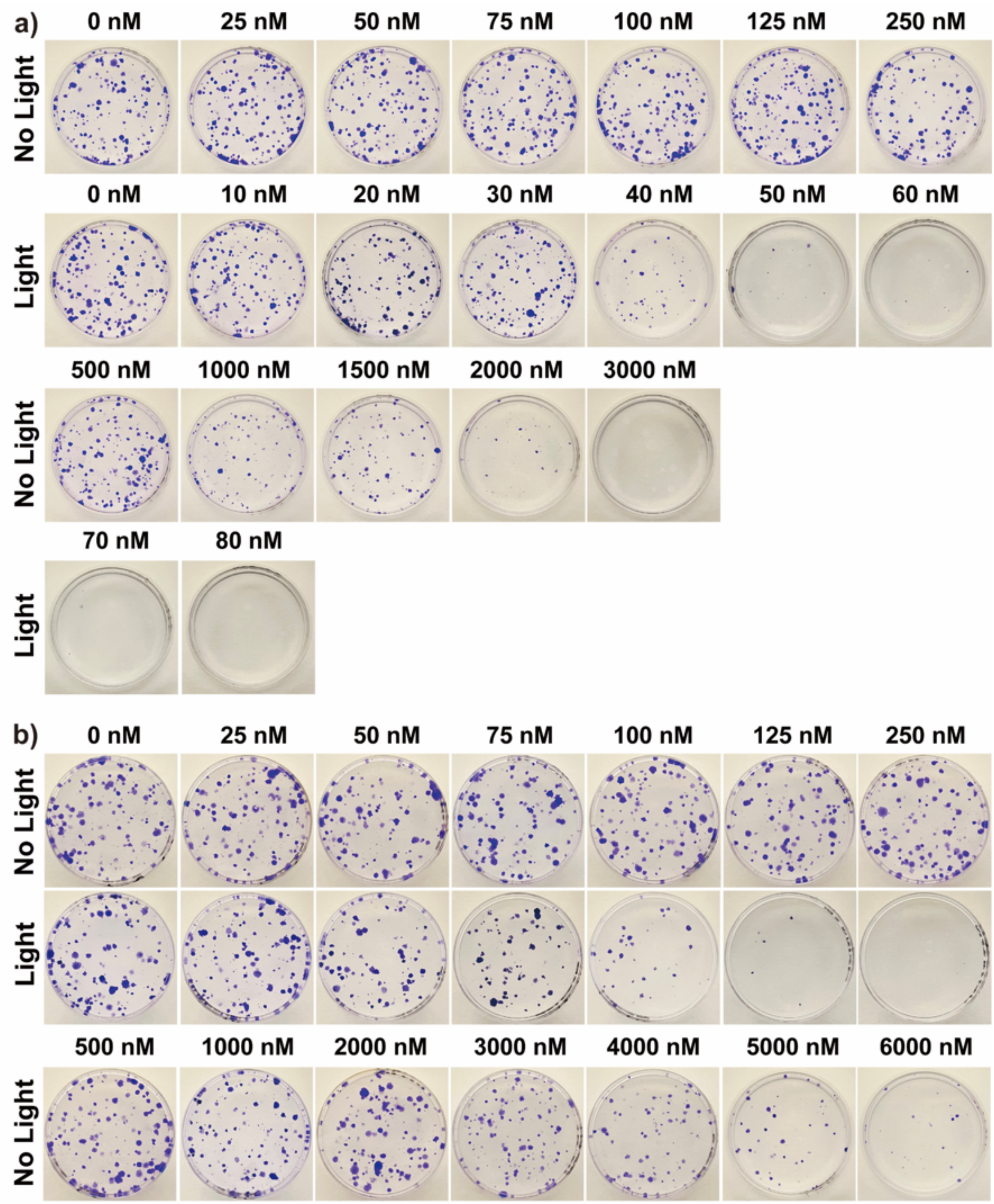


**Figure S53. Picture of clonogenic assay for the VDA activity (with light) and toxicity (without light) with PC-3 cells.** Both properties were measured using clonogenic assay with 40 min of incubation (contact time between molecules and PC-3 cells) and 12 d for colony formation. The light treatment consisted of 730 nm LED light at 80 mW/cm$^2$ for 10 min. (a) Molecule **4p** 0-3 µM. (b) Molecule **4h** at 0-6 µM. In average, 300 cells were seeded and analyzed in each plate.

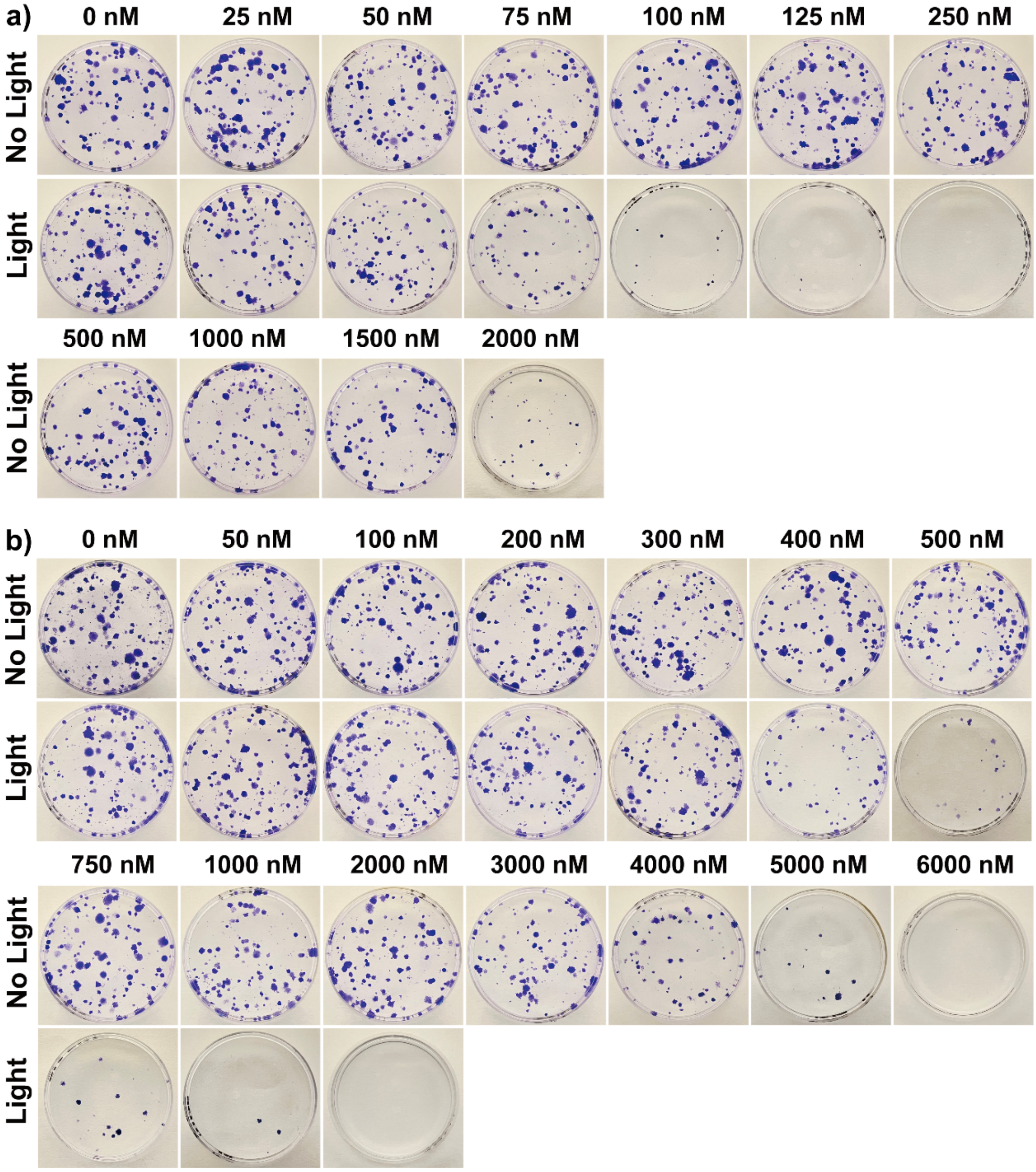


**Figure S54. Picture of clonogenic assay for the VDA activity (with light) and toxicity (without light) with PC-3 cells.** Both properties were measured using clonogenic assay with 40 min of incubation (contact time between molecules and PC-3 cells) and 12 d for colony formation. The light treatment consisted of 730 nm LED light at 80 mW/cm$^2$ for 10 min. (a) Molecule **4q** 0-1 μM. (b) Cy7.5 amine at 0-6 μM. In average, 300 cells were seeded and analyzed in each plate.

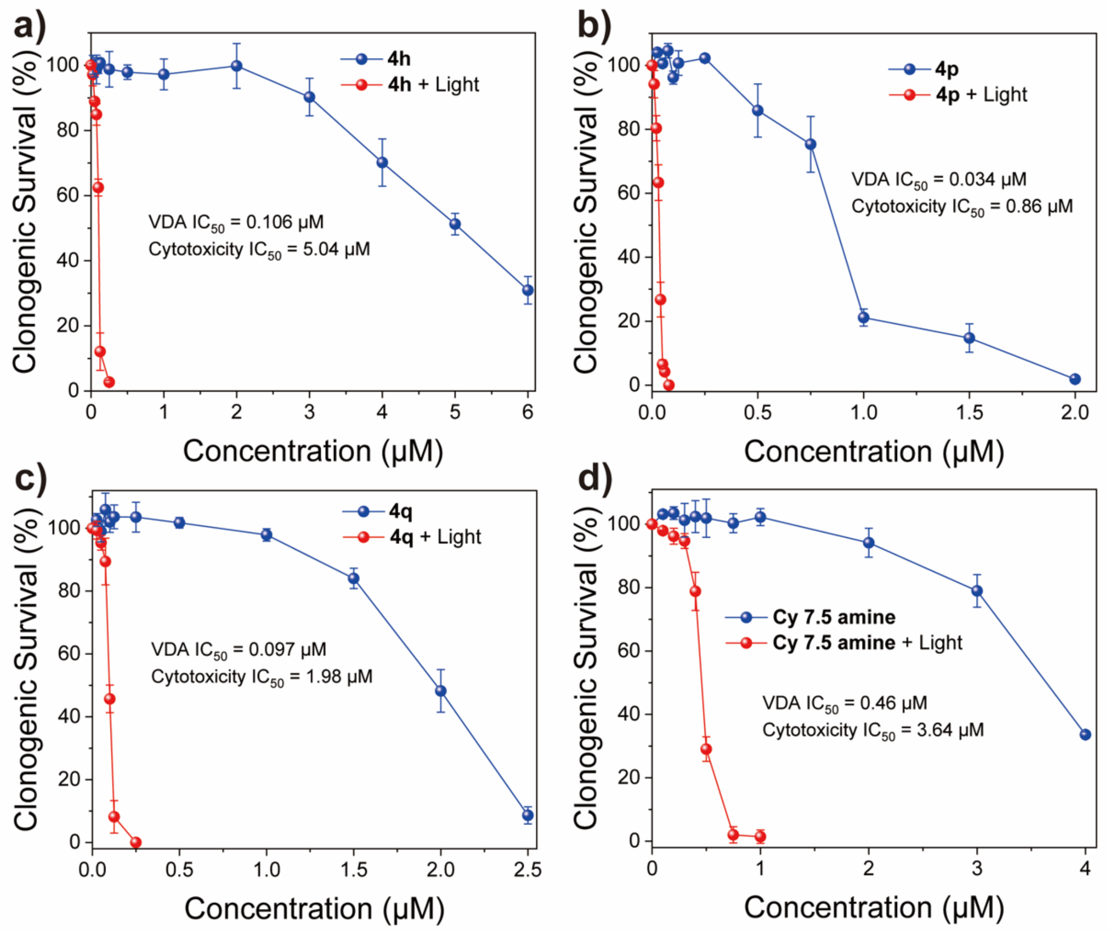


**Figure S55. Clonogenic assay to measure VDA activity (with light) and toxicity (without light) with HCT-116 cells under the same experiment.** Both properties were measured using clonogenic assay with 40 min of incubation (contact time between molecules and HCT-116 cells) and 8 d for colony formation. The light treatment consisted of 730 nm LED light at 80 mW/cm$^2$ for 10 min. In average, 500 cells were seeded and analyzed for each data point.

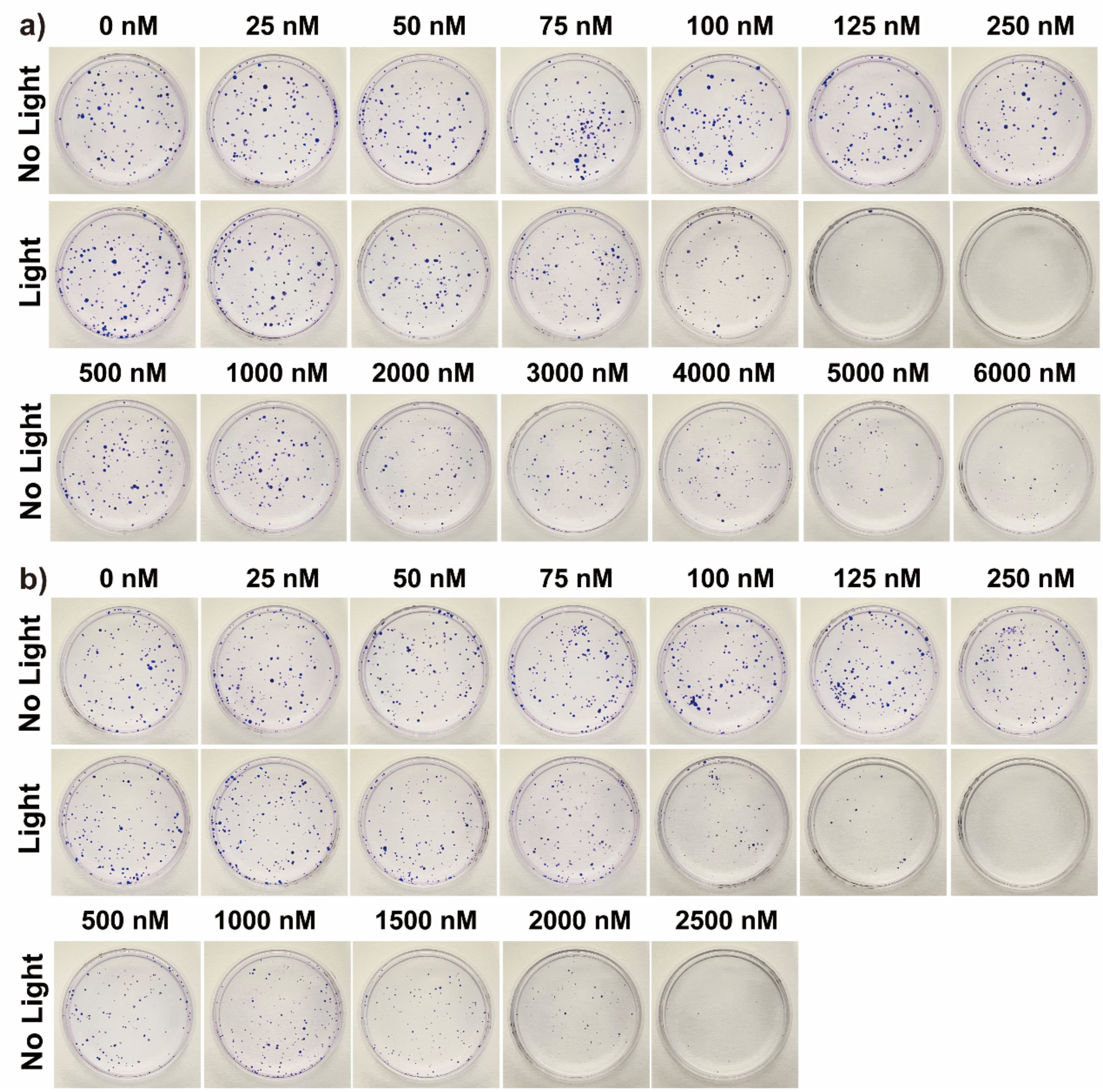


**Figure S56. Picture of clonogenic assay for the VDA activity (with light) and toxicity (without light) with HCT-116 cells.** Both properties were measured using clonogenic assay with 40 min of incubation (contact time between molecules and HCT-116 cells) and 8 d for colony formation. The light treatment consisted of 730 nm LED light at 80 mW/cm$^2$ for 10 min. (a) Molecule **4h** 0-6 µM. (b) Molecule **4q** at 0-2.5 µM. In average, 500 cells were seeded and analyzed in each plate.

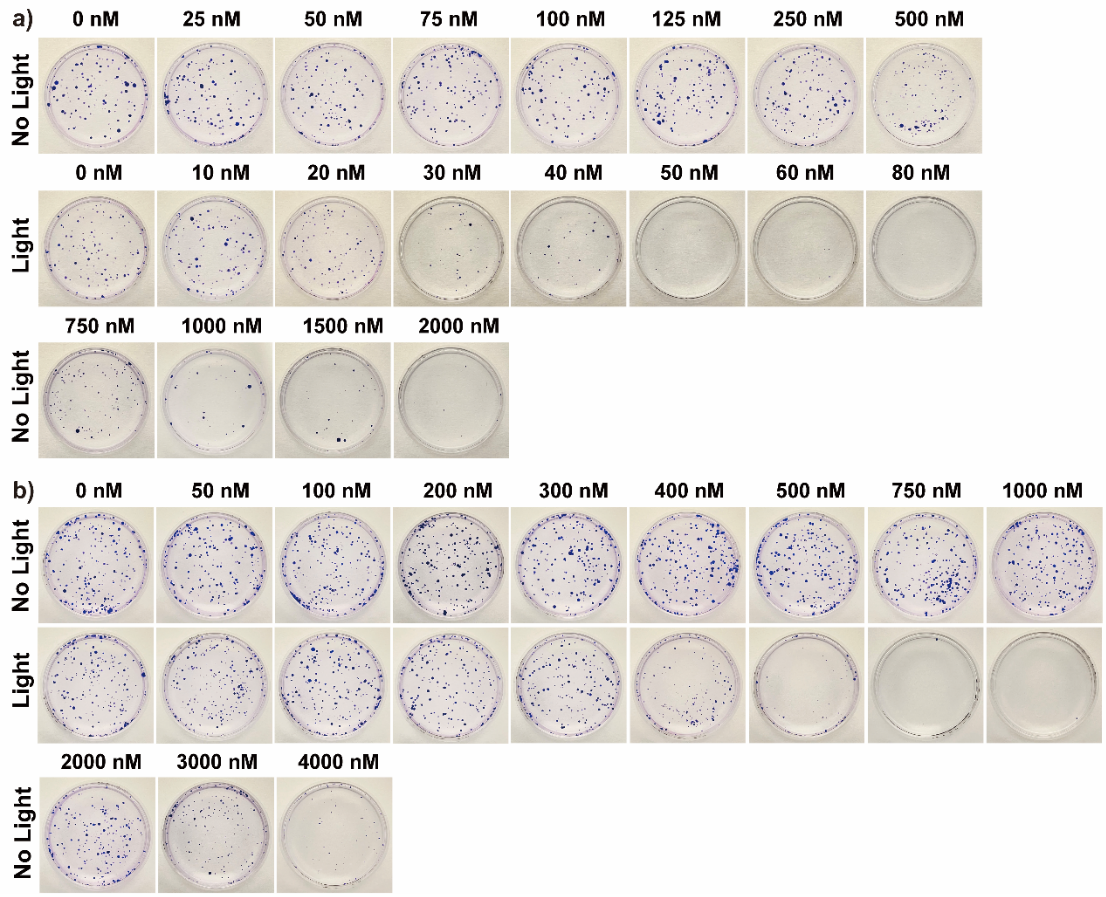


**Figure S57. Picture of clonogenic assay for the VDA activity (with light) and toxicity (without light) with HCT-116 cells.** Both properties were measured using clonogenic assay with 40 min of incubation (contact time between molecules and HCT-116 cells) and 8 d for colony formation. The light treatment consisted of 730 nm LED light at 80 mW/cm$^2$ for 10 min. (a) Molecule **4p** 0-2 µM. (b) Cy7.5 amine at 0-4 µM. In average, 500 cells were seeded and analyzed in each plate.

## 9. References

1. C. Ayala-Orozco, D. Galvez-Aranda, A. Corona, J. M. Seminario, R. Rangel, J. N. Myers, J. M. Tour, *Nat. Chem.*, **2024**, *16*, 456–465.
2. C. Ayala-Orozco, G. Li, B. Li, V. Vardanyan, A. B. Kolomeisky, J. M. Tour, *Adv. Mater.*, **2024**, 2309910.

3. C. Ayala-Orozco, V. Vardanyan, K. Lopez-Jaime, Z. Wang, J. Seminario, A. Kolomeisky, J. M. Tour, *ChemRxiv.* preprint, DOI: 10.26434/chemrxiv-2024-8cfcc.
4. J. Liu, W. Zhang, C. Zhou, M. Li, X. Wang, W. Zhang, Z. Liu, L. Wu, T. D. James, P. Li, B. Tang, *J. Am. Chem. Soc.* **2022**, *144*, 13586−13599.
5. K. Kiyose, K. Hanaoka, D. Oushiki, T. Nakamura, M. Kajimura, M. Suematsu, H. Nishimatsu, T. Yamane, T. Terai, Y. Hirata, T. Nagano, *J. Am. Chem. Soc.* **2010**, *132*, 15846−15848.

## 10. NMR spectra

**$^{1}$H-NMR spectrum of 2a**

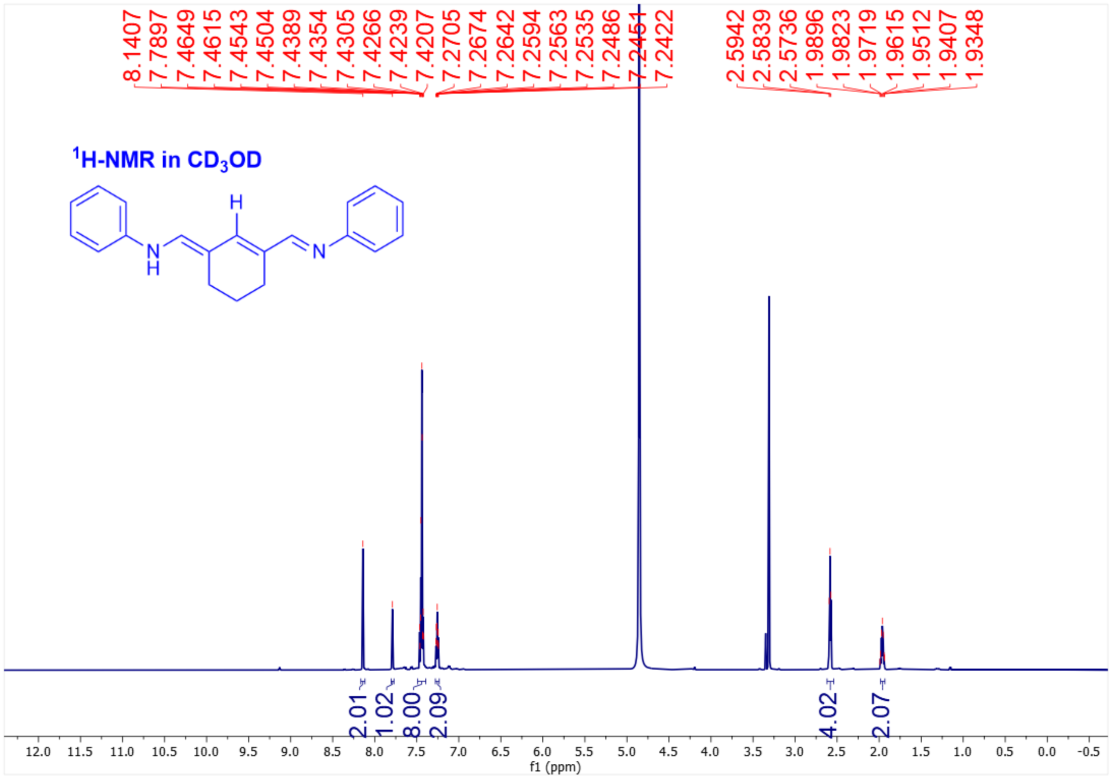


**$^{13}$C-NMR spectrum of 2a**

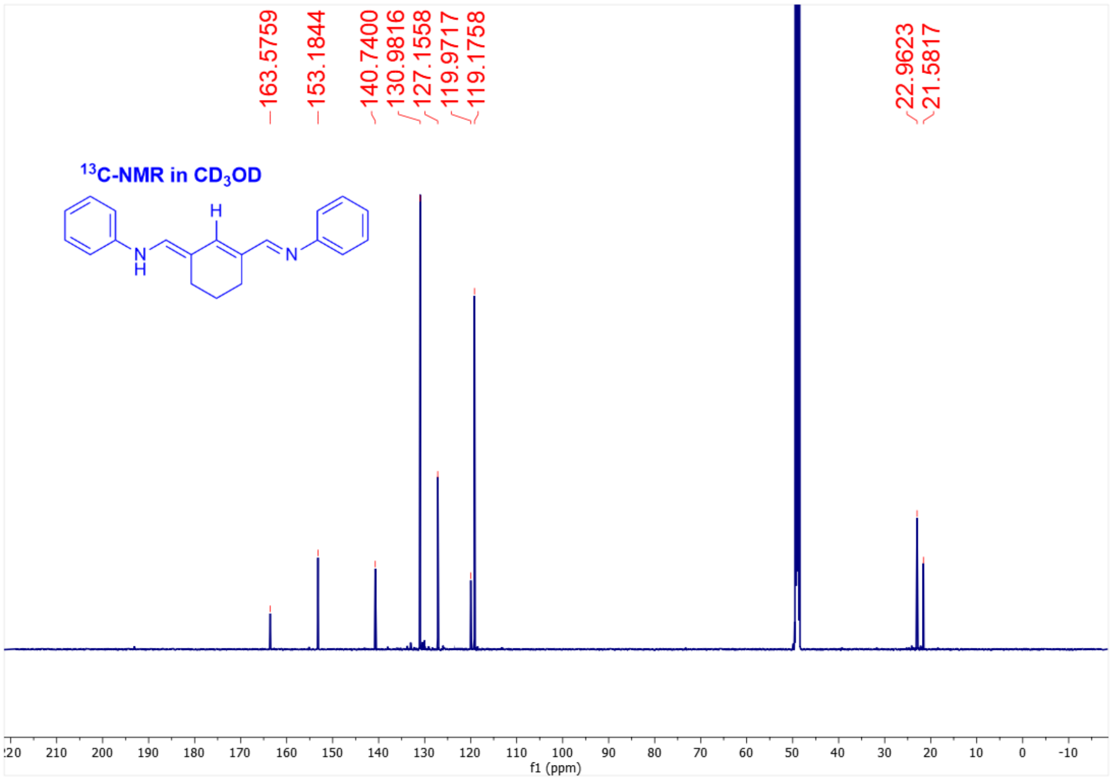

**$^{1}$H-NMR spectrum of 2b**

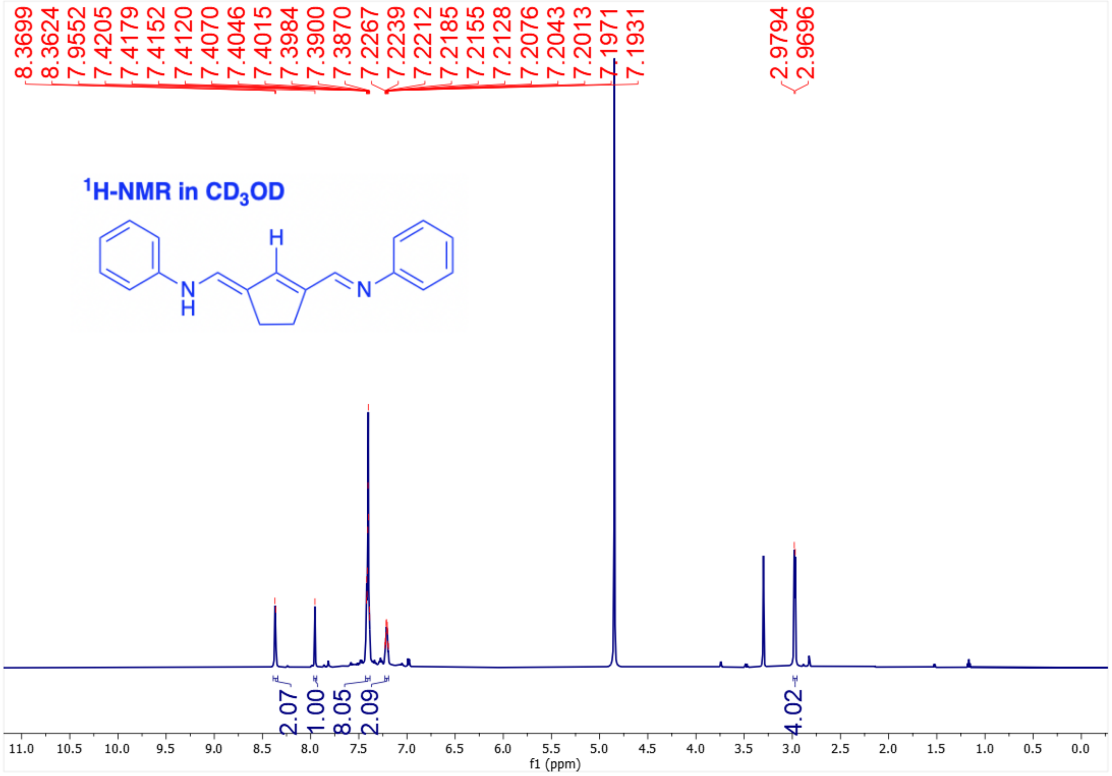


**$^{13}$C-NMR spectrum of 2b**

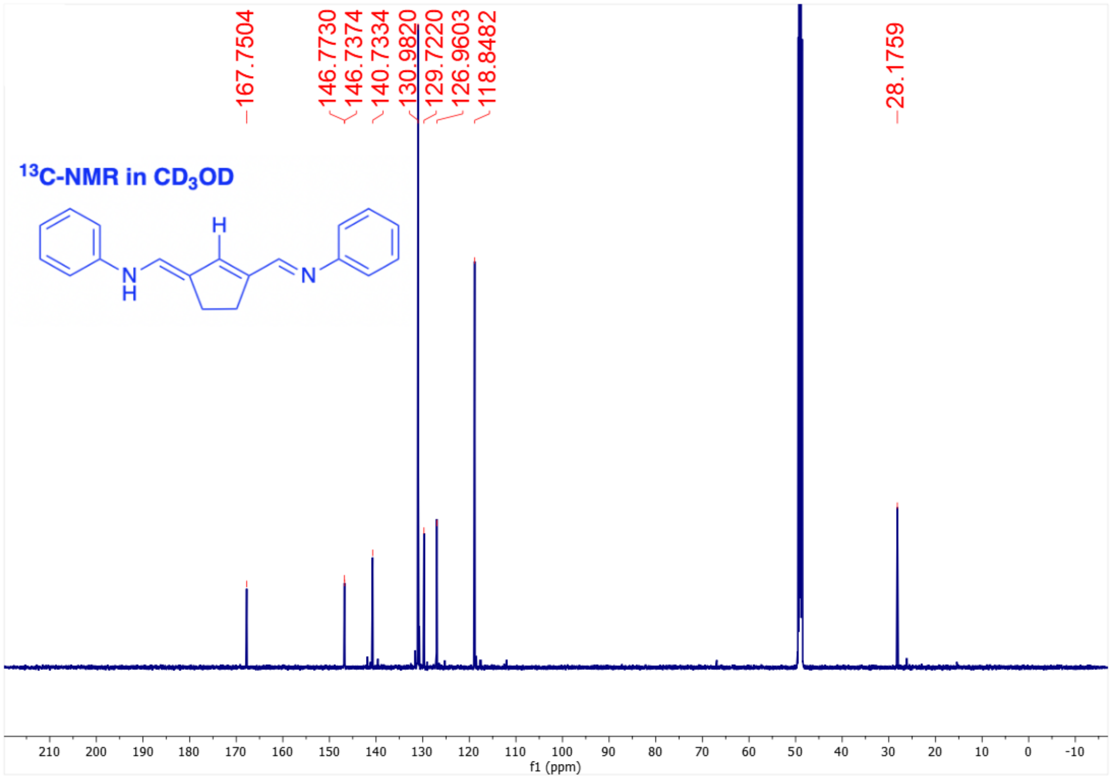

**$^{1}$H-NMR spectrum of 4a**

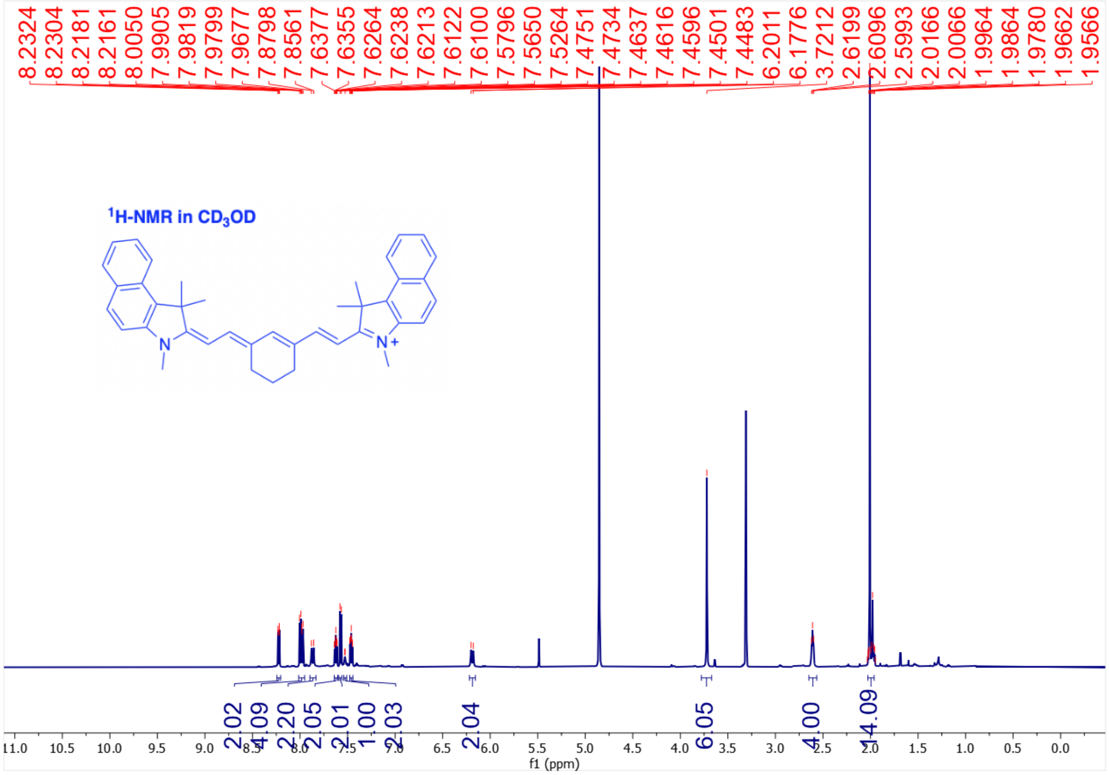


**$^{13}$C-NMR spectrum of 4a**

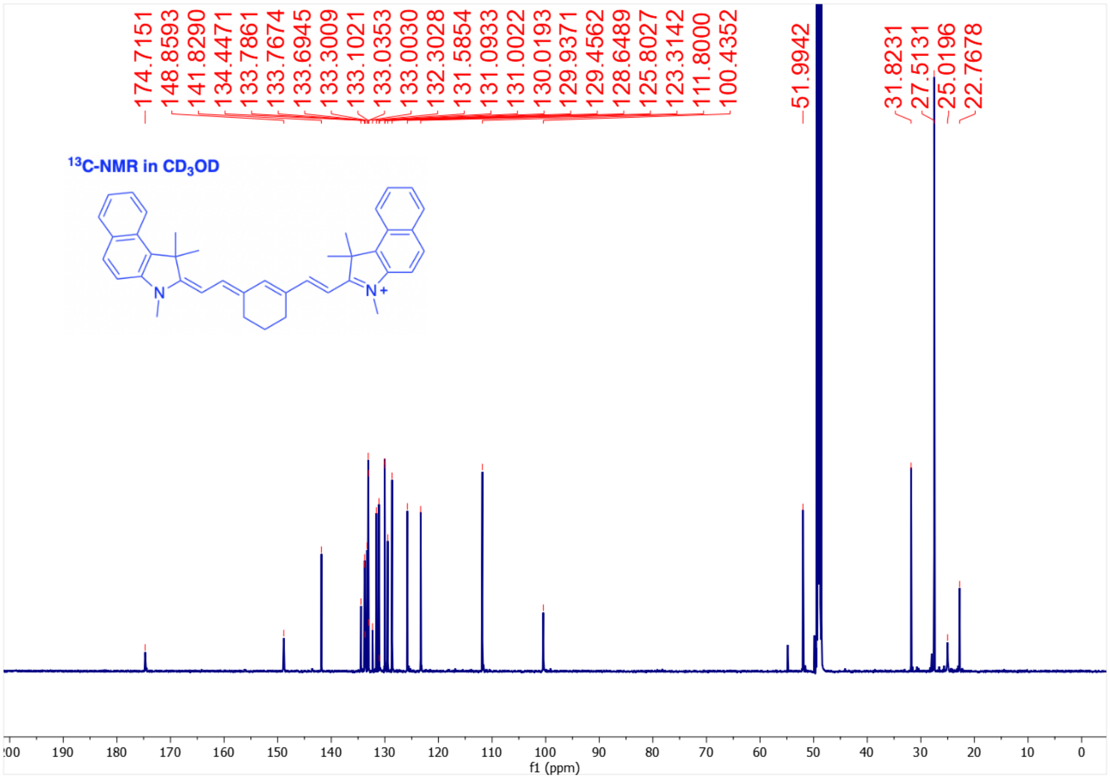

**$^{1}$H-NMR spectrum of 4b**

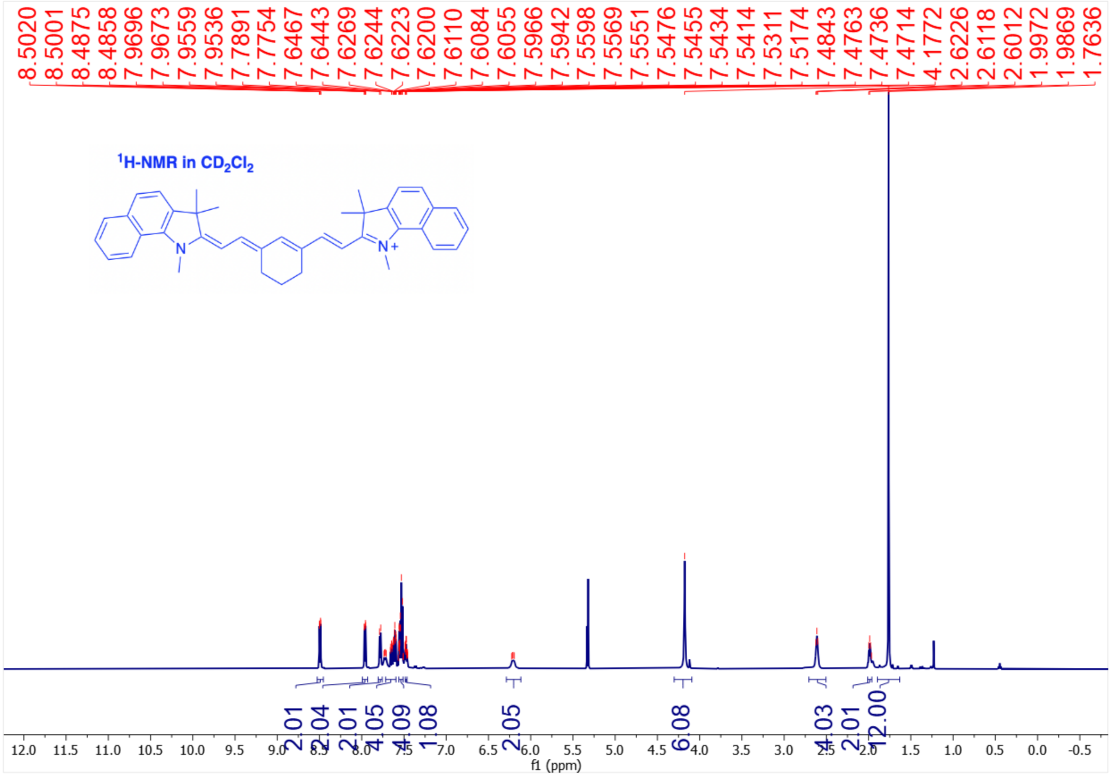


**$^{13}$C-NMR spectrum of 4b**

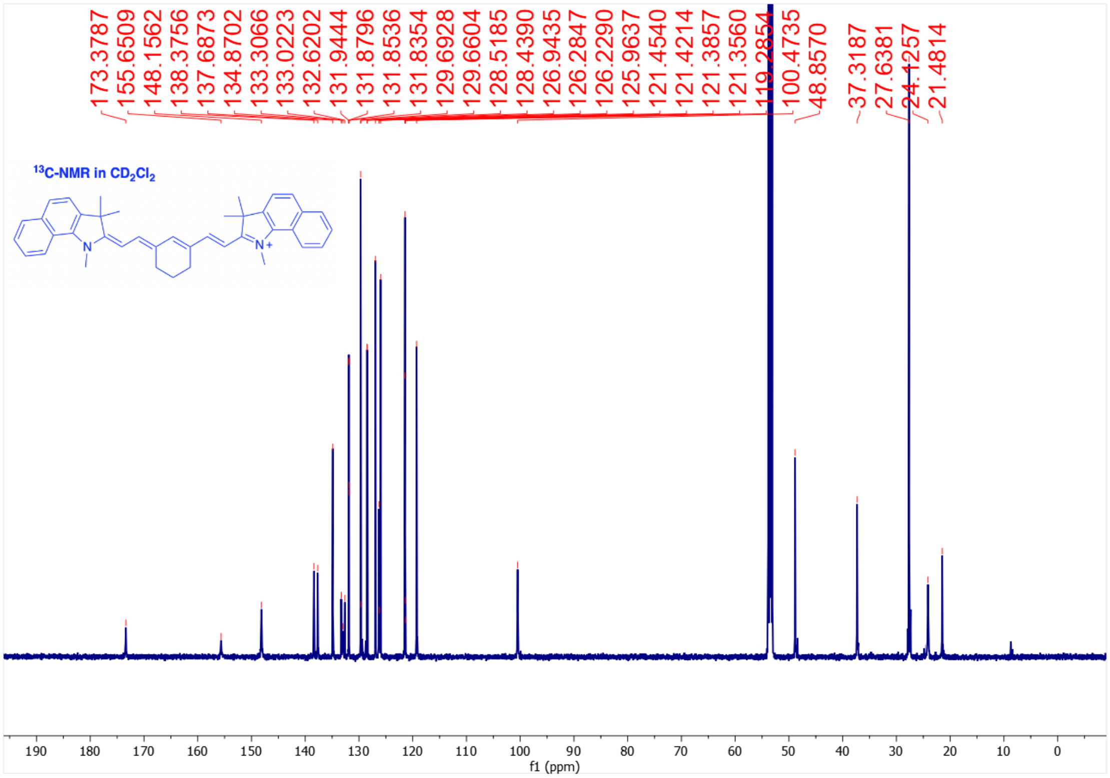

**$^{1}$H-NMR spectrum of 4c**

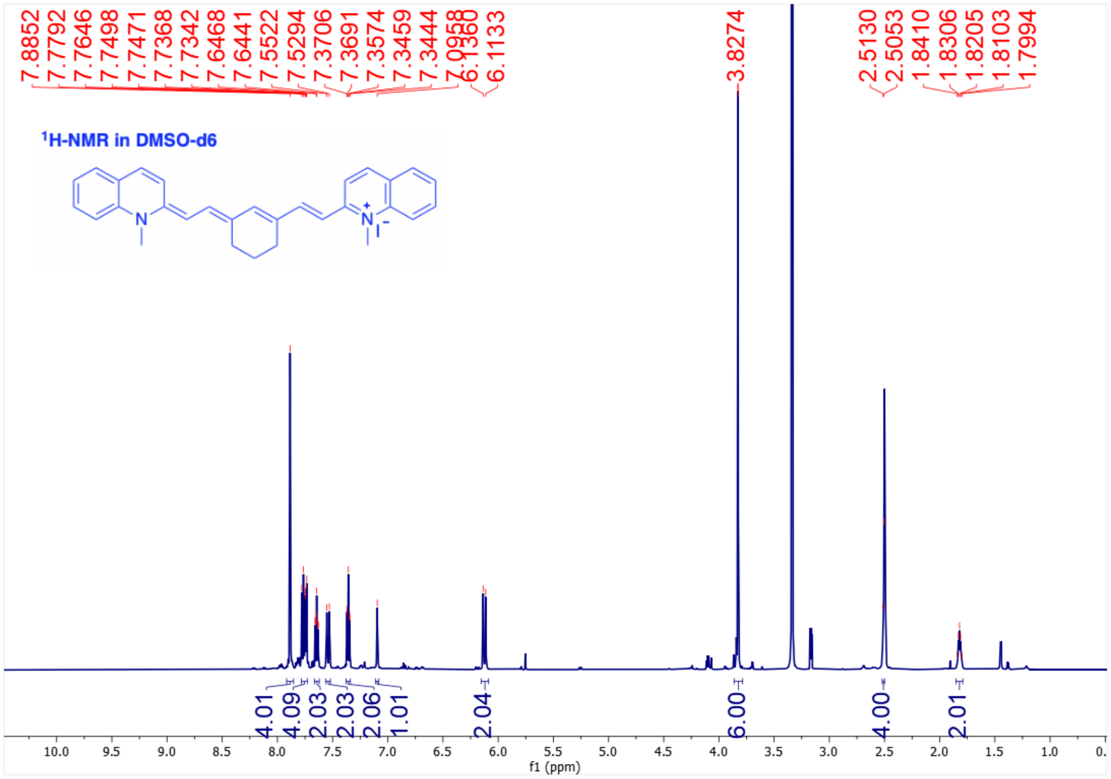


**$^{13}$C-NMR spectrum of 4c**

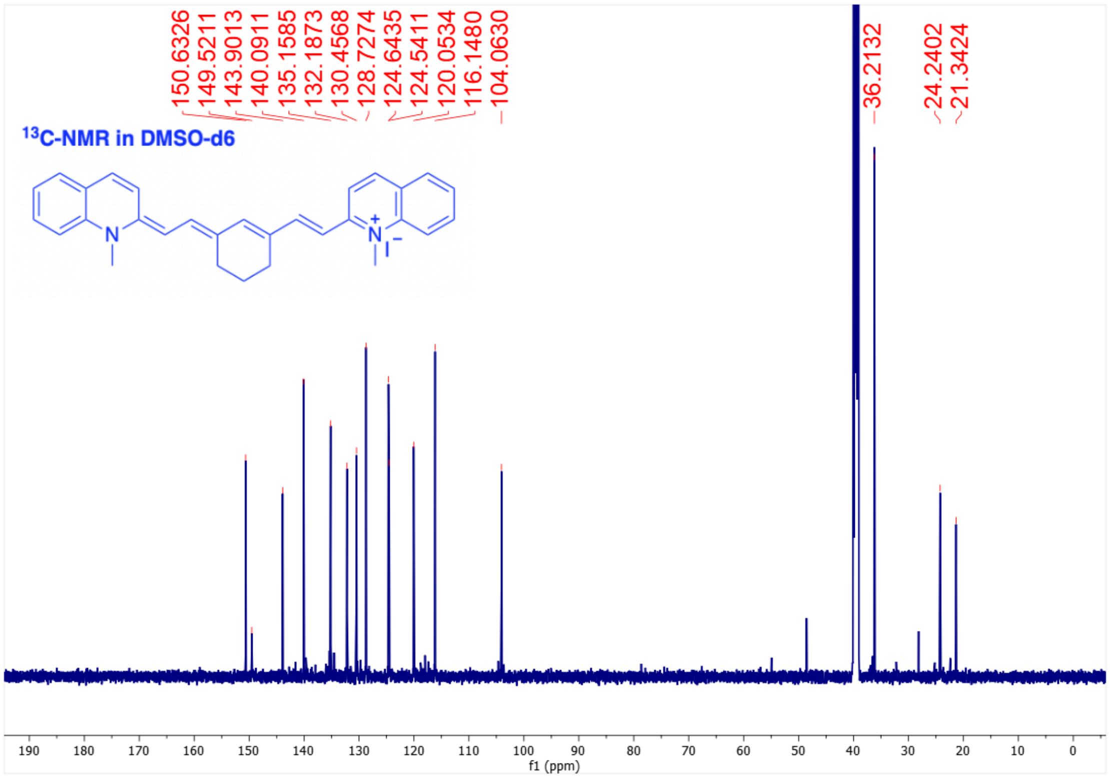

**$^{1}$H-NMR spectrum of 4d**

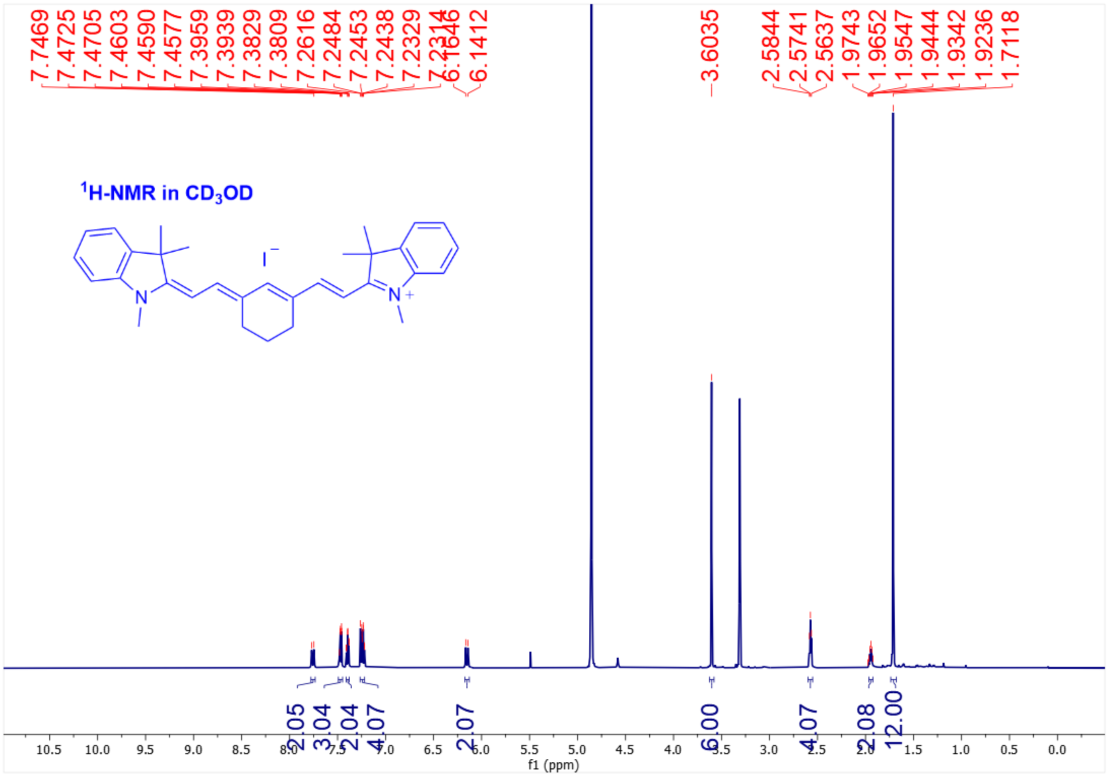


**$^{13}$C-NMR spectrum of 4d**

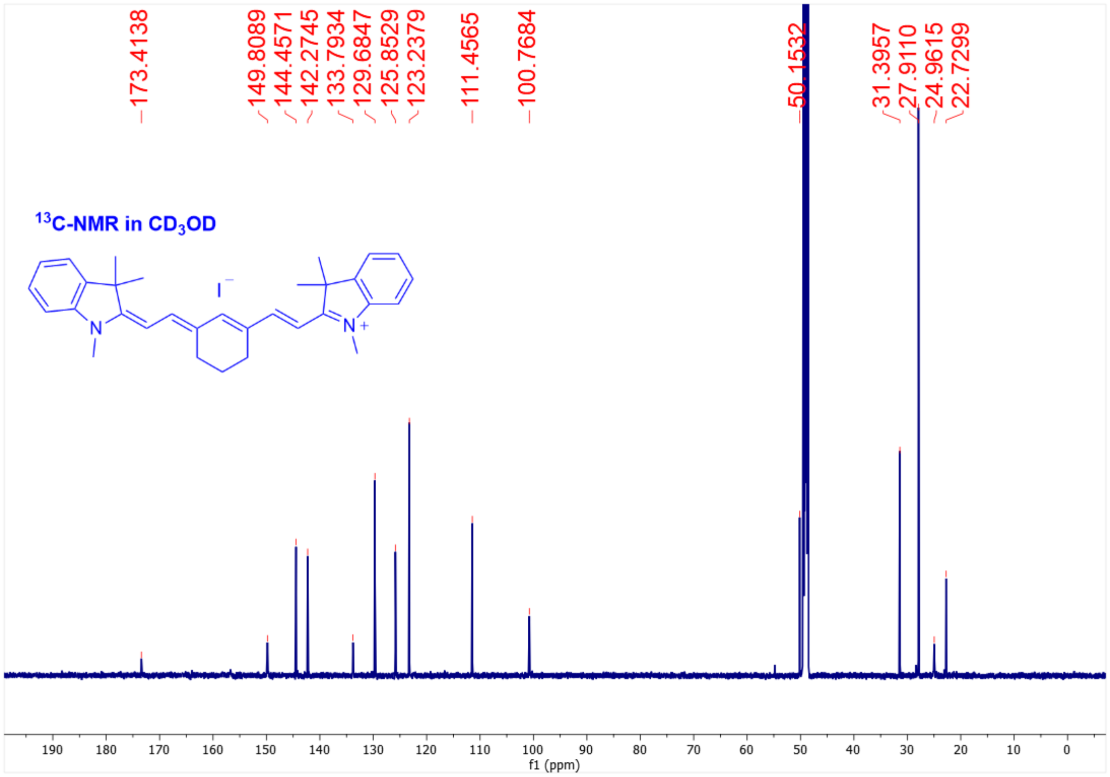

**$^{1}$H-NMR spectrum of 4e**

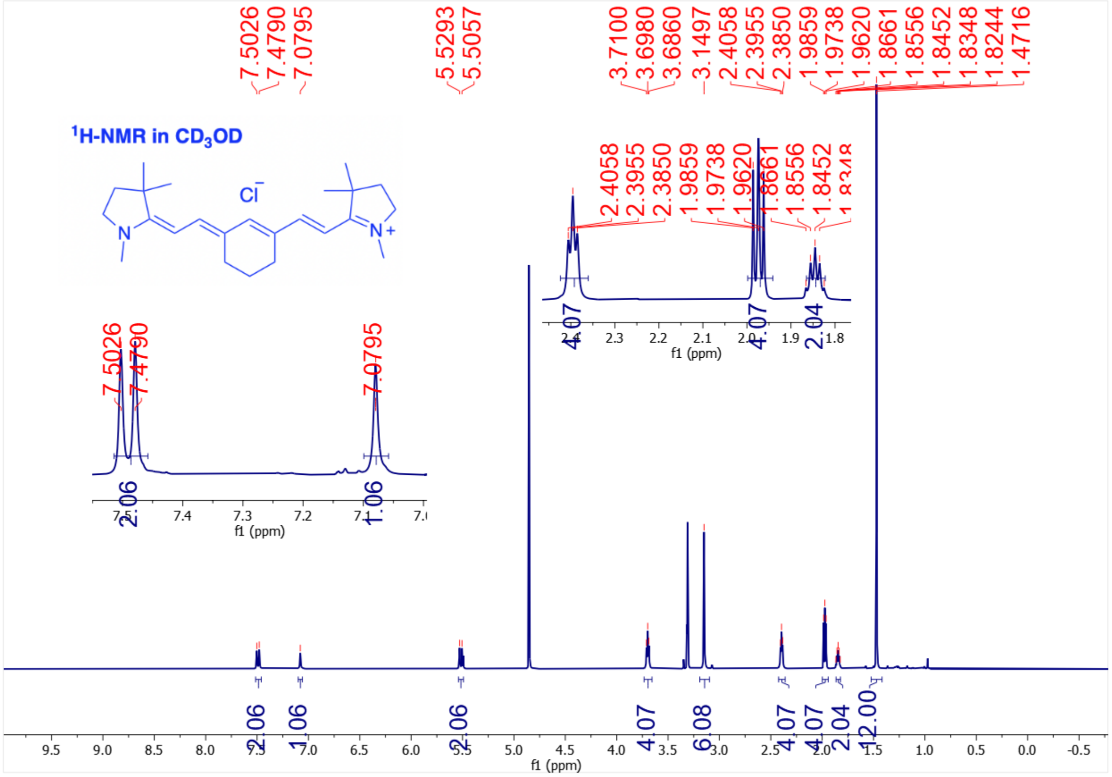


**$^{13}$C-NMR spectrum of 4e**

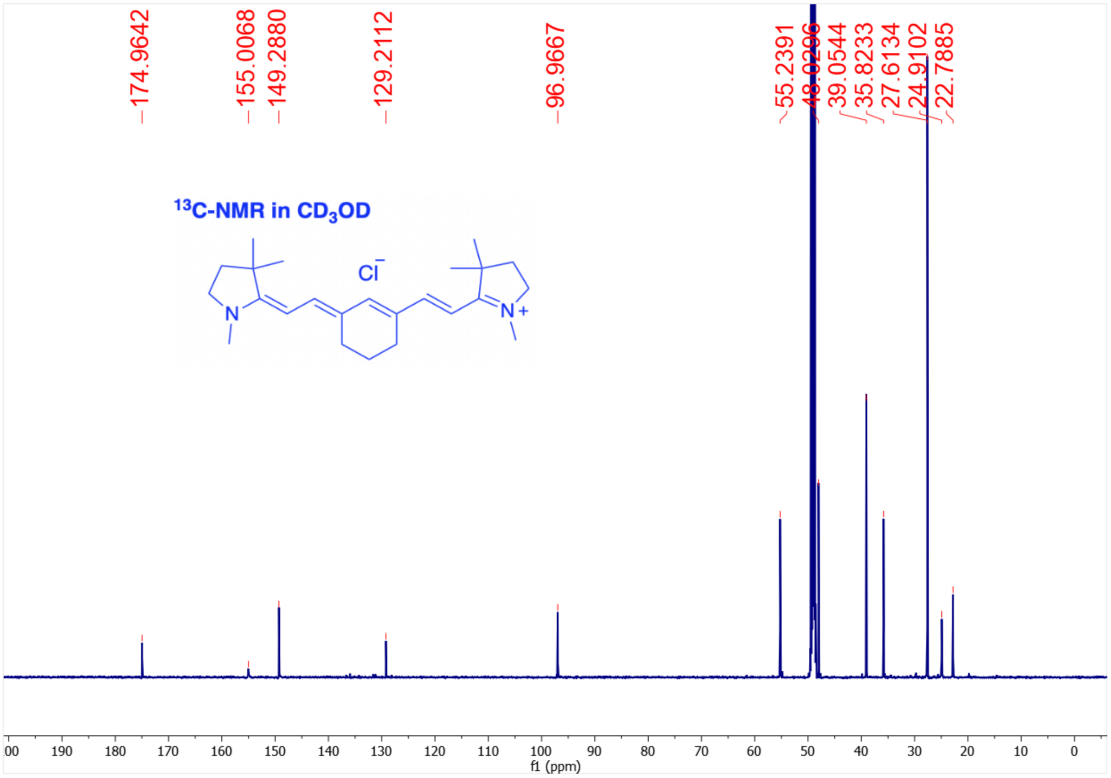

**$^{1}$H-NMR spectrum of 4f**

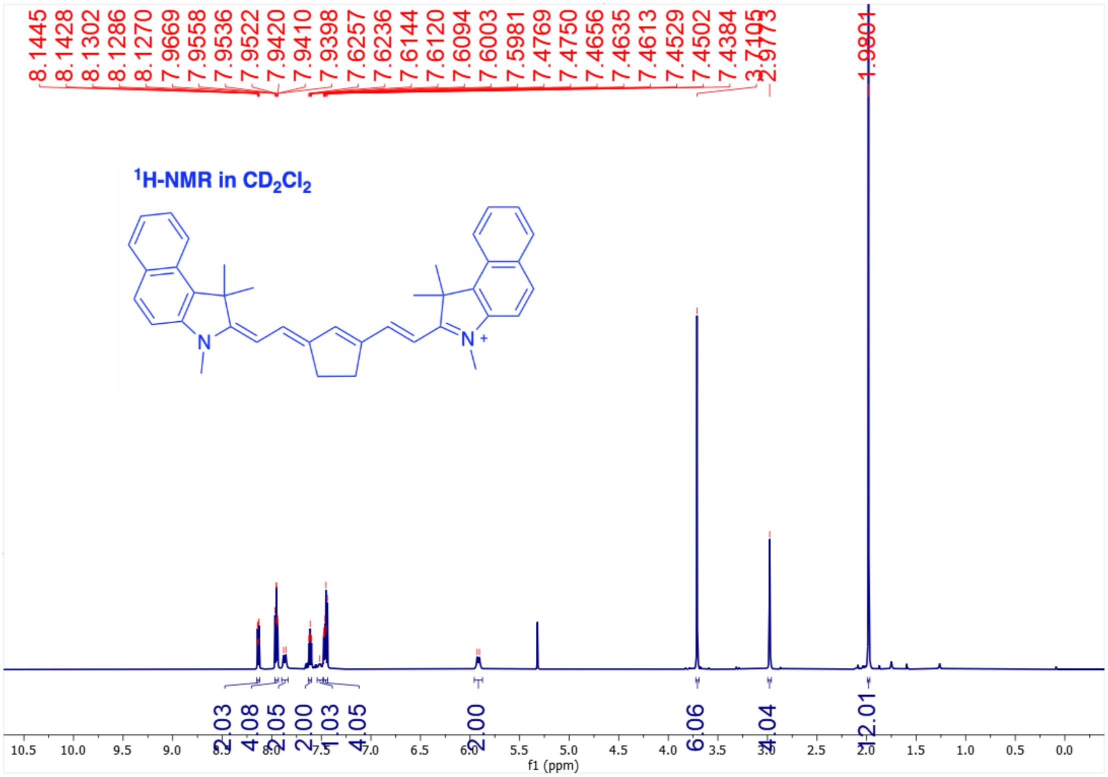


**$^{13}$C-NMR spectrum of 4f**

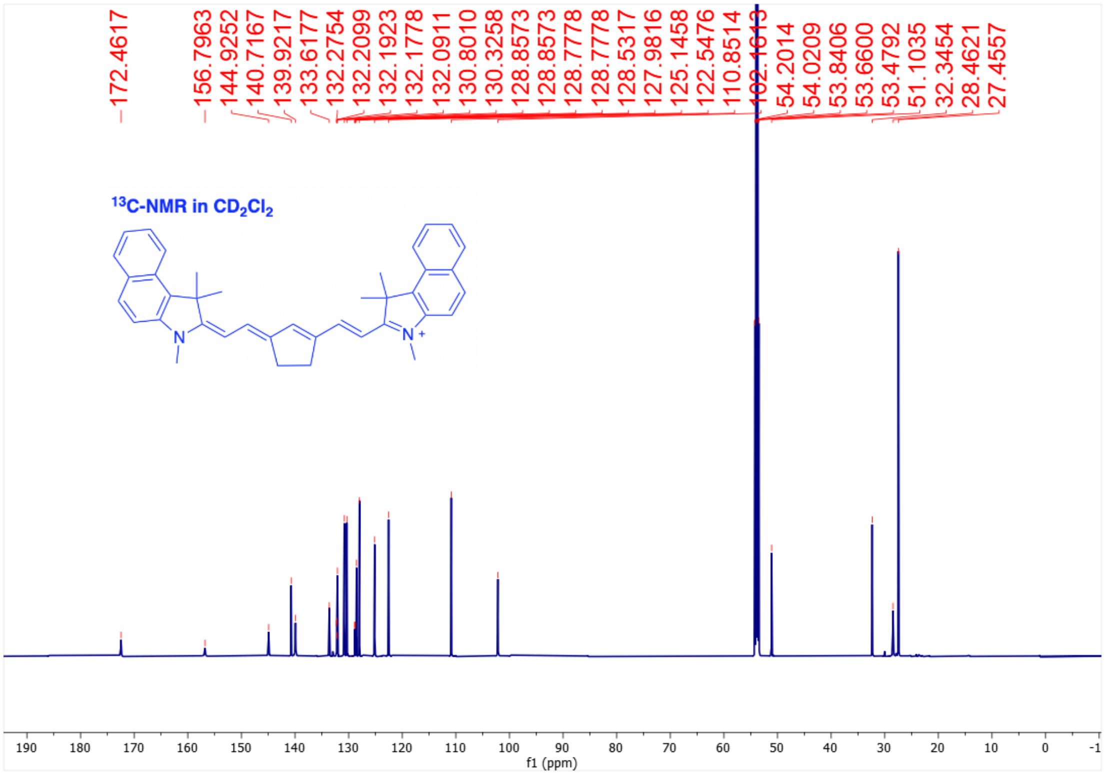

**$^{1}$H-NMR spectrum of 4g**

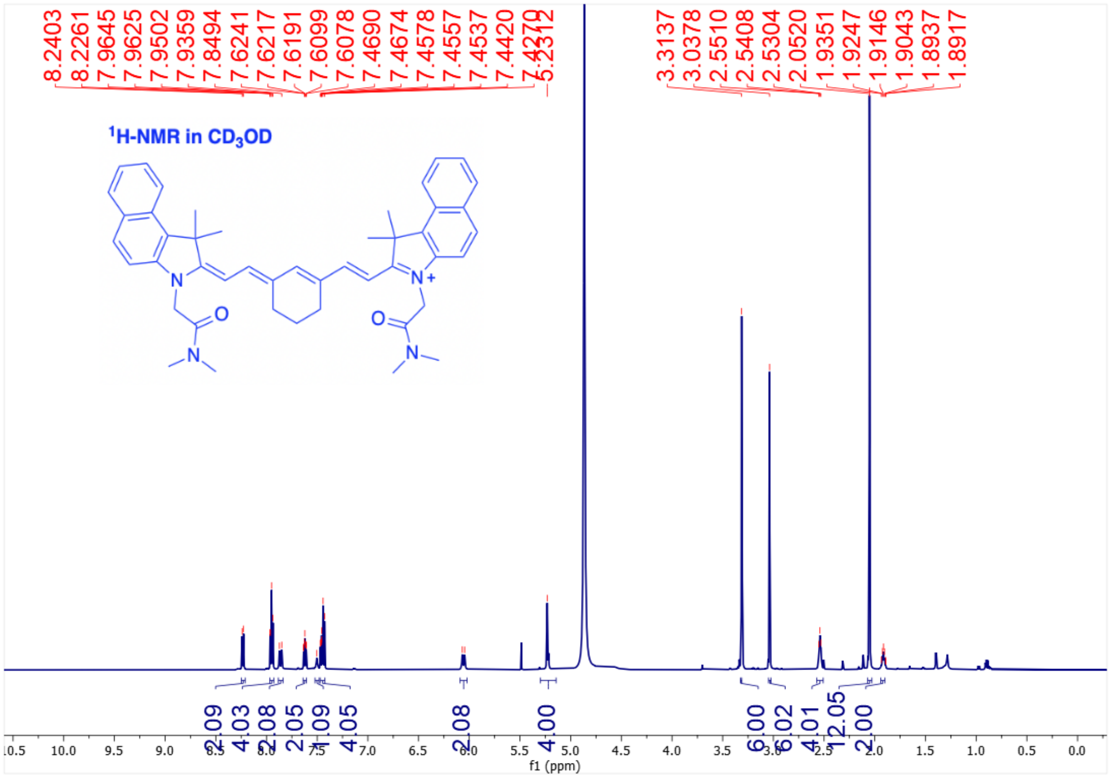


**$^{13}$C-NMR spectrum of 4g**

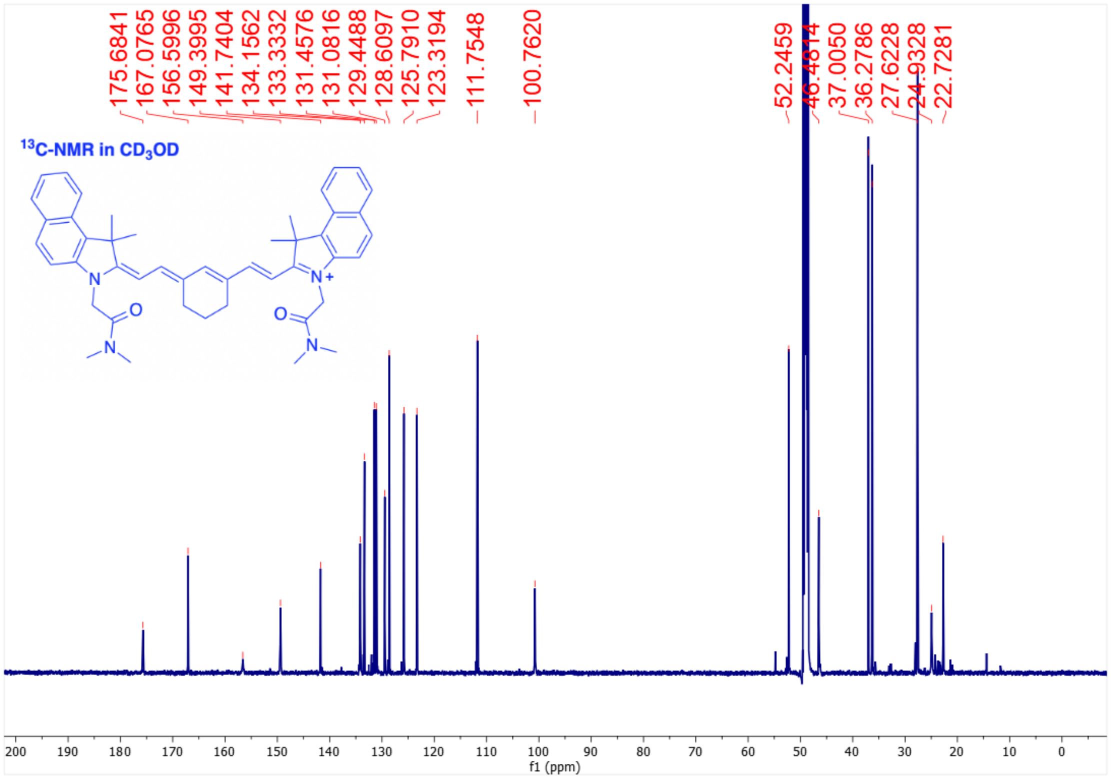

**$^{1}$H-NMR spectrum of 4h**

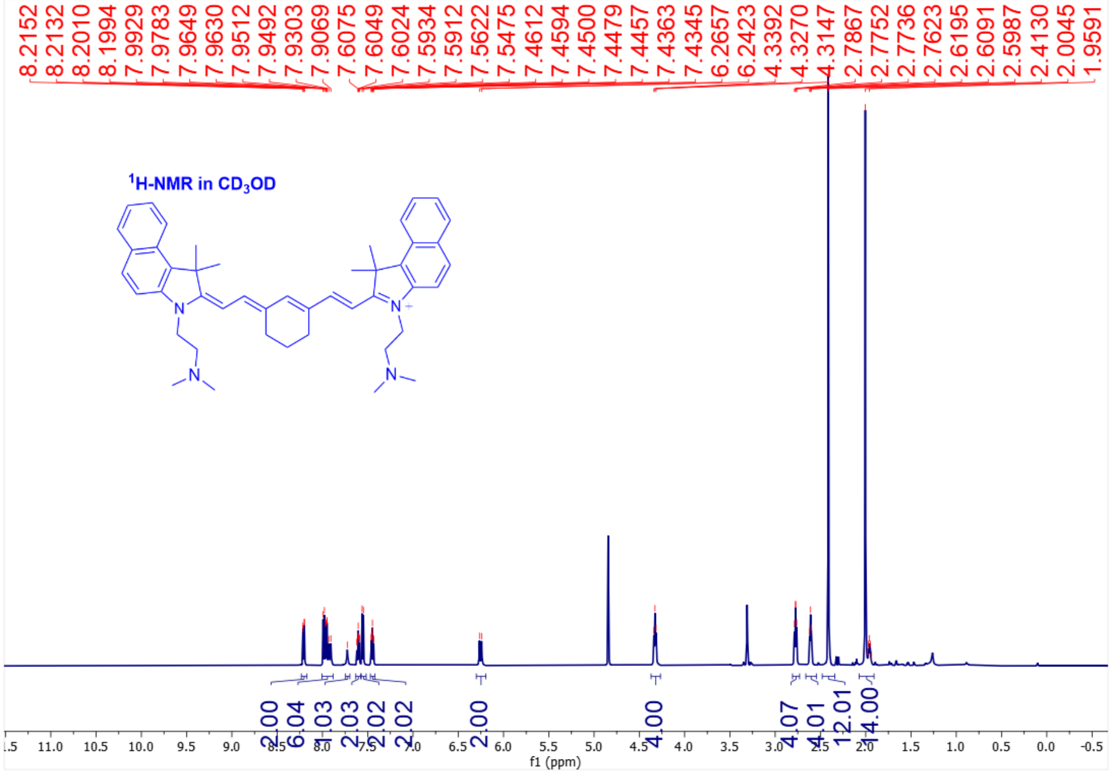


**$^{13}$C-NMR spectrum of 4h**

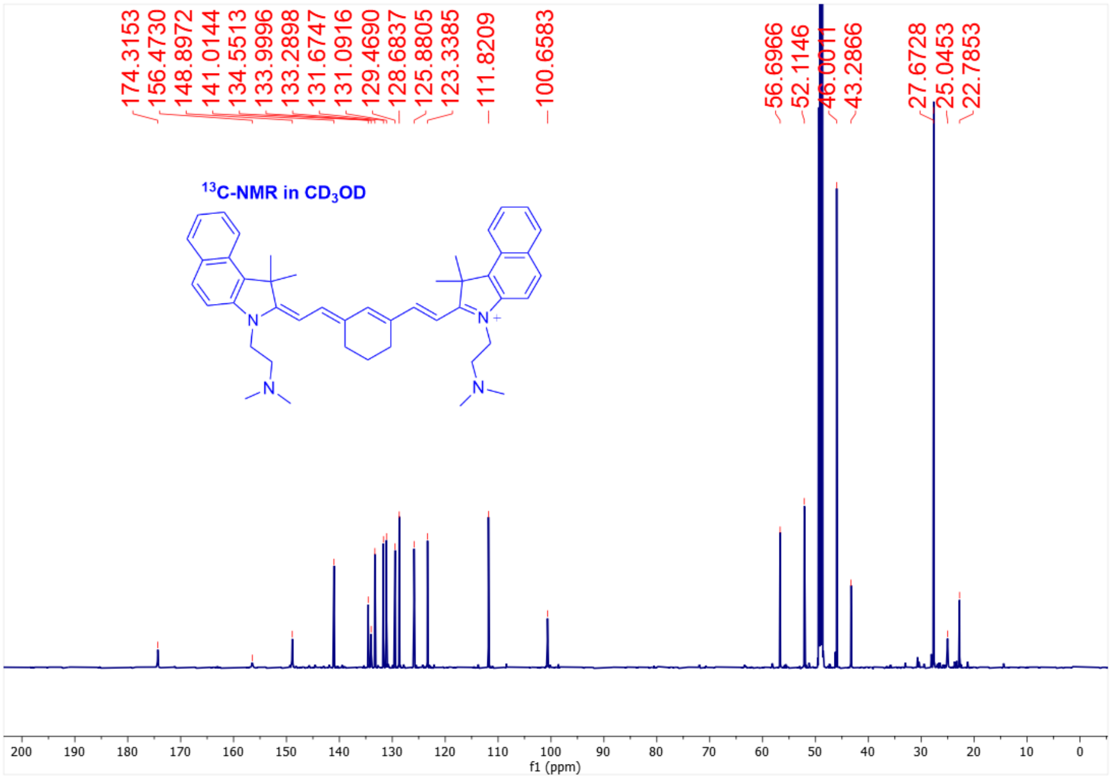

**$^{1}$H-NMR spectrum of 4i**

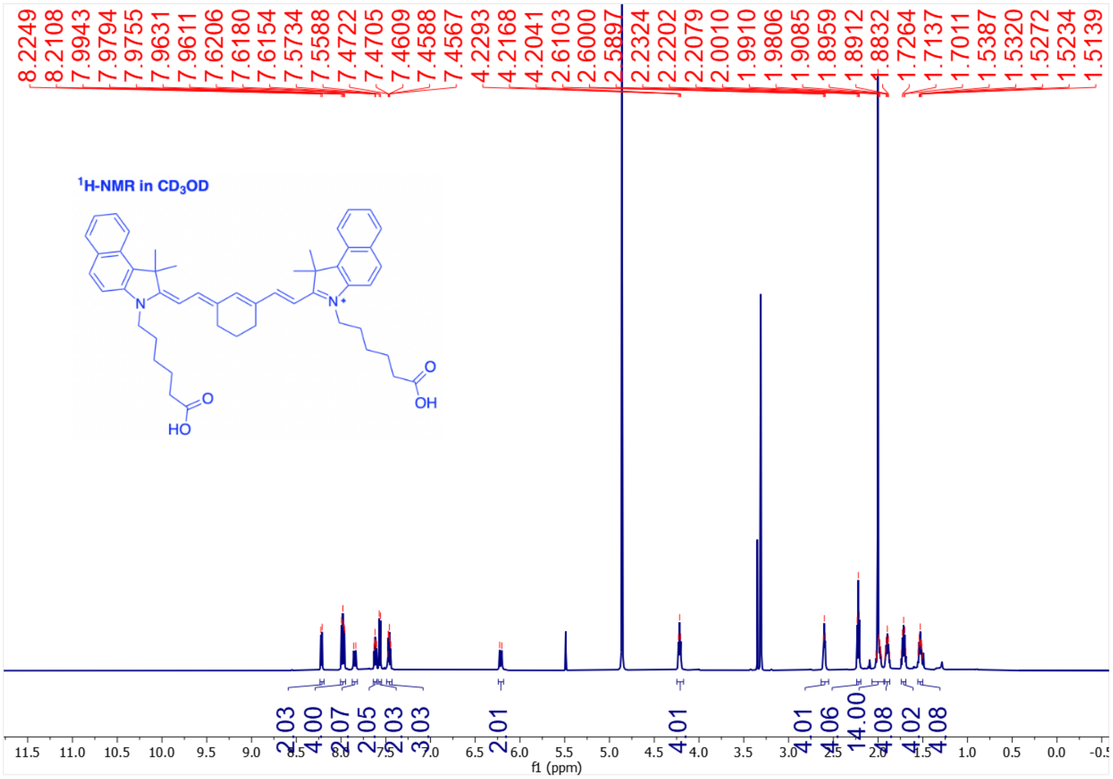


**$^{13}$C-NMR spectrum of 4i**

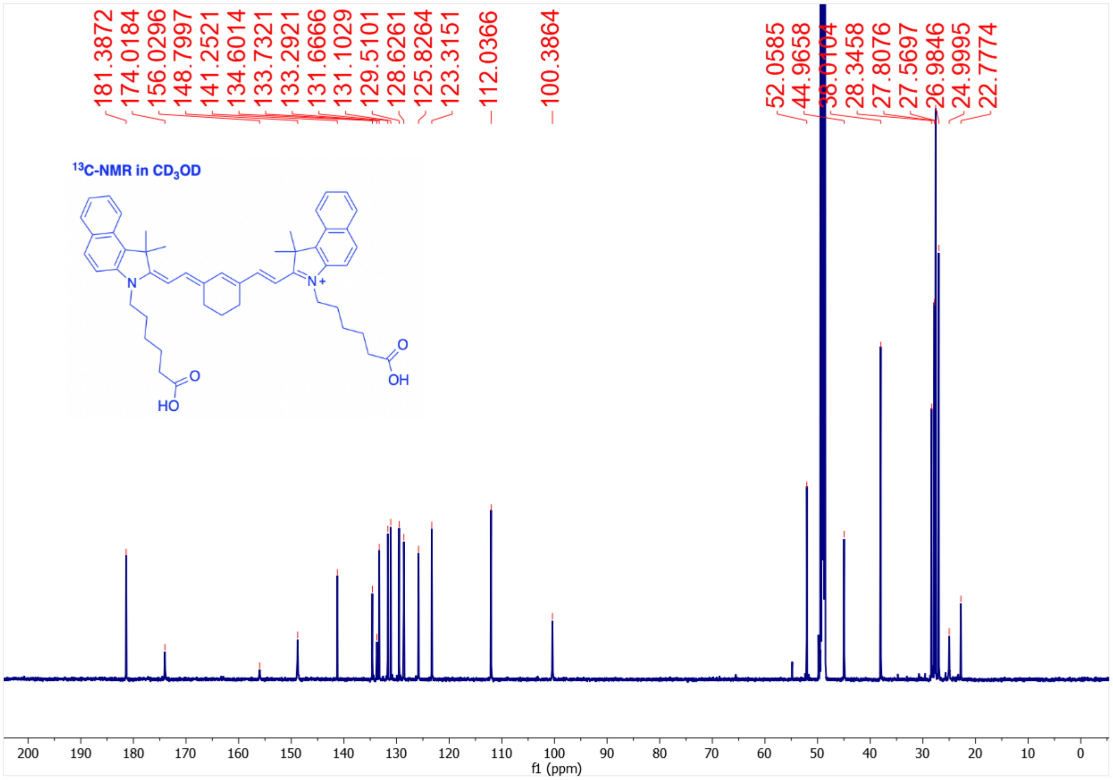

**$^{1}$H-NMR spectrum of 4j**

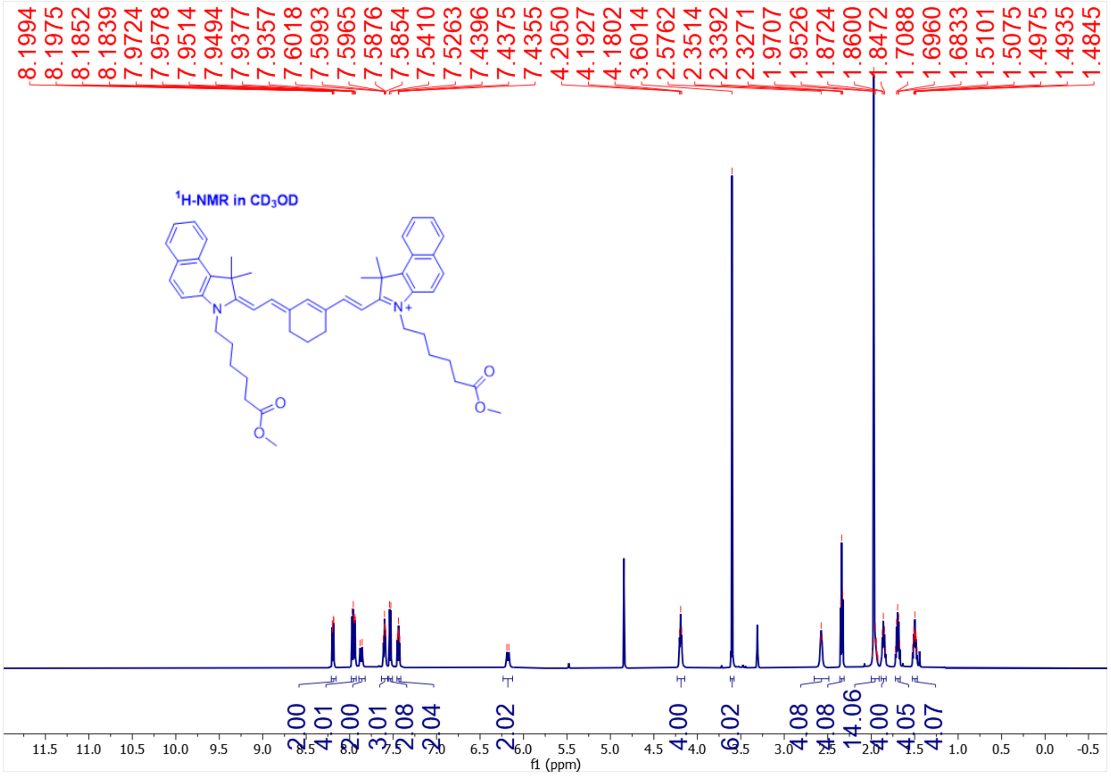

**$^{13}$C-NMR spectrum of 4j**

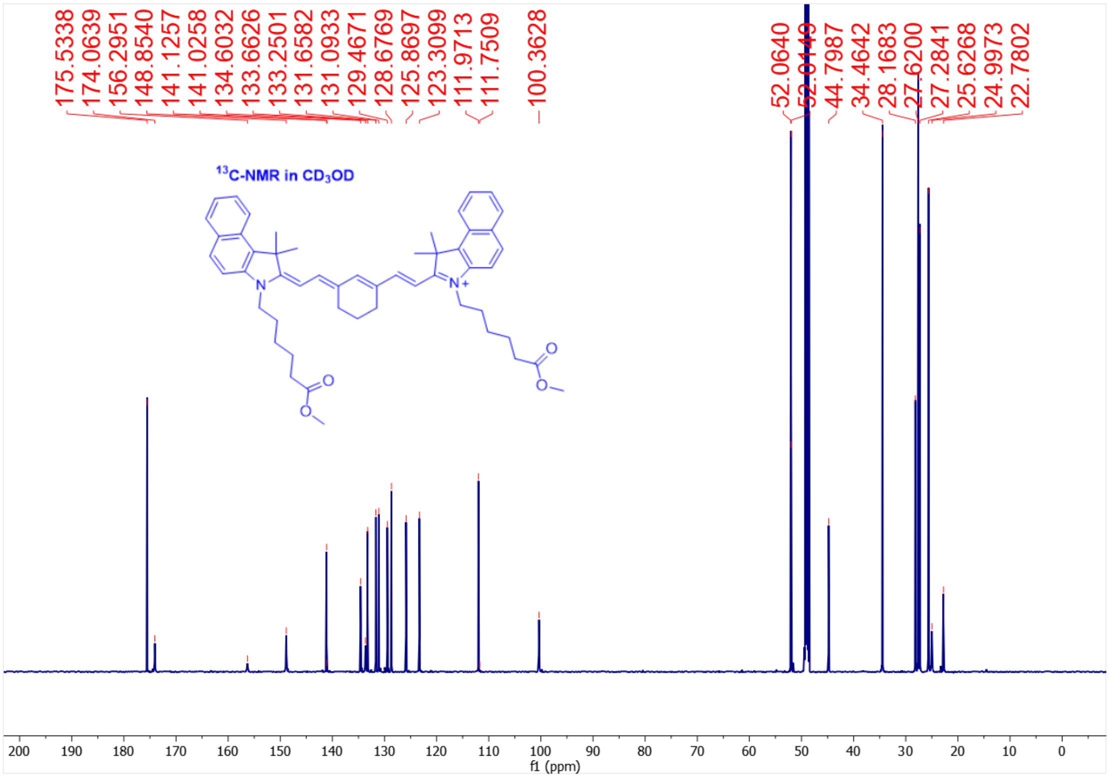

$^{1}$H-NMR spectrum of 4k

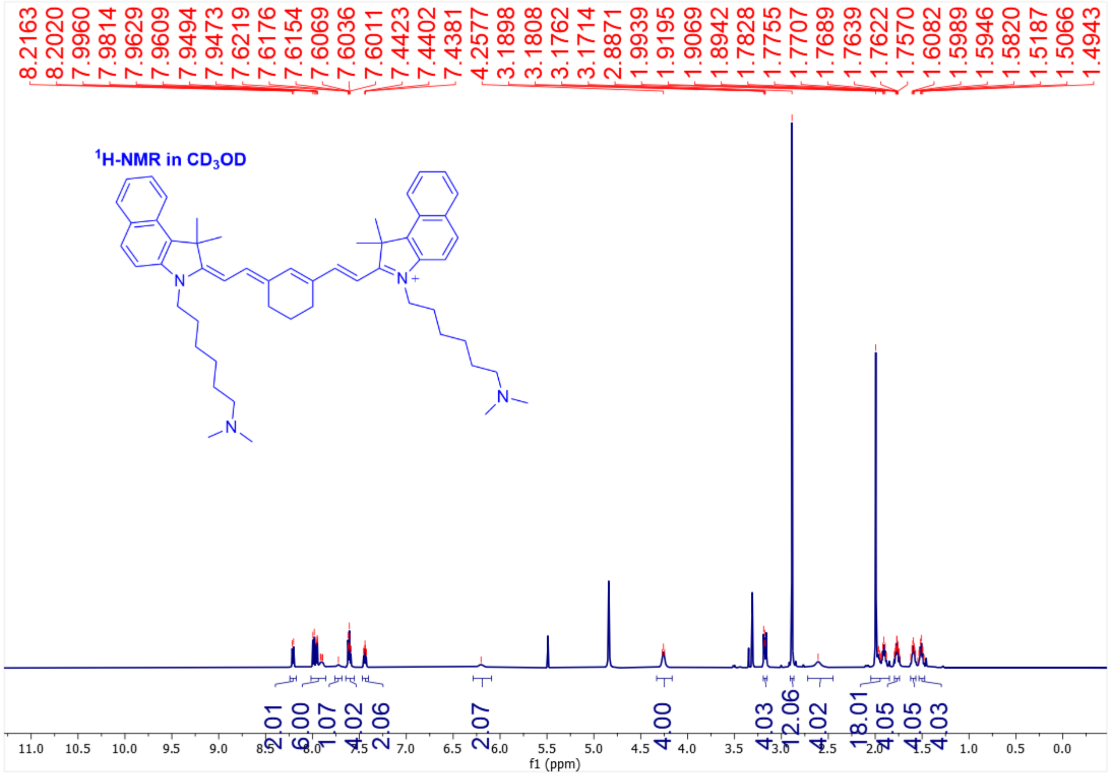


$^{13}$C-NMR spectrum of 4k

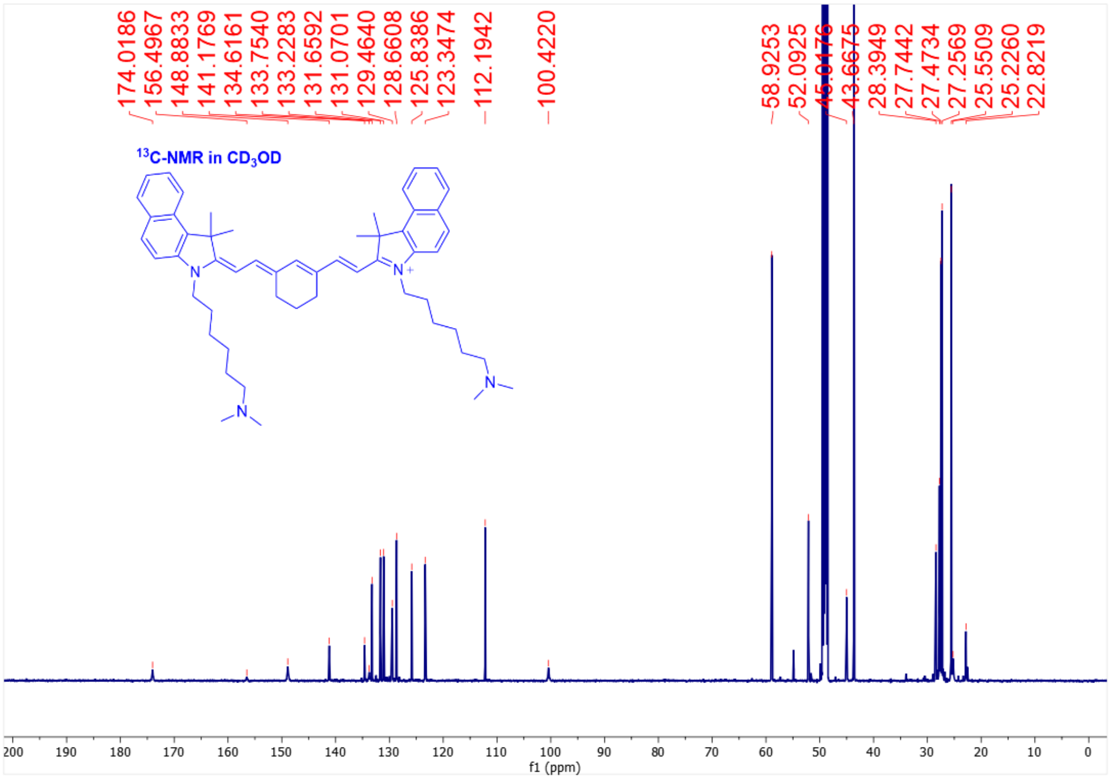

**$^{1}$H-NMR spectrum of 4l**

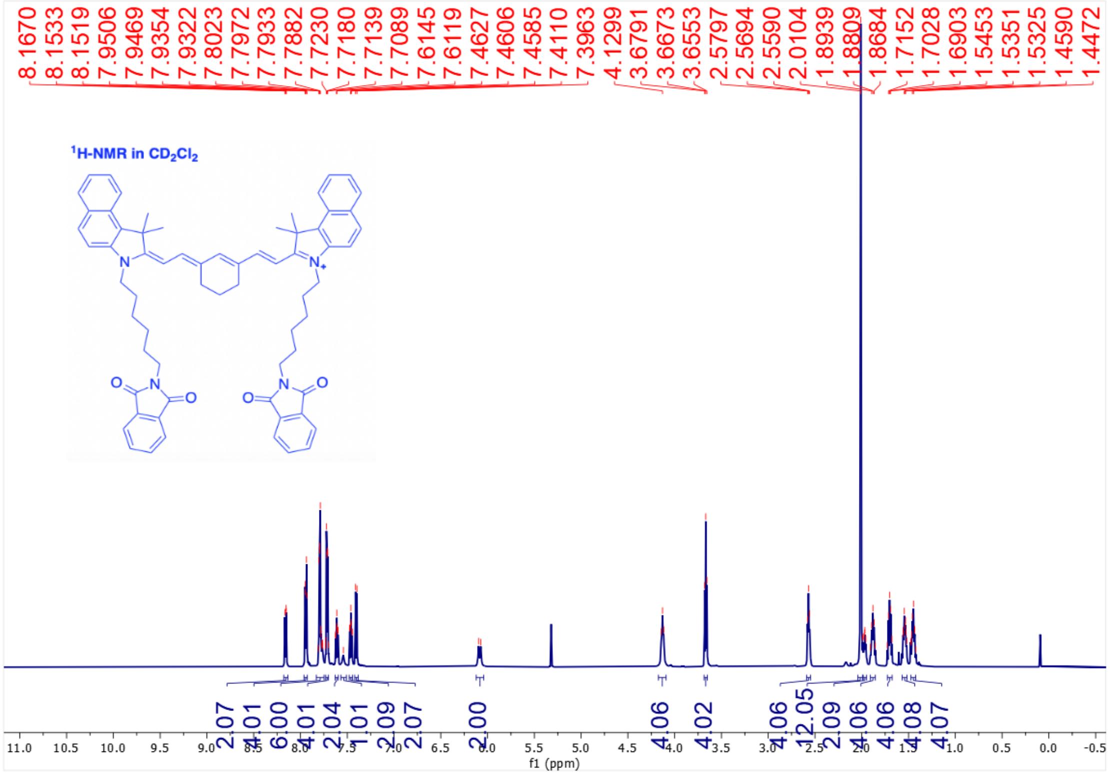

**$^{13}$C-NMR spectrum of 4l**

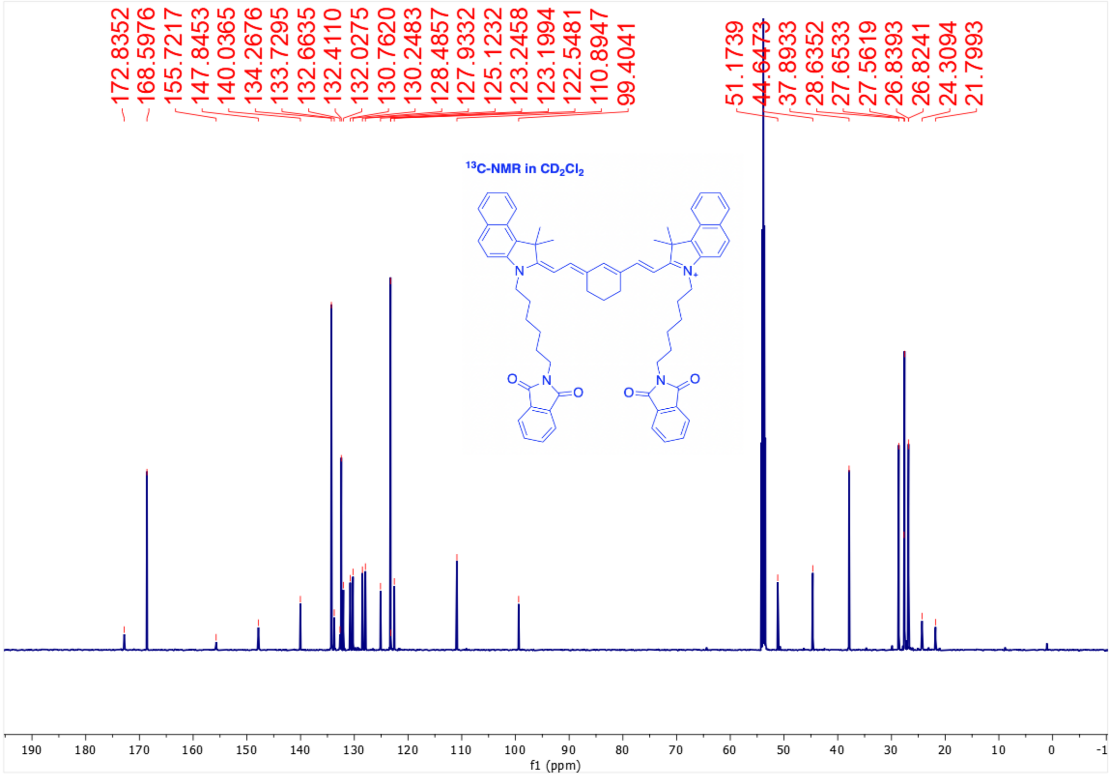

**$^{1}$H-NMR spectrum of 4m**

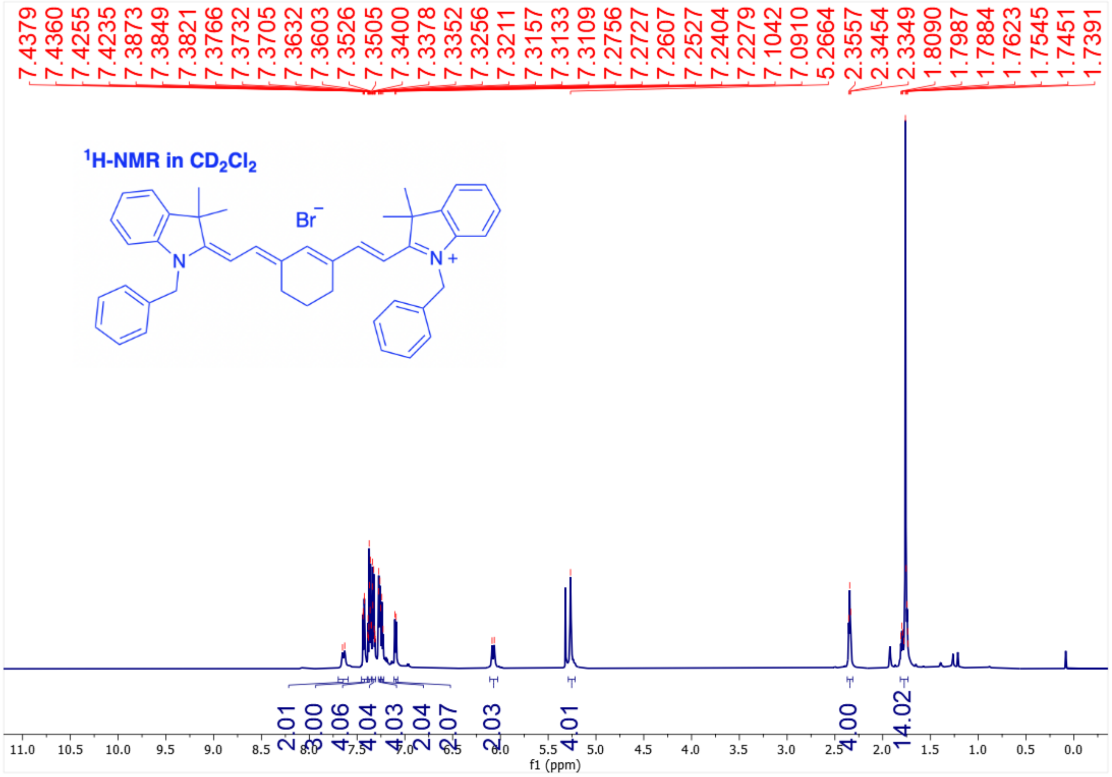

**$^{13}$C-NMR spectrum of 4m**

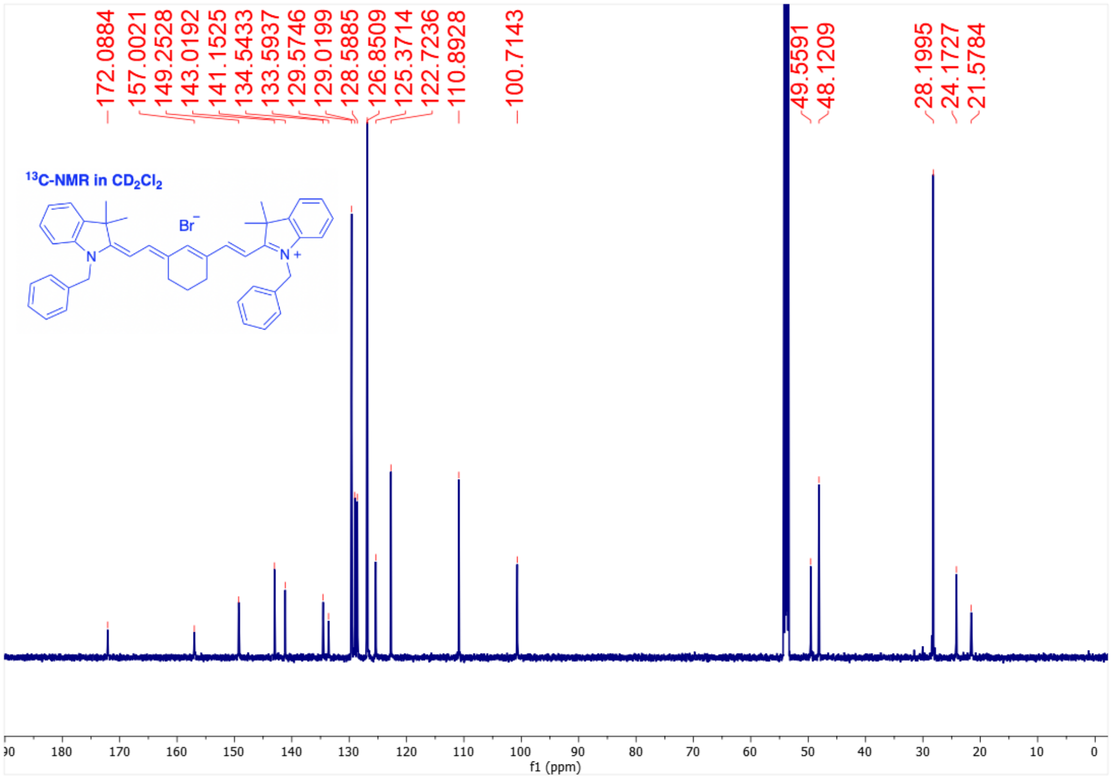

$^{1}$H-NMR spectrum of 4n

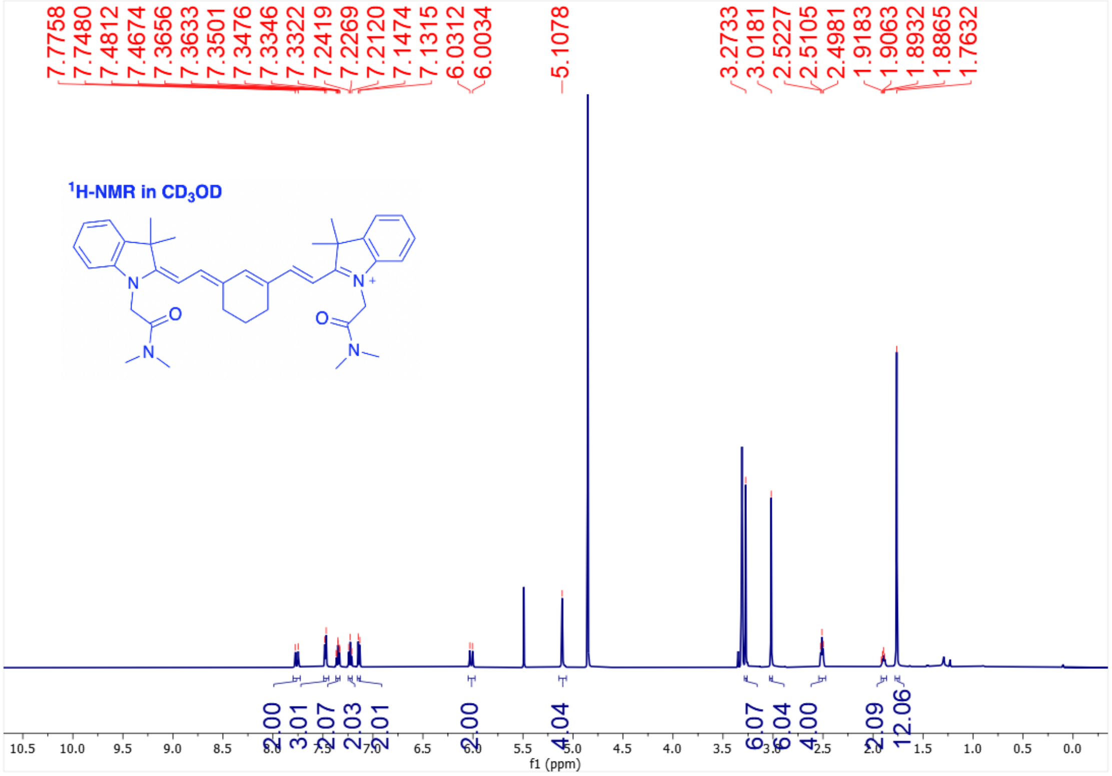


$^{13}$C-NMR spectrum of 4n

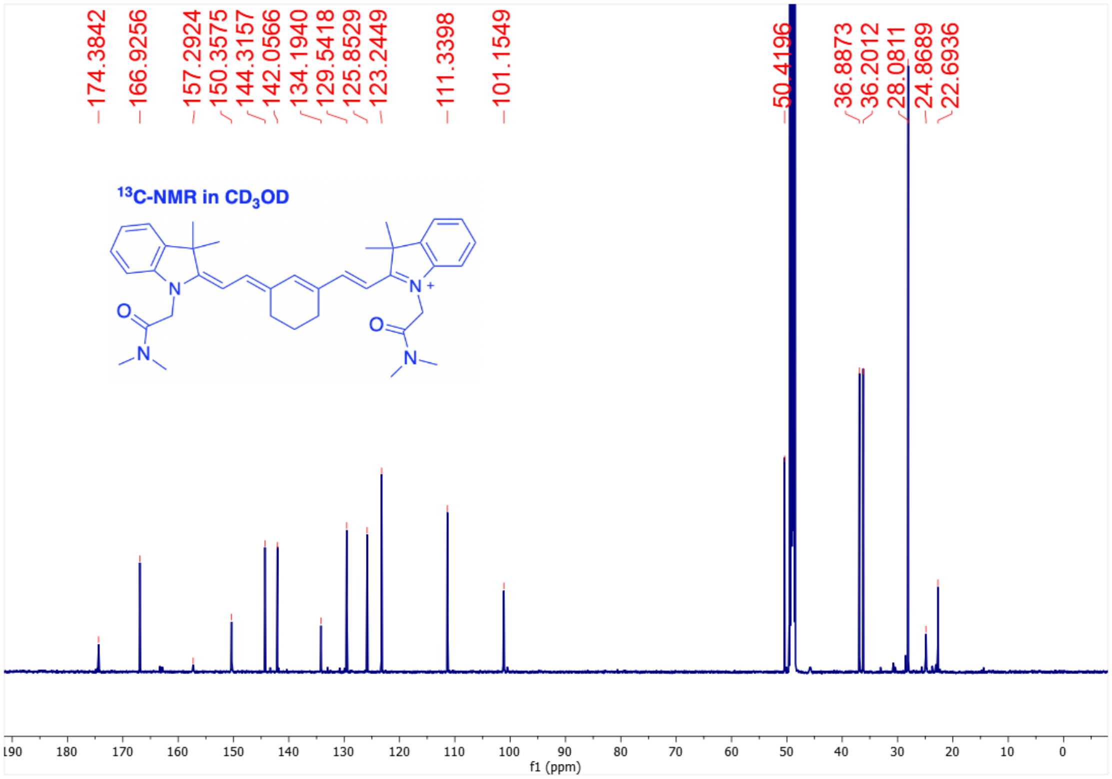

$^{1}$H-NMR spectrum of 4o

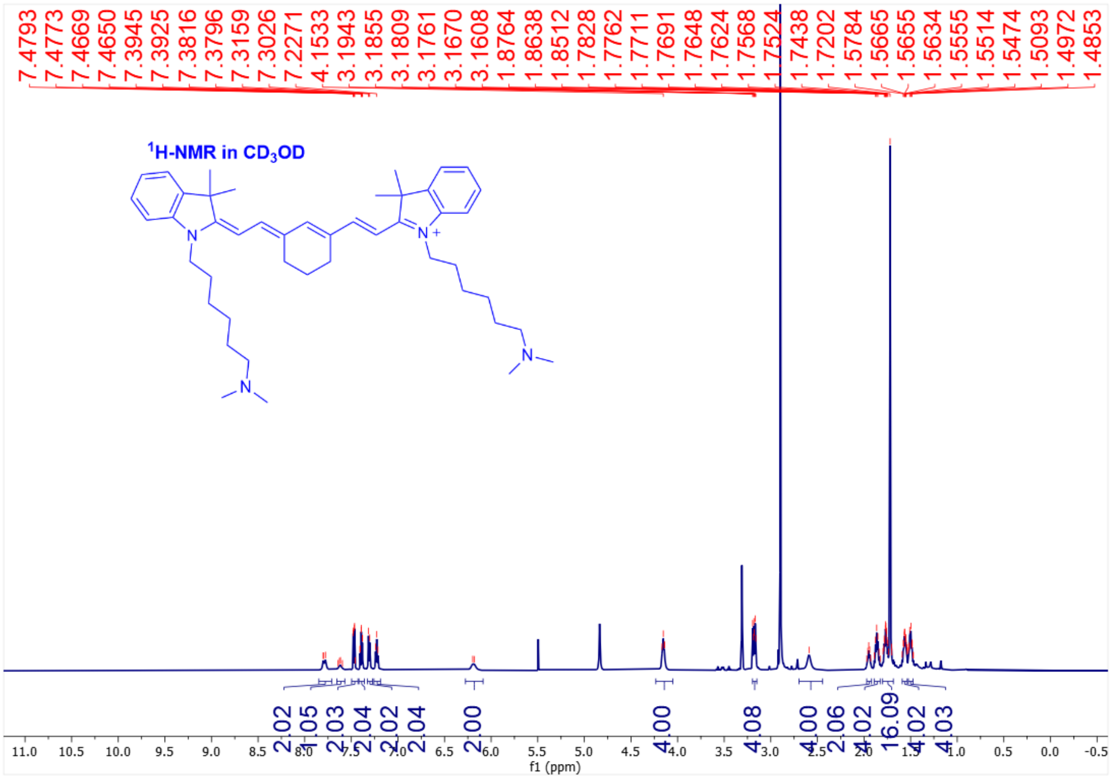


$^{13}$C-NMR spectrum of 4o

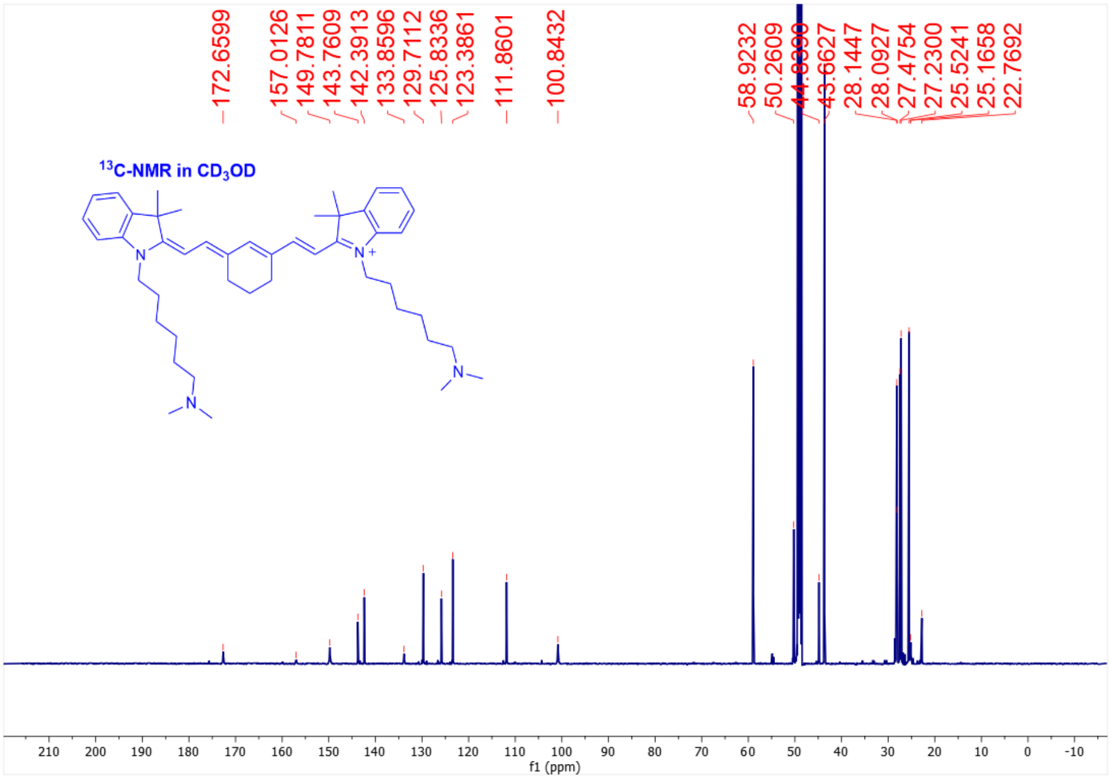

**$^{1}$H-NMR spectrum of 4p**

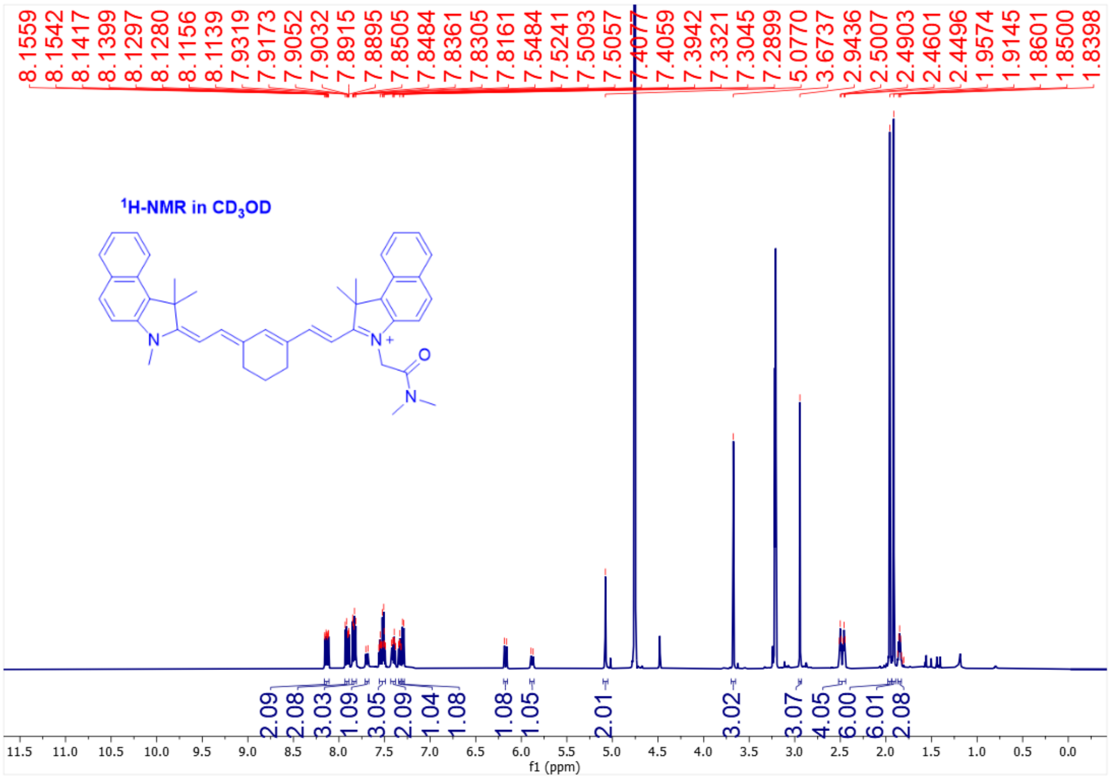

**$^{13}$C-NMR spectrum of 4p**

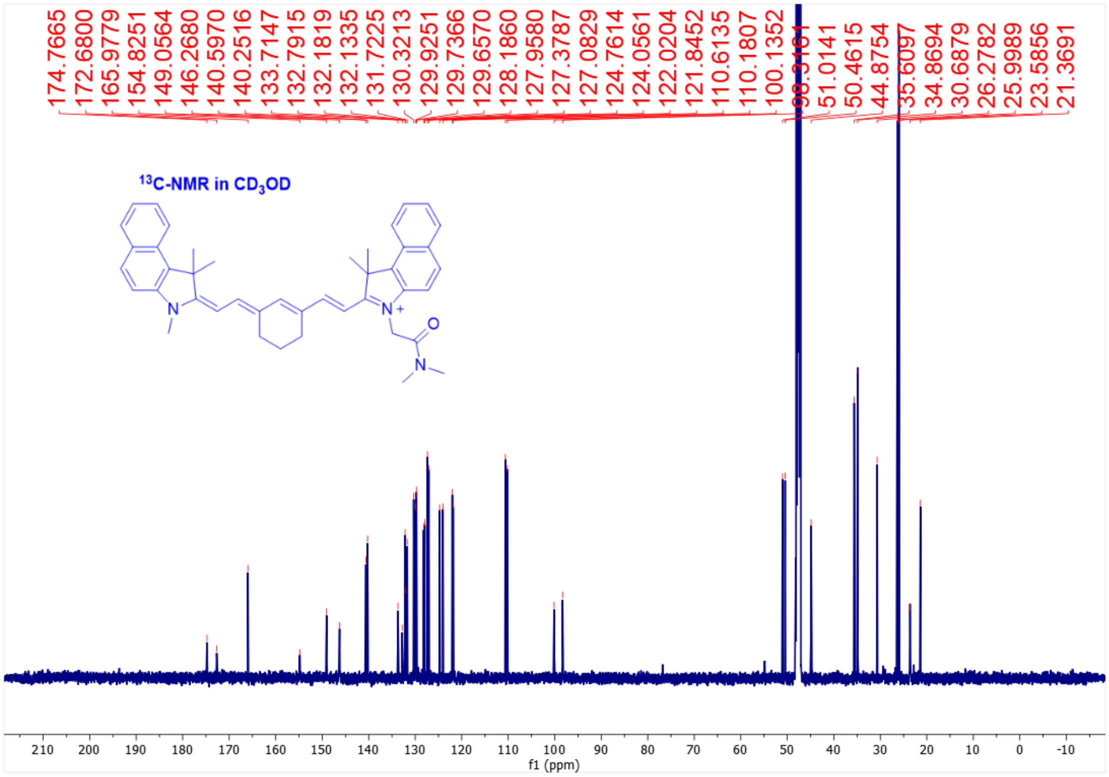

$^{1}$H-NMR spectrum of 4q

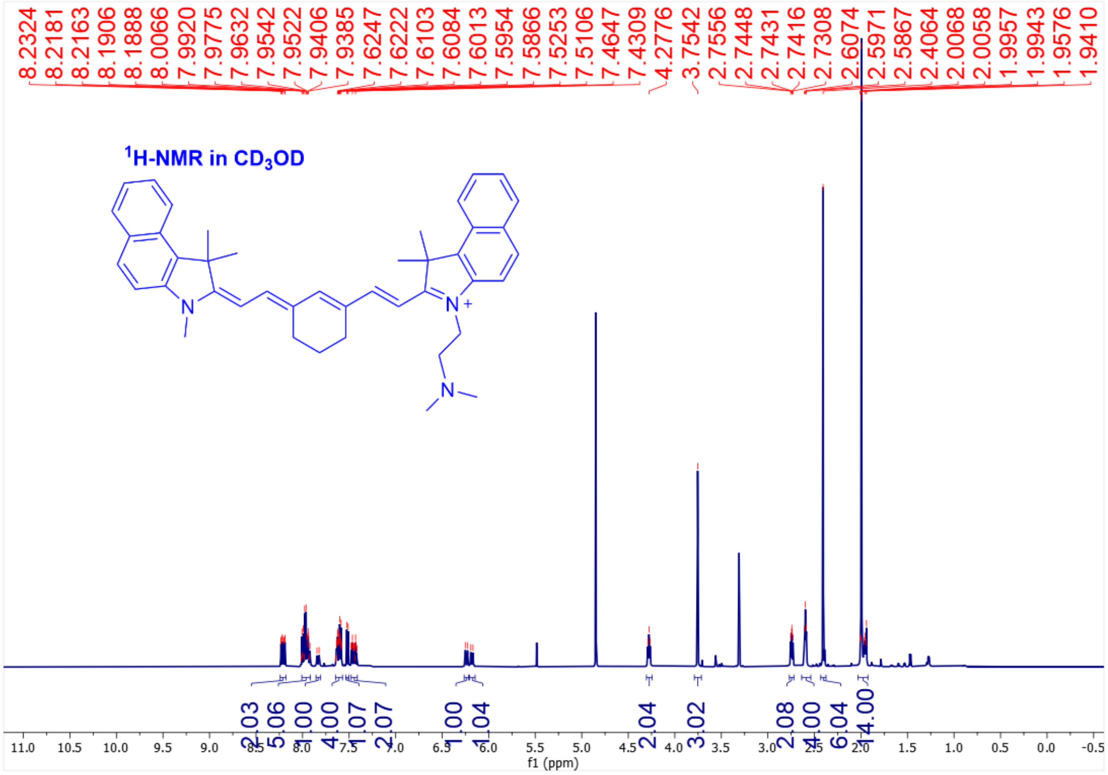


$^{13}$C-NMR spectrum of 4q

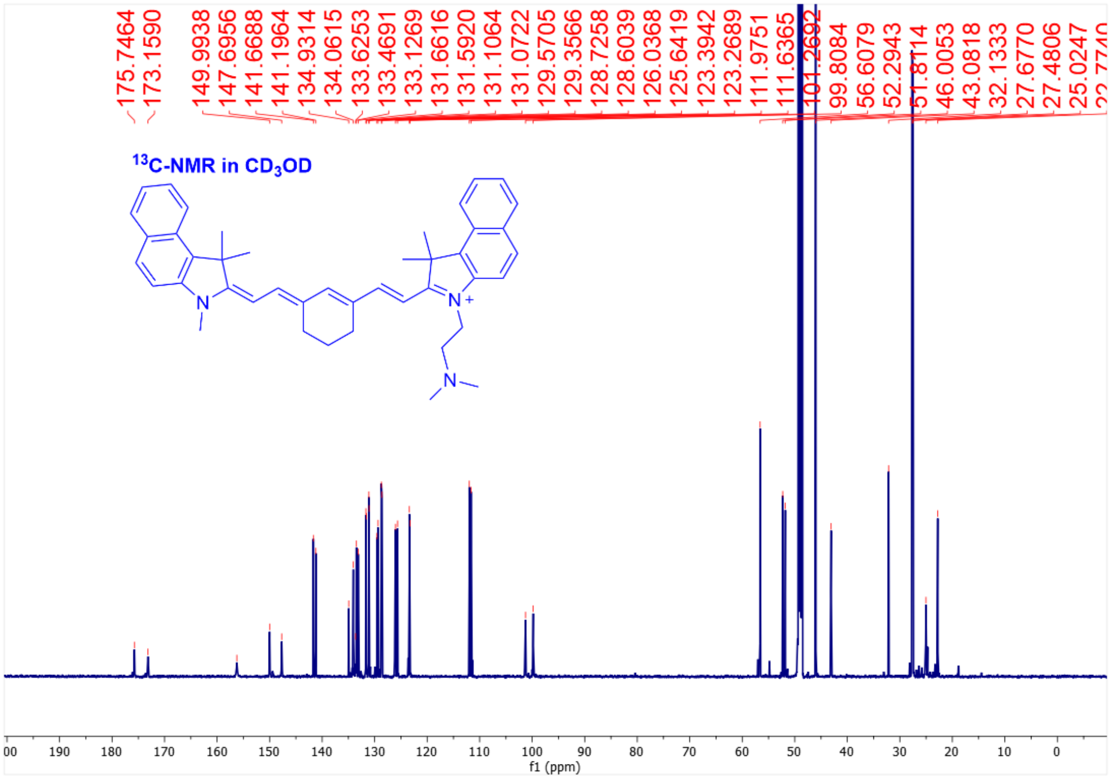

**$^{1}$H-NMR spectrum of 4r**

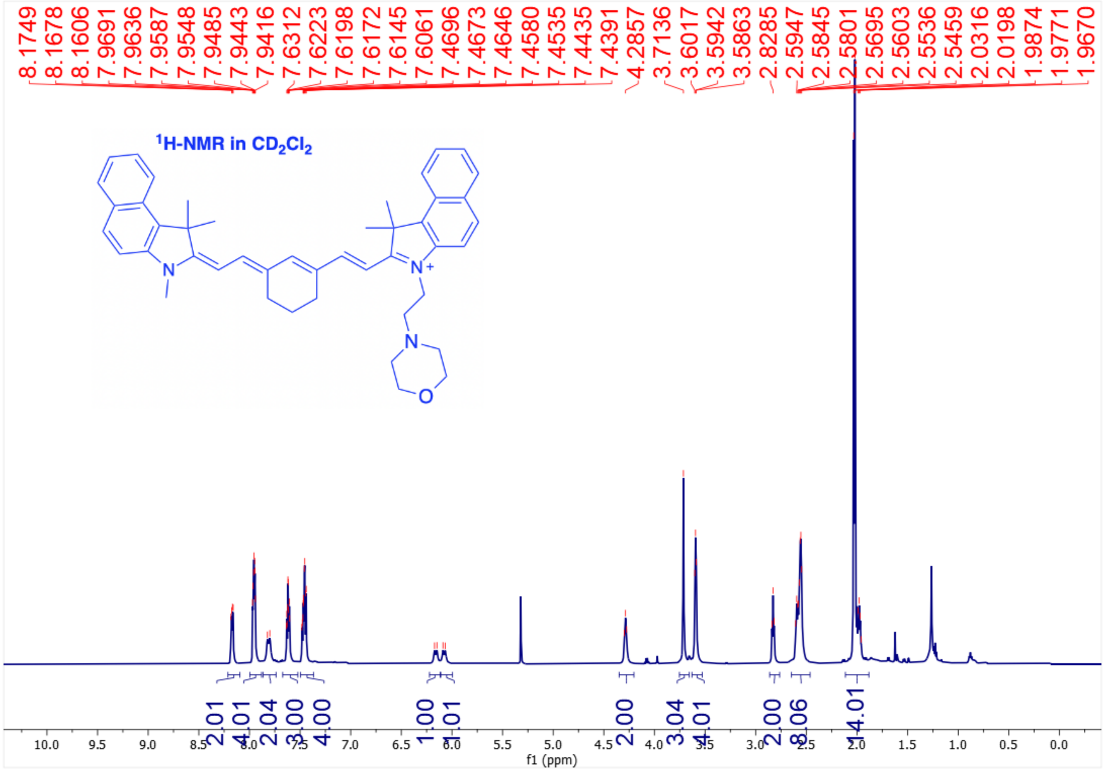

**$^{13}$C-NMR spectrum of 4r**

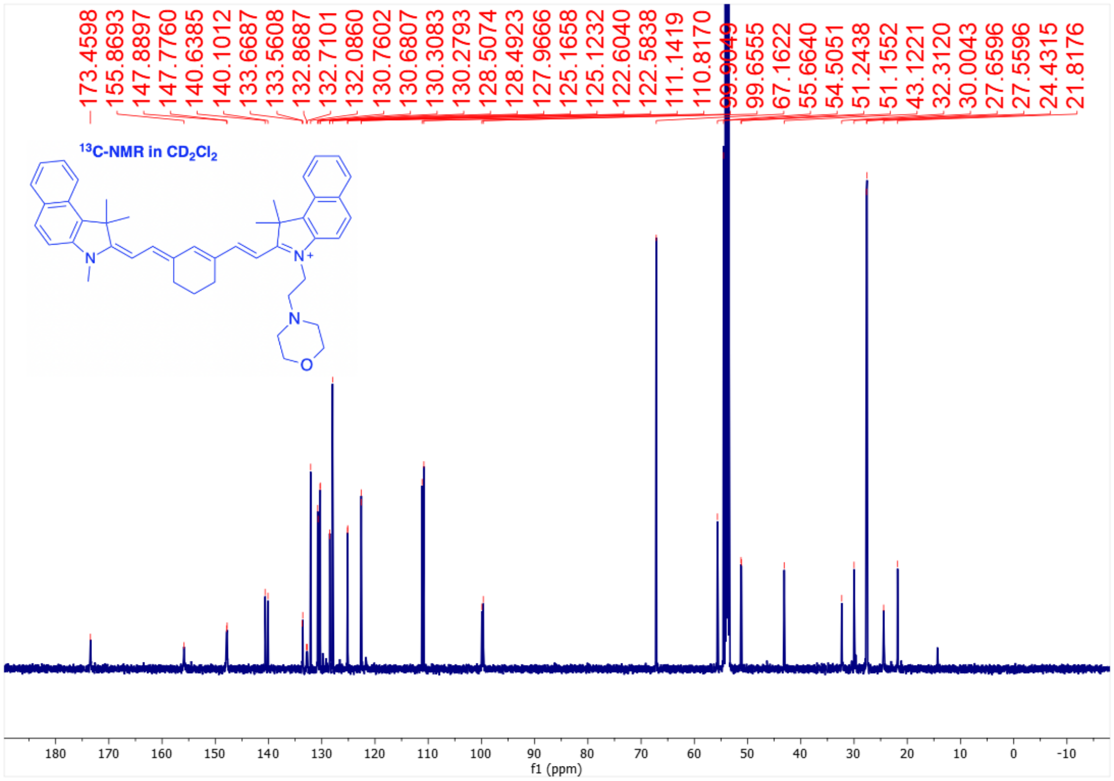

### $^{1}$H-NMR spectrum of 4s

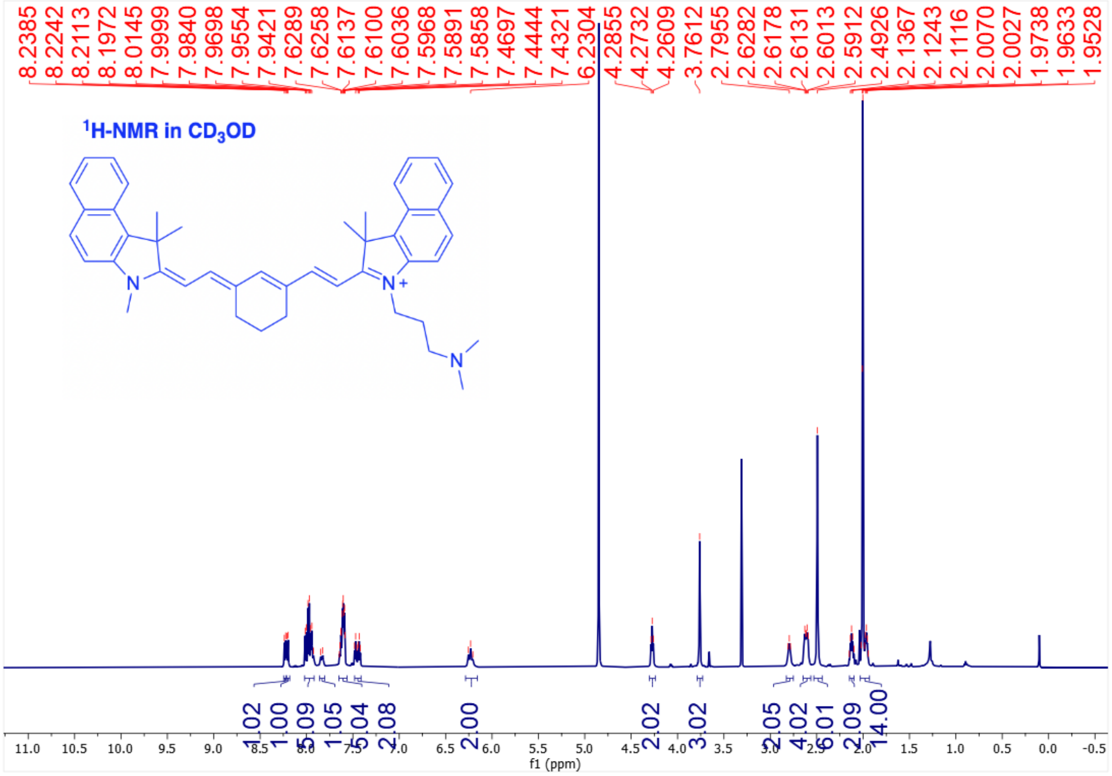


### $^{13}$C-NMR spectrum of 4s

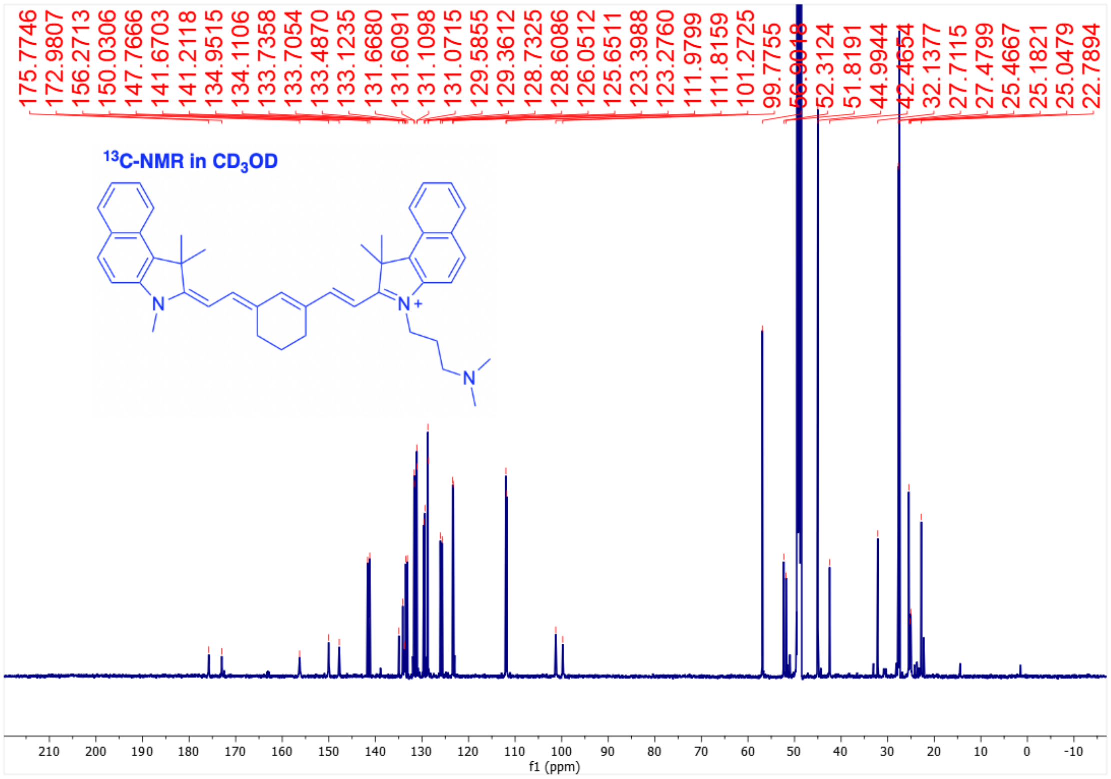

**$^{1}$H-NMR spectrum of 4t**

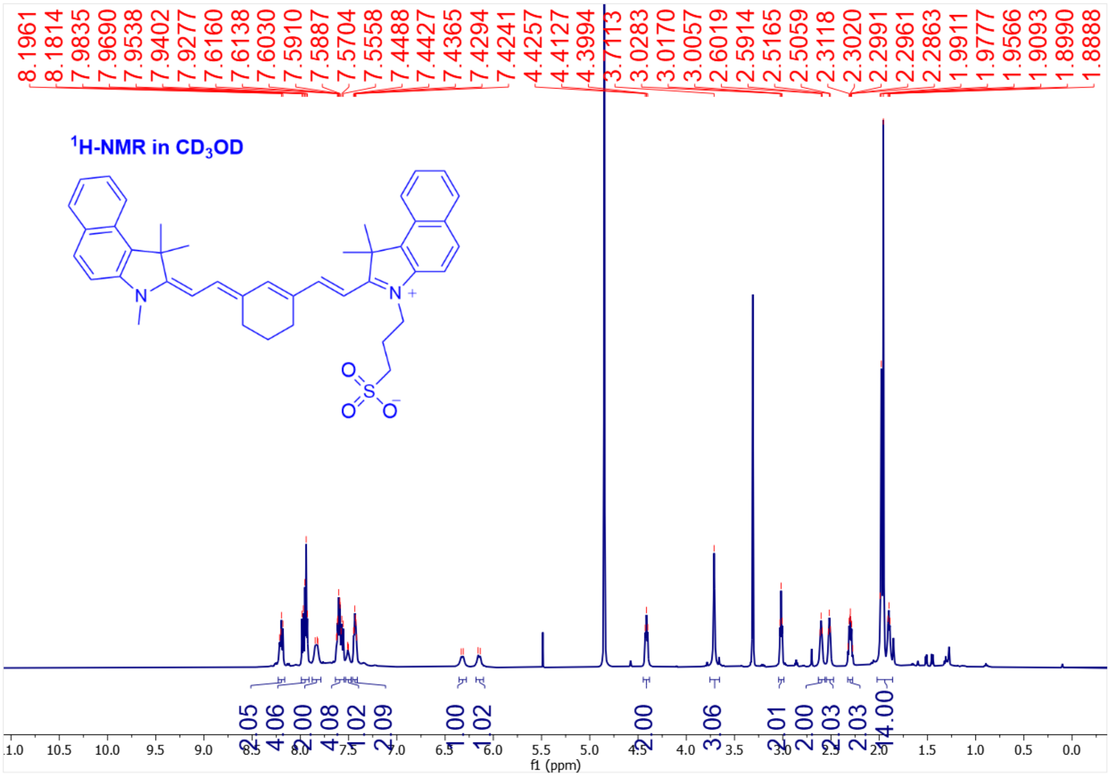


**$^{13}$C-NMR spectrum of 4t**

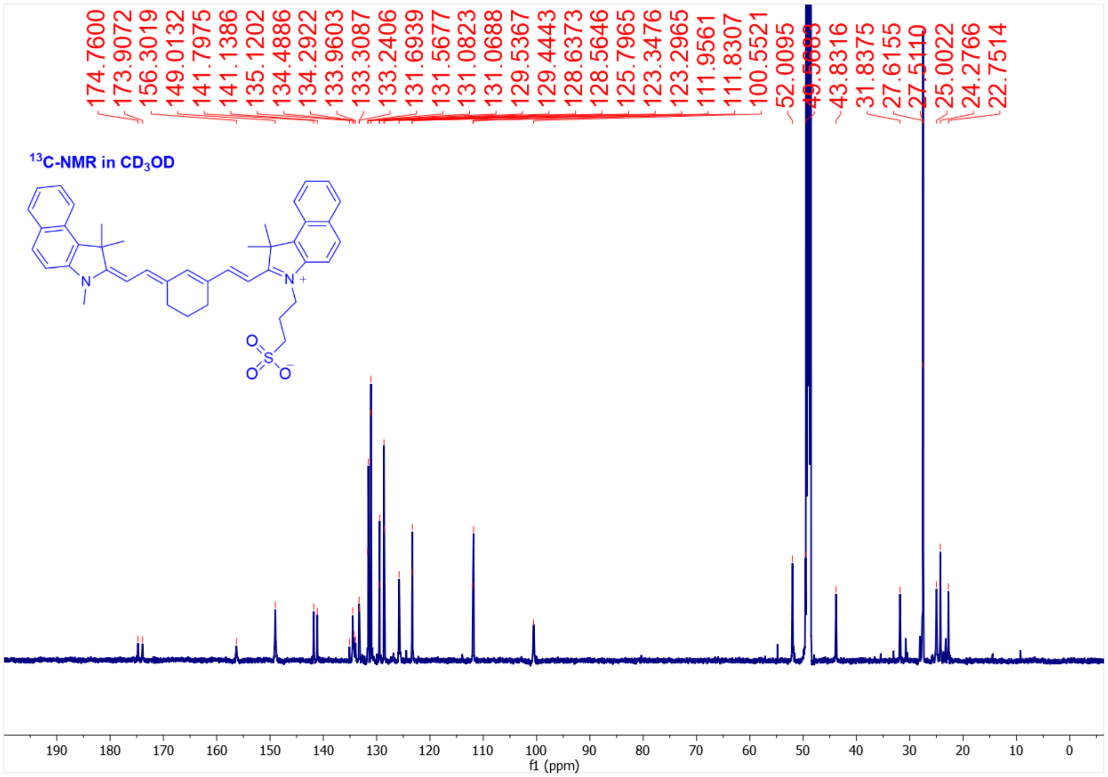

**$^{1}$H-NMR spectrum of 4u**

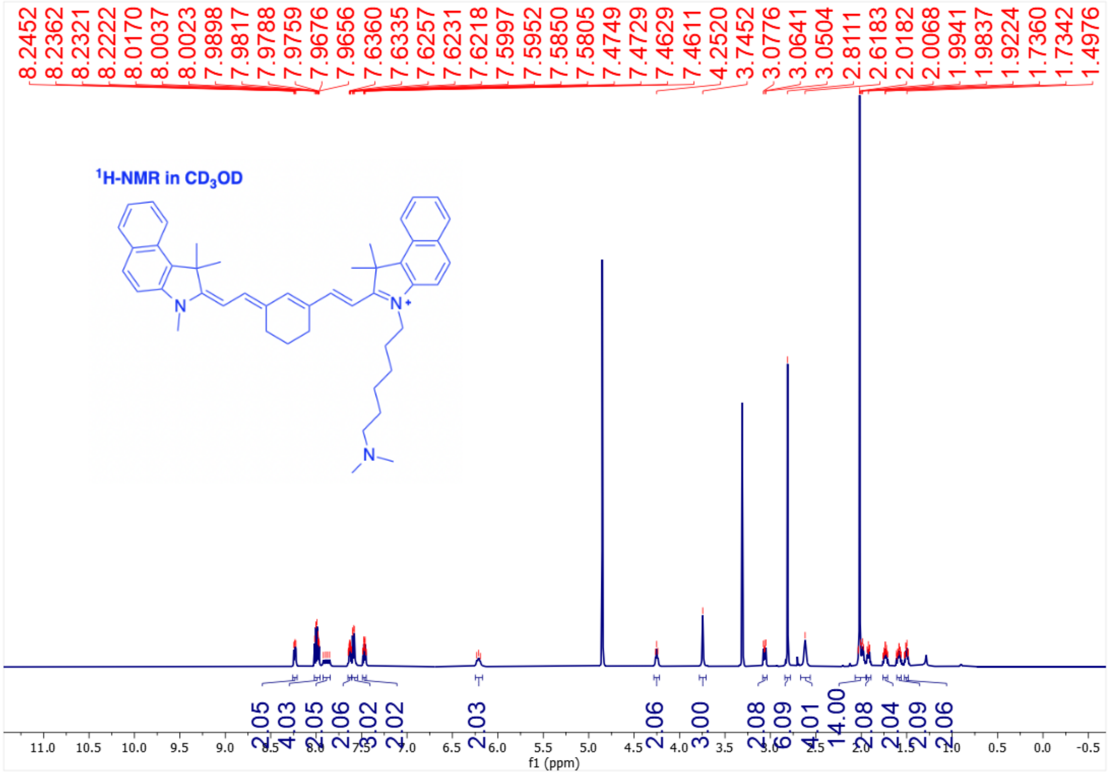

**$^{13}$C-NMR spectrum of 4u**

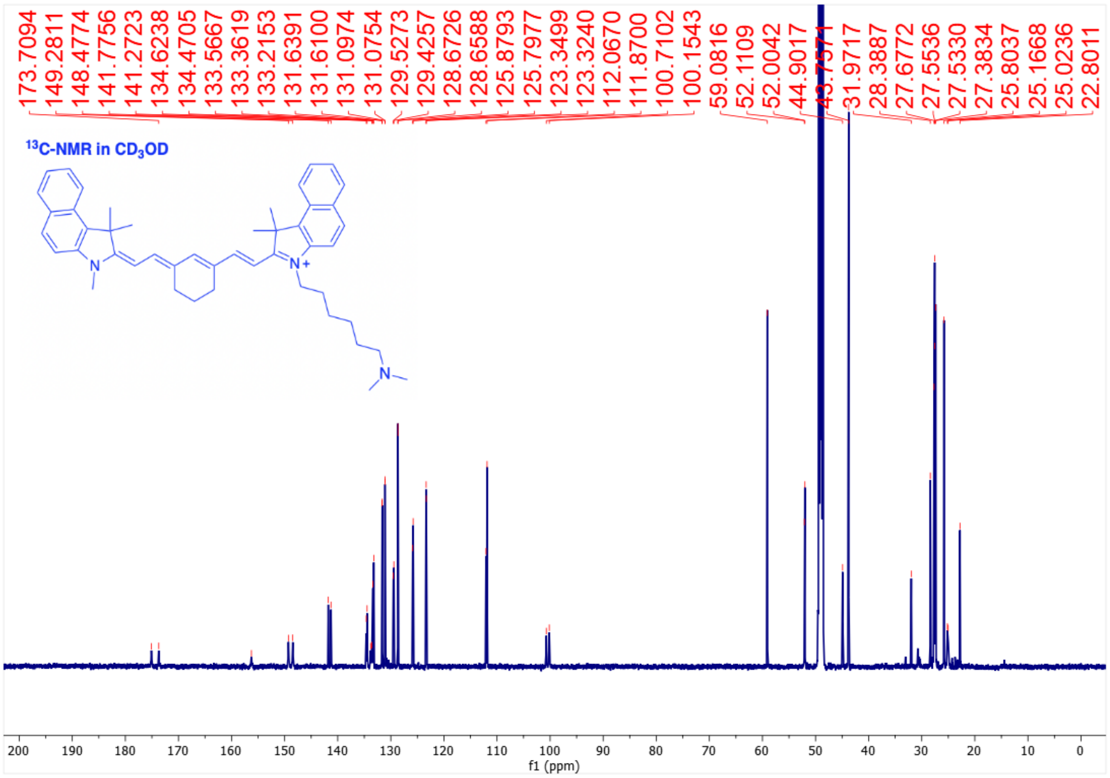

**$^{1}$H-NMR spectrum of 4v**

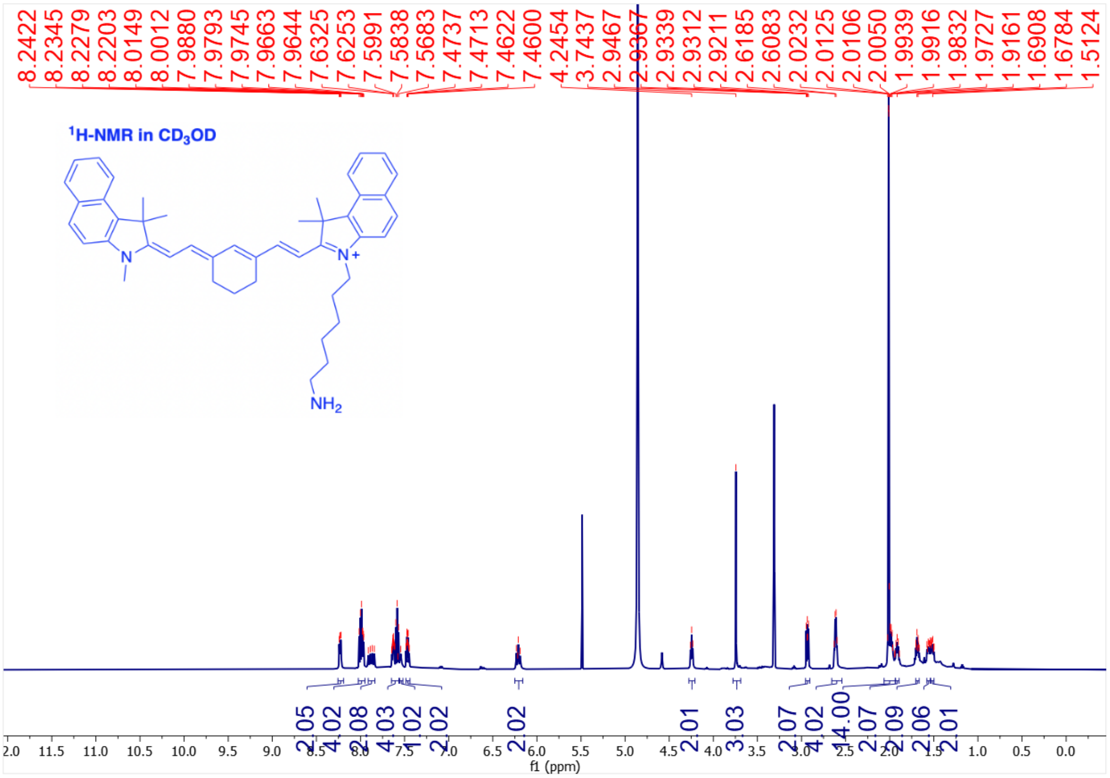

**$^{13}$C-NMR spectrum of 4v**

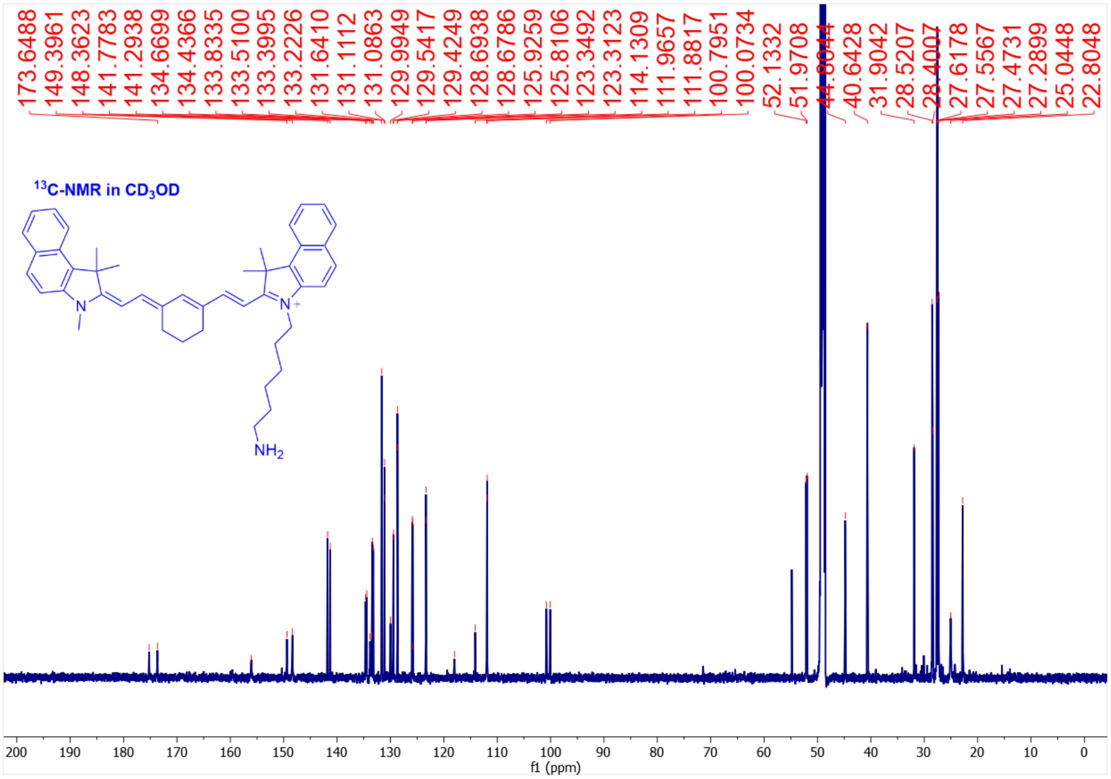

**$^{1}$H-NMR spectrum of 4w**

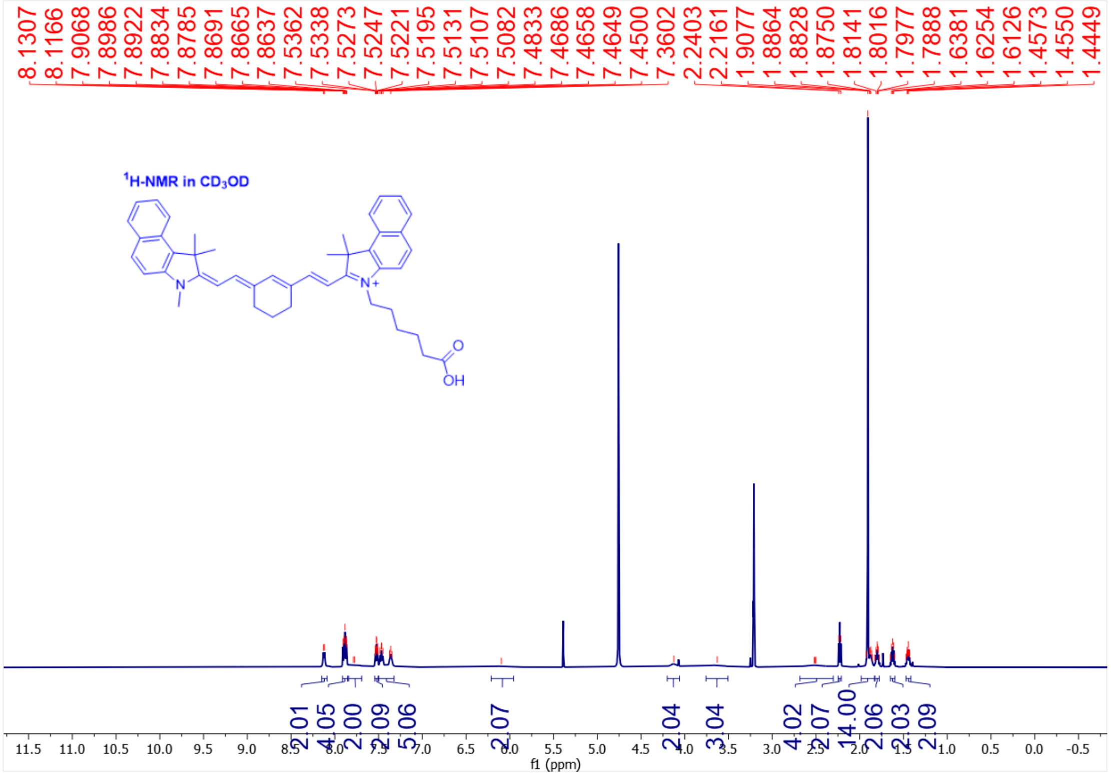


**$^{13}$C-NMR spectrum of 4w**

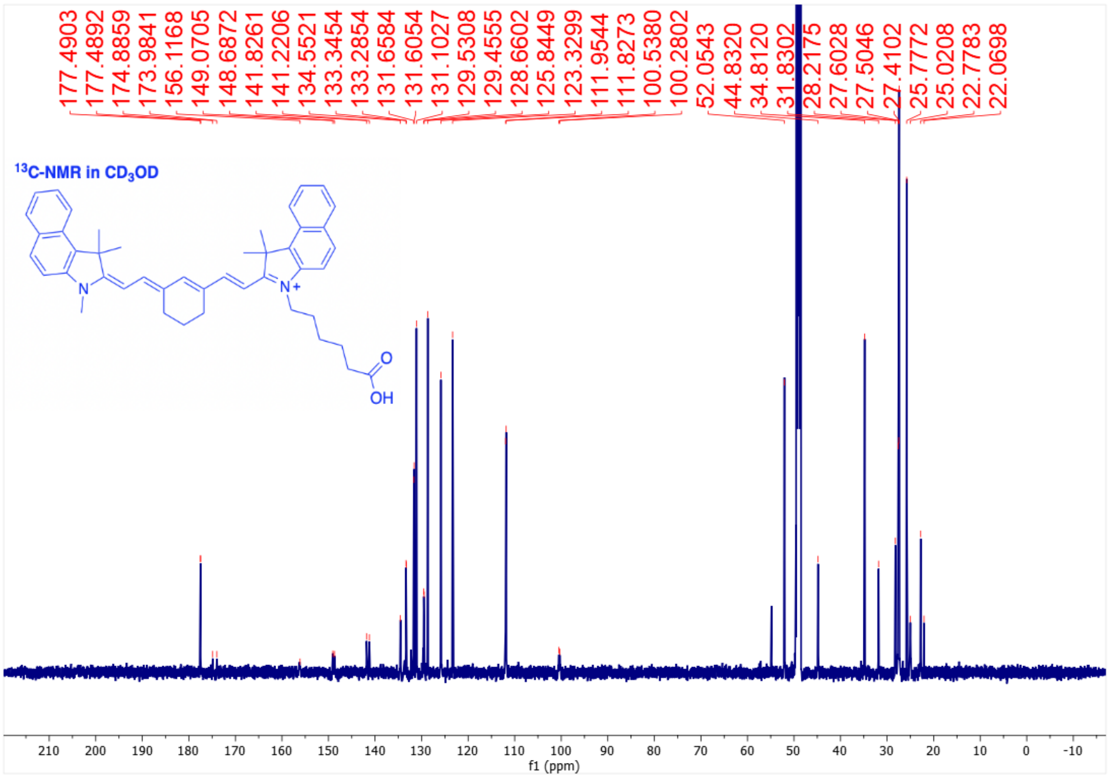

## $^{1}$H-NMR spectrum of 4x

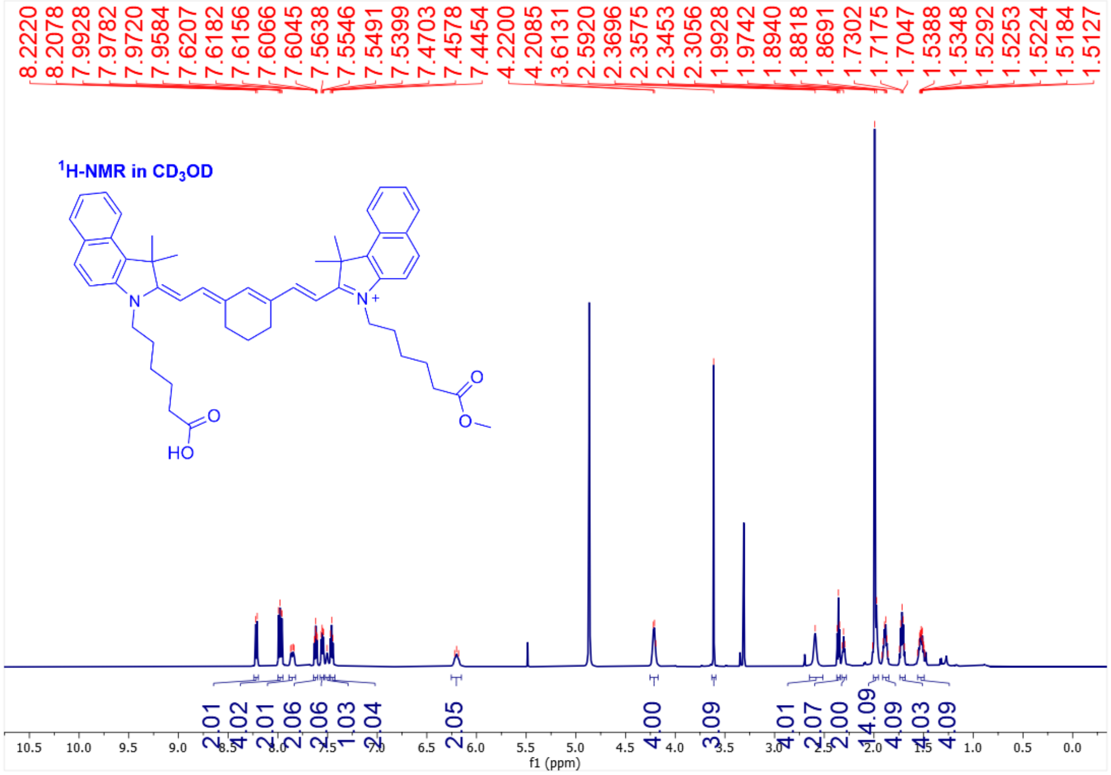


## $^{13}$C-NMR spectrum of 4x

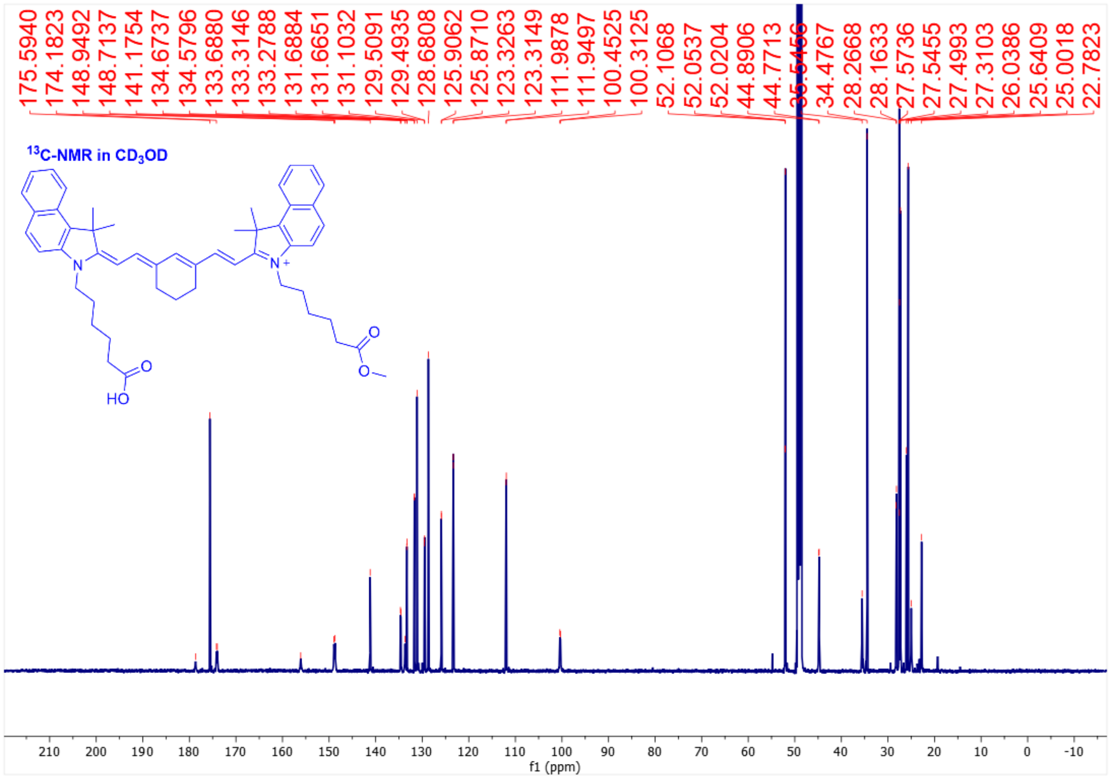

**$^{1}$H-NMR spectrum of 4y**

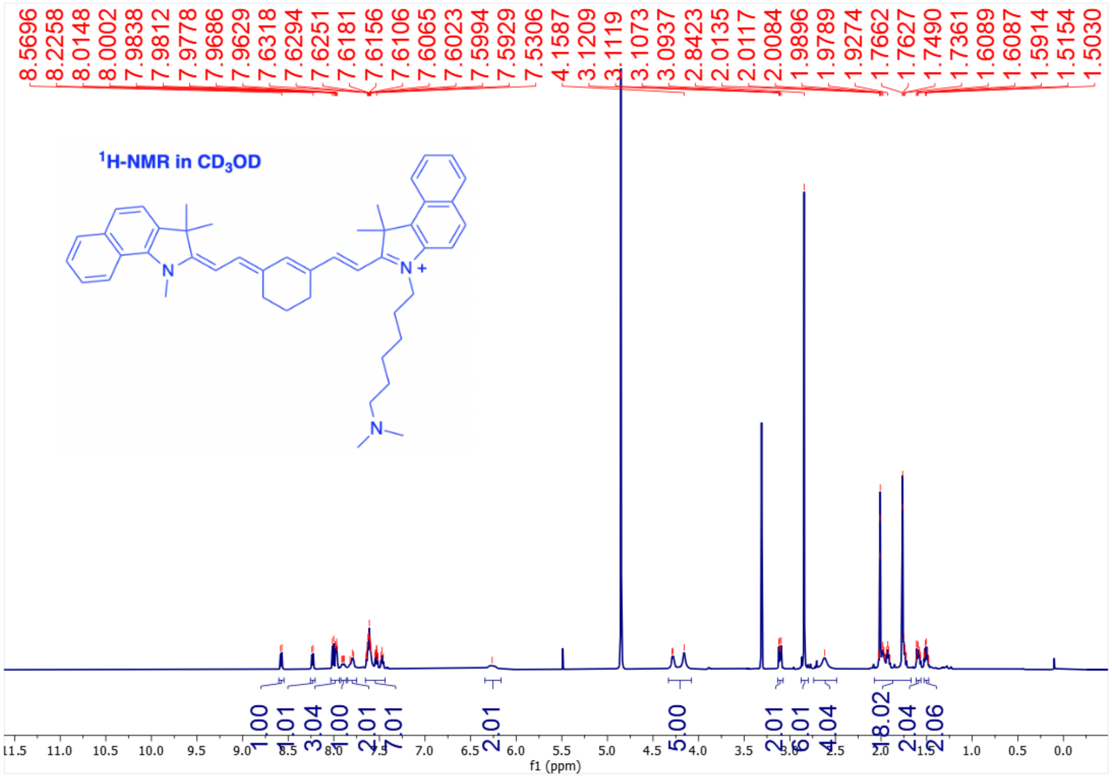


**$^{13}$C-NMR spectrum of 4x**

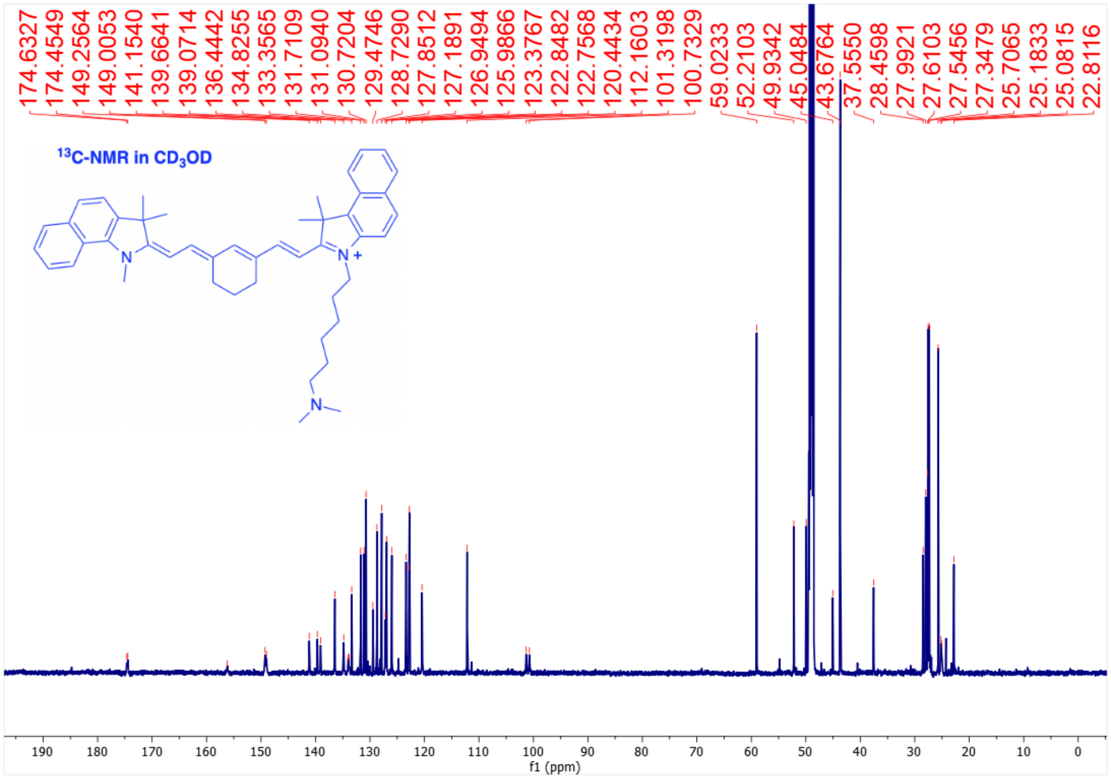

**$^{1}$H-NMR spectrum of 4z**

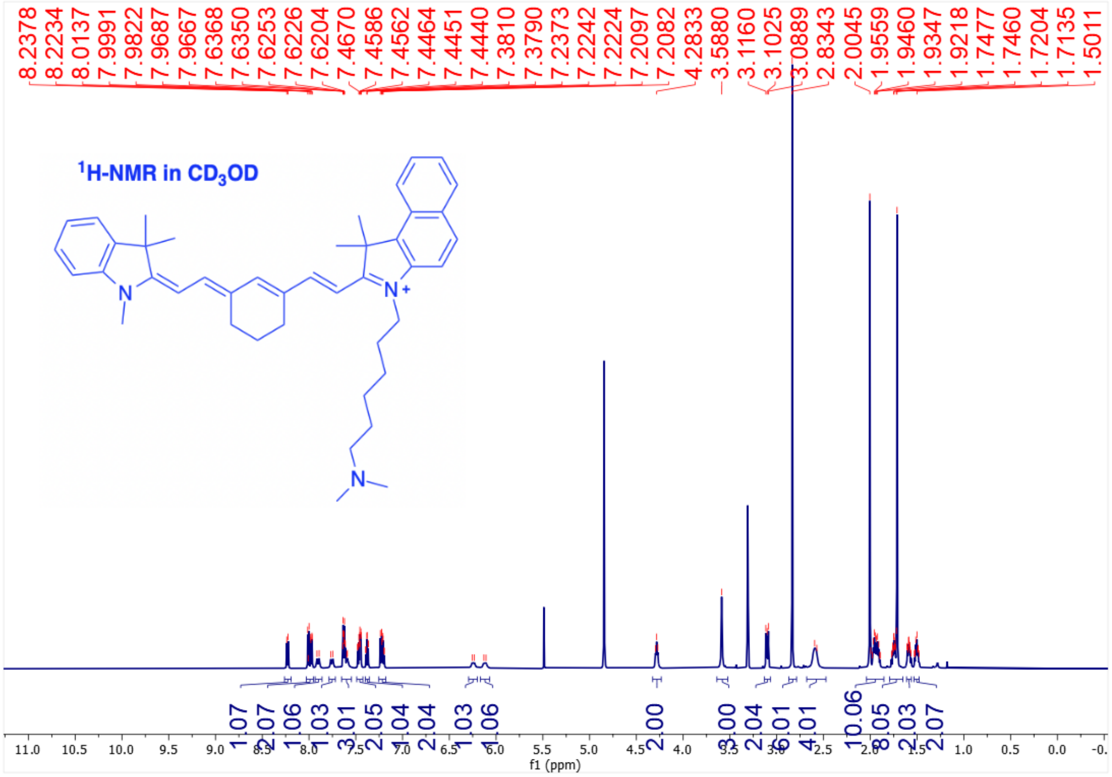

**$^{13}$C-NMR spectrum of 4x**

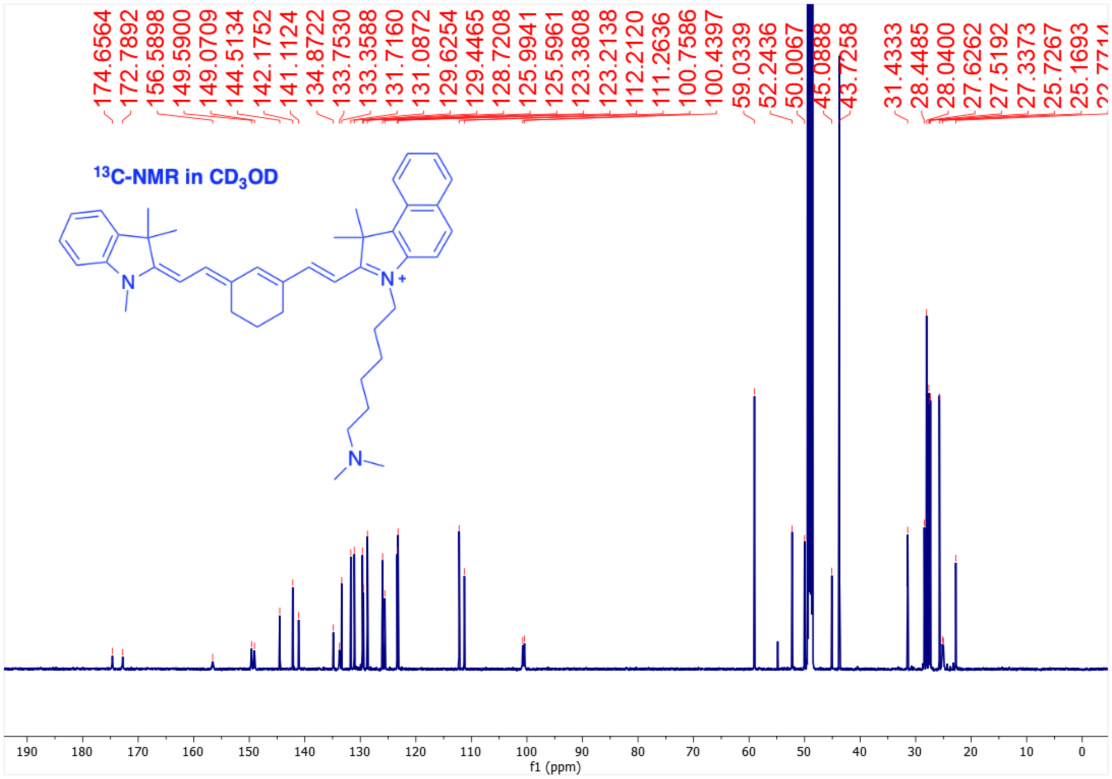

**$^{1}$H-NMR spectrum of 4aa**

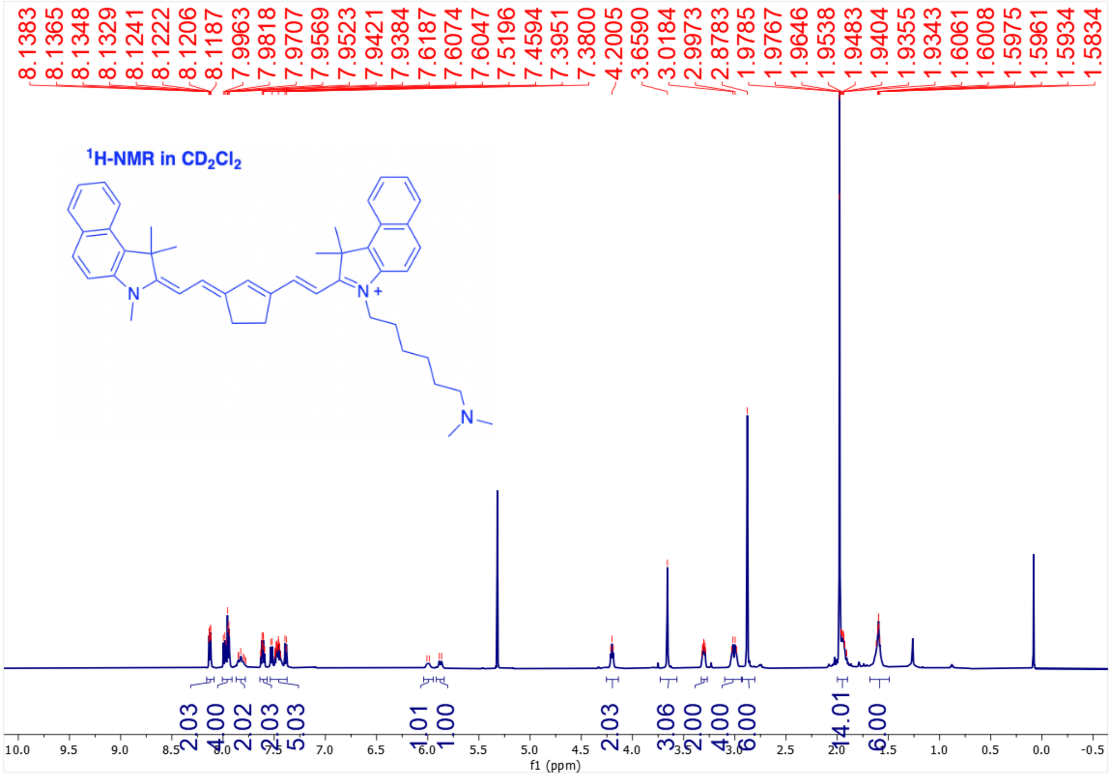

**$^{13}$C-NMR spectrum of 4aa**

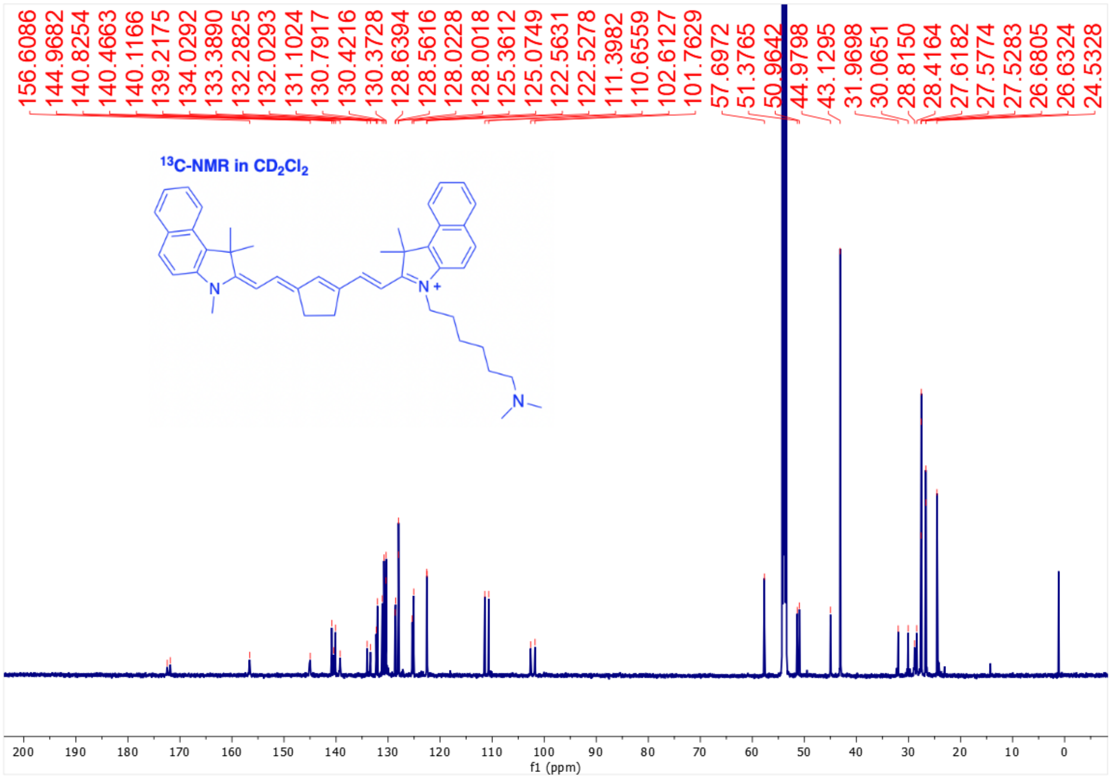

**$^1$H-NMR spectrum of 4ab**

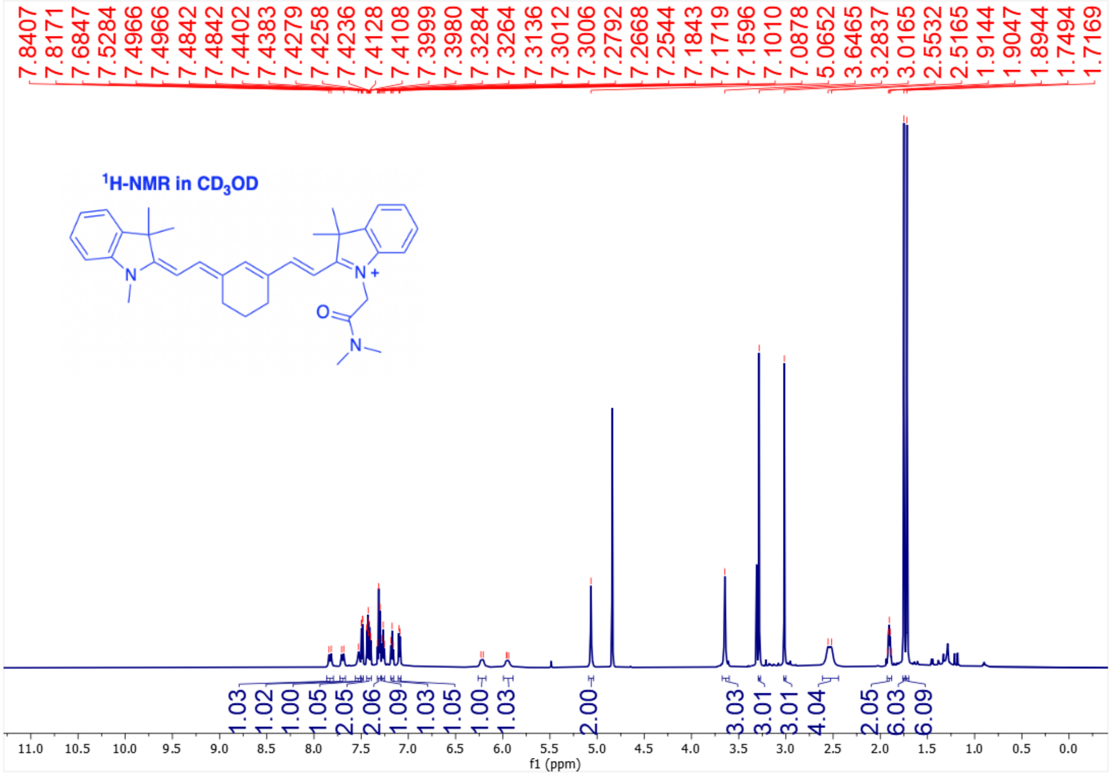


**$^{13}$C-NMR spectrum of 4ab**

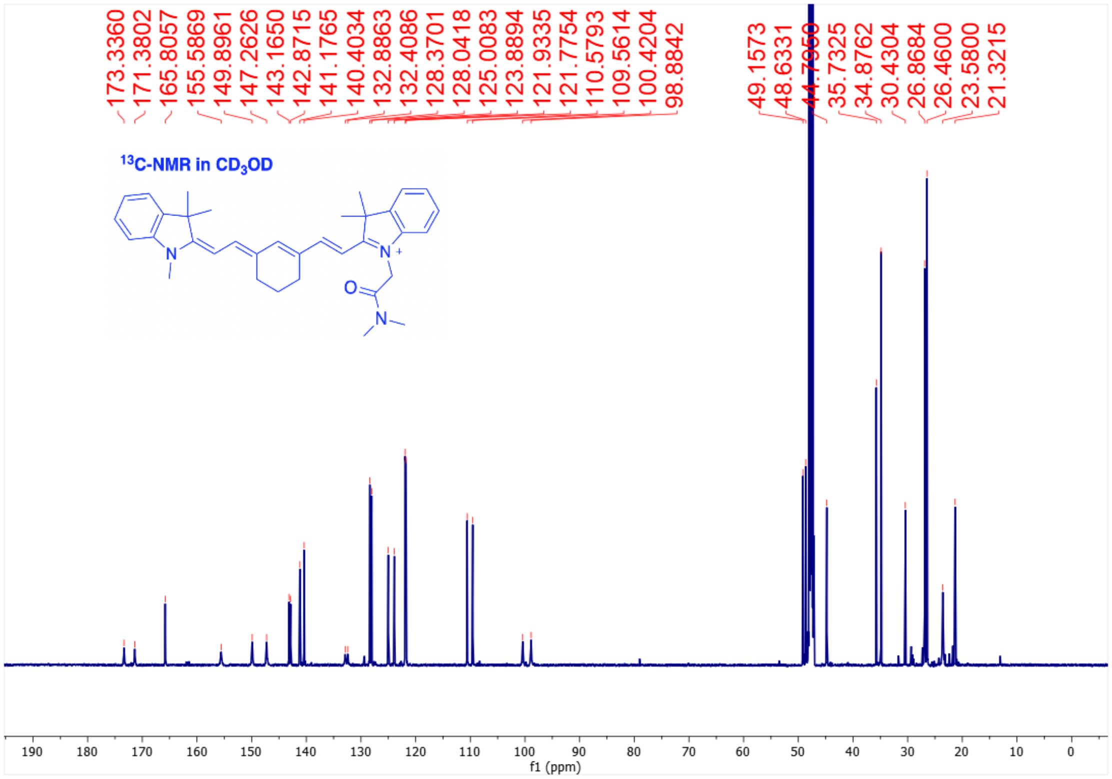

**$^{1}$H-NMR spectrum of 4ac**

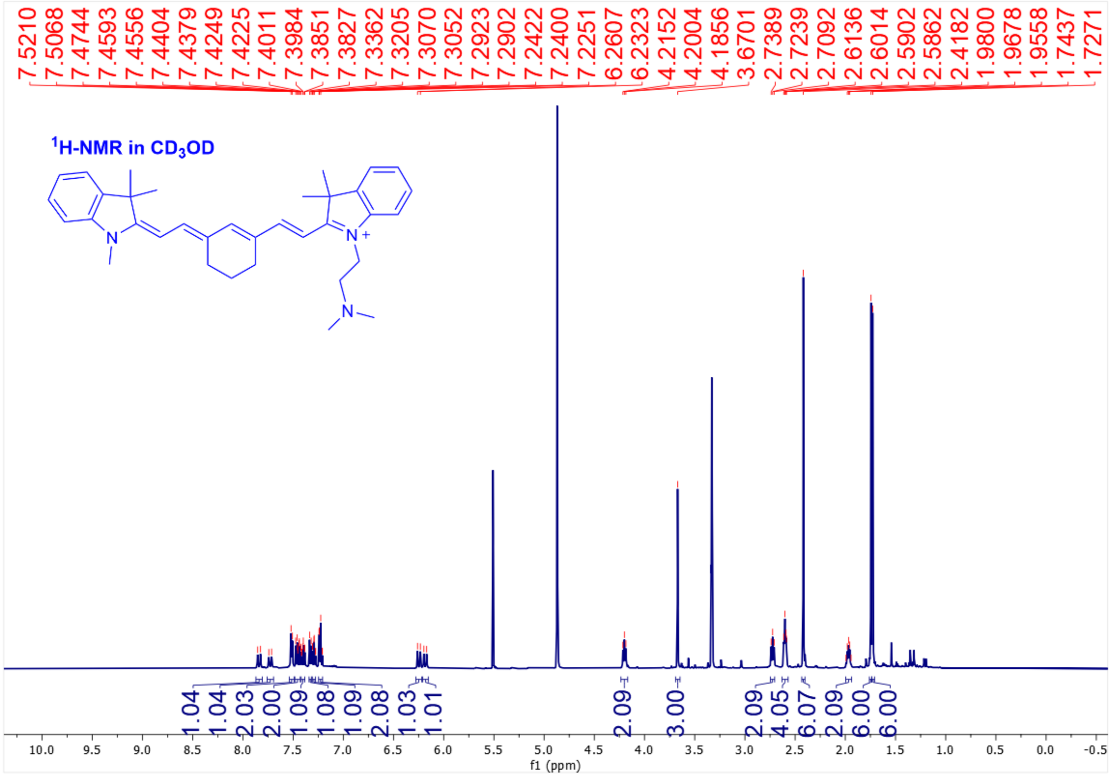


**$^{13}$C-NMR spectrum of 4ac**

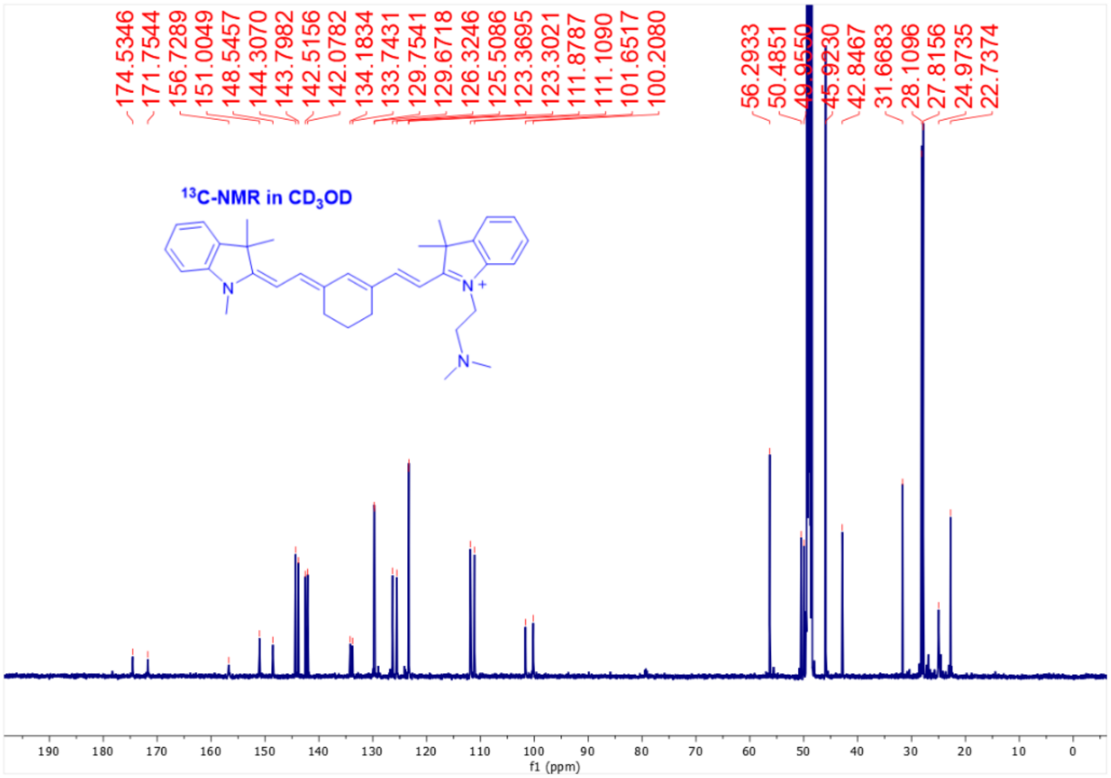

**$^{1}$H-NMR spectrum of 4ad**

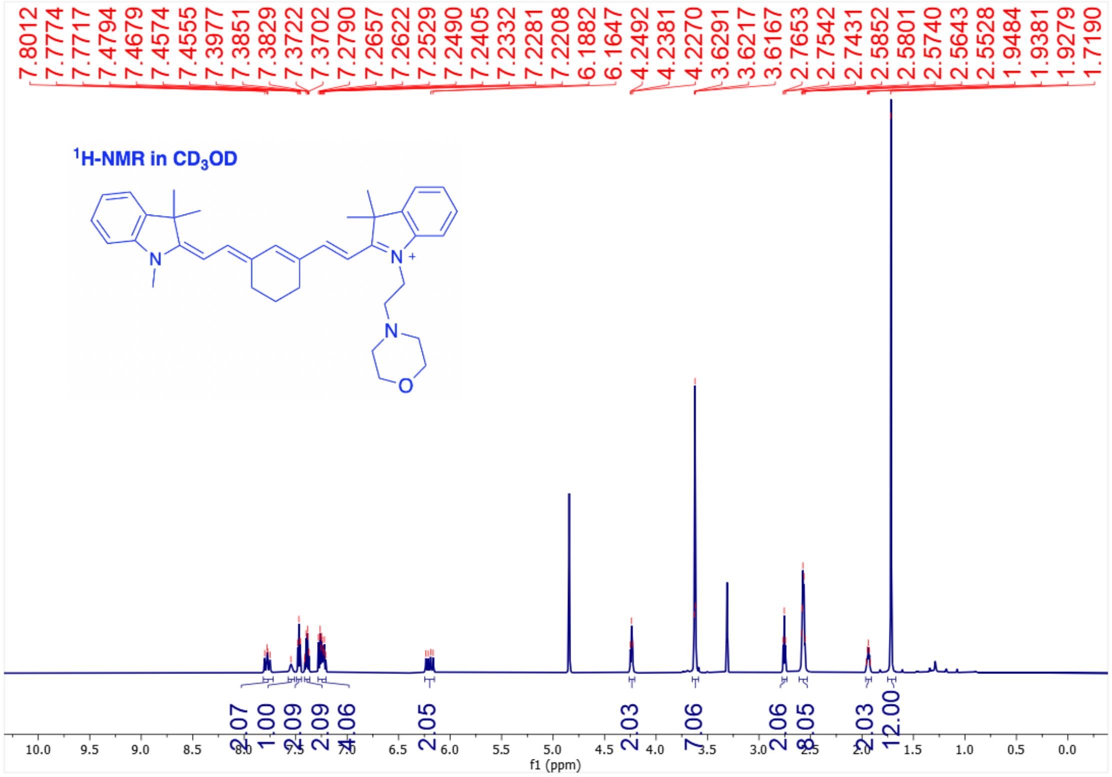

**$^{13}$C-NMR spectrum of 4ad**

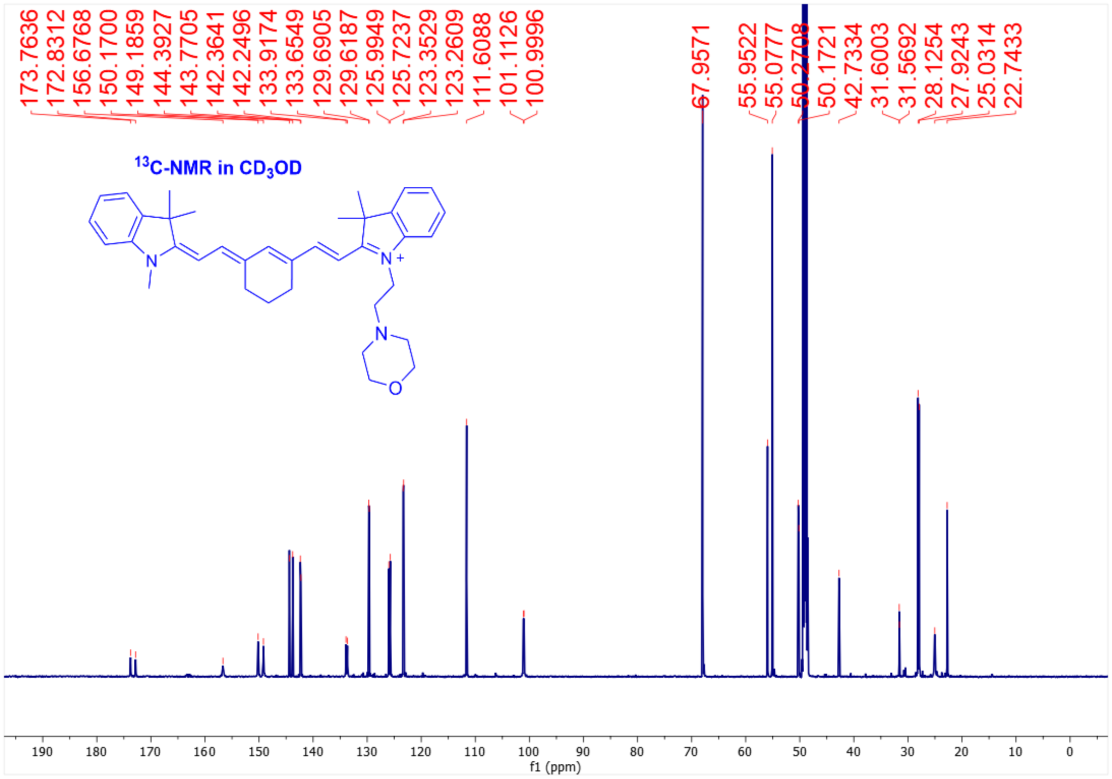

**$^{1}$H-NMR spectrum of 4ae**

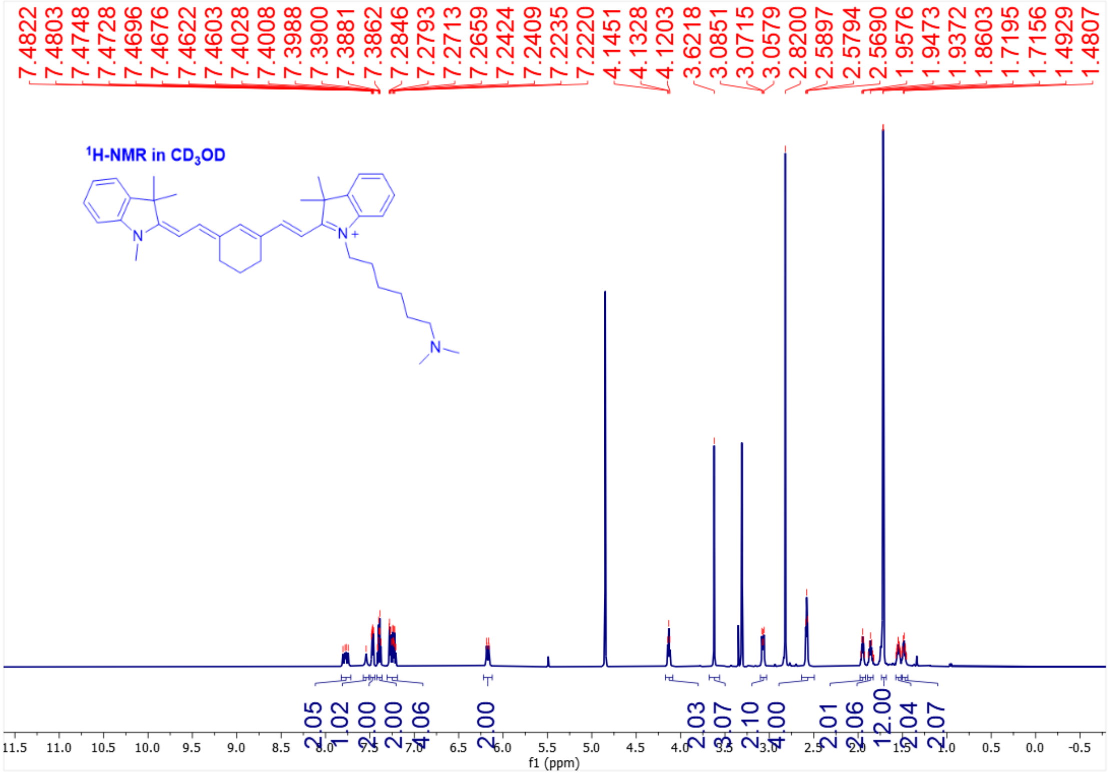

**$^{13}$C-NMR spectrum of 4ae**

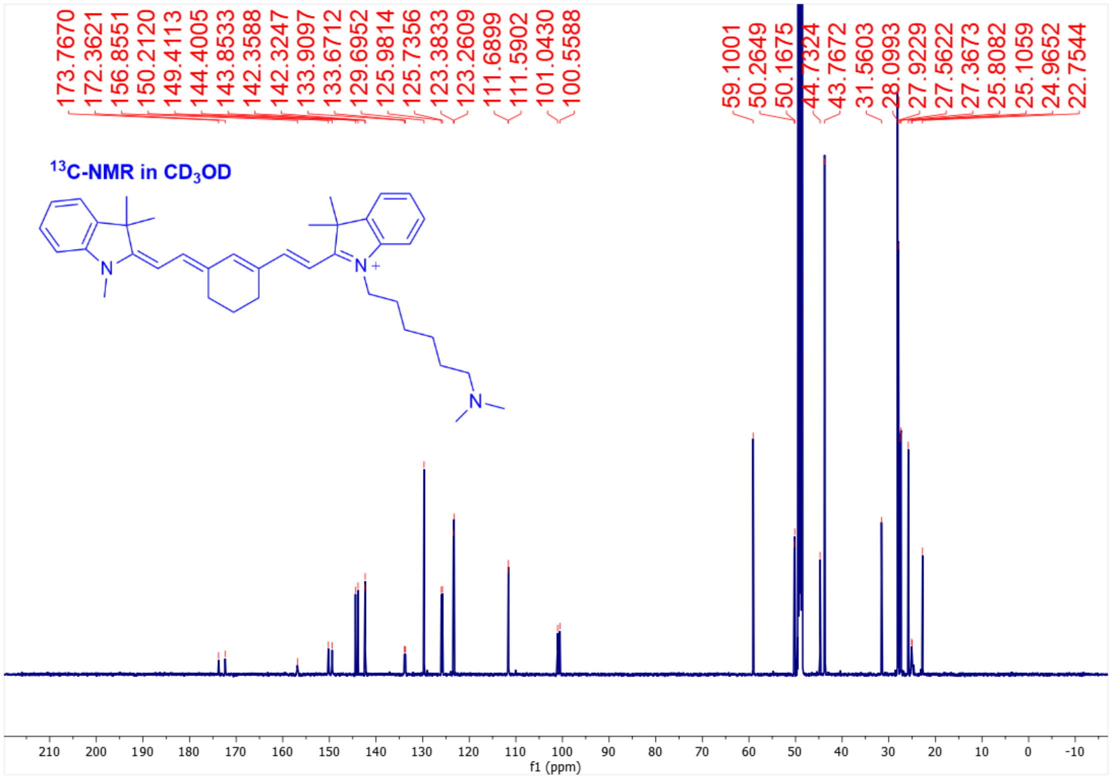

**$^{1}$H-NMR spectrum of 4af**

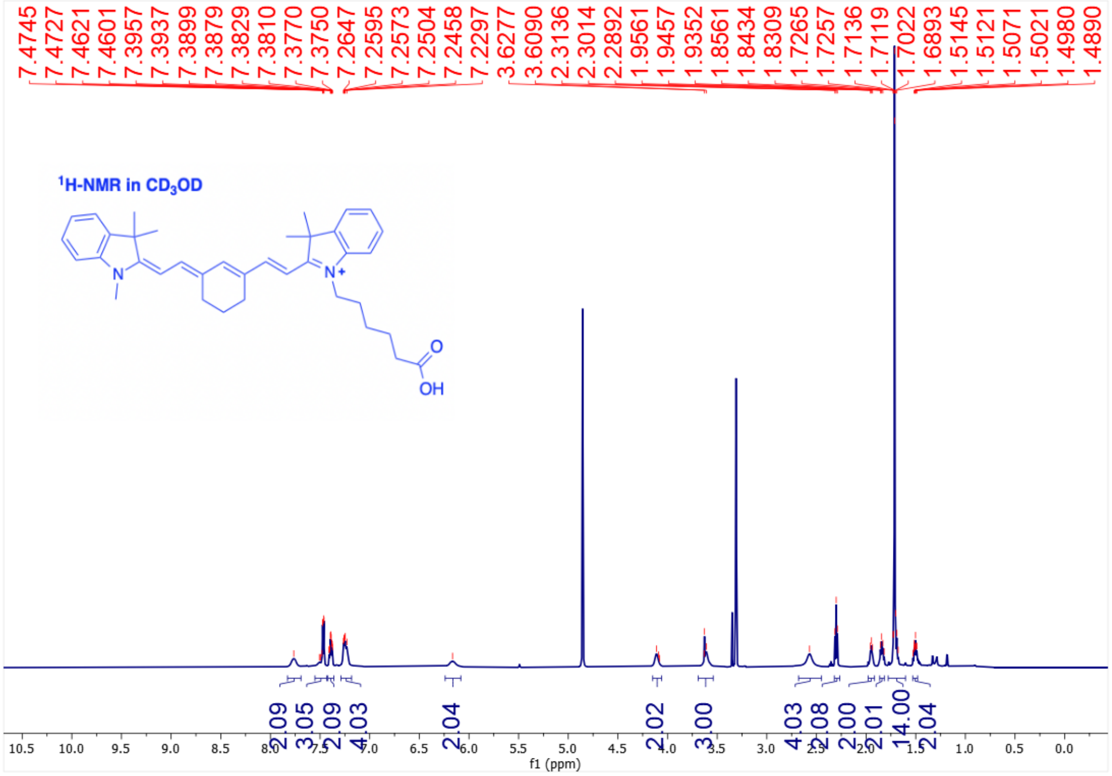

**$^{13}$C-NMR spectrum of 4af**

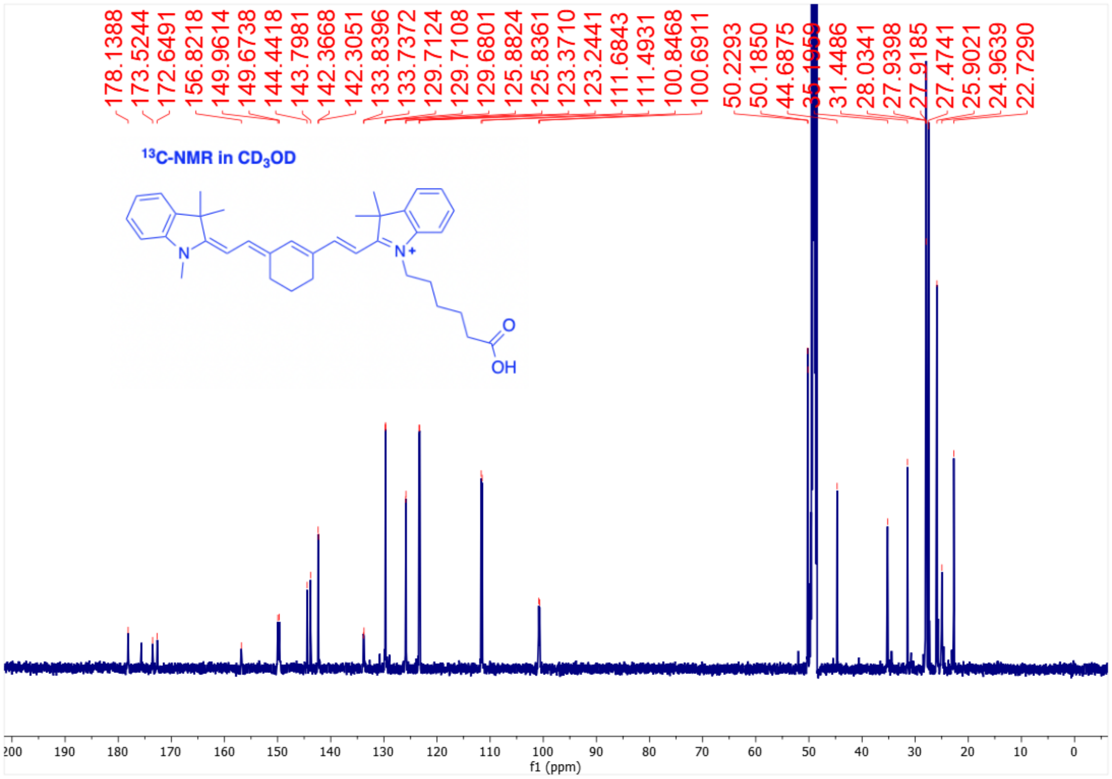

Supplement: Supplementary file 1 — Supporting Information [file ADVS-11-2405965-s001.pdf]
